# Puerarin attenuates myocardial ischemic injury and endoplasmic reticulum stress by upregulating the Mzb1 signal pathway

**DOI:** 10.3389/fphar.2024.1442831

**Published:** 2024-08-13

**Authors:** Jiaojiao Xue, Haolin Ren, Qi Zhang, Jing Gu, Qian Xu, Jiaxi Sun, Lu Zhang, Ming-Sheng Zhou

**Affiliations:** ^1^ Science and Experiment Research Center, Shenyang Medical College, Shenyang, China; ^2^ School of Basic Medicine, Shenyang Medical College, Shenyang, China; ^3^ Department of Pathology, Women and Children’s Hospital, School of Medicine, Xiamen University, Xiamen, Fujian, China; ^4^ Department of Radiology, The First Affiliated Hospital of Nanjing Medical University, Nanjing, China

**Keywords:** puerarin, acute myocardial infarction, endoplasmic reticulum, mitochondria function, Mzb1

## Abstract

**Objective:**

This study investigated the role of Mzb1 in puerarin protection against heart injury and dysfunction in acute myocardial infarction (AMI) mice.

**Methods:**

C57BL/6 mice were pretreated with and without puerarin at doses of 50 mg/kg and 100 mg/kg for 14 days before establishing the AMI model. An AMI model was induced by ligating the left descending anterior coronary artery, and AC16 cardiomyocytes were treated with H_2_O_2_ *in vitro*. Echocardiography was performed to measure cardiac function. DHE staining, nicotinamide adenine dinucleotide phosphate (NADPH) oxidase assay, and DCFH-DA oxidative fluorescence staining were used to determine reactive oxygen species (ROS) production *in vivo* and *in vitro*. Bioinformatics analysis was used to predict potential upstream transcription factors of Mzb1.

**Results:**

Puerarin dose-dependently reduced myocardial infarction area and injury, accompanied by the improvement of cardiac function in AMI mice. AMI mice manifested an increase in myocardial oxidative stress, endoplasmic reticulum (ER) stress, apoptosis, and mitochondrial biogenesis dysfunction, which were inhibited by pretreatment with puerarin. Puerarin also prevented Mzb1 downregulation in the hearts of AMI mice or H_2_O_2_-treated AC16 cells. Consistent with the *in vivo* findings, puerarin inhibited H_2_O_2_-induced cardiomyocyte apoptosis, ER stress, and mitochondrial dysfunction, which were attenuated by siRNA Mzb1. Furthermore, the JASPAR website predicted that KLF4 may be a transcription factor for Mzb1. The expression of KLF4 was partially reversed by puerarin in the cardiomyocyte injury model, and KLF4 inhibitor (kenpaullone) inhibited Mzb1 expression and affected its function.

**Conclusion:**

These results suggest that puerarin can protect against cardiac injury by attenuating oxidative stress and endoplasmic reticulum stress through upregulating the KLF4/Mzb1 pathway and that puerarin may expand our armamentarium for the prevention and treatment of ischemic heart diseases.

## 1 Introduction

Acute myocardial infarction (AMI) is a clinical syndrome characterized by the interruption of myocardial blood supply caused by sudden occlusion of coronary arteries (Damluji et al., 2021; Dauerman and Ibanez, 2021). Although substantial advances have been made in the treatment of ischemic heart disease and AMI, such as timely coronary thrombolytic therapy, percutaneous coronary intervention, and coronary artery bypass (Hausenloy and Yellon, 2013; Bagai et al., 2014; McCarthy et al., 2018), Western medicine has not yet fully met the needs for treatment of ischemia heart disease. Some safe and effective traditional Chinese medicines may be an alternative strategy for the treatment of ischemic heart disease or AMI.

Ischemia heart disease or AMI is often associated with oxidative stress, endoplasmic reticulum (ER) stress, and mitochondrial dysfunction (Zou et al., 2019; Ramachandra et al., 2020; Souza-Neto et al., 2022; Zhang et al., 2022). The ER is a major site of protein synthesis, folding, and assembling. Ischemia increases reactive oxygen species (ROS) production in the myocardium, which may disturb ER homeostasis, leading to increased misfolded or unfolded protein accumulation, known as ER stress (Wang and Kaufman, 2016). Structurally, mitochondria and the ER are interconnected through the mitochondrial-associated endoplasmic reticulum membrane, and ER stress is one of the important factors that can induce mitochondrial dysfunction (Rowland and Voeltz, 2012; Wang et al., 2021). Emerging evidence has shown that ER stress-associated mitochondrial dysfunction plays a critical role in the pathogenesis of ischemia/reperfusion heart diseases (Dia et al., 2020; Yuan et al., 2020).

Marginal zone B and B1 cell-specific protein (Mzb1) is an ER-located protein. Mzb1 is originally identified as a molecular chaperon of glucose-regulated protein 94 (GRP94)-binding immunoglobulins protein (Bip) complex and a key molecule to regulate the assembly and secretion of multiple immunoglobulins (Rosenbaum et al., 2014; Andreani et al., 2018). Mzb1 has been implicated in a wide range of human diseases, including several chronic auto-immune diseases and cancers (Suzuki et al., 2019; Xiong et al., 2019; Wu et al., 2020). Recently, we have shown that AMI downregulates Mzb1 expression in the heart, and the overexpression of Mzb1 protects against AMI-induced myocardial injury and cardiac dysfunction, attenuates cardiac inflammation and ER stress, and preserves mitochondria function. This suggests that Mzb1 is an important cardioprotective molecule and that loss of Mzb1 may be an important mechanism of ischemia-induced myocardial injury (Zhang et al., 2021).

Puerarin is a natural isoflavone compound extracted from the root of Pueraria, which has a long history of being used to treat cardiovascular diseases and diabetes in China (Wang S. et al., 2020; Bai et al., 2021). Puerarin has important pharmacological properties, such as inhibiting oxidative stress, inflammation, and cell apoptosis, and exerts cardiovascular beneficial effects (Hou et al., 2021; Lv et al., 2022). We have recently shown that long-term treatment with puerarin improves endothelial function and vascular insulin phosphatidylinositol-3-kinase (PI3K) signaling and insulin-mediated vasorelaxation in several hypertensive animal models (Li et al., 2017; Tan et al., 2017). In MI rats, puerarin protects against myocardial ischemia/reperfusion injury and reduces cardiac inflammation, the infarction zone, and the incidence of mortality (Wang Z. K. et al., 2020). However, the mechanisms by which puerarin protects against ischemia myocardial injury are not fully understood. In our preliminary study, we found that pretreatment with puerarin prevented the downregulation of Mzb1 expression in AMI mice. In the present study, we investigated a novel pathway, Kruppel-like factor 4 (KLF4)/Mzb1, in the protection of puerarin against ER stress and myocardial injury induced by myocardial ischemia.

## 2 Materials and methods

### 2.1 Animals

C57BL/6 mice (male, approximately 25 g) were purchased from Liaoning Changsheng Biotechnology Co., LTD. (Liaoning, China). All animal studies complied with the Care and Use of Laboratory Animals of Shenyang Medical College, and all animal experimental procedures were approved by the Institute Animal Use and Care Committee of Shenyang Medical College (No. SYYXY2021110101). The mice lived in the animal facility, with 3–4 mice per cage and 12 h/12 h of light/dark cycle. After adapting to the new environment for 2 weeks, the mice were randomly divided into four groups: a sham group with mice that underwent sham surgery, an AMI group with mice that underwent surgery ligating the left anterior descending coronary artery (LAD) to induce AMI, an AMI group with mice pretreated with a low dose of puerarin (AMI/L-Pue): the mice were administrated with a low dose of puerarin (50 mg/kg/day, dissolve in 20% Arabic gum, Shanghai, China) for 14 days, then underwent the surgery with LAD ligation for 24 h to induce AMI model; an AMI group of mice pretreated with a high dose of puerarin (AMI/H-Pue): the mice were pretreated with a high dose of puerarin (100 mg/kg/day) for 14 days before AMI was induced. Puerarin was administrated by gavage. After 24 h of AMI induction, cardiac function was evaluated by echocardiography with MYLAB™ Sigma VET (Esaote SpA, Genoa, Italy). At the end of the experiments, the mice were euthanatized by cervical dislocation, and heart tissues were snap frozen with liquid nitrogen and stored in a deep freezer (−80°C) or fixed with 4% paraformaldehyde.

### 2.2 Mouse model of AMI

Mice were anesthetized with an intraperitoneal injection of 2% 2,2,2-tribromoethanol (Avertin) (Sigma-Aldrich, Louis, MO) at a dose of 0.1 mL/10 g body weight. The skin and intercostal muscles of the left chest were incised, and the heart was squeezed out from the 3rd or 4th intercostal space. A 7-0 suture needle was used to ligate the LAD. After ligating, the heart was gently pushed back into the chest, and air was squeezed out of the tightened suture at the ligature incision. The mice were monitored with electric cardiography during and after LAD ligation. A typical ischemic elevation of the ST-T segment indicated a successful model of AMI, which was also confirmed by 2,3,5-triphenyltetrazolium chloride (TTC) staining to show myocardial ischemia and necrosis (Lu et al., 2020).

### 2.3 Echocardiography image examination

Left ventricular (LV) echocardiography images were collected and measured by MYLAB™ Sigma VET (Esaote SpA, Genoa, Italy). The probe was placed vertically on the left margin of the mouse parasternal bone, with an azimuth approximately 20–30° from the long axis of the mouse body. A long-axis view image of the left ventricle in the parasternal bone was obtained, with the sampling line avoiding the papillary muscle. Left ventricular ejection fraction (EF) and left ventricular fractional shortening (FS) were measured and calculated from M-mode recording. All measurements were taken from an average of three cardiac cycles.

### 2.4 TTC staining

After 24 h of ischemia, the hearts were rapidly removed and prepared for TTC staining. Heart tissue was frozen in a −20°C freezer for 30 min and cut into four slices perpendicular to the long axis. The slices were placed in 2% TTC staining solution for 30 min at 100 RPM and 37°C and then fixed with 4% paraformaldehyde for 30 min. The infarcted area was white, while the normal area was red. The ratio of the infarcted area to the total area was analyzed with ImageJ V1.53 (ImageJ software).

### 2.5 Hematoxylin–eosin (H&E) staining

Heart tissue was fixed in 4% paraformaldehyde and cut into 5-μm slices. H&E (Solarbio, Beijing, China) staining was used to evaluate cardiac morphologic changes. The images were acquired using a Leica DM4B microscope (Leica, Vizsla, Germany).

### 2.6 DHE staining

Paraffin-embedded heart tissues were cut into 4-μm sections and incubated with dihydroethidium (DHE) solution (3 μg/mL) for 30 min at 37°C without light. Oxidative fluorescent images were obtained by Leica DM4B fluorescence microscope (Leica, Vizsla, Germany). Average fluorescence intensity was measured and calculated by ImageJ V1.53 (ImageJ software).

### 2.7 NADPH oxidase assay

Nicotinamide adenine dinucleotide phosphate (NADPH) oxidase activity in heart homogenates was determined by a luminometer (Berthold, Black Forest, Germany) in the presence of NADPH substrate, as previously described (Zhou et al., 2005). In brief, 20 μL of cardiac homogenates were added to a 50 mmol/L phosphate assay buffer (PH 7.4) containing 1 mmol/L ethylene glycol diethylether diamine tetraacetic acid (EGTA). The reaction was started by adding lucigenin (5 μmol/L) and 100 μmol/L NADPH substrate. The result was expressed as counts/min/mg protein.

### 2.8 AC16 cardiomyocyte culture

Human AC16 cardiomyocytes were propagated using Dulbecco’s modified Eagle medium (DMEM) with 10% fetal bovine serum (FBS) in 6-well plates at 37°C, 5% CO_2_, and 95% gas. Puerarin (HPLC≥98%, Sigma-Aldrich Inco. St. Louis) was dissolved in the methanol solution, and the control group was treated with the same volume of solvent. Kenpaullone, a KLF4 small-molecule inhibitor, was dissolved in dimethyl sulfoxide (DMSO). The cardiomyocytes were pretreated with puerarin (50 μmol/L, 100 μmol/L, or 200 μmol/L) or kenpaullone (2 μmol/L) (Lei et al., 2018; Mayra et al., 2021) for 36 h, then cells were exposed to 200 μmol/L of H_2_O_2_ for 12 h, and the cells at vehicle group were treated with corresponding solvents. In some experiments, cells were transfected with siRNA (si-Mzb1 with the sequence of GGT​GTC​AGC​CAC​AAG​AGA​A) or negative control (si-NC) for 36 h and then exposed to 200 μmol/L H_2_O_2_ for 12 h. Cells were collected for further analysis.

### 2.9 MTT assay

Cell viability was measured by methylthiazolyldiphenyl-tetrazolium bromide (MTT) assay. Human AC16 cardiomyocytes were seeded in a 96-well plate. After appropriate treatment, AC16 cardiomyocytes were incubated with 5 mg/mL MTT (20 μL) for 4 h. After removing DMEM, 150 μL of DMSO was added to each well and shaken for 10 min. A Fluostar Omega (Bmg Labtech, Ofenburg, Germany) was used to detect absorbance at 490 nm, and relative absorbance values (normalized to the control) were utilized to indicate cell viability.

### 2.10 TUNEL assay

Human AC16 cardiomyocytes were fixed in 4% paraformaldehyde of PBS buffer and permeabilized with 0.1% sodium citrate containing 0.1% Triton X 100. One step transferase-mediated dUTP nick-end labeling (TUNEL) Apoptosis Assay Kit (Beyotime, Shanghai, China) was used to detect apoptotic cells according to the manufacturer’s instructions. Each sample was incubated with 50 μL TUNEL solution at 37°C for 1 h without light exposure. After washing with PBS twice, the cell nucleus was stained with 5 μg/mL of 4’,6-diamidino-2-phenylindole (DAPI) in PBS solution. The fluorescence signals were analyzed by a Leica DM4B fluorescence microscope (Leica, Vizsla, Germany). ImageJ V1.53 (ImageJ software) was used to calculate the total cell number (blue) and TUNEL-positive cell count (green).

### 2.11 Measurement of intracellular ROS

Fluorogenic dye [10 μmol/L 2′,7′-dichlorodihydrofluorescein diacetate (DCFH-DA probe, Beyotime, Shanghai, China)] was used to quantify intracellular ROS. Human AC16 cardiomyocytes were incubated with DCFH-DA for 30 min. The cells were fixed in 4% paraformaldehyde, washed three times in PBS, and then stained for 7 min with DAPI. A Leica DM4B fluorescence microscope (Leica, Vizsla, Germany) was used to examine the fluorescence signals. ImageJ V1.53 (ImageJ program) was used to calculate the number of positive fluorescence-staining cells (green) and the total cell number (blue).

### 2.12 Western blot

Total proteins were isolated from human AC16 cardiomyocytes or the left ventricular wall infarction border zone. Heart tissues were homogenized with lysis buffer, and cardiomyocytes were lysed with radioimmunoprecipitation assay (RIPA) buffer (30 μL). After centrifugation, the protein concentration in the supernatant was detected using a bicinchoninic acid assay (BCA) kit (Beyotime, Shanghai, China) according to the manufacturer’s instructions. SDS-PAGE gels were used to separate protein samples (50–80 g), which were then transferred to polyvinylidene fluoride (PVDF) membranes. The membranes were incubated with 5% bovine serum albumin/tris buffered saline (BSA/TBS) blocking solution for 1 h at room temperature, then incubated with various primary antibodies at 4°C overnight, including anti-Mzb1 (ab181205, Abcam, Cambridge, MA, United States), anti-dynamin-related protein 1 (Drp1, ab56788, Abcam, Cambridge, MA, United States), anti-caspase3 (sc-7148, Santa Cruz Biotechnology, Texas, United States), anti-CCAAT/enhancer binding protein homologous protein (CHOP, 15204-1-AP, Proteintech, Wuhan, China), anti-glucose-regulated protein 78 (GRP78, 11587-1-AP, Proteintech, Wuhan, China), anti-phospho-inositol-requiring enzyme 1 (p-IRE1, AF7150, Affinity, Jiangsu, China), anti-IRE1 (DF7709, Affinity, Jiangsu, China), anti-p-Drp1ser616 (DF2972, Affinity, Jiangsu, China), and anti-KLF4 (WL02532, Wanleibio, Shenyang, China) antibodies. After washing with TBS buffer three times, the membranes were incubated with the peroxidase-conjugated secondary antibodies at room temperature for 1 h. The protein signal was visualized by Omega LUM C (Aplegen, PA, United States) using the enhanced chemiluminescence (ECL) reagent and quantified by ImageJ V1.53 (ImageJ software). The data was normalized to the internal reference protein GAPDH and expressed as fold increase vs. control group.

### 2.13 Determination of ATP content

ATP content was determined by luminescence assay according to the manufacturer’s instructions (Beyotime, Shanghai, China). Briefly, AC16 cardiomyocytes were incubated with 100 μL ATP lysis buffer. After centrifugation at 12,000 g at 4°C for 5 min, the cells were incubated with 100 μL ATP test solution (containing firefly luciferase) for 5 min, then 20 μL of lysate cells was added into a test tube and mixed quickly. The relative light units (RLU) value was measured using a luminometer (Berthold, Black Forest, Germany), and the protein concentration was determined using the BCA assay. Data were expressed as ATP nmol/mg protein.

### 2.14 qPCR

Total RNA was isolated using a FastPure^®^ Cell/Tissue Total RNA Isolation Kit V2 (Vazyme, Nanjing, China) according to the manufacturer’s instructions. Reverse transcription using HiScript^®^ III RT SuperMix for qPCR regent (Vazyme, Nanjing, China) was used to create complementary DNA (cDNA) from RNA. A 9600 qPCR system (Bioer, Hangzhou, China) was used to perform qPCR using gene-specific primers in the presence of SYBR qPCR Master Mix (Vazyme, Nanjing, China). The results of qPCR were expressed as 2^−ΔΔCT^, and the relative mRNA expressions were normalized by the control group. The primer sequences for the target genes are presented in Table 1.

**TABLE 1:** Primer sequences list.

Primer name	Forward	Reverse
Has-Mzb1	5′-ACT​GGC​AGG​ACT​ACG​GAG​TTC​G-3′	5′-CAC​GCT​GAT​GCT​TGG​CTC​TGG-3′
Mmu-Mzb1	5′-GCG​AAA​GCA​GAG​GCT​AAA​TC-3′	5′-GGA​CCC​CCA​GAA​ATC​ATC​A-3′
Mmu-SDHA	5′-GAC​AGG​GGA​ATG​GTT​TGG​A-3′	5′-CAG​CCC​GCA​CTT​TGT​AAT​C-3′
Mmu-NDUFB2	5′-GTA​CAG​GGA​GTT​TCC​CCA​GC-3′	5′-CGA​GTC​ATG​CCA​AAA​TCG​CC-3′
Mmu-Drp1	5′-ATT​CTT​CGG​TTC​ATC​AGT​AAT​CCC​A-3′	5′-AAT​AAC​CCT​TCC​CAT​CAA​TAC​ATC​C-3′
Mmu-Fis1	5′-TGA​ATA​CGC​CTG​GTG​CCT​GGT​T-3′	5′-TCC​CGC​TGC​TCC​TCT​TTG​CTA​C-3′
Mmu-Opa1	5′-ATA​CTG​GGA​TCT​GCT​GTT​GG-3′	5′-AAG​TCA​GGC​ACA​ATC​CAC​TT-3′
Mmu-Mfn1	5′-AGA​TAA​TGC​AGC​CCA​GGA​AGA​G-3′	5′-GCA​CGA​GTA​GTC​CAA​GTC​AGT-3′
Mmu-Mfn2	5′-GAG​TGT​CAA​GAC​CGT​GAA​CCA-3′	5′-CAT​CCA​GGC​AAA​ACT​TAT​CAA​TCC​A-3′
Mmu-HSP90AA1	5′-ACC​TTT​GCC​TTT​CAG​GCA​GAA-3′	5′-CCG​ATG​AAT​TGG​AGA​TGA​GCT​C-3′
Mmu-Hspa1b	5′-GCT​TGG​GCA​CCG​ATT​ACT​GT-3′	5′-CAG​TGC​TGC​TCC​CAA​CAT​TAC-3′
Mmu-Tomm20	5′-GCC​CTC​TTC​ATC​GGG​TAC​TG-3′	5′-ACC​AAG​CTG​TAT​CTC​TTC​AAG​GA-3′
Mmu-Timm23	5′-GGA​TTG​AAG​GAA​ACC​CAG-3′	5′-CTA​GAG​TAT​TAG​CCC​AAA​GTG-3′

### 2.15 Bioinformatics analysis

The transcription factors and binding sites of Mzb1 were predicted by the UCSC database (http://genome.ucsc.edu/) and the JASPAR website (http://jaspar.genereg.net/).

### 2.16 Statistical analysis

All values are expressed as mean ± SEM. For experiments with two groups, *p*-values were calculated using an unpaired, two-tailed Student’s t-test. One-way ANOVA was applied for multiple-group comparisons. GraphPad Prism V 10.0 (GraphPad Software) was used to analyze the data. The resulting *p*-values were corrected with the Benjamini, Krieger, and Yekutieli method to control the false discovery rate., and experiments were repeated a minimum of three times.

## 3 Results

### 3.1 Pretreatment with puerarin protects against AMI-induced cardiac injury and cardiac dysfunction

**FIGURE 1:**
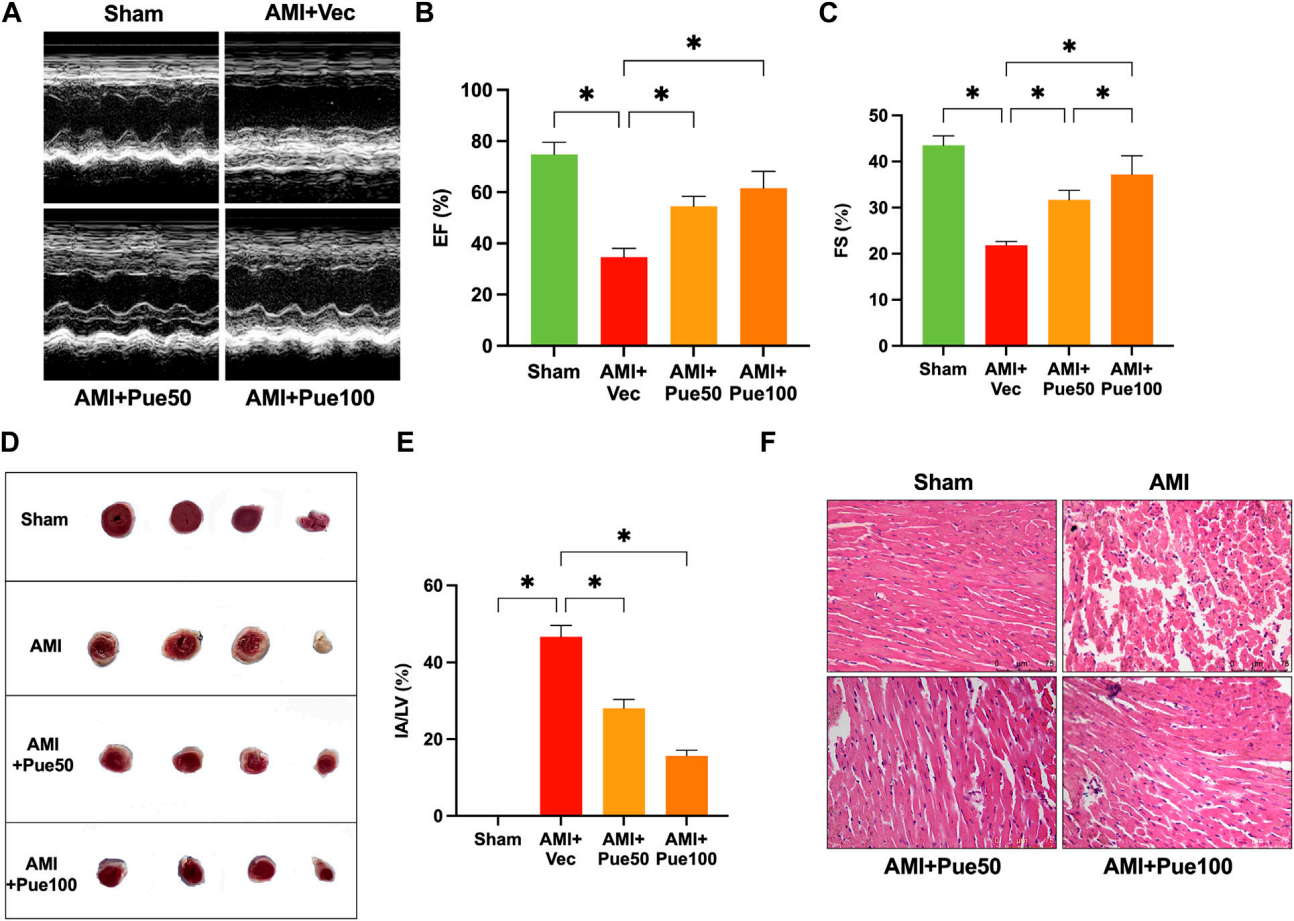
Puerarin attenuates cardiac structural injury and dysfunction in acute myocardial infarction (AMI) mice. **(A)** The representative images of echocardiography; Left ventricular EF **(B)** and FS **(C)** assessed by M-mode echocardiography (n = 4–6); **(D)** Representative image of cardiac infarct volume assessed by 2,3,5-triphenyltetrazolium chloride (TTC)-staining; **(E)** The percentage of the infarct area (IA) over the left ventricular (LV) area (n = 3); **(F)** H&E staining shows cardiomyocyte disarrangement and structural disorder (magnification ×200, scale bars = 75 μm, n = 3); Sham, sham-operated group; AMI + Vec, acute myocardial infarction + vehicle group. AMI + Pue50, acute myocardial infarction + puerarin 50 mg/kg treated group. AMI + Pue100, acute myocardial infarction + puerarin 100 mg/kg treated group. *p < 0.05.

C57BL/6 mice were treated with different doses (50 and 100 mg/kg/day) of puerarin for 14 days, followed by the induction of the AMI model by ligating LAD. We used the M-model of echocardiography to assess the cardiac function of mice, and the parameters used for evaluating cardiac function include left ventricular EF and FS. As shown in Figures 1A–C, EF and FS significantly decreased in AMI mice, and pretreatment with puerarin dose-dependently increased EF and FS in AMI mice. TTC staining was used to evaluate cardiac infarct areas. LAD ligation caused large areas of myocardial ischemia and infarction (white) in the left ventricle. The pretreatment with puerarin dose-dependently reduced the areas of myocardial ischemia and infarction (Figures 1D, E). H&E staining showed that the mice hearts in the AMI group had a cardiomyocyte disarrangement and structural disorder, which was partly improved by puerarin pretreatment (Figure 1F).

### 3.2 Puerarin attenuates AMI-induced cardiac ROS production, ER stress, and apoptosis

**FIGURE 2:**
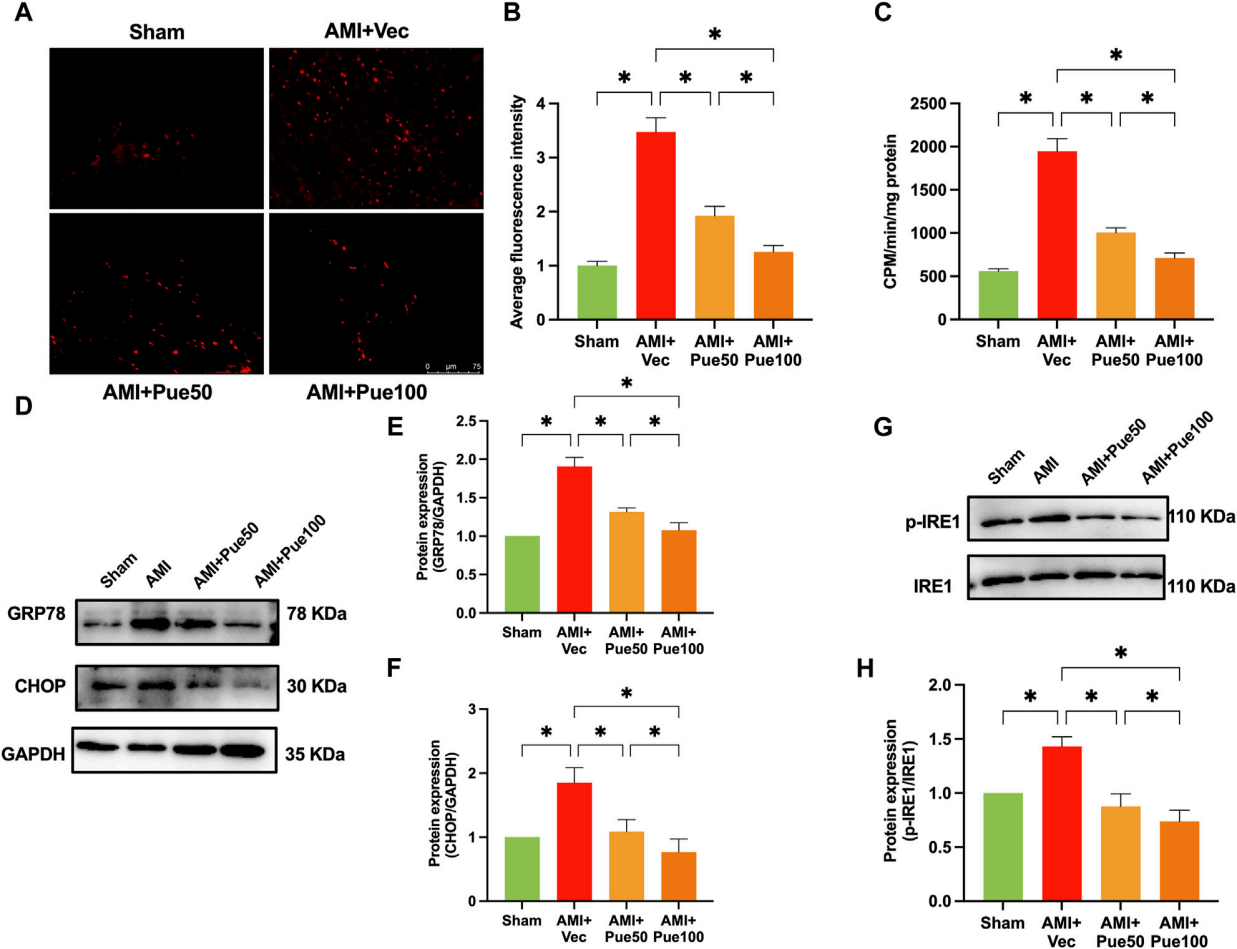
Puerarin attenuates cardiac oxidative stress and ER stress in AMI mice. **(A)** The representative images of cardiac oxidative fluorescence intensity assessed by hydroethidine (DHE) staining (magnification ×200, scale bars = 75 μm); **(B)** The quantitation of relative average DHE fluorescence intensity (n = 6); **(C)** NADPH oxidative activity (n = 6); **(D)** The representative WB bands of GRP78 and CHOP; The quantitation protein level of GRP78 **(E)** and CHOP **(F)**, (n = 6); The representative WB images of p-IRE1 and IRE1 **(G)**; The ratio of p-IRE1/IRE1 **(H)**, (n = 6) *p < 0.05.

Cardiac ROS production was determined by measuring the DHE oxidative fluorescence intensity, as shown in Figures 2A, B. The average oxidative fluorescence intensity significantly increased in the hearts of AMI mice and was reduced in AMI mice pretreated with puerarin in a dose-dependent manner. The NADPH oxidase activity significantly increased in the AMI mice and was reduced in the puerarin-pretreated AMI mice (Figure 2C). GRP78 is an important chaperone protein of ER, and CHOP and IRE1 are important signaling molecules of ER; ER stress increases the expression of GRP78 and CHOP and induces IRE1 phosphorylation. As shown in Figures 2D–H, the protein expressions of GRP78, CHOP, and the ratio of p-IRE1/IRE1 significantly increased in AMI mice. Puerarin reduced the expressions of the ER stress proteins in AMI mice, suggesting that pretreatment with puerarin can attenuate AMI-induced oxidative stress and ER stress.

**FIGURE 3:**
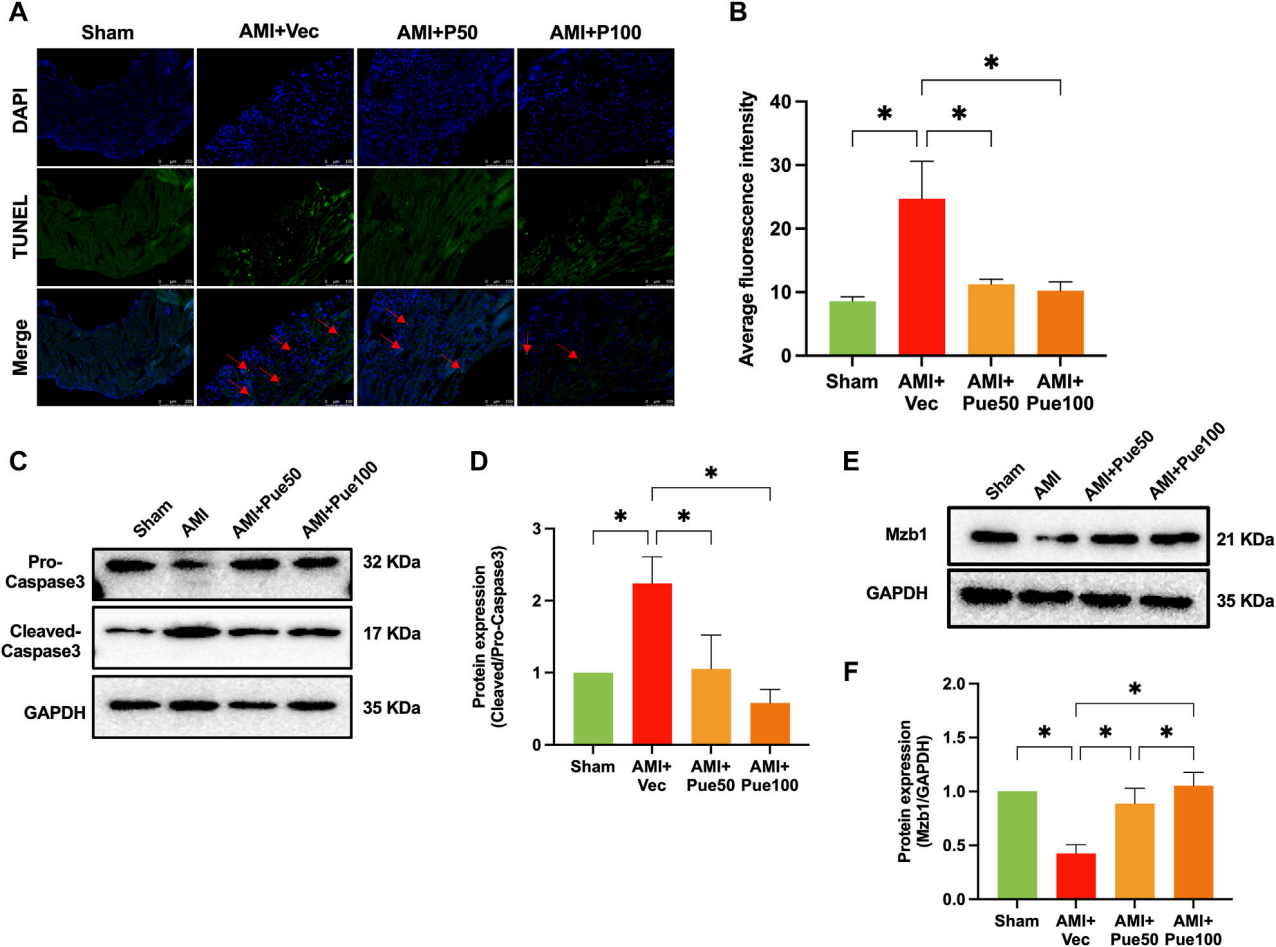
Puerarin increases Mzb1 expression and inhibits cardiomyocyte apoptosis in AMI mice. **(A)** The representative images of TUNEL staining for the assessment of cardiomyocyte apoptosis (magnification ×200, scale bars = 100 μm); **(B)** The quantitative analysis of the average fluorescence intensity of cardiomyocyte apoptosis (n = 6). **(C)** The representative WB bands of cleaved-caspase 3; **(D)** The quantitation protein level of cleaved-caspase 3 (n = 6); **(E)** The representative WB bands of Mzb1; **(F)** the quantitation protein level of Mzb1 expression (n = 6). *p < 0.05.

We assessed cardiac apoptosis using TUNEL staining and cleaved-caspase 3 expression. The average fluorescence intensity of TUNEL staining (Figures 3A, B) and the protein expression of cleaved-caspase 3 (Figures 3C, D) were significantly increased in AMI mice. Puerarin dose-dependently reduced the average fluorescence intensity and cleaved-caspase 3 expression, suggesting that pretreatment with puerarin prevented AMI-induced cardiomyocyte apoptosis. Recently, we have shown that AMI reduces the expression of the cardioprotective molecule Mzb1. Consistent with the findings in our previous study (Zhang et al., 2021), the protein expression of Mzb1 significantly decreased in AMI mice and was upregulated by puerarin (Figures 3E, F).

### 3.3 Puerarin blocks AMI-induced changes in mitochondrial biogenesis-related genes

**FIGURE 4:**
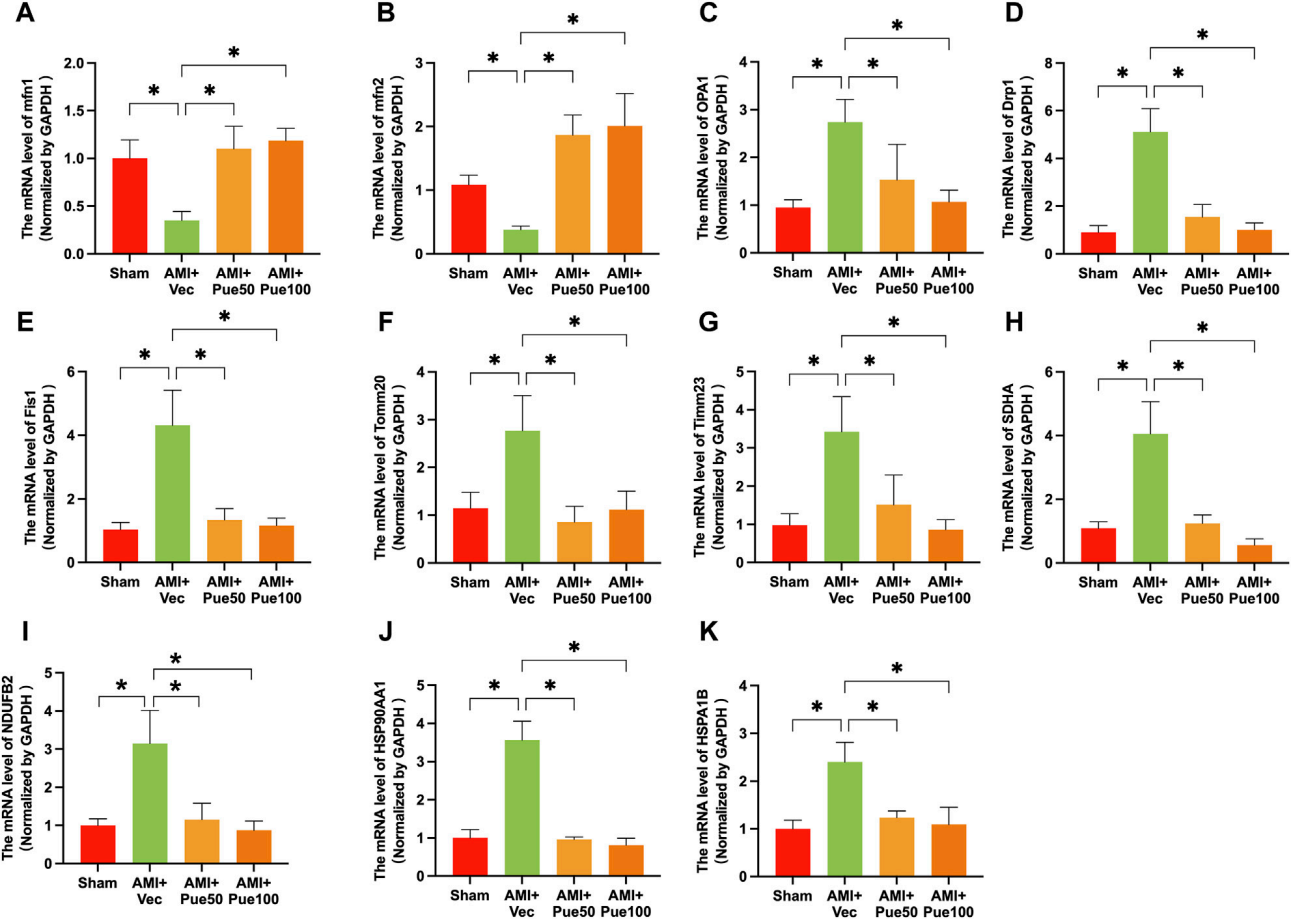
Puerarin reverses the mRNA expressions of mitochondrial biogenesis-related genes in AMI mice. The mRNA expression of mitochondrial fission and fusion proteins mitofusin 1 [mfn1, **(A)**, n = 5] and mitofusin 2 [mfn2, **(B)**, n = 6–8], optic atrophy 1 [OPA1, **(C)**, n = 6], dynamin-related protein 1 [Drp1, **(D)**, n = 6], mitochondrial fission 1 protein [Fis1, **(E)**, n = 6]; the mRNA expression of mitochondrial membrane proteins Tomm20 [mitochondrial import receptor subunit Tom20 homolog, **(F)**, n = 6] and Timm23 [translocase of inner mitochondrial membrane 23, **(G)**, n = 6]. **(H,I)** The mRNA level of mitochondrial electron transport chain proteins SDHA [succinate dehydrogenase complex flavoprotein subunit A, **(H)** n = 6–8] and NDUF2 [NADH: ubiquinone oxidoreductase subunit B2, **(I)**, n = 6–8]; The molecular chaperones associated with mitochondrial function HSP90AA1 [heat shock protein 90 alpha family class A member 1 **(J)**, n = 5–6] and HSPA1B [heat shock protein family **(A)** member 1 **(B,K)**, n = 6]. Vec, vehicle group; *p < 0.05.

AMI often causes abnormal mitochondrial biogenesis. To investigate whether puerarin protects against AMI-induced mitochondrial biogenesis dysfunction, we determined the mRNA expression of a series of mitochondrial biogenesis genes. AMI significantly decreased the mRNA expressions of the mitochondrial fusion proteins mfn1 and mfn2 (Figures 4A, B) and increased the mRNA levels of the mitochondrial fusion protein OPA1 (Figure 4C) and the mitochondrial fission proteins Drp1 and Fis1 (Figures 4D, E). AMI also increased the mRNA expressions of the mitochondrial membrane proteins Tomm20 and Timm23 (Figures 4F, G), the mitochondrial electron transport chain proteins SDHA and NDUFB2 (Figures 4H, I), and the molecular chaperones associated with mitochondrial function HSP90AA1 and HSPA1B (Figures 4J, K). Pretreatment with puerarin could reverse all alterations in these gene expressions, suggesting that puerarin can protect AMI mice from mitochondrial biogenesis dysfunction.

### 3.4 siRNA Mzb1 reverses puerarin improvement of cell viability and the inhibition of apoptosis in H_2_O_2_-induced AC16 cardiomyocytes

**FIGURE 5:**
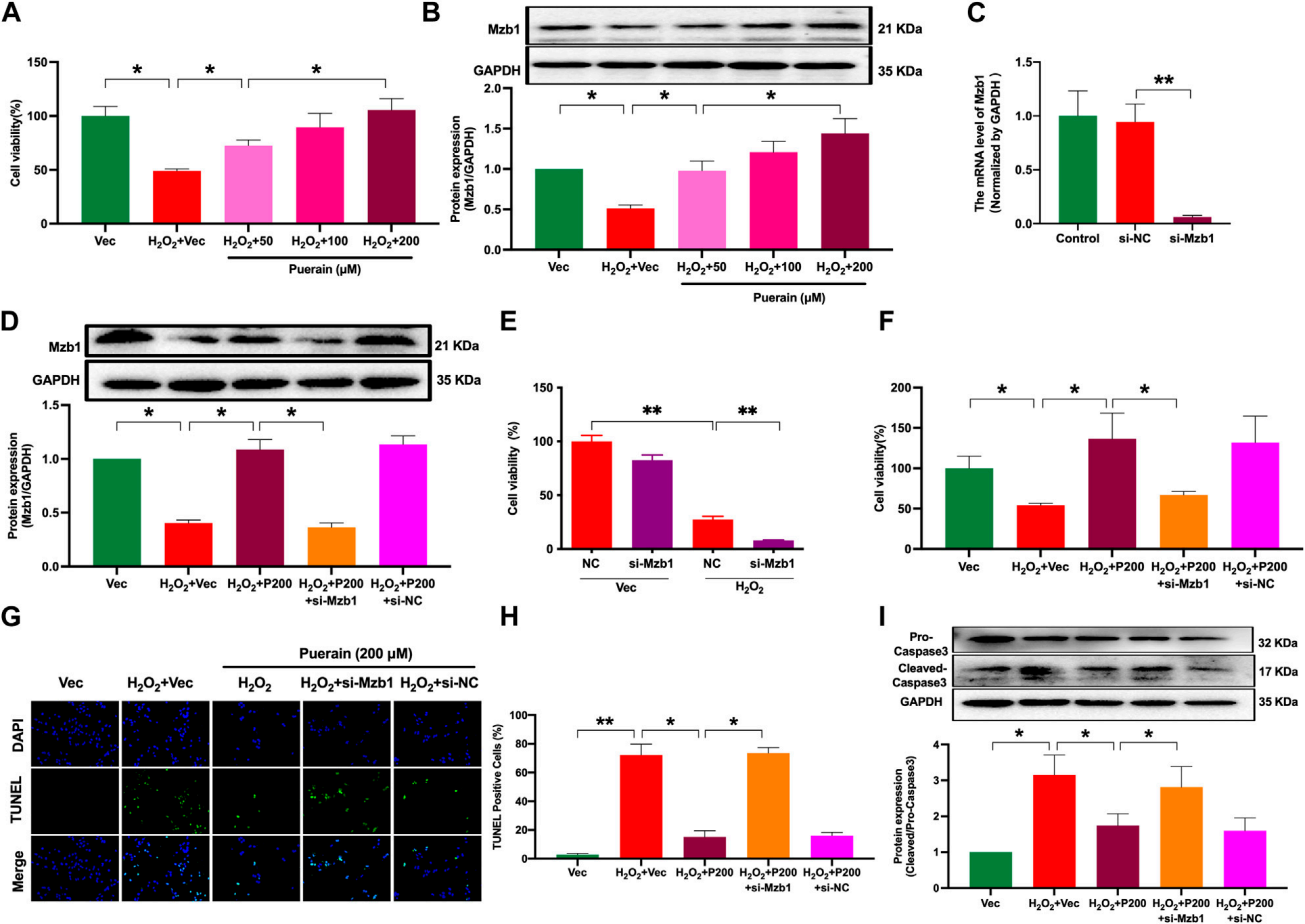
siRNA Mzb1 attenuates the effects of puerarin on improving cell viability and inhibiting apoptosis in H_2_O_2_-treated AC16 cardiomyocytes. **(A)** Puerarin dose-dependently increased cell viability in H_2_O_2_-treated cardiomyocytes (n = 10); **(B)** The protein expression of Mzb1 (n = 6); **(C)** siRNA Mzb1 reduced mRNA expression of Mzb1 (n = 6); **(D)** siRNA Mzb1 suppressed puerarin upregulation of Mzb1 protein expression in H_2_O_2_-treated cardiomyocytes (n = 6); **(E)** The effect of siRNA Mzb1 on cell viability in H_2_O_2_-treated AC16 cardiomyocytes (n = 10); **(F)** siRNA Mzb1 reversed puerarin increase of cell viability in H_2_O_2_-treated cardiomyocytes (n = 10); **(G,H)** siRNA Mzb1 blocked puerarin inhibition of H_2_O_2_-induced cardiomyocyte apoptosis; the representative image shows TUNEL staining positive cells [magnification ×200, scale bars = 250 μm, **(G)**]; the quantification of TUNEL staining positive cells **(H)**, (n = 6). **(I)** The protein expression of cleaved-caspase 3 (n = 6). *p < 0.05; **p < 0.01.

H_2_O_2_-induced cardiomyocyte injury is a common cell model used to mimic ischemia-induced cardiomyocyte injury *in vitro* (Madiha Zahra et al., 2019). As shown in Figure 5A, cardiomyocytes exposure to H_2_O_2_ exhibited a significant reduction in cell viability; pretreatment with puerarin dose-dependently increased cell viability in H_2_O_2_-treated AC16 cardiomyocytes. H_2_O_2_ treatment increased the number of positive TUNEL staining cells and the protein expression of cleaved-caspase 3, and both were prevented by puerarin treatment (Supplementary Figures S1A–C). These results suggest that puerarin can inhibit cardiomyocyte apoptosis and increase cell survival. We have previously shown that the overexpression of Mzb1 protected against AMI-induced mitochondrial dysfunction and cardiomyocyte injury (Zhang et al., 2021). Consistent with our findings in AMI mice *in vivo,* puerarin pretreatment upregulated the protein expression of Mzb1 in AC16 cardiomyocytes (Figure 5B). We silenced the Mzb1 gene using siRNA Mzb1 to investigate the role of Mzb1 in puerarin protection of the ischemic myocardium. The siRNA Mzb1 gene reduced mRNA expression of Mzb1 in normal AC16 cells by more than 90% (Figure 5C), and it significantly reduced puerarin upregulation of the Mzb1 protein expression in H_2_O_2_-treated cardiomyocytes (Figure 5D), confirming the efficiency of the knockout Mzb1 gene. siRNA Mzb1 did not significantly affect cell viability in normal AC16 cells but further reduced cell viability in H_2_O_2_-treated AC16 cells (Figure 5E) and blocked the effects of puerarin on increasing cell viability (Figure 5F). siRNA Mzb1 blocked puerarin downregulation of the positive number of TUNEL staining cells and cleaved-caspase 3 expression in H_2_O_2_-treated AC16 cardiomyocytes (Figures 5G–I), suggesting an important role of Mzb1 in puerarin inhibition of H_2_O_2_-induced cardiomyocyte apoptosis.

### 3.5 siRNA Mzb1 attenuates puerarin inhibition of H_2_O_2_-induced oxidative stress and ER stress in AC16 cardiomyocytes

**FIGURE 6:**
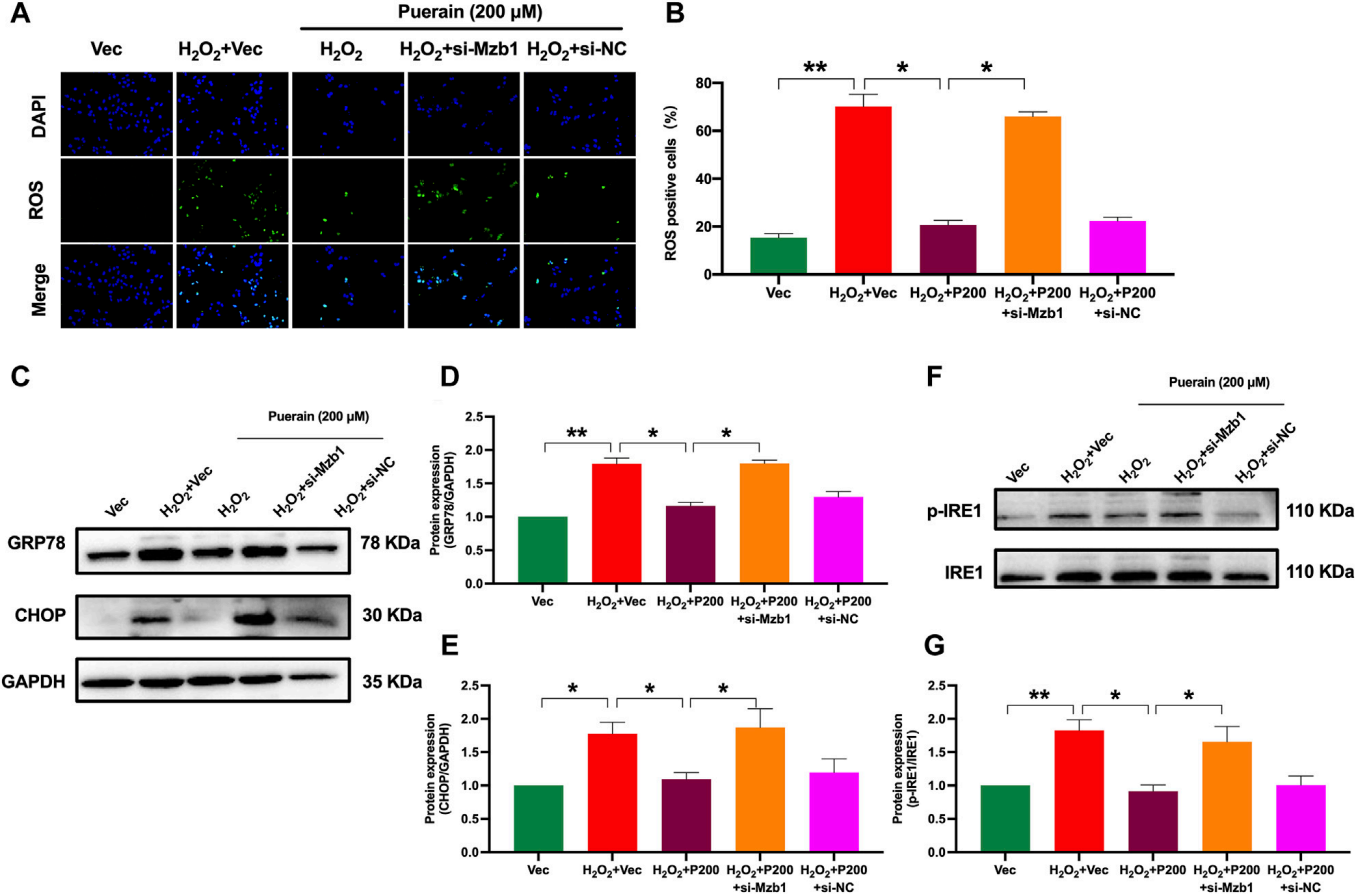
siRNA Mzb1 attenuates puerarin inhibition of H_2_O_2_-induced oxidative stress and ER stress in AC16 cardiomyocytes. **(A,B)** siRNA Mzb1 blocked puerarin inhibition of H_2_O_2_-induced ROS production (magnification ×200, scale bars = 250 μm, n = 6); **(C–G)** siRNA Mzb1 attenuated puerarin inhibition of the protein expression of ER stress molecules GRP78, CHOP, and p-IRE1 in H_2_O_2_-treated AC16 cardiomyocytes (n = 6). *p < 0.05; **p < 0.01.

AC16 cardiomyocytes exposure to H_2_O_2_ significantly increased ROS production, as demonstrated by an increased number of positive DCFH-DA fluorescence-staining cells. Puerarin significantly reduced the positive DCFH-DA staining cell number in a dose-dependent manner (Supplementary Figures S2A, B). siRNA Mzb1 attenuated the inhibitory effects of puerarin on H_2_O_2_-induced ROS production (Figures 6A, B). Moreover, puerarin significantly suppressed H_2_O_2_-induced protein expression of ER stress molecules GRP78, CHOP, and the ratio of p-IRE1/IRE1 (Figures 6C–G), which were also reversed by siRNA Mzb1. These results suggest that puerarin inhibits H_2_O_2_-induced ROS and ER stress by upregulating Mzb1.

### 3.6 siRNA Mzb1 reverses puerarin improvement of ATP production and downregulation of DRP1 expression in H_2_O_2_-treated AC16 cardiomyocytes

**FIGURE 7:**
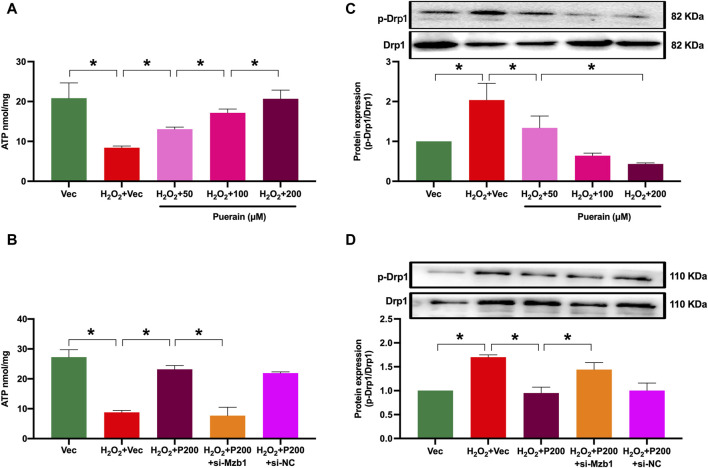
siRNA Mzb1 blocks the puerarin increase of ATP content and inhibits Drp-1 phosphorylation in H_2_O_2_-treated AC16 cardiomyocytes. **(A)** Puerarin dose-dependently increased ATP content in H_2_O_2_-treated cardiomyocytes (n = 6); **(B)** siRNA Mzb1 attenuated puerarin’s increase of ATP content in H_2_O_2_-treated cardiomyocytes (n = 3); **(C)** puerarin inhibited Drp1 phosphorylation in H_2_O_2_-treated cardiomyocytes (n = 3); **(D)** siRNA Mzb1 effectively reduced puerarin upregulation of p-Drp1 protein expression in H_2_O_2_-treated cardiomyocytes (n = 4). *p < 0.05.

Myocardial infarction is often associated with mitochondrial dysfunction and the impairment of mitochondria ATP production. Drp1 is a main pro-fission protein that regulates mitochondrial dynamics. Myocardial infarction or ischemia upregulates myocardial Drp1 expression to induce mitochondrial dysfunction. As shown in Figure 7A, ATP content was significantly reduced in H_2_O_2_-treated AC16 cardiomyocytes. Puerarin prevented an H_2_O_2_-induced decrease in ATP content. Moreover, siRNA Mzb1 diminished puerarin restoration of ATP production in H_2_O_2-_treated cells (Figure 7B). Exposure to H_2_O_2_ increased Drp1 phosphorylation (p-Drp1), which was prevented by puerarin treatment (Figure 7C). siRNA Mzb1 reversed puerarin downregulation of p-Drp1 in H_2_O_2_-treated cells (Figure 7D). These results suggest that puerarin protects against H_2_O_2_-induced mitochondrial dysfunction by upregulating Mzb1.

### 3.7 Puerarin increases Mzb1 expression through regulating transcription factor KLF4

**FIGURE 8:**
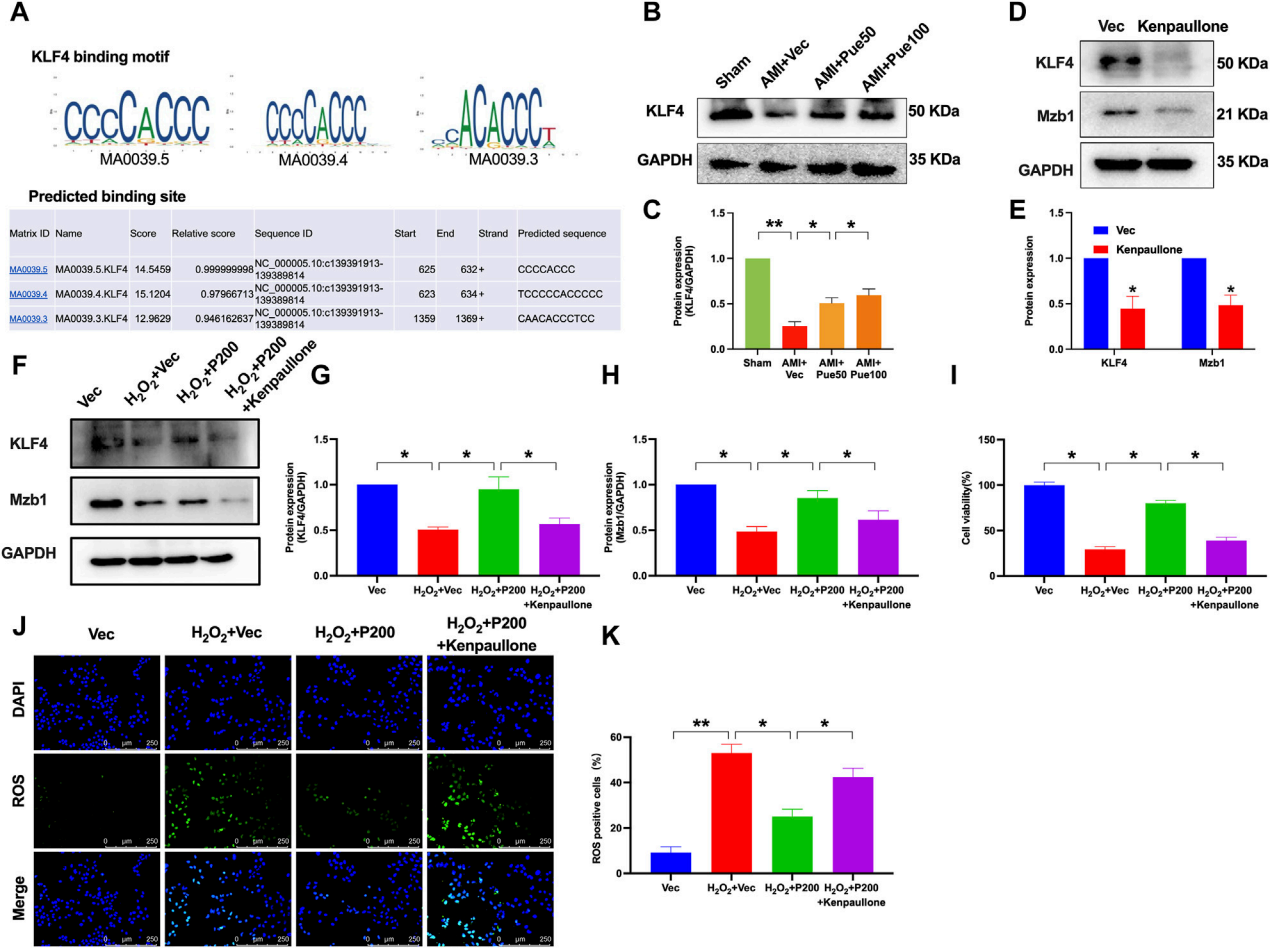
KLF4 mediates the effects of puerarin on Mzb1 expression, cell viability, and ROS production in H_2_O_2_-treated AC16 cardiomyocytes. **(A)** UCSC and JASPAR were used to predict the binding site of KLF4 and Mzb1. **(B,C)** Puerarin dose-dependently increased the protein expression of KLF4 in AMI mice (n = 4); kenpaullone (KLF4 inhibitor) suppressed the expression of KLF4 and Mzb1 in normal AC16 cardiomyocytes **(D,E)** and blocked puerarin upregulation of KLF4 and Mzb1 **(F–H)** expression in H_2_O_2_-treated cells; **(I)** kenpaullone blocked puerarin increase of cell viability in H_2_O_2_-treated AC16 cells; kenpaullone blocked puerarin inhibition of ROS formation; the representative images show ROS immunofluorescence staining [**(J)**, magnification ×200, scale bars = 250 μm], and the quantitation of oxidative immunofluorescence intensity **(K)**, (n = 6). *p < 0.05; **p < 0.01.

We used bioinformatics analysis through the UCSC database and JASPAR website to predict potential upstream molecules by which puerarin upregulates Mzb1. We found that the transcript factor KLF4 was a potential upstream molecule of Mzb1 because there were binding sites between KLF4 and Mzb1 (Figure 8A). To further confirm whether KLF4 is an upstream molecule of puerarin upregulation of Mzb1, we investigated the effect of puerarin on KLF4 expression in the hearts of AMI mice. The protein expression of KLF4 was downregulated in the AMI group, while pretreatment with puerarin prevented the downregulation of AMI-induced KLF4 expression (Figures 8B, C). Next, we treated AC16 cells with kenpaullone (a small-molecular KLF4 inhibitor) to inhibit KLF4. Kenpaullone significantly reduced the protein expression of KLF4 and Mzb1 in normal AC16 cardiomyocytes (Figures 8D, E). Exposure to H_2_O_2_ significantly reduced the expression of KLF4 and Mzb1, while puerarin prevented H_2_O_2_-induced downregulation of KLF4 and Mzb1 expressions, but this effect of puerarin was blocked by kenpaullone (Figures 8F–H). 

We also investigated the effects of kenpaullone on cell viability and ROS formation in H_2_O_2_ and puerarin-treated cells. The inhibition of KLF4 by kenpaullone blocked the effect of puerarin on increasing cell viability and reducing ROS formation in H_2_O_2_-treated AC16 cardiomyocytes (Figures 8I–K). These results suggest that puerarin may increase Mzb1 expression by upregulating transcription factor KLF4, and the cardiac protective effects of puerarin are at least in part mediated by the activation of KLF4/Mzb1 pathway.

## 4 Discussion

**FIGURE 9:**
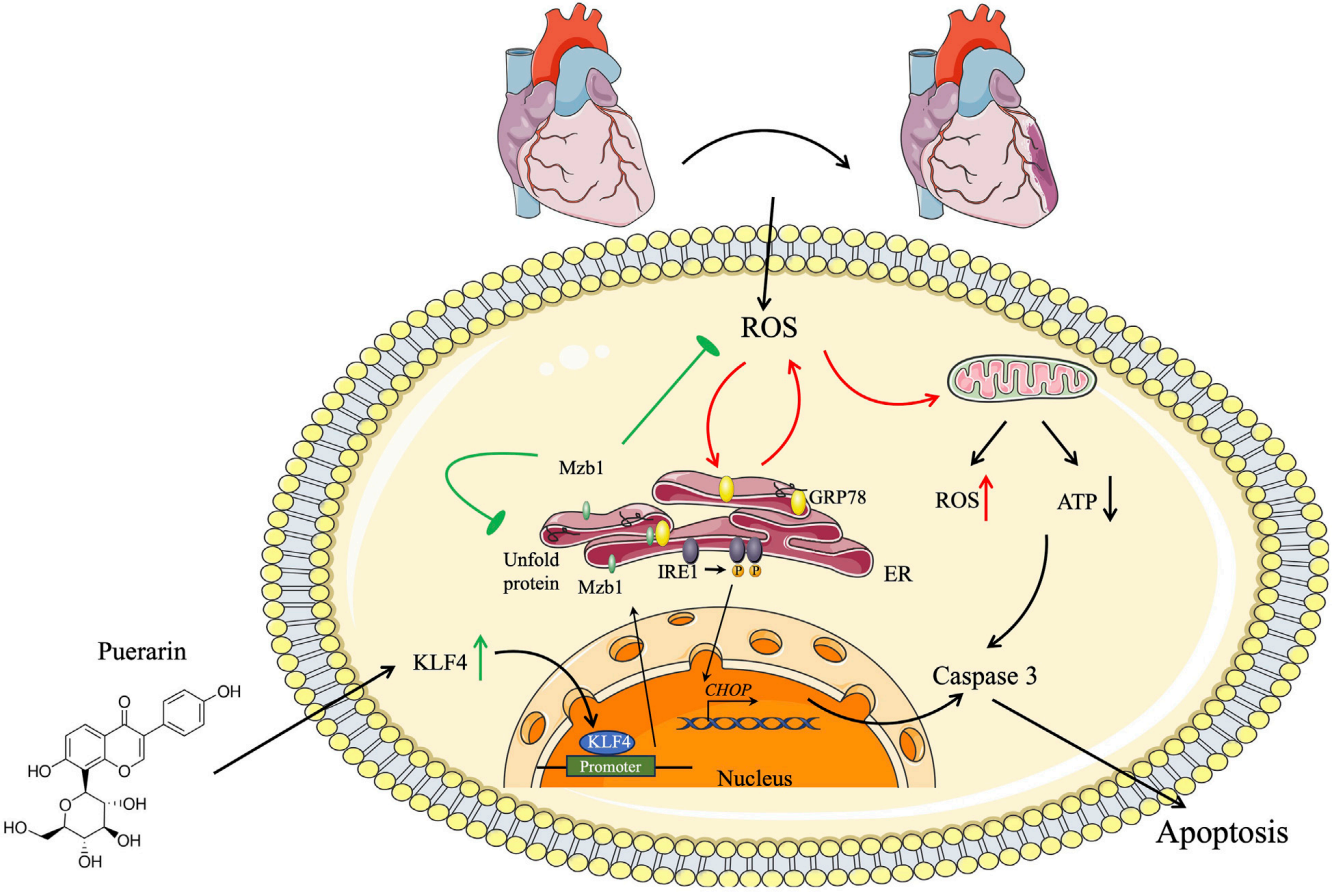
Schematic diagram. Cardioprotective effect of puerarin by inhibiting ER stress and improving mitochondrial function by inducing the KLF4/Mzb1 signal pathway.

In the present study, we demonstrate that pretreatment with puerarin protects against AMI-induced myocardial injury and alleviates ER stress and mitochondrial dysfunction in AMI mice *in vivo* and H_2_O_2_-treated cardiomyocytes *in vitro.* The cardioprotective effects of puerarin are mediated by activating the KLF4/Mzb1 pathway because either silence of Mzb1 or inhibition of KLF4 diminishes the inhibitory effects of puerarin on H_2_O_2_-induced cardiomyocyte apoptosis, ER stress, and mitochondrial dysfunction (Figure 9).

Puerarin is an isoflavone compound extracted from Chinese *Pueraria montana* var. *lobata* (Willd.) Maesen & S.M.Almeida ex Sanjappa & Predeep. It is widely prescribed for the treatment of cardiovascular diseases and diabetic complications in Eastern countries. Puerarin has a broad range of pharmacological properties, including antioxidant, anti-apoptotic effects, inflammation inhibition, and endothelial function improvement (Chang et al., 2021; Wang et al., 2022; Yu et al., 2022). Several preclinical studies have shown that puerarin can reduce the infarction size of an MI and ischemia/reperfusion myocardial injury and inflammation (Han et al., 2021). Consistent with the previous findings (Zhang et al., 2021), we show that pretreatment with puerarin protects against AMI-induced myocardial injury and reduces the infarction size associated with an attenuation of myocardial oxidative stress, ER stress, and apoptosis. The results from the *in vitro s*tudy also indicate that puerarin exerts protective effects on H_2_O_2_-induced cardiomyocyte injury by inhibiting ROS, ER stress, and apoptosis and reversing mitochondrial dysfunction. These results suggest that puerarin has important therapeutic effects on MI or ischemia-induced myocardial injury.

MI often causes widespread myocardial cell death, which leads to severe cardiac dysfunction and complications. Apoptosis is a major form of cardiomyocyte death in MI, as the myocardium is a terminally differentiated tissue. In general, it is difficult to replace damaged cardiomyocytes through regeneration (Takemura and Fujiwara, 2004). Therefore, reducing cardiomyocyte death in the ischemic zone by inhibiting apoptosis is crucial for the treatment of MI.

MI-induced cardiomyocyte apoptosis involves multiple signaling pathways, such as ROS, ER stress, and mitochondrial dysfunction. The ER is recognized as an important organelle that decides the fate of cells. Ischemia and ROS can interfere with ER function to promote the accumulation of unfolded proteins and trigger the unfolded protein response (UPR) (Ren et al., 2021). The initial UPR induced by ER stress has a protective effect on maintaining ER homeostasis. However, prolonged ER stress during MI may activate pro-apoptotic signaling molecules, such as IRE1, CHOP, and c-Jun amino-terminal Kinase (JNK), which induce myocardial apoptosis. IRE1 promotes apoptosis through activating proapoptotic signaling molecules CHOP and JNK (Wang et al., 2018). It has been shown that ER stress impairs mitochondria oxidative phosphorylation and respiratory enzyme activities, inducing mitochondrial dysfunction. Mitochondria are an important source of ROS; mitochondrial dysfunction, in turn, promotes ROS production and ER stress. Therefore, MI causes oxidative stress, ER stress, and mitochondrial dysfunction, which may form a vicious cycle to concomitantly promote myocardial cell death and injury.

In the present study, pretreatment with puerarin markedly reduced myocardial ROS and ER stress, which may inhibit pro-apoptosis signaling IRE1 and CHOP and maintain mitochondria homeostasis. Therefore, puerarin may inhibit ER stress and ROS by disrupting a vicious cycle among ROS, ER stress, and mitochondrial dysfunction, thereby protecting the myocardium from myocardial injury and cell death induced by AMI. Furthermore, we demonstrate that puerarin inhibits myocardial ER stress and apoptosis via upregulating Mzb1.

Mzb1 is an ER-located protein that functions as a cochaperone of the substrate-specific chaperone GRP94 under ER stress conditions. We have previously shown that the overexpression of Mzb1 can attenuate MI-induced ER stress, ROS formation, apoptosis, and mitochondrial dysfunction. Puerarin prevents AMI-induced downregulation of Mzb1 expression and cardiomyocyte apoptosis, while siRNA Mzb1 blocks the effect of puerarin on increasing and inhibiting ROS, ER stress, pro-apoptosis protein IRE1, and CHOP expression in H_2_O_2_-treated AC16 cells. Therefore, puerarin may protect against AMI-induced myocardial injury by upregulating Mzb1, which inhibits ER stress to interrupt the vicious cycle among ROS, ER stress, and mitochondrial dysfunction.

We performed a bioinformatic analysis through the UCSC database and JASAR websites to identify the upstream molecule by which puerarin upregulates Mzb1. We found that KLF4 is a potential upstream signaling molecule by which puerarin upregulates Mzb1. KLF4 is a zinc finger protein transcription factor that plays an important role in cell growth, apoptosis, proliferation, and differentiation (Zhihong et al., 2023). KLF4 knockout is associated with mitochondrial dysfunction, myocardial fibrosis, and even heart failure. Dongmei et al. found that the overexpression of KLF4 can alleviate myocardial infarction injury and improve cardiac function in mice (Dongmei et al., 2015), suggesting KLF4 has a cardiac protective effect (Qian et al., 2019; Xi et al., 2022; Tao et al., 2023). In the present study, puerarin prevents AMI-induced downregulation of KLF4 expression, and the inhibition of KLF4 can block puerarin upregulation of Mzb1 and reverse the protective effect of puerarin on H_2_O_2_-induced cardiomyocyte injury, ER stress, and mitochondrial dysfunction. These results are consistent with the findings that siRNA Mzb1 reverses the cardioprotective effects of puerarin in AMI mice or H_2_O_2_-treated cells. Therefore, we surmise that puerarin protection against AMI-induced myocardial injury is at least in part mediated by activating the KLF4/Mzb1 pathway.

Limitation: Although we have shown that puerarin can protect against AMI-induced myocardial injury and cardiac dysfunction by upregulating Mzb1 to inhibit ROS and ER stress, the results of this study cannot be immediately applied for treating AMI patients. The extrapolation of the results from experimental animals to the patient should be done with great caution because there are great differences in species, gene expression, and drug dosage between humans and mice, and the results of this study should be further verified by clinical trials.

In conclusion, given the long history of puerarin (Kudzu root) in the treatment of ischemic heart disease and various cardiovascular diseases, it is necessary to clarify the underlying mechanisms of the therapeutic effects of puerarin in these diseases. In the present study, we provide solid evidence showing that puerarin exerts cardiac beneficial and therapeutic effects in experimental AMI. The cardiac beneficial effects of puerarin include the inhibition of ER stress, apoptosis, and the improvement of mitochondrial dysfunction. More importantly, we found that the beneficial effects of puerarin are mediated by the regulation of KLF4, which rescues the expression of cardioprotective ER protein Mzb1. Our results suggest that puerarin may be an important adjunct medicine used for the treatment of ischemic heart disease or MI, and rescuing Mzb1 may be a new target for the prevention and treatment of cardiovascular diseases.

## Figures and Tables

**Table:** 

**AMI**	Acute myocardial infarction
**ER**	Endoplasmic reticulum
**ROS**	Reactive oxygen species
**Mzb1**	Marginal zone B and B1 cell-specific protein
**GRP94**	Glucose-regulated protein 94
**Bip**	Binding immunoglobulin protein
**PI3K**	Phosphatidylinositol-3-kinase
**KLF4**	Kruppel-like factor 4
**LAD**	Left anterior descending coronary artery
**TTC**	2,3,5-Triphenyltetrazolium chloride
**LV**	Left ventricular
**EF**	Ejection fraction
**FS**	Fractional shortening
**H&E**	Hematoxylin–eosin
**DHE**	Dihydroethidium
**NADPH**	Nicotinamide adenine dinucleotide phosphate
**EGTA**	Ethylene glycol diethylether diamine tetraacetic acid
**FBS**	Fetal bovine serum
**DMEM**	Dulbecco’s modified Eagle medium
**DMSO**	Dimethyl sulfoxide
**MTT**	Methylthiazolyldiphenyl-tetrazolium bromide
**TUNEL**	Terminal deoxynucleotidyl transferase-mediated dUTP nick end labeling
**DAPI**	4′, 6-Diamidino-2-phenylindole
**DCFH-DA**	2′,7′-Dichlorodihydrofluorescein diacetate
**RIPA**	Radio immunoprecipitation assay
**BCA**	Bicinchoninic acid assay
**PVDF**	Polyvinylidene fluoride
**BSA**	Bovine serum albumin
**TBS**	Tris buffered saline
**Drp1**	Dynamin-related protein 1
**GRP78**	Glucose-regulated protein 78
**CHOP**	C/EBP-homologous protein
**IRE1**	C/EBP-homologous protein
**mfn1**	Mitofusin 1
**mfn2**	Mitofusin 2
**OPA1**	Optic atrophy 1
**Drp1**	Dynamin-related protein 1
**Fis1**	Mitochondrial fission 1 protein
**Tomm20**	Mitochondrial import receptor subunit TOM20 homolog
**Timm23**	Translocase of inner mitochondrial membrane 23
**SDHA**	Succinate dehydrogenase complex flavoprotein subunit A
**NDUFB2**	NADH: ubiquinone oxidoreductase subunit B2
**HSP90AA1**	Heat shock protein 90 alpha A1

## Data Availability

The original contributions presented in the study are included in the article/Supplementary Material; further inquiries can be directed to the corresponding authors.
