# Supplementary figures and images for: Puerarin attenuates myocardial ischemic injury and endoplasmic reticulum stress by upregulating the Mzb1 signal pathway (part 1 of 2)
Source: Front Pharmacol. 2024 Aug 13;15:1442831. doi: 10.3389/fphar.2024.1442831 (PMC11350615; doi:10.3389/fphar.2024.1442831)

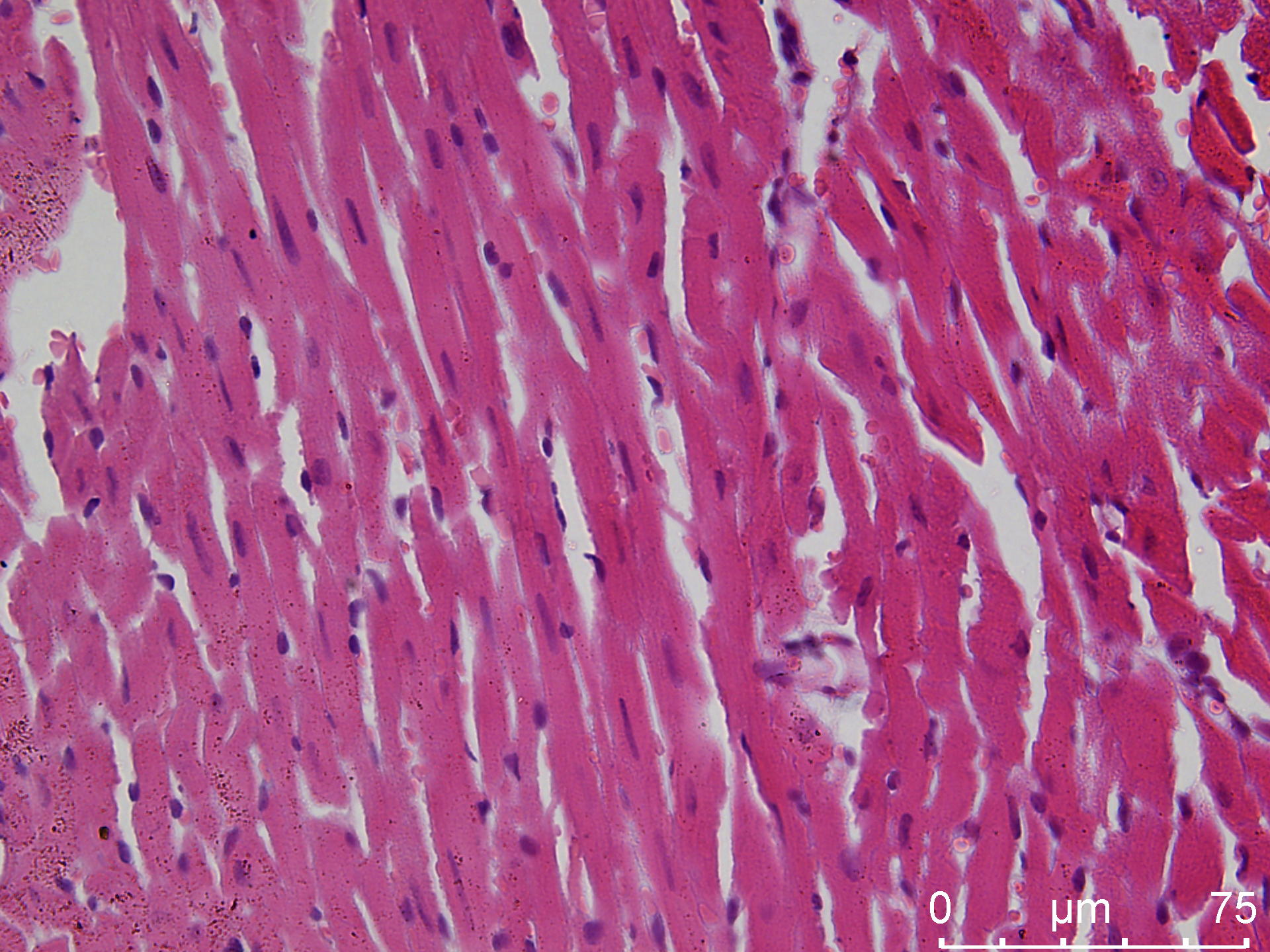

Supplement: Supplementary file 1 [file DataSheet3.zip › Figure 1F/P50-4_Image011.tif]

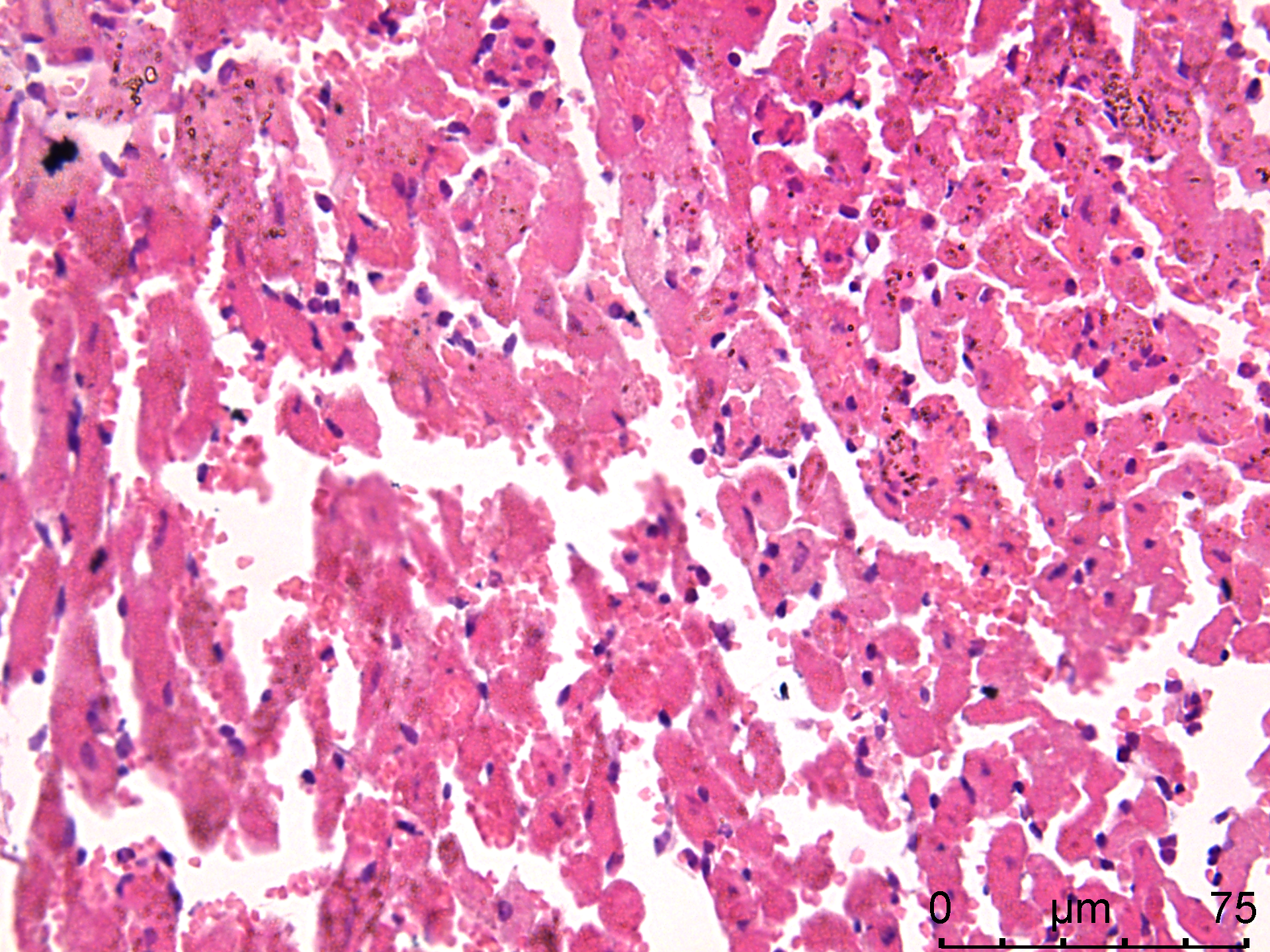

Supplement: Supplementary file 1 [file DataSheet3.zip › Figure 1F/ami-1_Image043.tif]

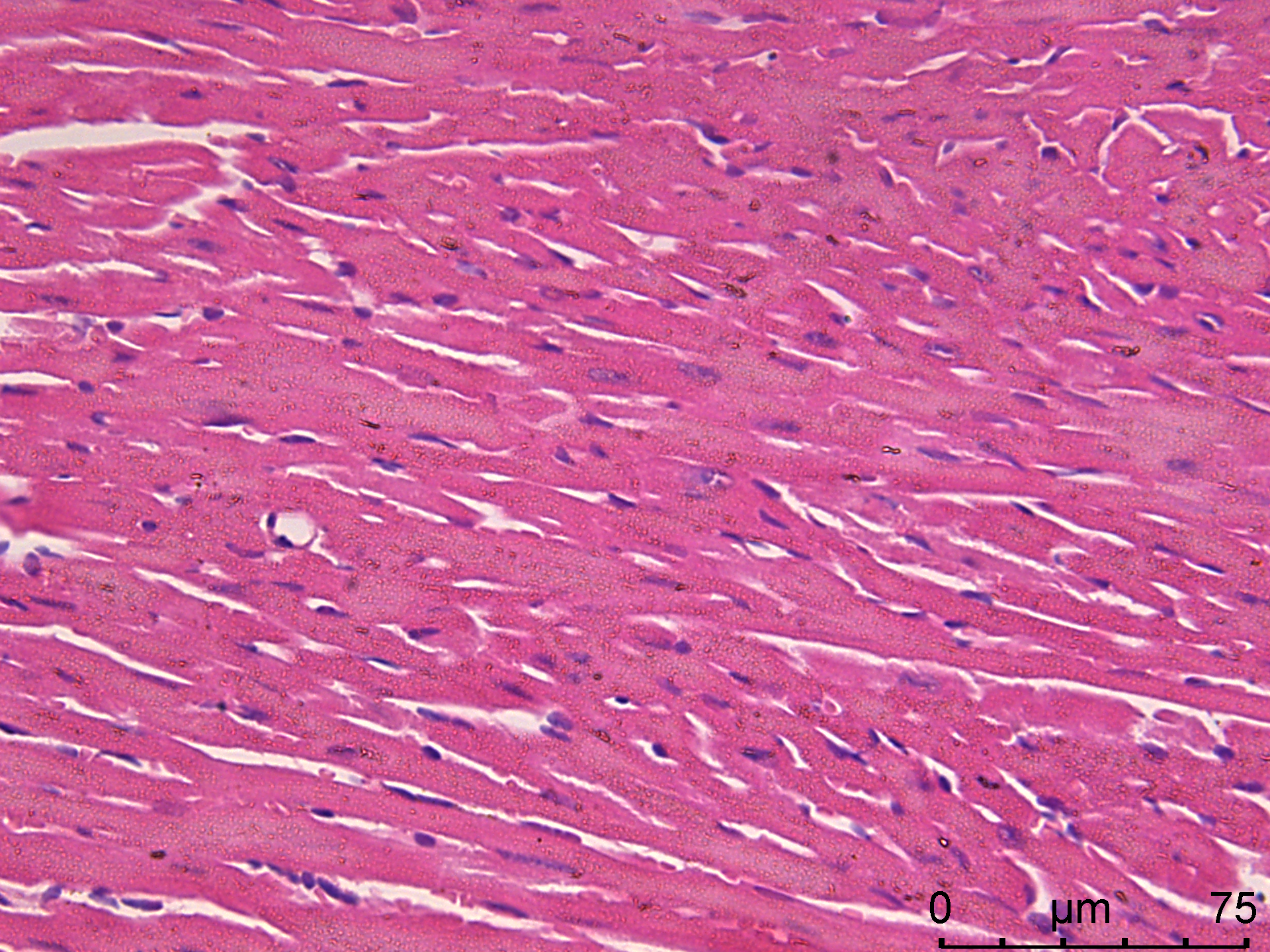

Supplement: Supplementary file 1 [file DataSheet3.zip › Figure 1F/ctl-3_Image016.tif]

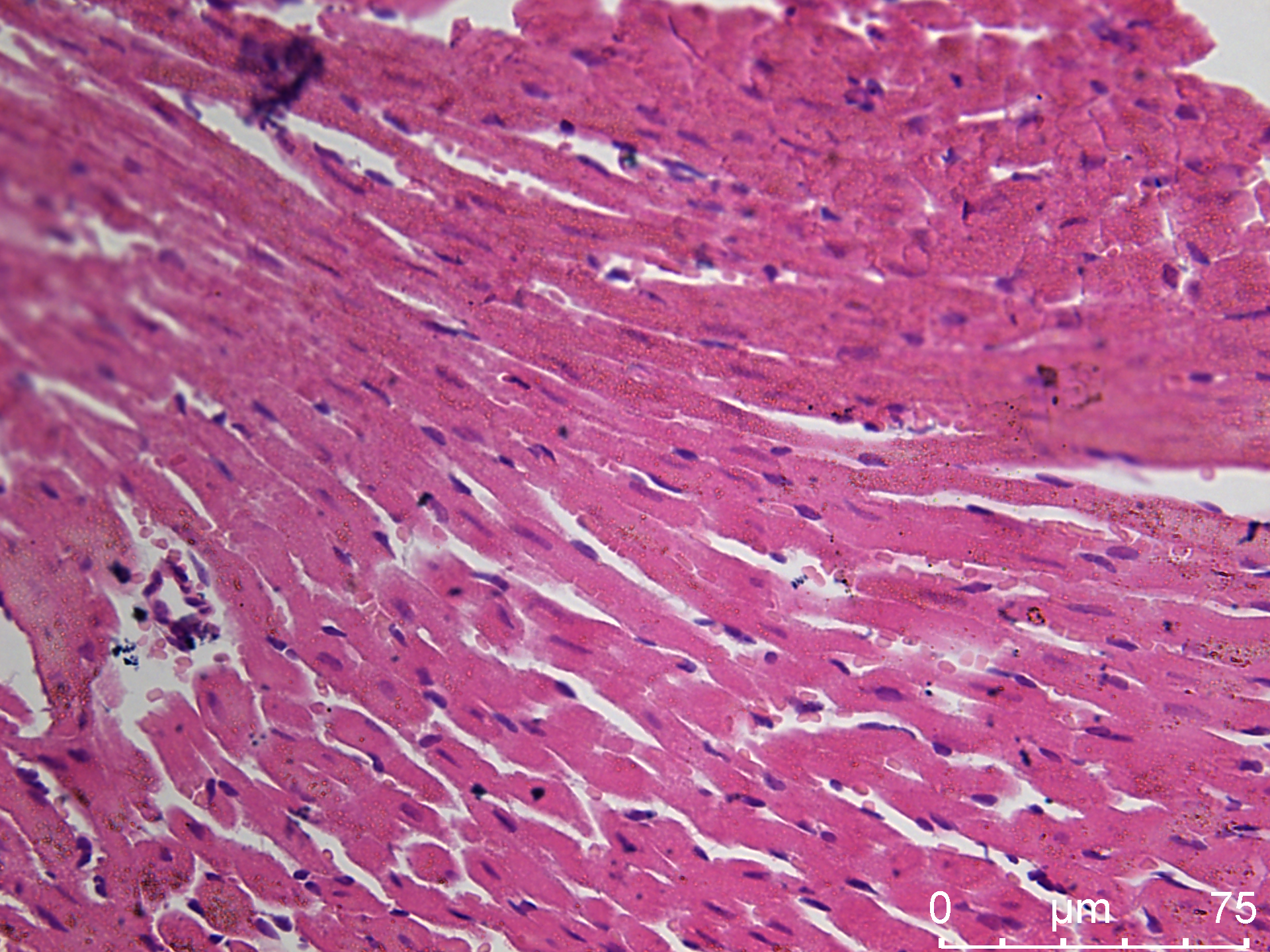

Supplement: Supplementary file 1 [file DataSheet3.zip › Figure 1F/P100-4_Image015.tif]

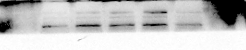

Supplement: Supplementary file 2 [file DataSheet8.zip › Figure 6/Figure 6F/p-IRE1-1-Straighten.tif]

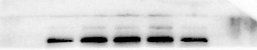

Supplement: Supplementary file 2 [file DataSheet8.zip › Figure 6/Figure 6F/IRE1-1-Straighten.tif]

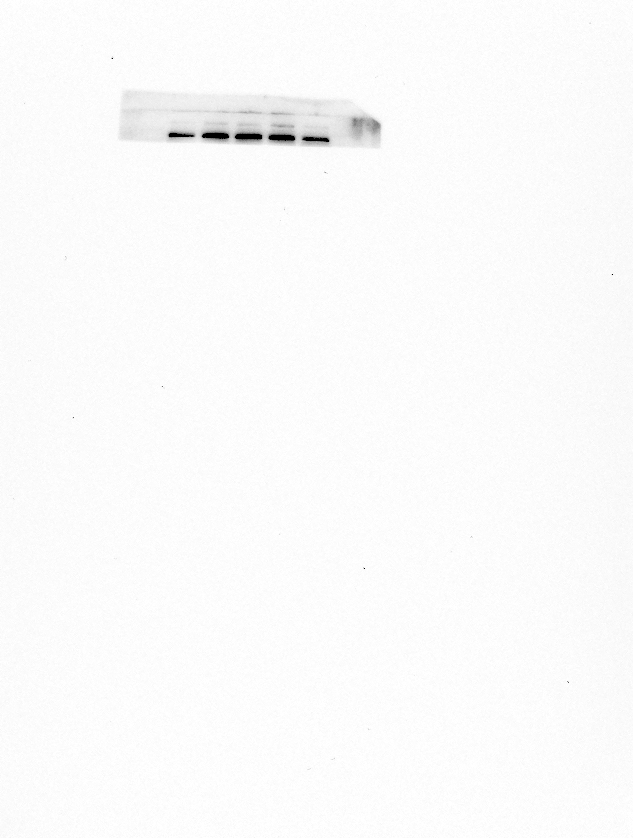

Supplement: Supplementary file 2 [file DataSheet8.zip › Figure 6/Figure 6F/ire1-1-c.h.h+p200.h+p200+simzb1.h+p200+sinc.tif]

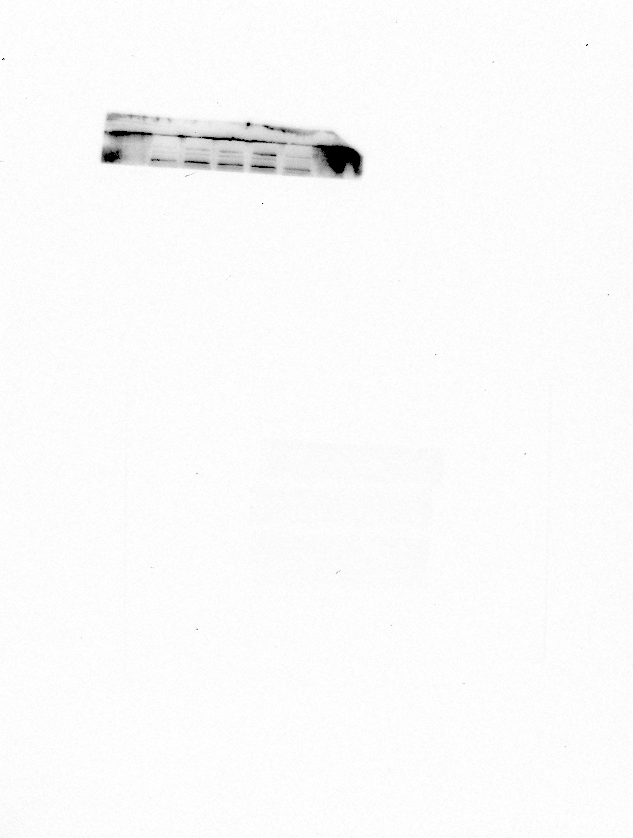

Supplement: Supplementary file 2 [file DataSheet8.zip › Figure 6/Figure 6F/p-ire1-1-c.h.h+p200.h+p200+simzb1.h+p200+sinc.tif]

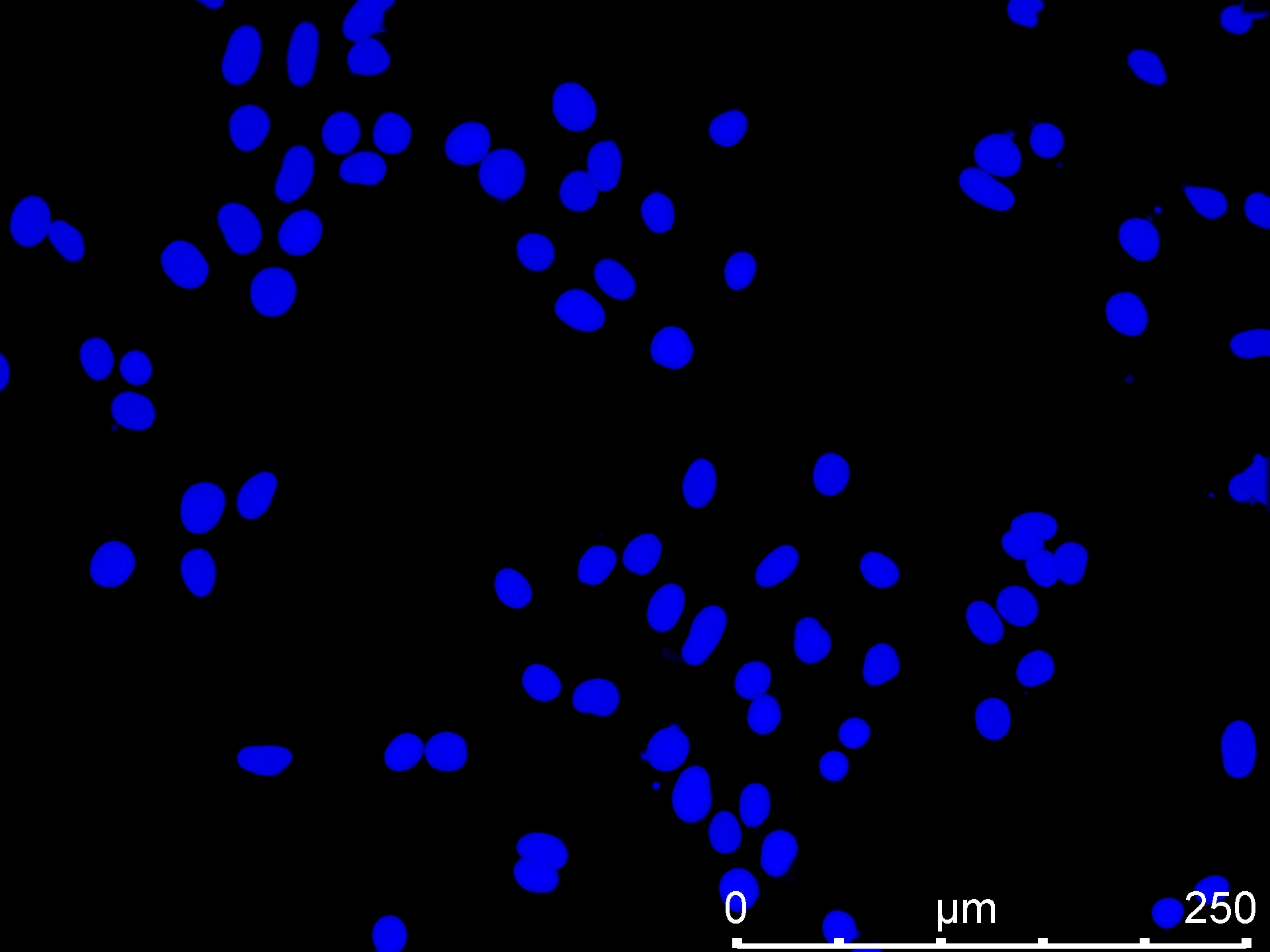

Supplement: Supplementary file 2 [file DataSheet8.zip › Figure 6/Figure 6A/H+P200+si-nc_15-1.tif]

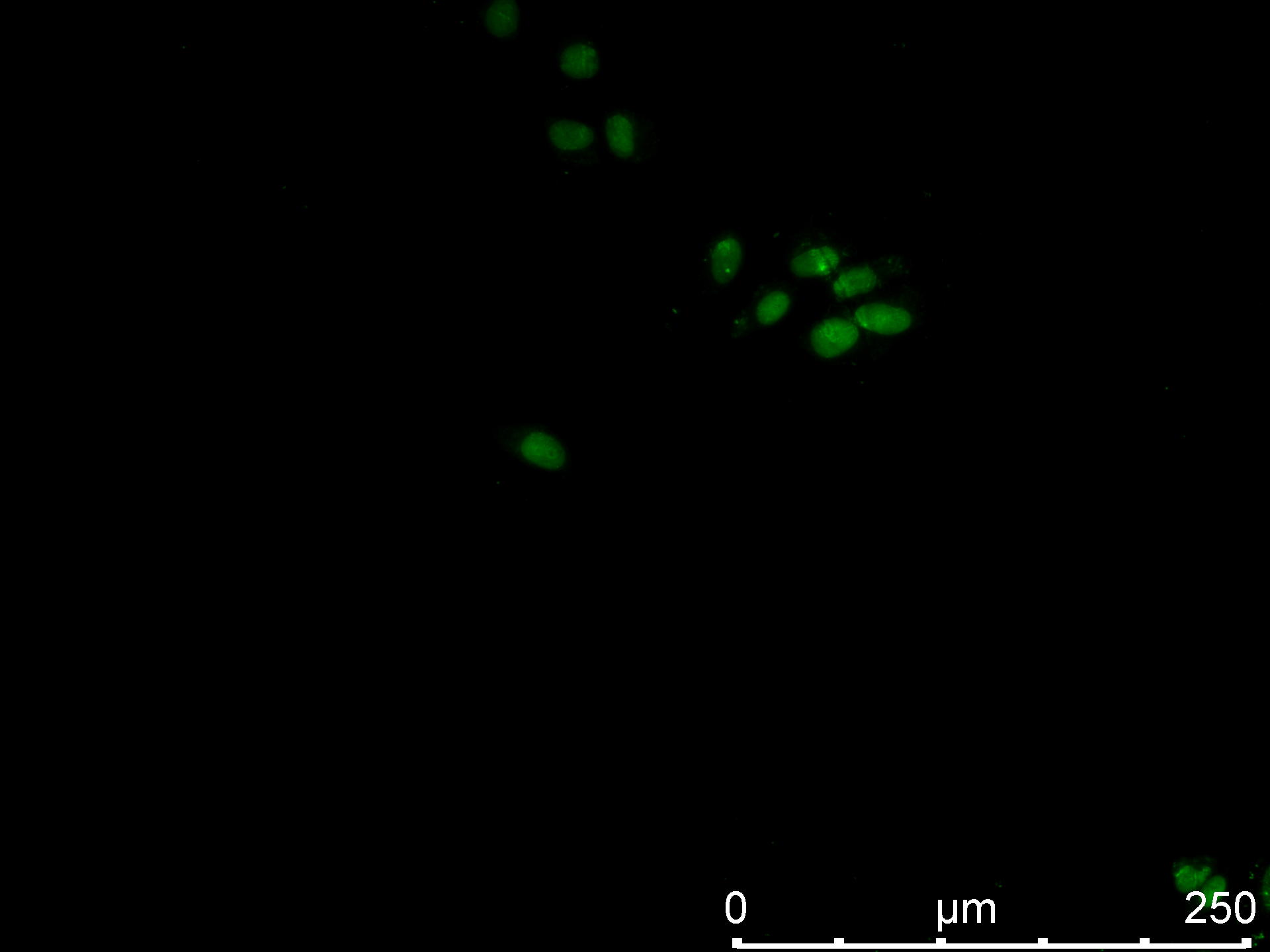

Supplement: Supplementary file 2 [file DataSheet8.zip › Figure 6/Figure 6A/h+p200_11-2.tif]

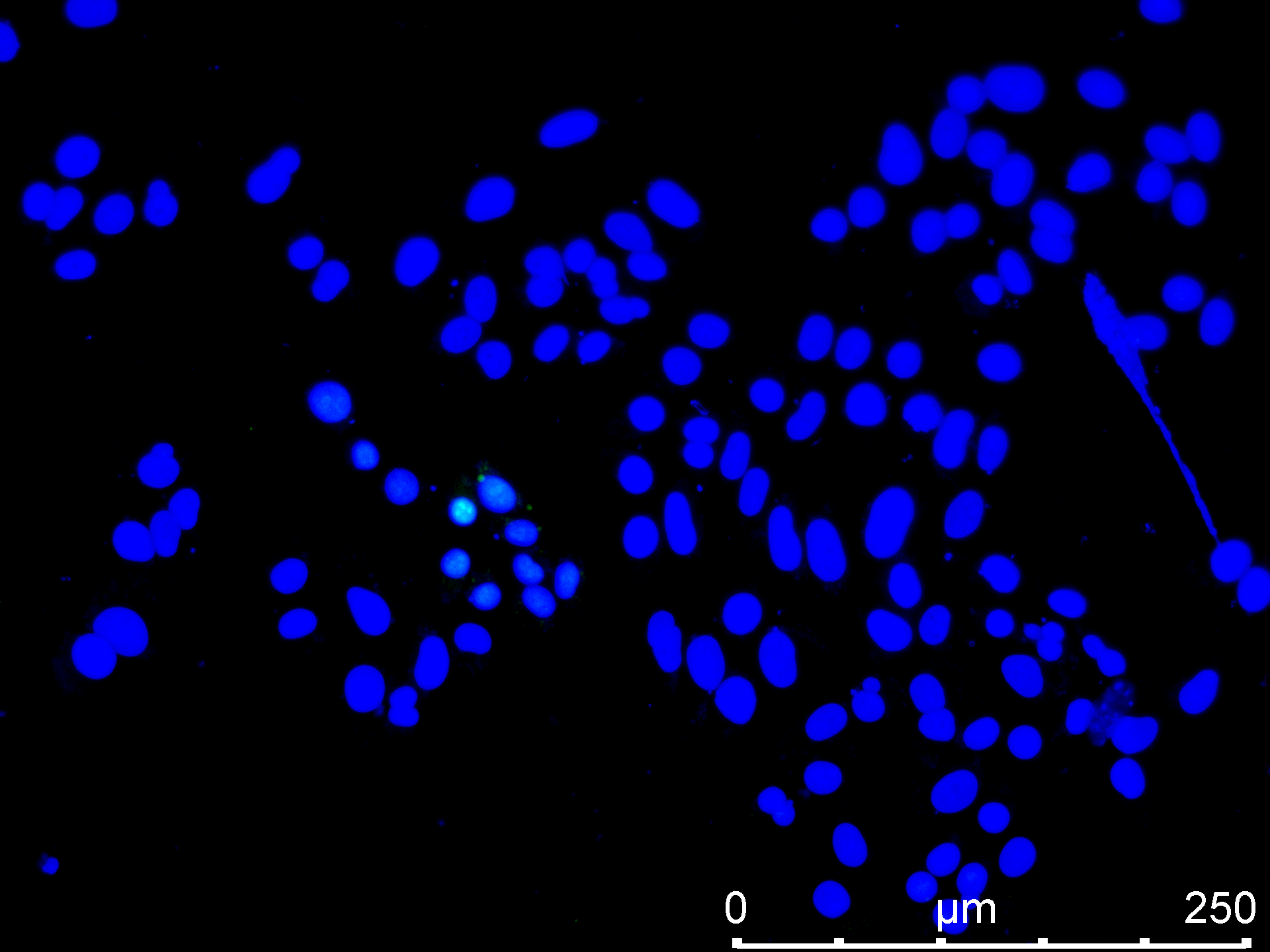

Supplement: Supplementary file 2 [file DataSheet8.zip › Figure 6/Figure 6A/CTL_5.tif]

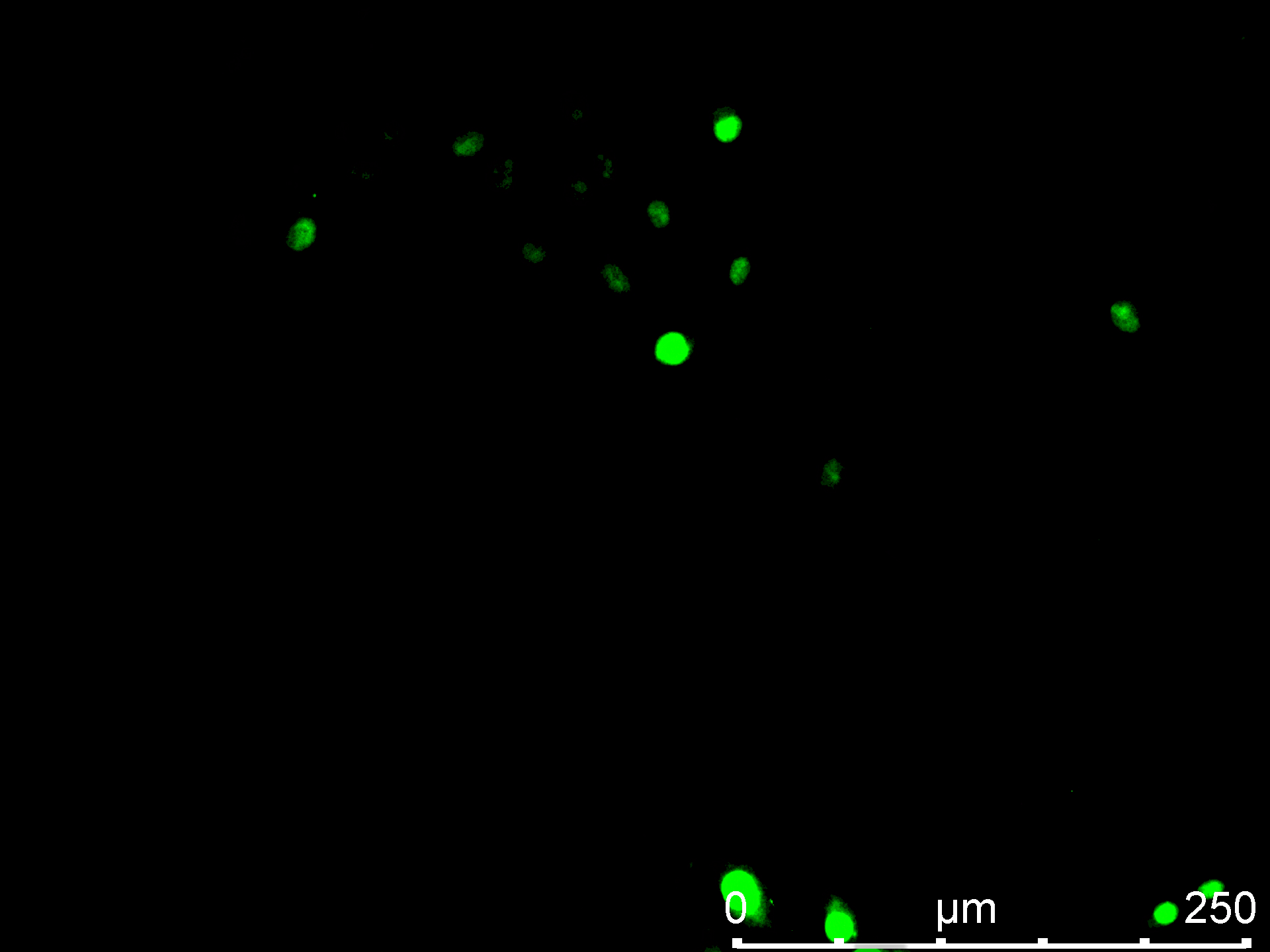

Supplement: Supplementary file 2 [file DataSheet8.zip › Figure 6/Figure 6A/H+P200+si-nc_15-2.tif]

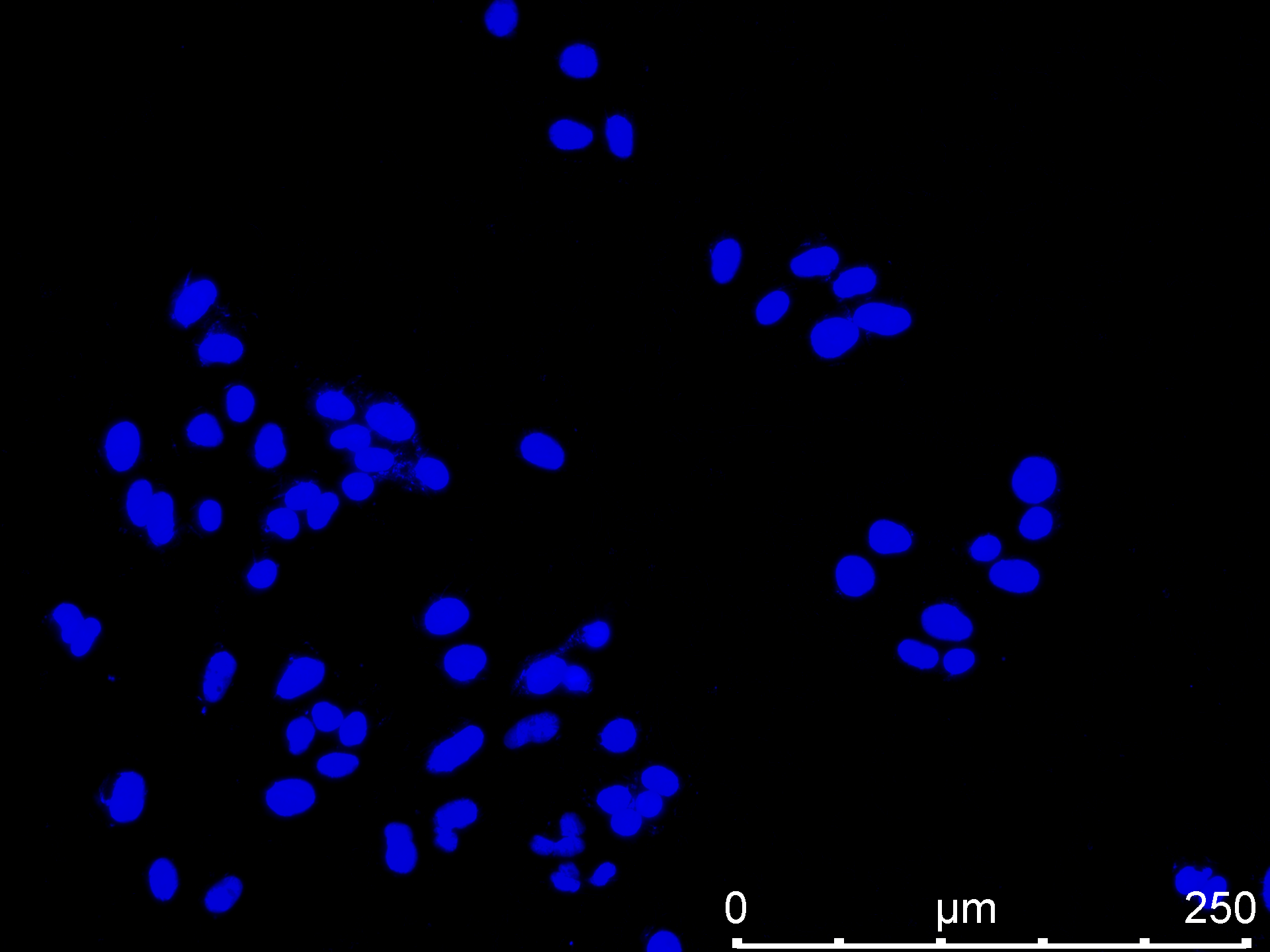

Supplement: Supplementary file 2 [file DataSheet8.zip › Figure 6/Figure 6A/h+p200_11-1.tif]

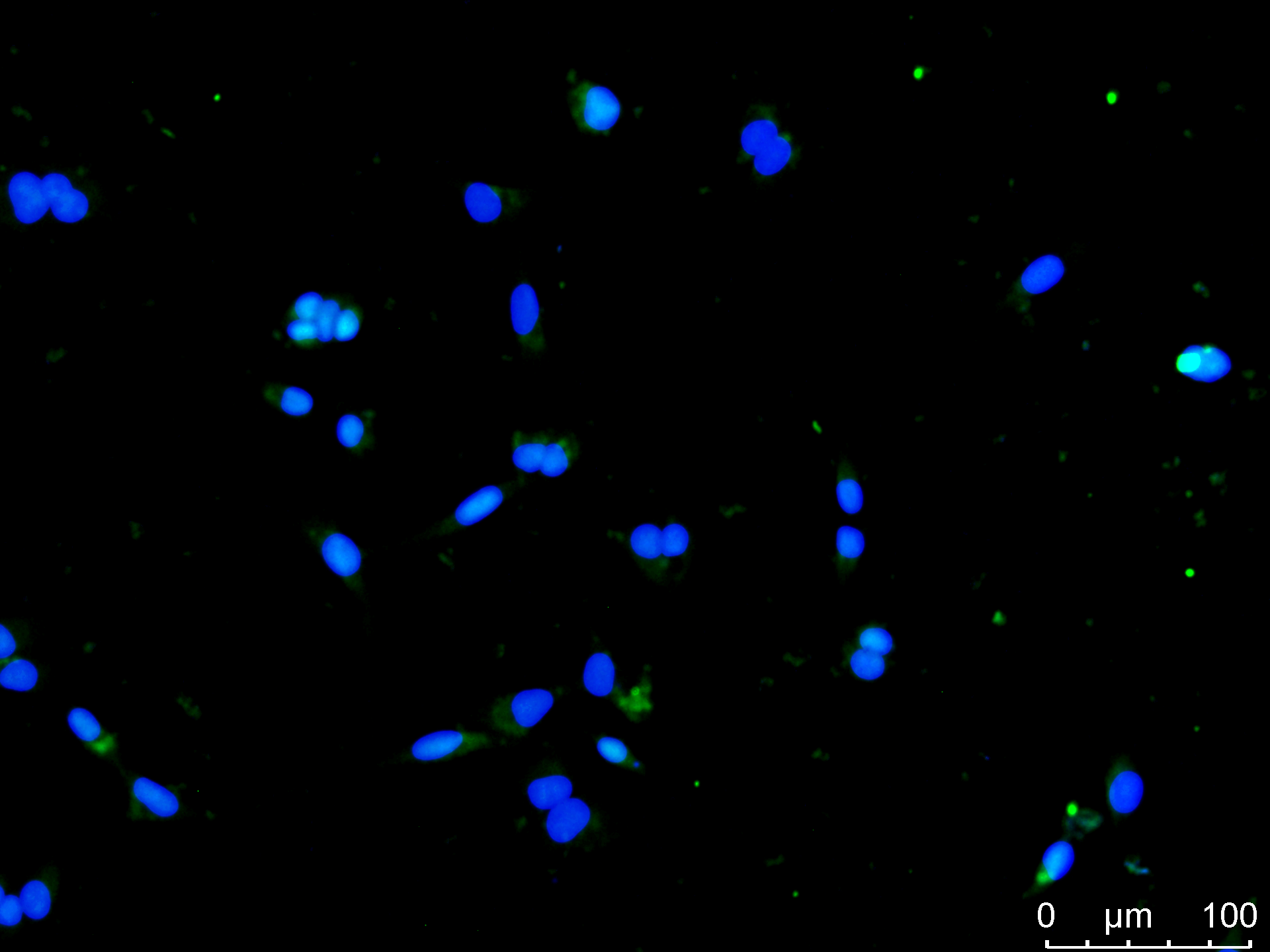

Supplement: Supplementary file 2 [file DataSheet8.zip › Figure 6/Figure 6A/h2o2_13.tif]

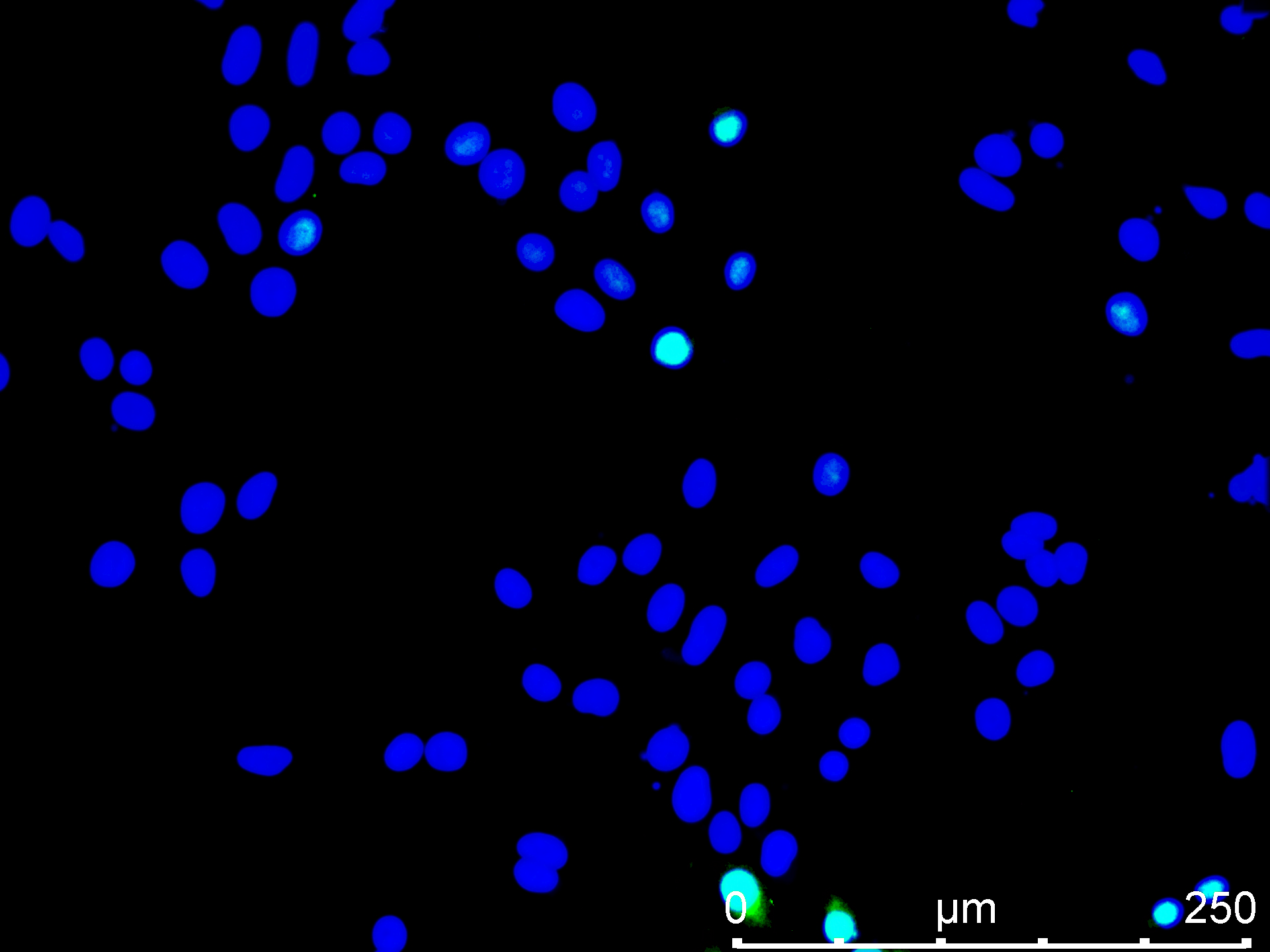

Supplement: Supplementary file 2 [file DataSheet8.zip › Figure 6/Figure 6A/H+P200+si-nc_15.tif]

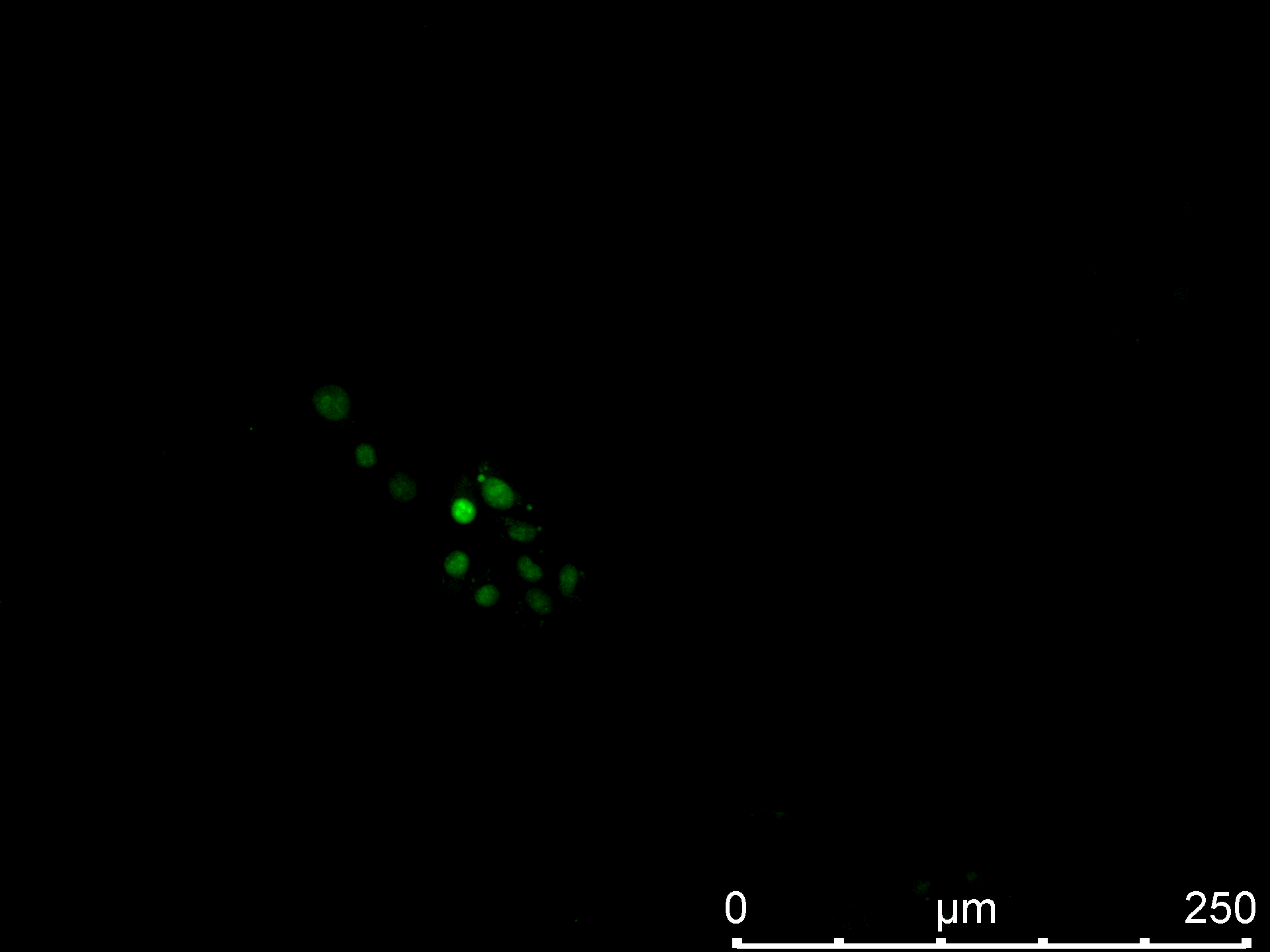

Supplement: Supplementary file 2 [file DataSheet8.zip › Figure 6/Figure 6A/CTL_5-2.tif]

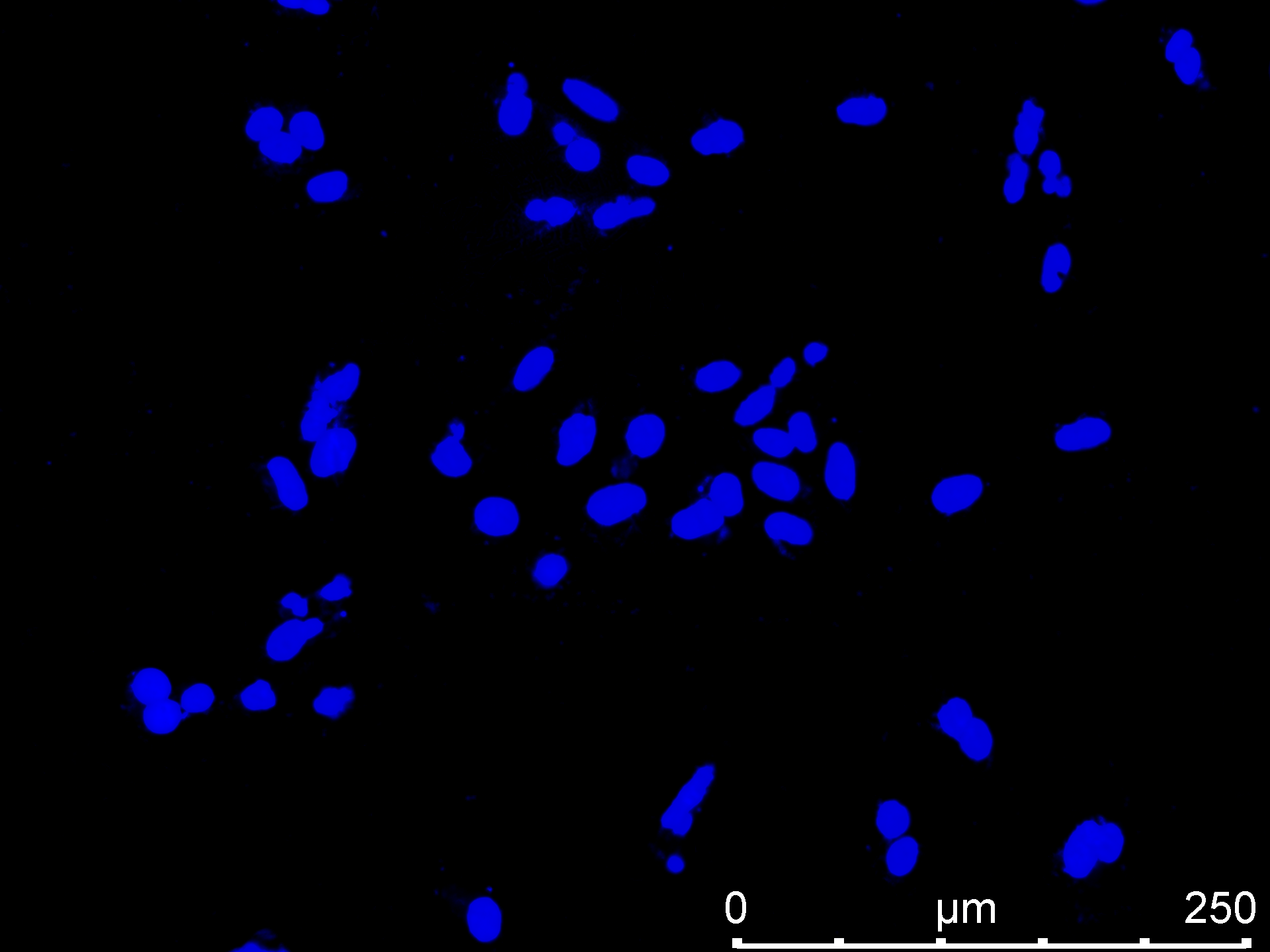

Supplement: Supplementary file 2 [file DataSheet8.zip › Figure 6/Figure 6A/h+p200+si-mzb1#3_12-1.tif]

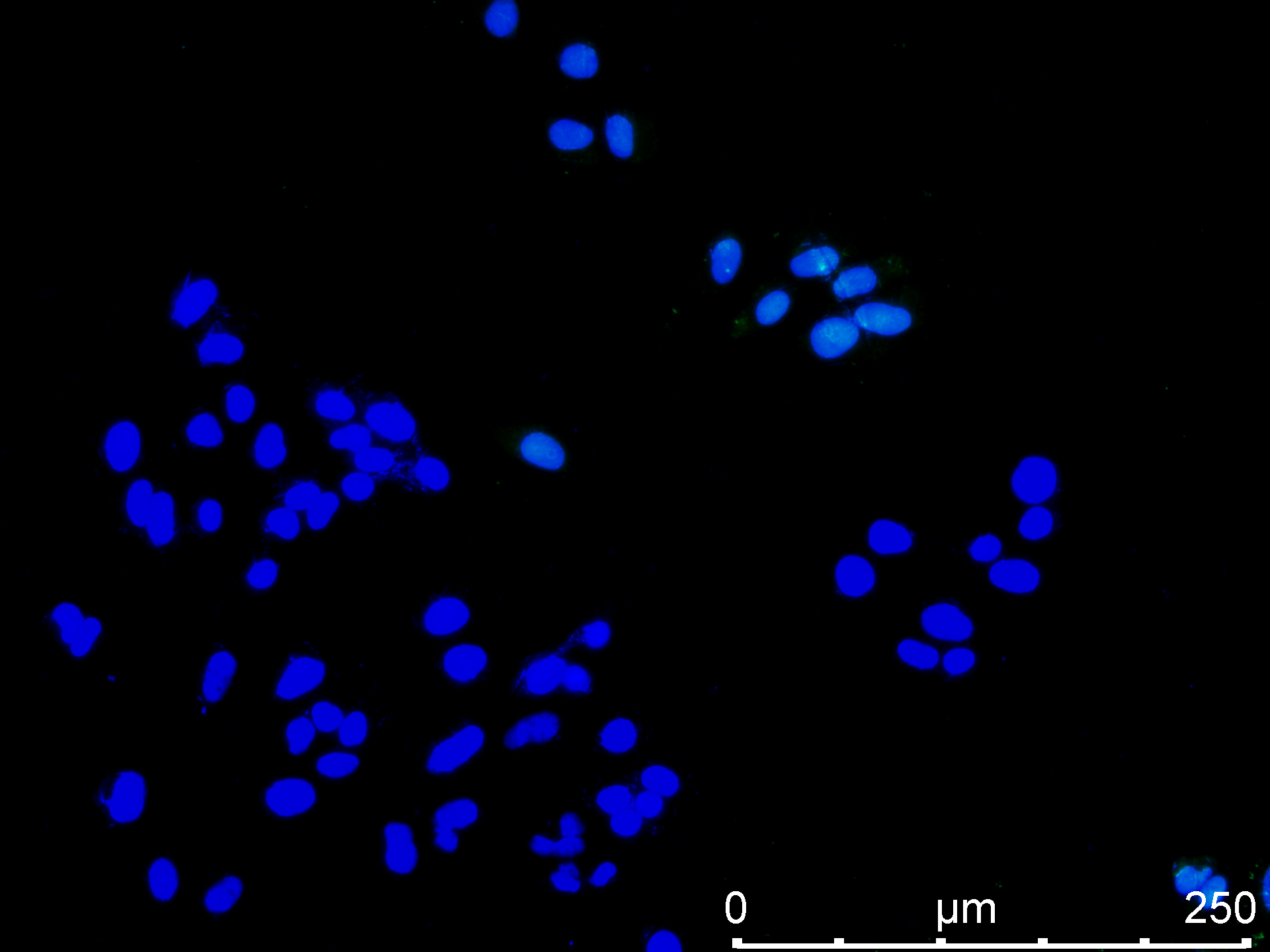

Supplement: Supplementary file 2 [file DataSheet8.zip › Figure 6/Figure 6A/h+p200_11.tif]

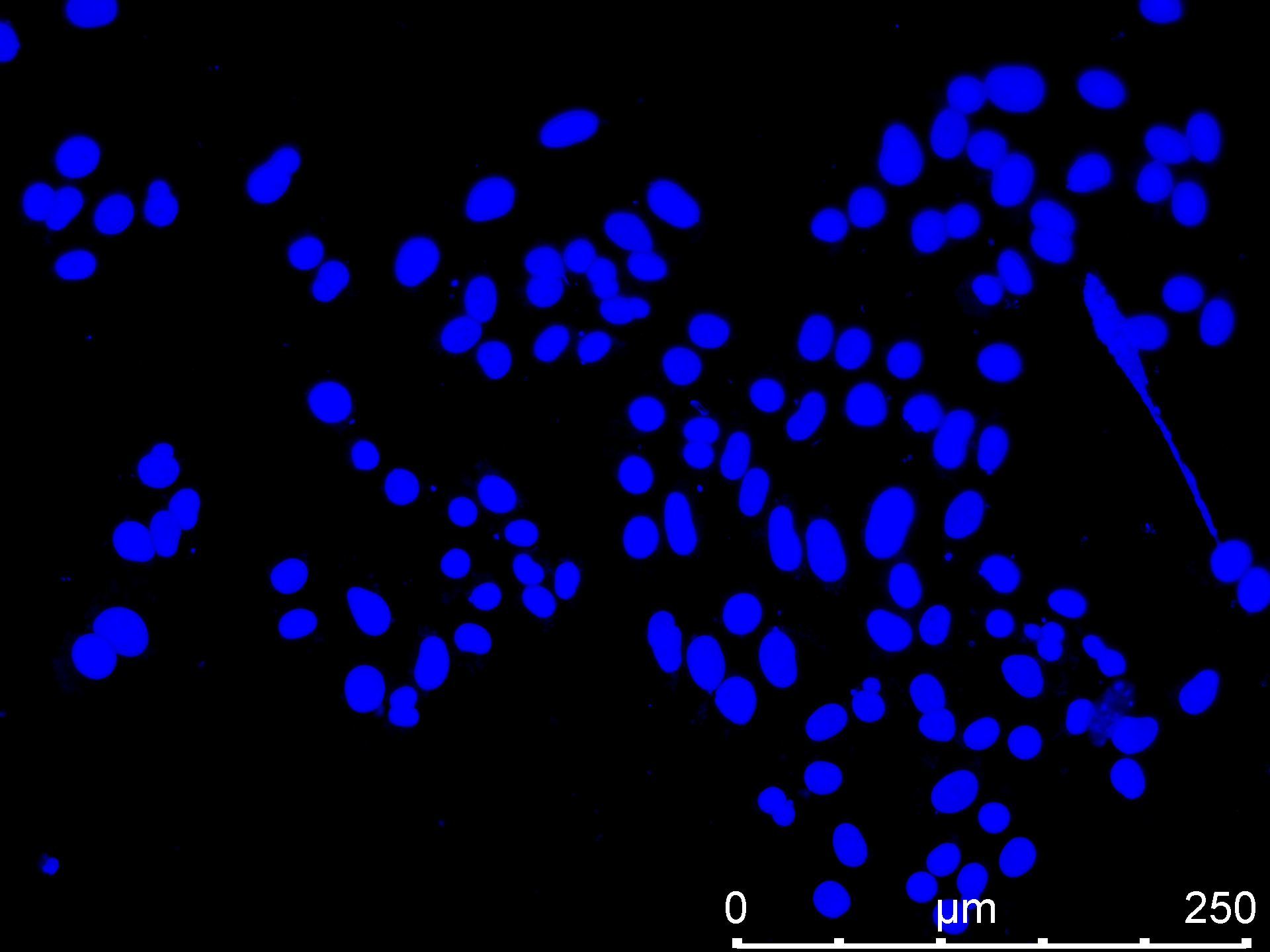

Supplement: Supplementary file 2 [file DataSheet8.zip › Figure 6/Figure 6A/CTL_5-1.tif]

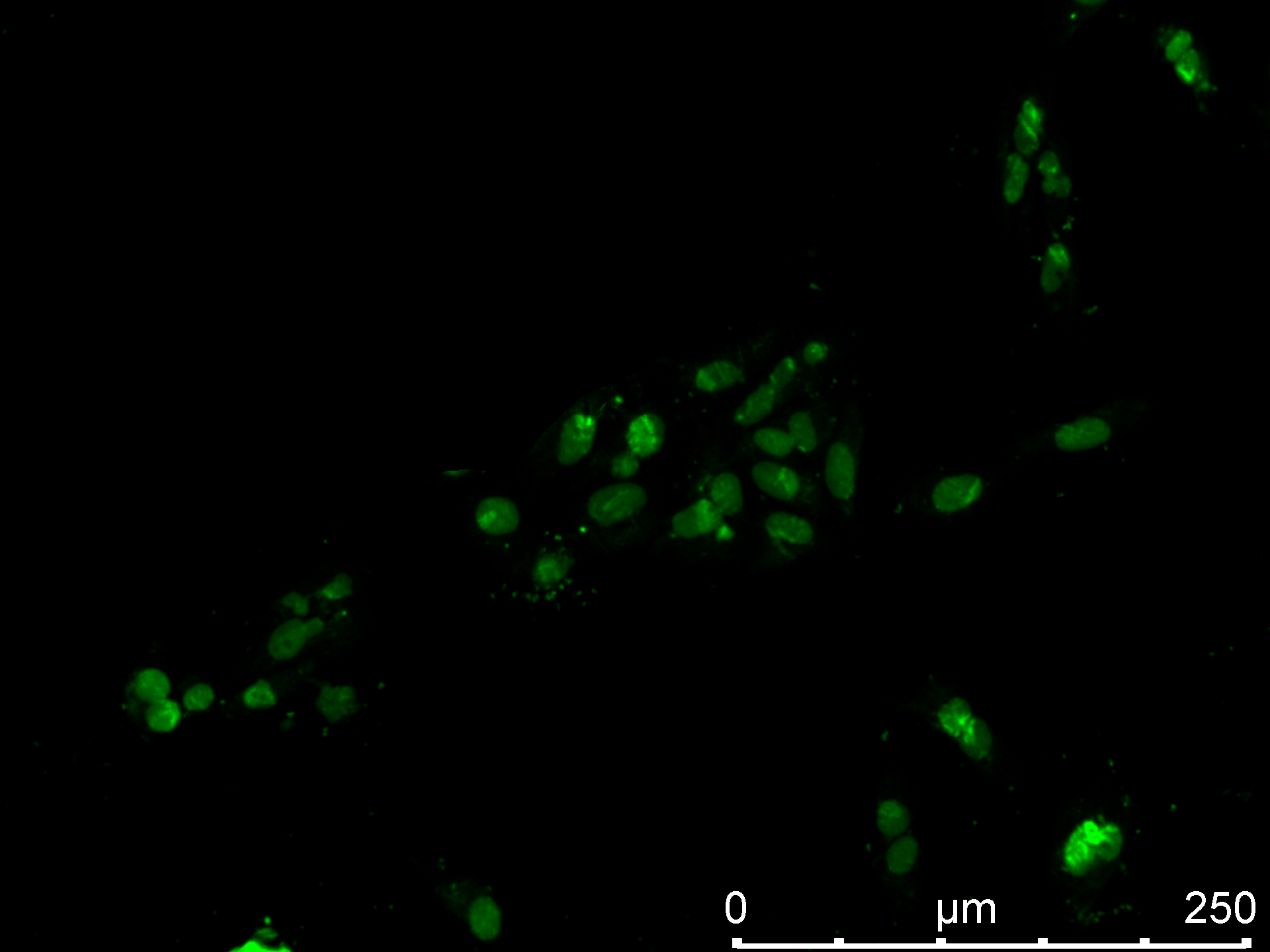

Supplement: Supplementary file 2 [file DataSheet8.zip › Figure 6/Figure 6A/h+p200+si-mzb1#3_12-2.tif]

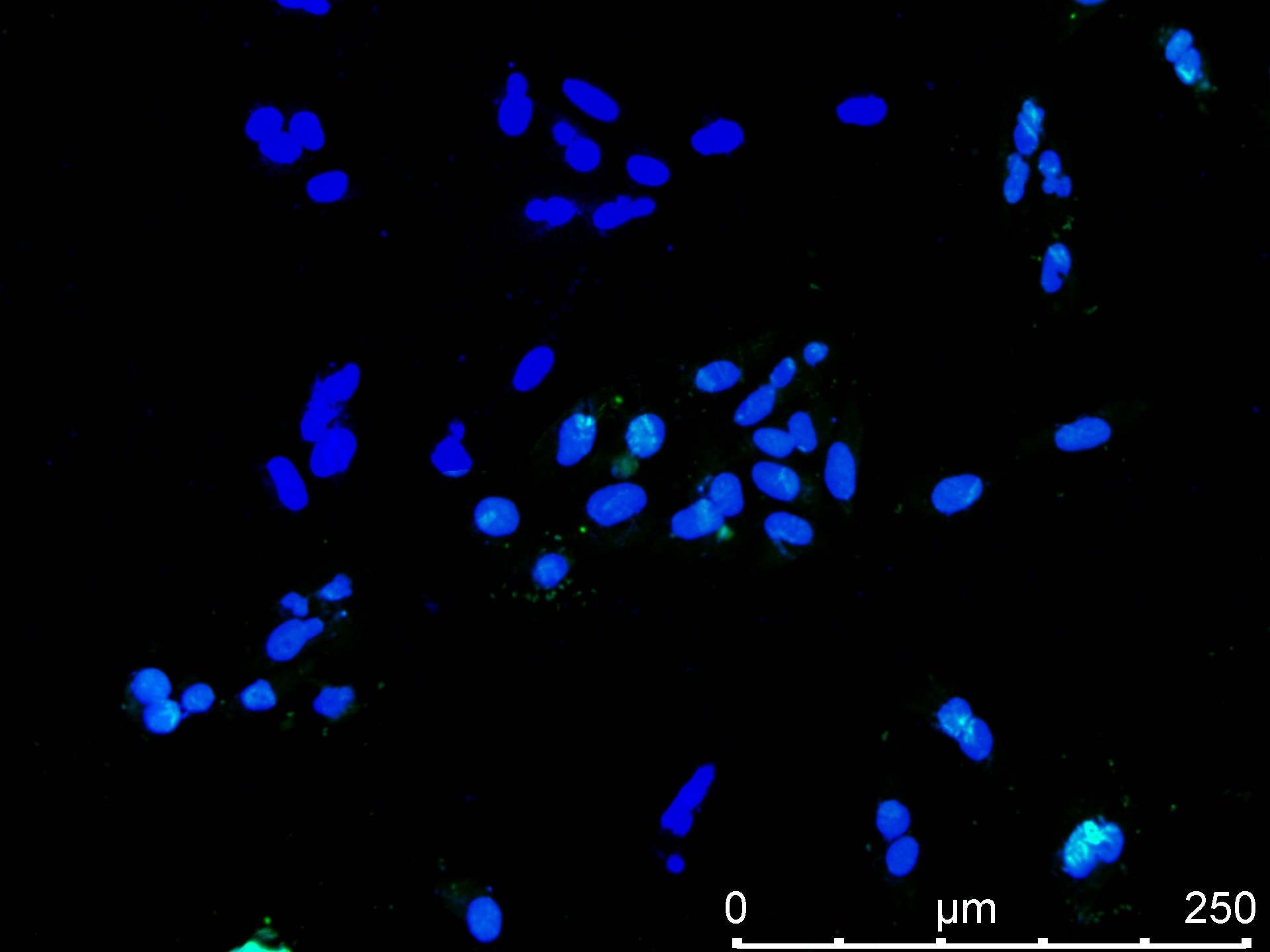

Supplement: Supplementary file 2 [file DataSheet8.zip › Figure 6/Figure 6A/h+p200+si-mzb1#3_12.tif]

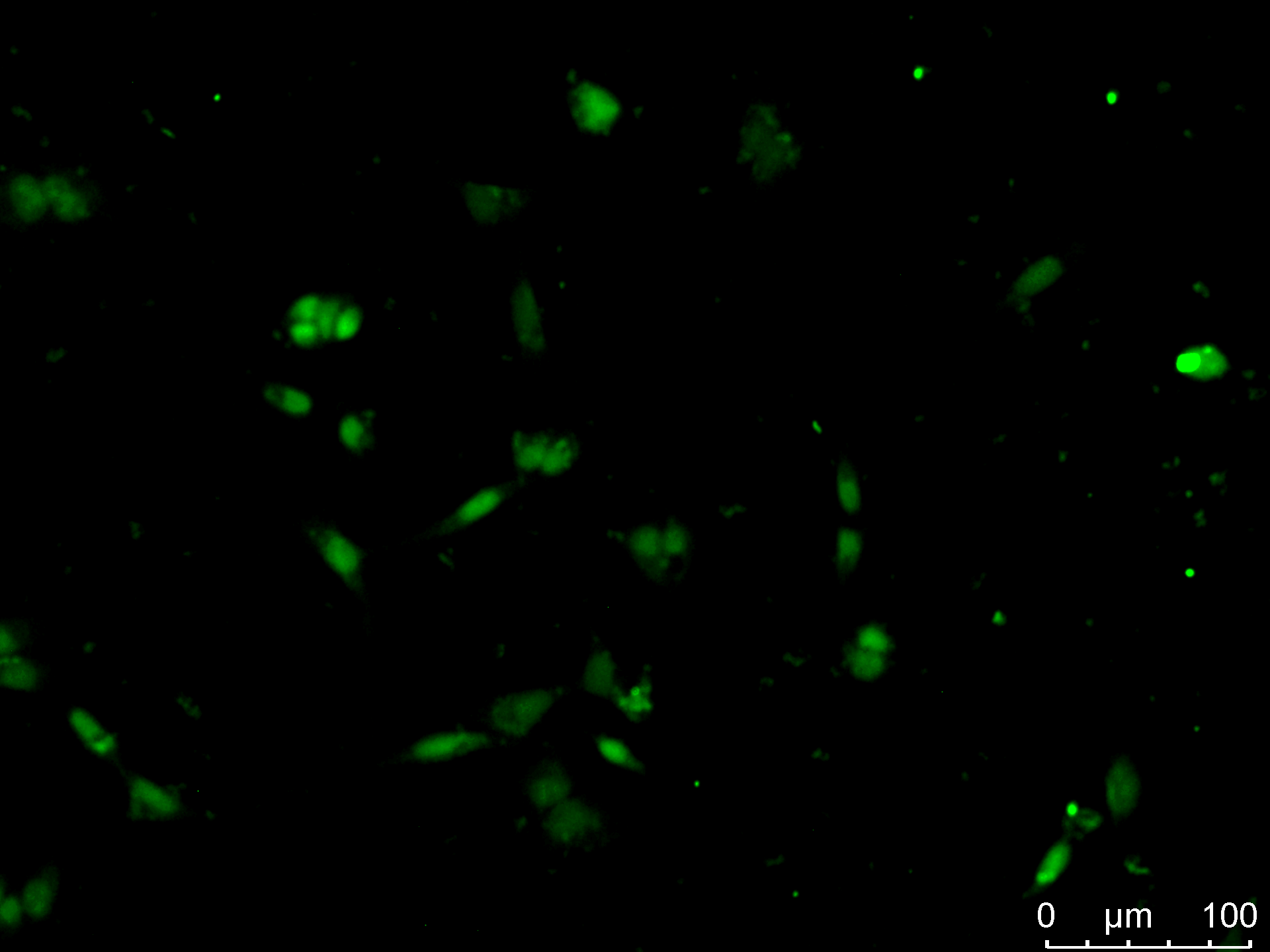

Supplement: Supplementary file 2 [file DataSheet8.zip › Figure 6/Figure 6A/h2o2_13-2.tif]

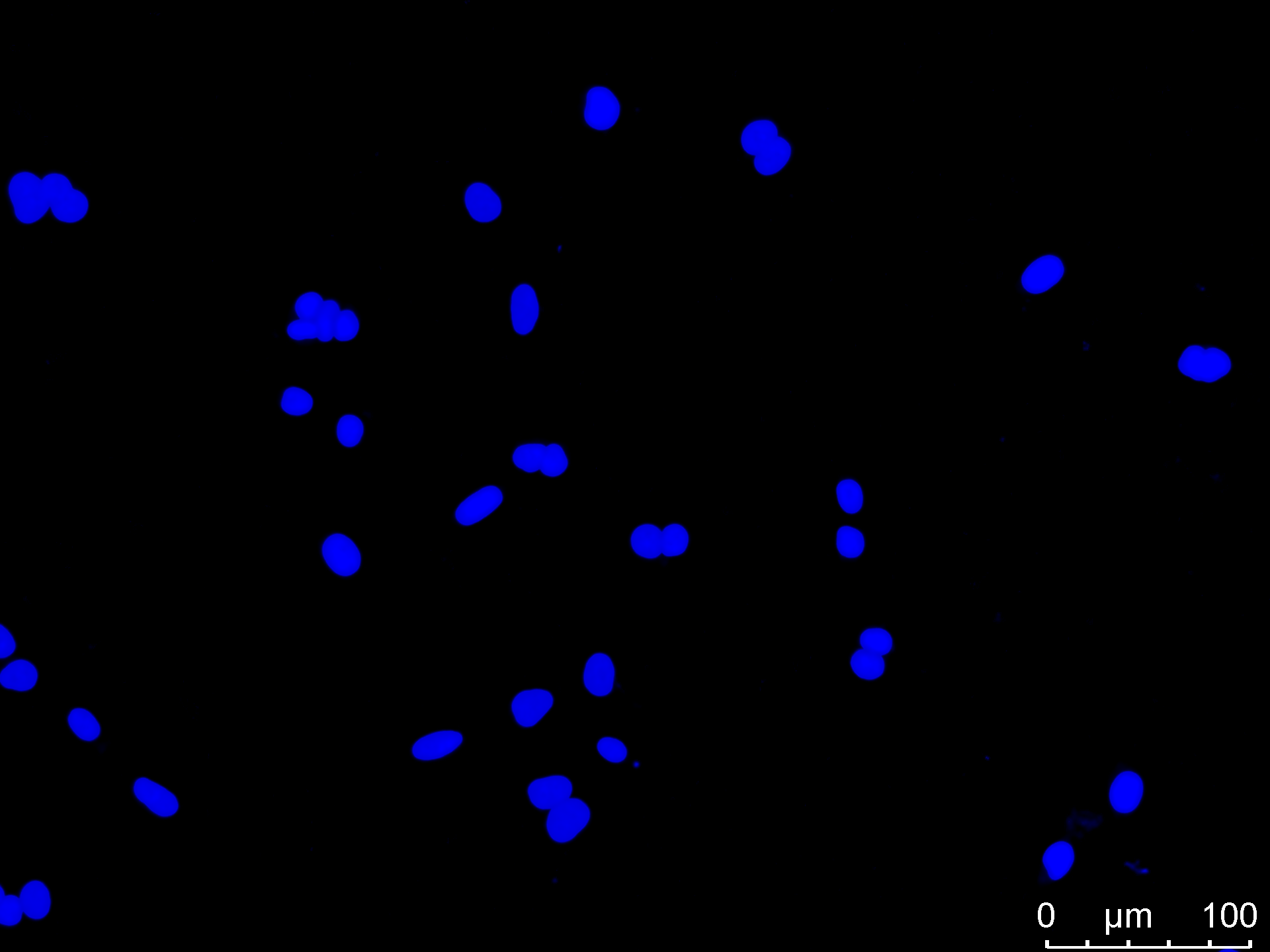

Supplement: Supplementary file 2 [file DataSheet8.zip › Figure 6/Figure 6A/h2o2_13-1.tif]

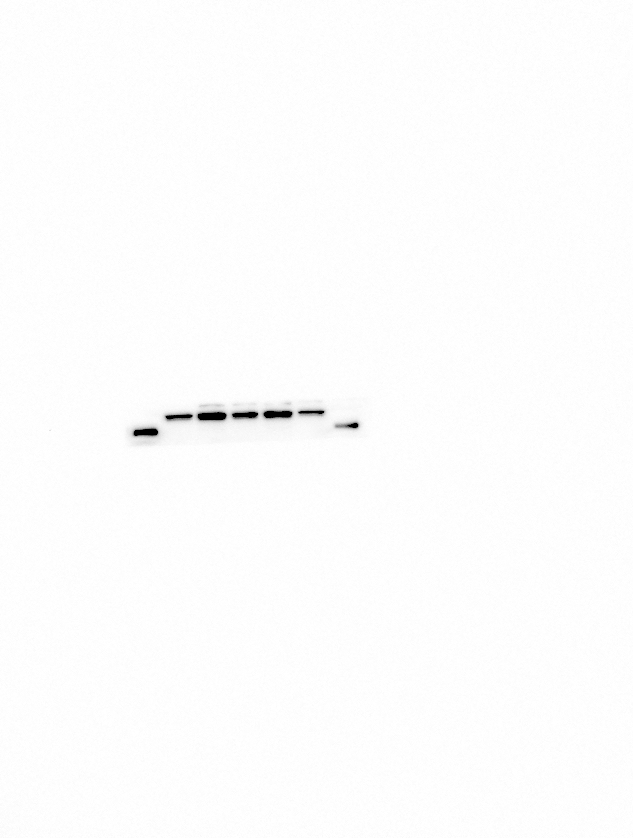

Supplement: Supplementary file 2 [file DataSheet8.zip › Figure 6/Figure 6C/grp78-2.tif]

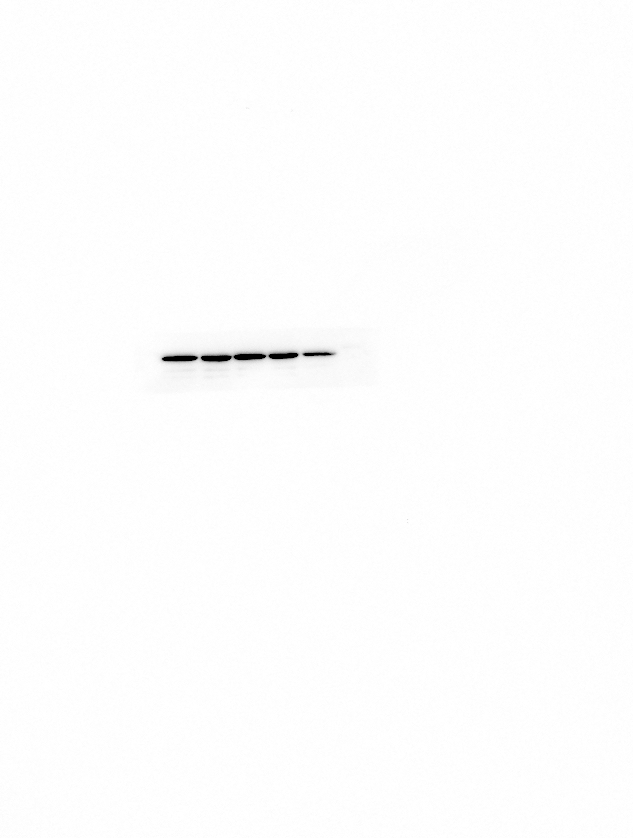

Supplement: Supplementary file 2 [file DataSheet8.zip › Figure 6/Figure 6C/gapdh-2.tif]

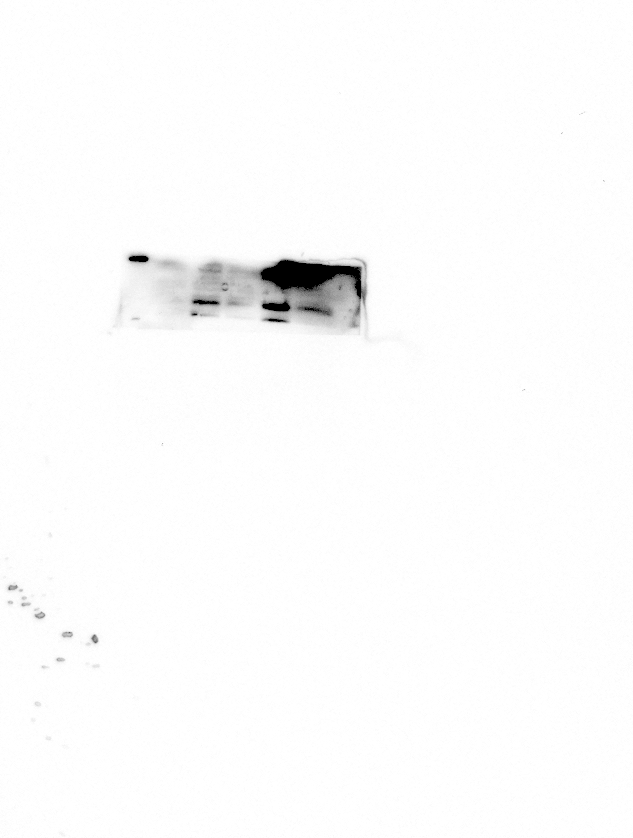

Supplement: Supplementary file 2 [file DataSheet8.zip › Figure 6/Figure 6C/chop-2.tif]

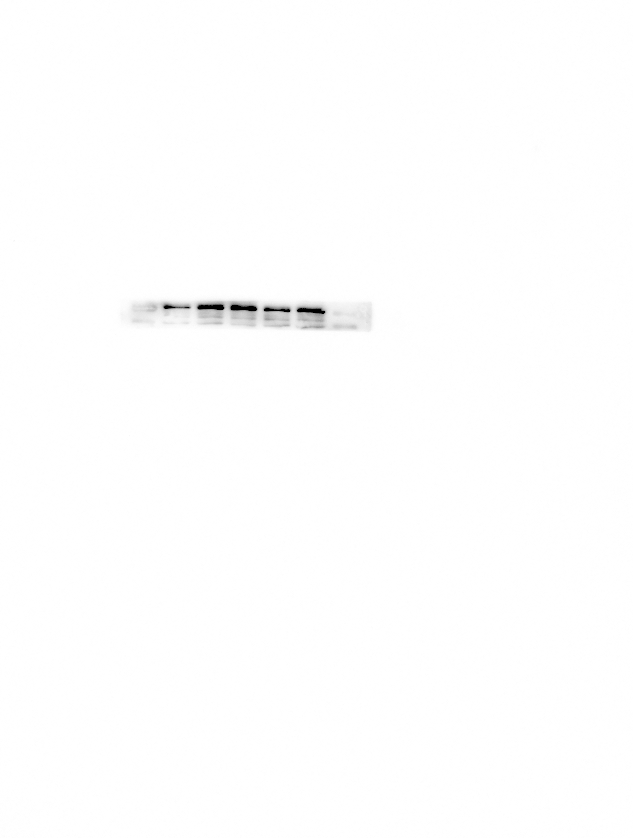

Supplement: Supplementary file 3 [file DataSheet9.zip › Figure 7/Figure 7D/drp1-1.tif]

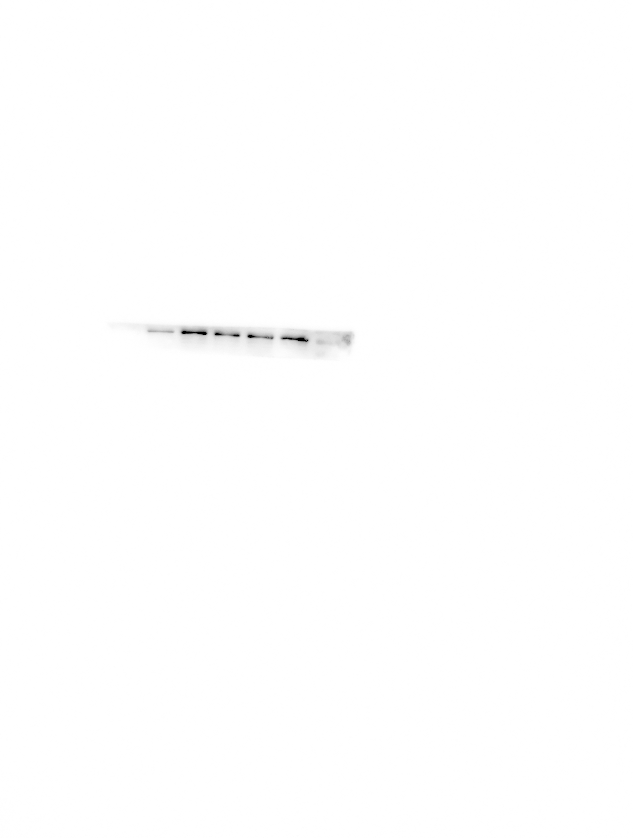

Supplement: Supplementary file 3 [file DataSheet9.zip › Figure 7/Figure 7D/p-drp1-1.tif]

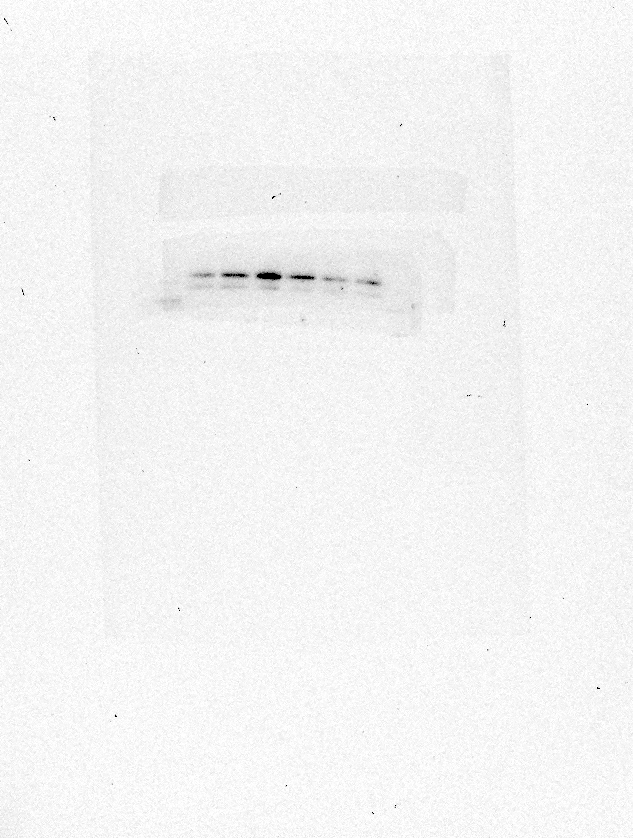

Supplement: Supplementary file 3 [file DataSheet9.zip › Figure 7/Figure 7C/p-drp1-1-cπÇüτö▓∩╝îh∩╝î50.100.200.tif]

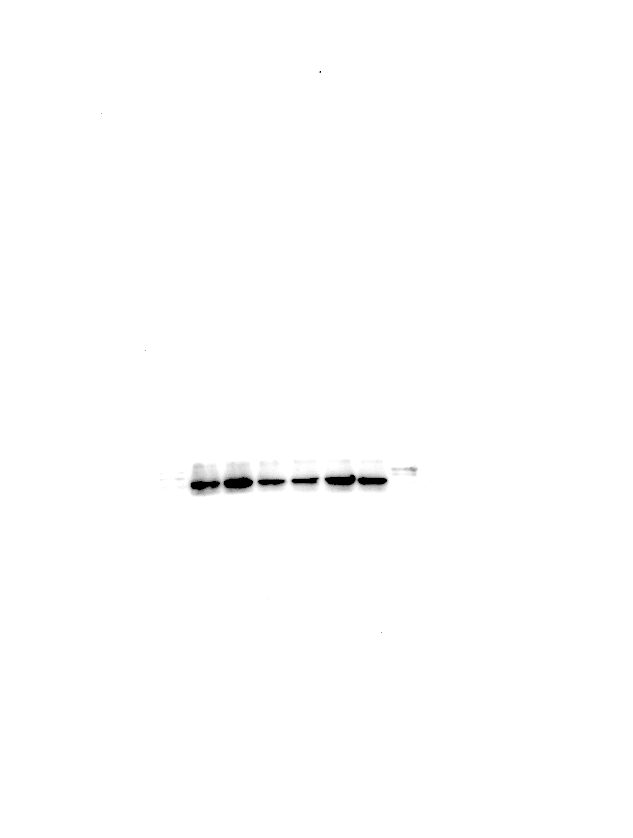

Supplement: Supplementary file 3 [file DataSheet9.zip › Figure 7/Figure 7C/drp1_1-cπÇüτö▓∩╝îh∩╝î50.100.200.tif]

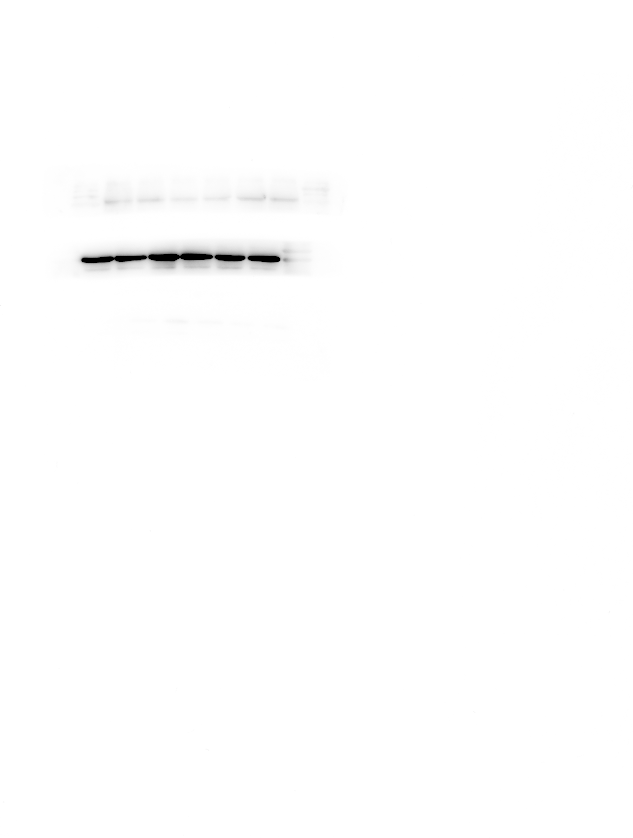

Supplement: Supplementary file 3 [file DataSheet9.zip › Figure 7/Figure 7C/drp1,gapdh,mzb1-1-cπÇüτö▓∩╝îh∩╝î50.100.200.tif]

Figure 2G-H

Sham   AMI+   AMI+   AMI+  
Vec   Pue50   Pue100

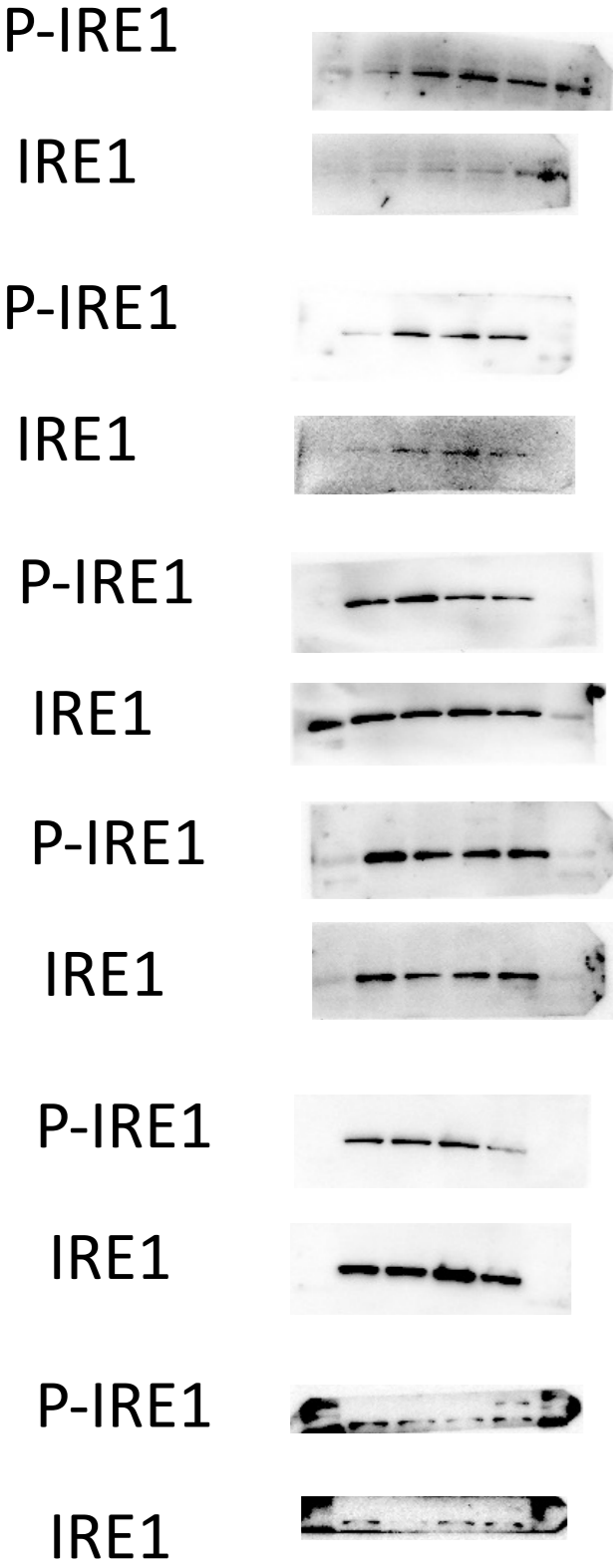

| P-IRE1/IRE1 | Sham | AMI+<br>Vec | AMI+<br>Pue50 | AMI+<br>Pue100 |
|-------------|------|-------------|---------------|----------------|
|             | 1    | 1.314808055 | 1.03050376    | 0.839703409    |
|             | 1    | 1.206772791 | 0.850997745   | 0.767426473    |
|             | 1    | 1.239450534 | 0.888878976   | 0.587244995    |
|             | 1    | 1.726860091 | 0.468957684   | 0.425073635    |
|             | 1    | 1.477382497 | 0.721078459   | 0.655609811    |
|             | 1    | 1.630549168 | 1.302488444   | 1.157057319    |

Supplement: Supplementary file 4 [file DataSheet4.zip › Figure 2/Figure 2G/2G data.pdf]

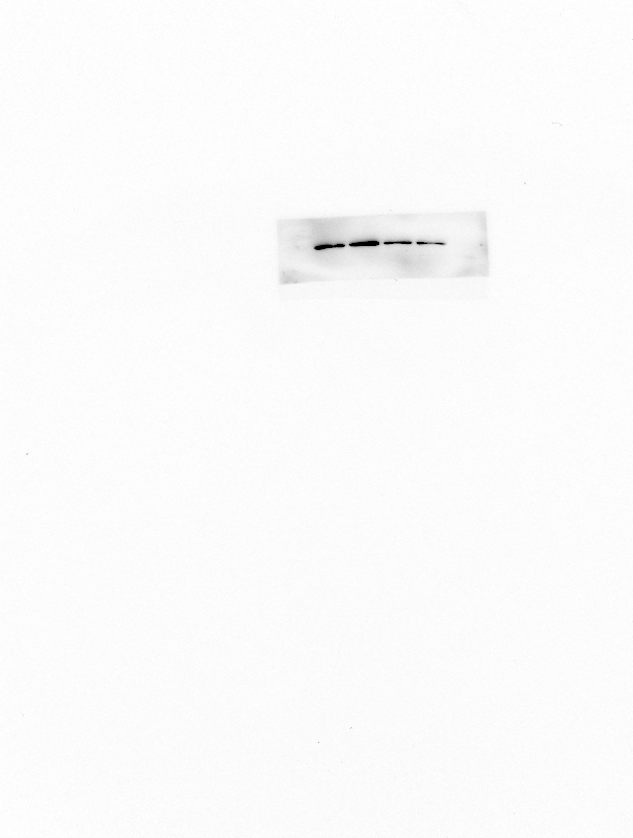

Supplement: Supplementary file 4 [file DataSheet4.zip › Figure 2/Figure 2G/p-ire1-2.tif]

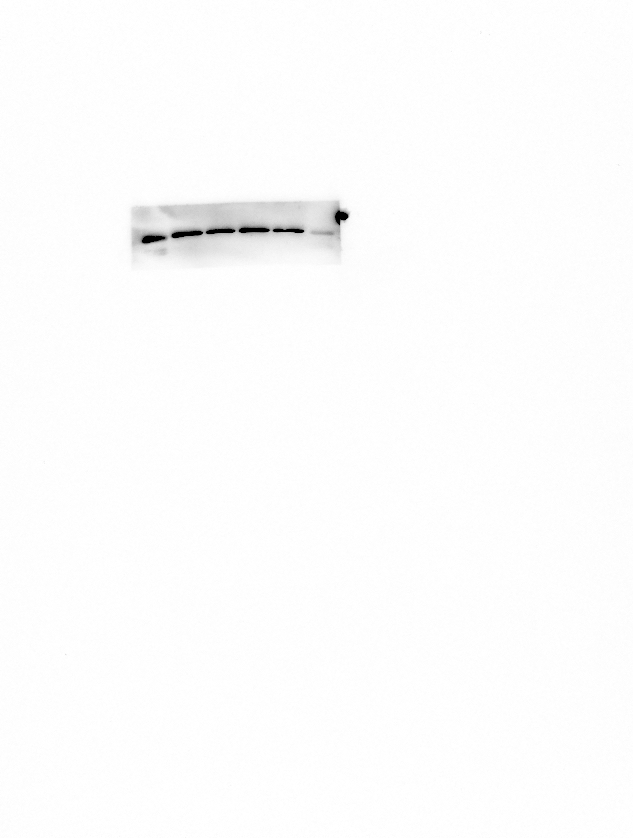

Supplement: Supplementary file 4 [file DataSheet4.zip › Figure 2/Figure 2G/ire1-2.tif]

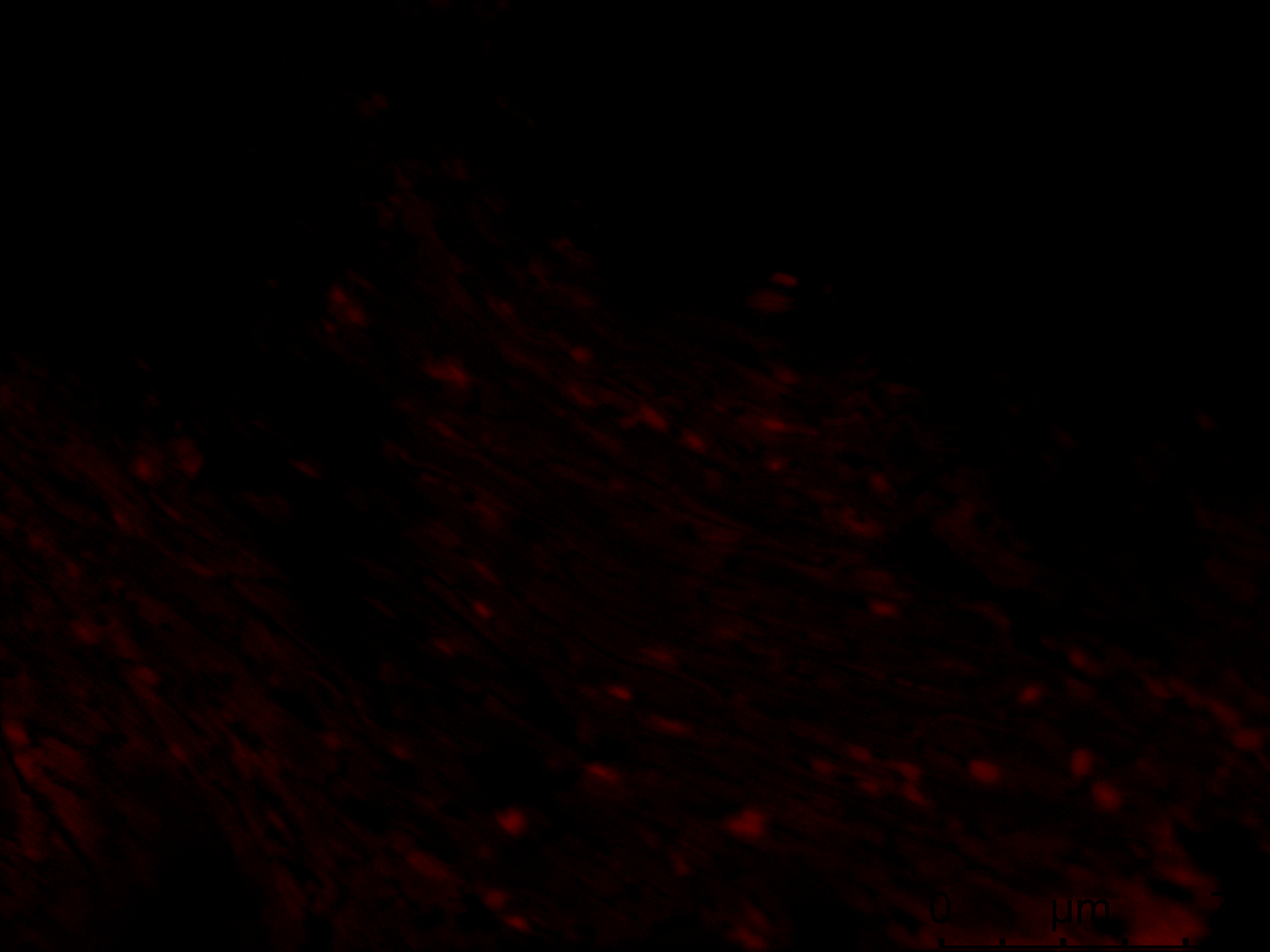

Supplement: Supplementary file 4 [file DataSheet4.zip › Figure 2/Figure 2A/p50-4_Image006.tif]

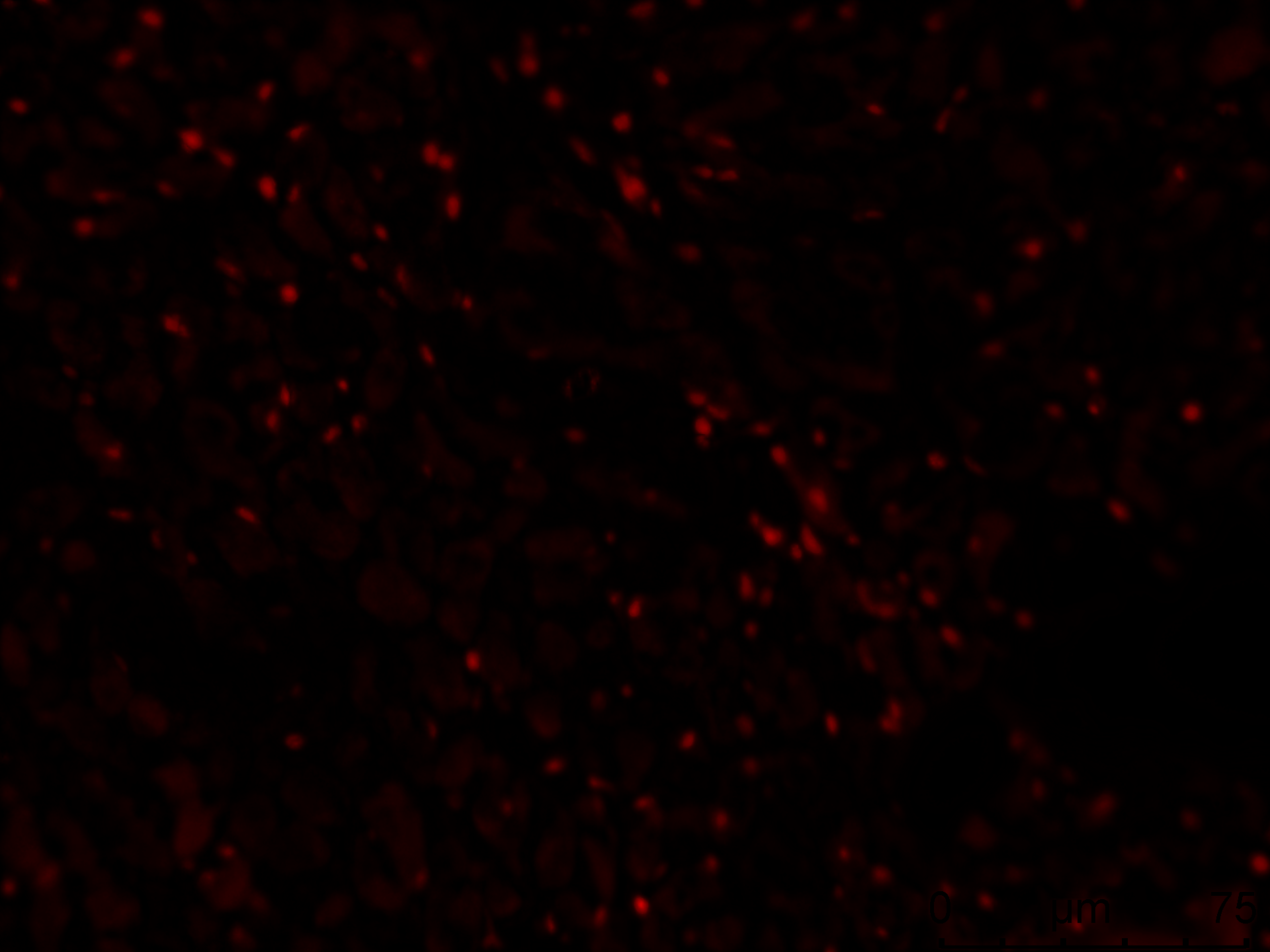

Supplement: Supplementary file 4 [file DataSheet4.zip › Figure 2/Figure 2A/ami-1_Image024.tif]

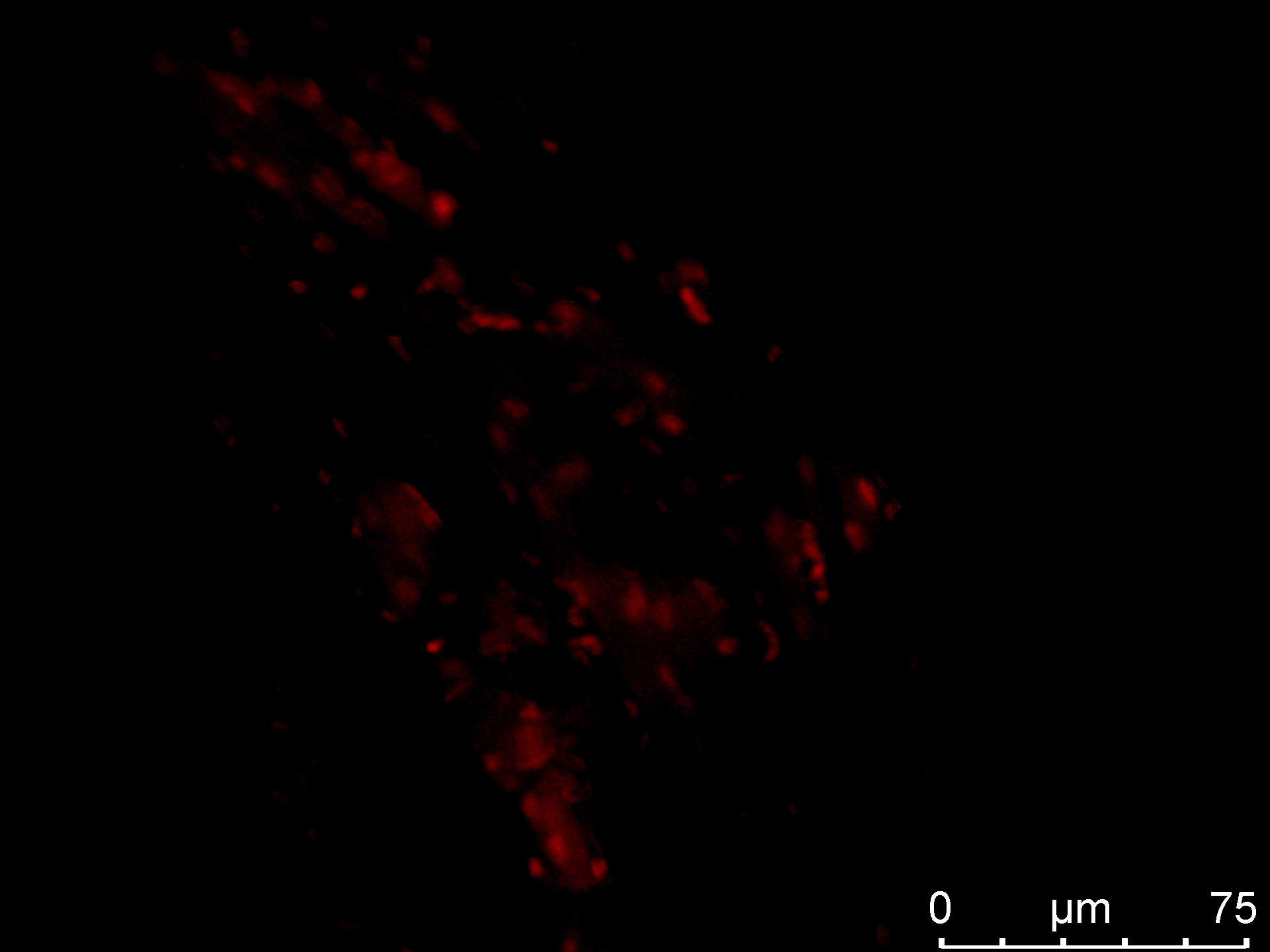

Supplement: Supplementary file 4 [file DataSheet4.zip › Figure 2/Figure 2A/P100-4_Image012.tif]

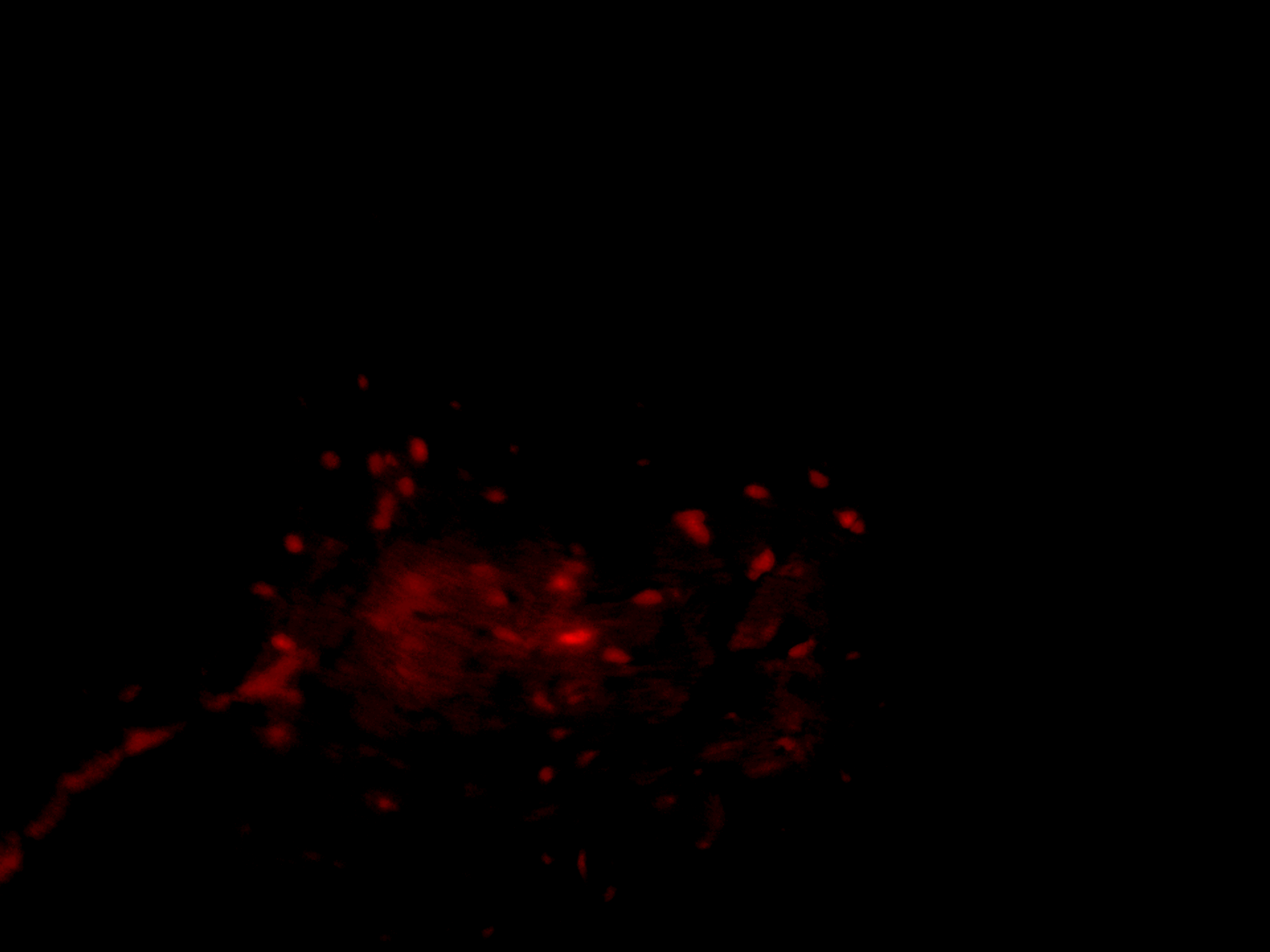

Supplement: Supplementary file 4 [file DataSheet4.zip › Figure 2/Figure 2A/ctl-3_Image025.tif]

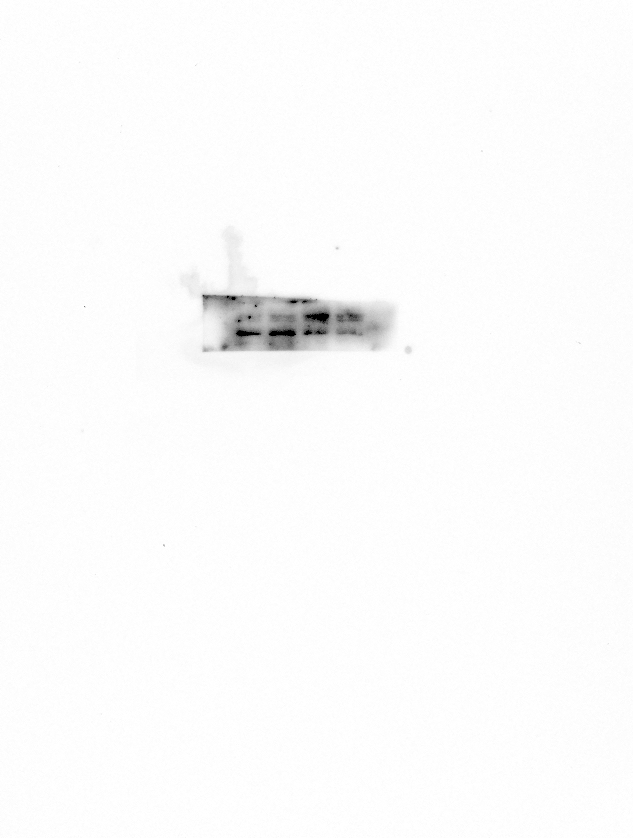

Supplement: Supplementary file 4 [file DataSheet4.zip › Figure 2/Figure 2D/chop.tif]

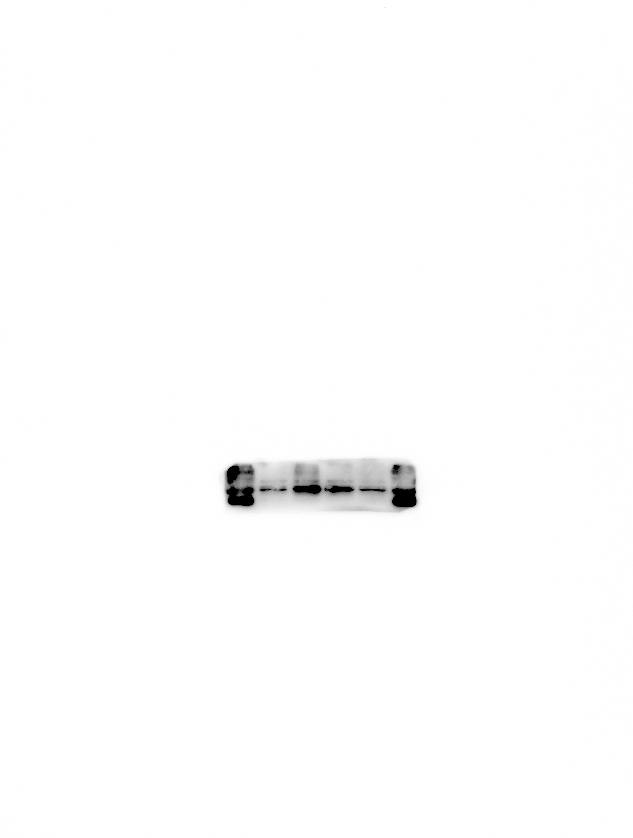

Supplement: Supplementary file 4 [file DataSheet4.zip › Figure 2/Figure 2D/grp78.tif]

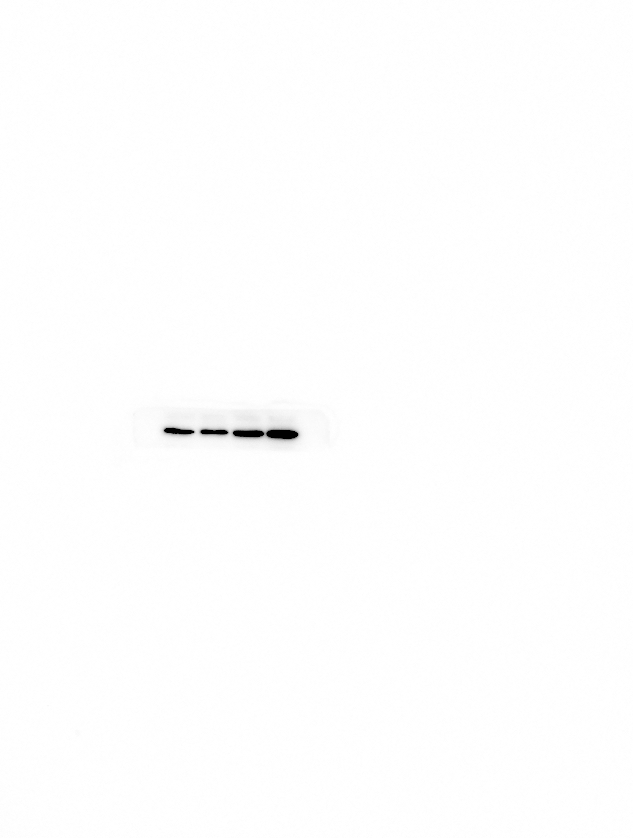

Supplement: Supplementary file 4 [file DataSheet4.zip › Figure 2/Figure 2D/gapdh.tif]

Figure 2E-F

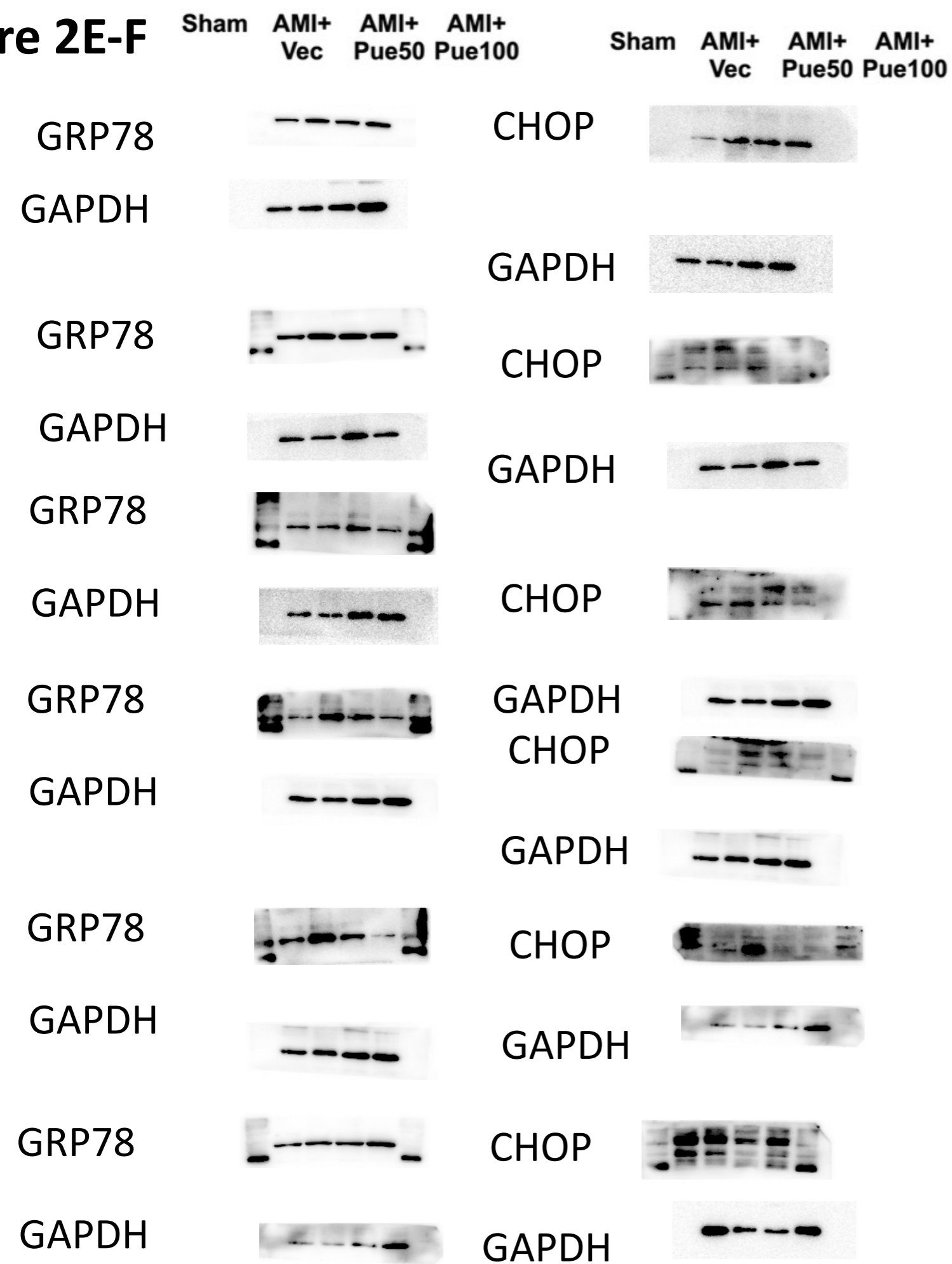

| GRP78 | Sham | AMI+ Vec    | AMI+ Pue50  | AMI+ Pue100 |
|-------|------|-------------|-------------|-------------|
|       | 1    | 1.60694155  | 1.2335149   | 1.00301228  |
|       | 1    | 1.739016627 | 1.229442636 | 0.978268893 |
|       | 1    | 1.877559649 | 1.162748643 | 1.433238538 |
|       | 1    | 2.360142764 | 1.471126126 | 1.056243445 |
|       | 1    | 2.129808609 | 1.349343214 | 0.737284754 |
|       | 1    | 1.736444401 | 1.449094759 | 1.259074831 |

| CHOP | Sham | AMI+ Vec    | AMI+ Pue50  | AMI+ Pue100 |
|------|------|-------------|-------------|-------------|
|      | 1    | 1.37731799  | 0.88918964  | 0.72450338  |
|      | 1    | 2.5011309   | 1.93880596  | 1.74302715  |
|      | 1    | 1.32501926  | 0.9647537   | 0.69107241  |
|      | 1    | 1.93931483  | 0.71094913  | 0.3698973   |
|      | 1    | 2.569811715 | 1.233615351 | 0.405939328 |
|      | 1    | 1.39123986  | 0.7844044   | 0.6586168   |

Supplement: Supplementary file 4 [file DataSheet4.zip › Figure 2/Figure 2E-F/2E-F data.pdf]

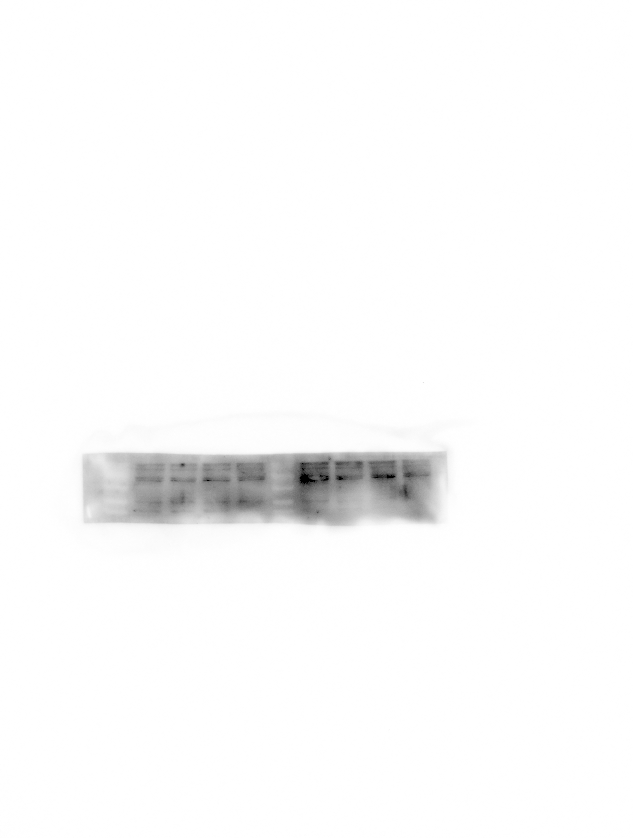

Supplement: Supplementary file 5 [file DataSheet10.zip › Figure 8/Figure 8F/KLF4-1-R-CTL.H2O2.H2O2+P.H2O2+P+K-72.55.40.33.25kd.tif]

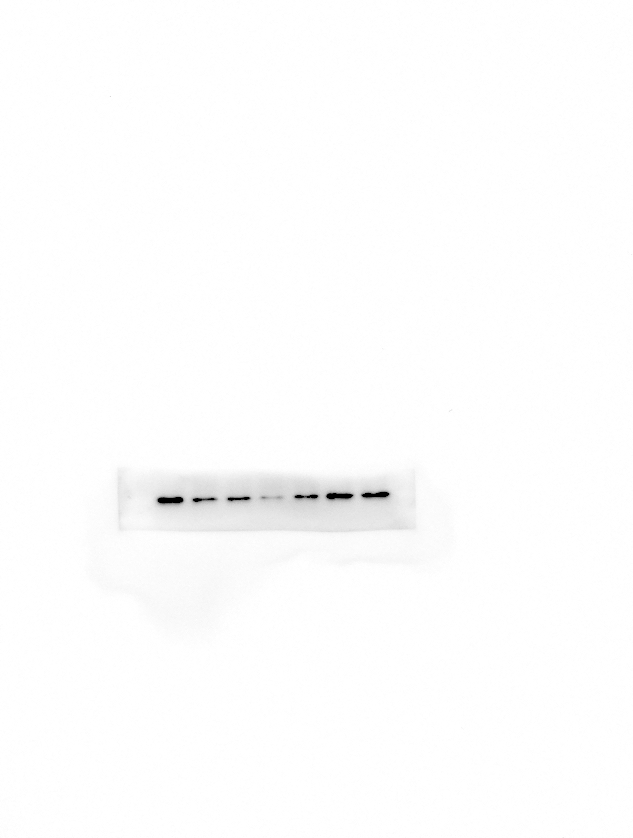

Supplement: Supplementary file 5 [file DataSheet10.zip › Figure 8/Figure 8F/MZB1-4-H+P+K.H+P.H.CTL.H.H+M.H+M+K-25.15.10kd.tif]

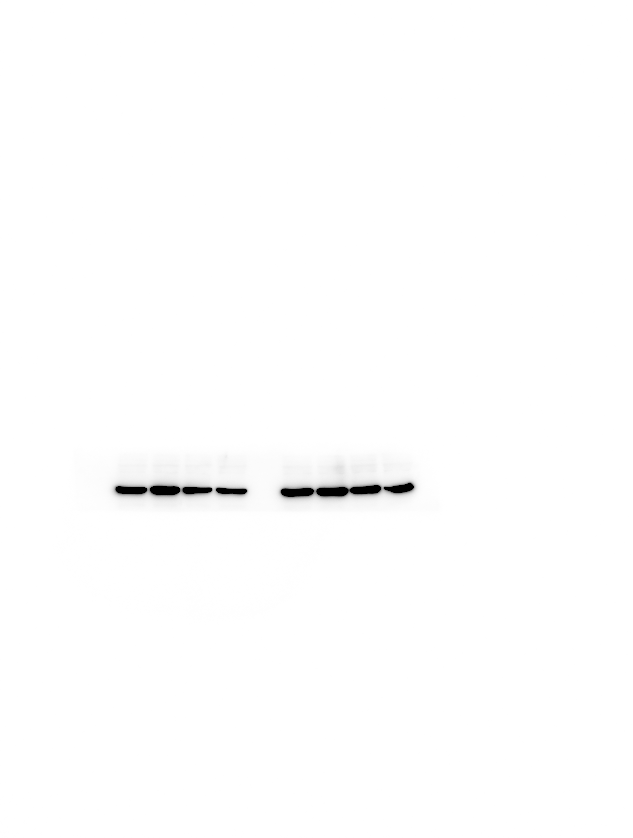

Supplement: Supplementary file 5 [file DataSheet10.zip › Figure 8/Figure 8F/GAPDH-2-CTL.H2O2.H2O2+P.H2O2+P+K-72.55.40.33.25kd.tif]

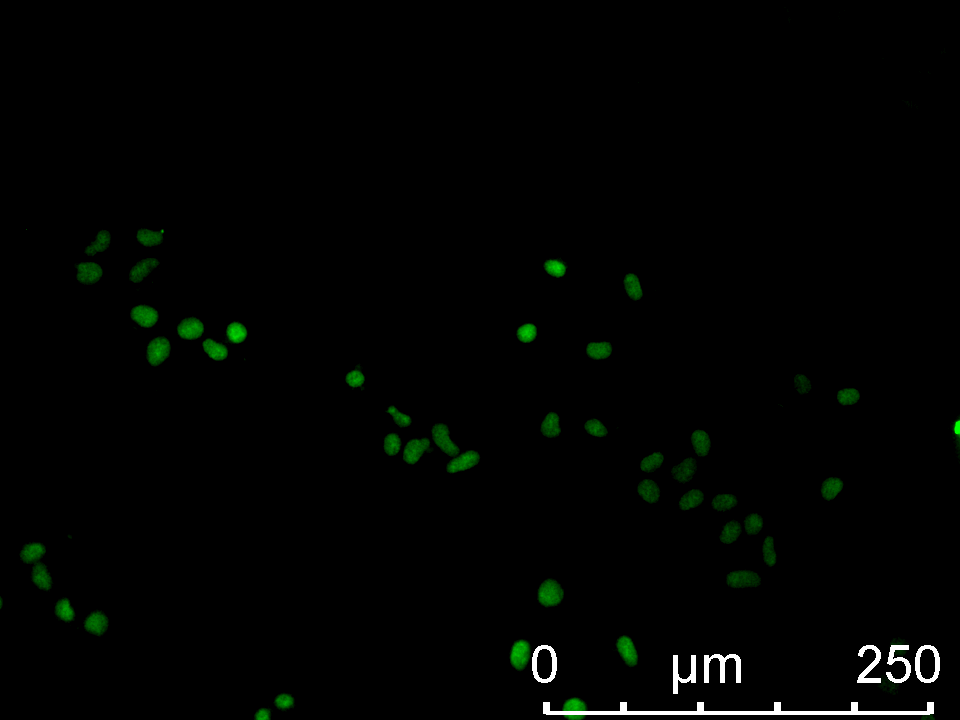

Supplement: Supplementary file 5 [file DataSheet10.zip › Figure 8/FIgure 8J/H+P_H+P-ROS-200-11-2.tif]

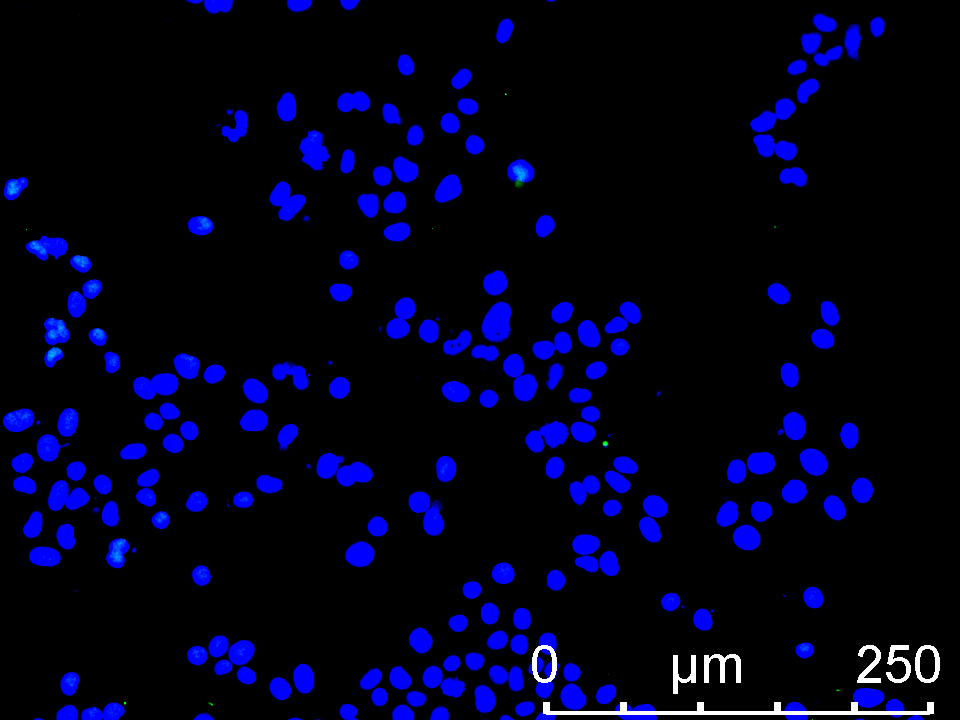

Supplement: Supplementary file 5 [file DataSheet10.zip › Figure 8/FIgure 8J/CTL_CTL-ROS-200-9.tif]

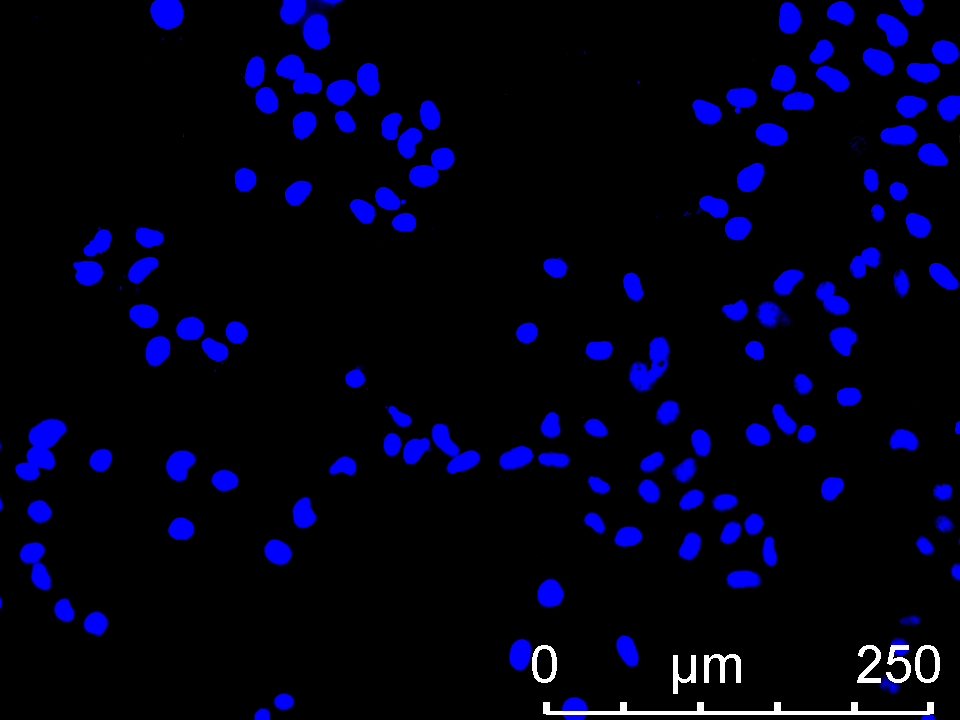

Supplement: Supplementary file 5 [file DataSheet10.zip › Figure 8/FIgure 8J/H+P_H+P-ROS-200-11-1.tif]

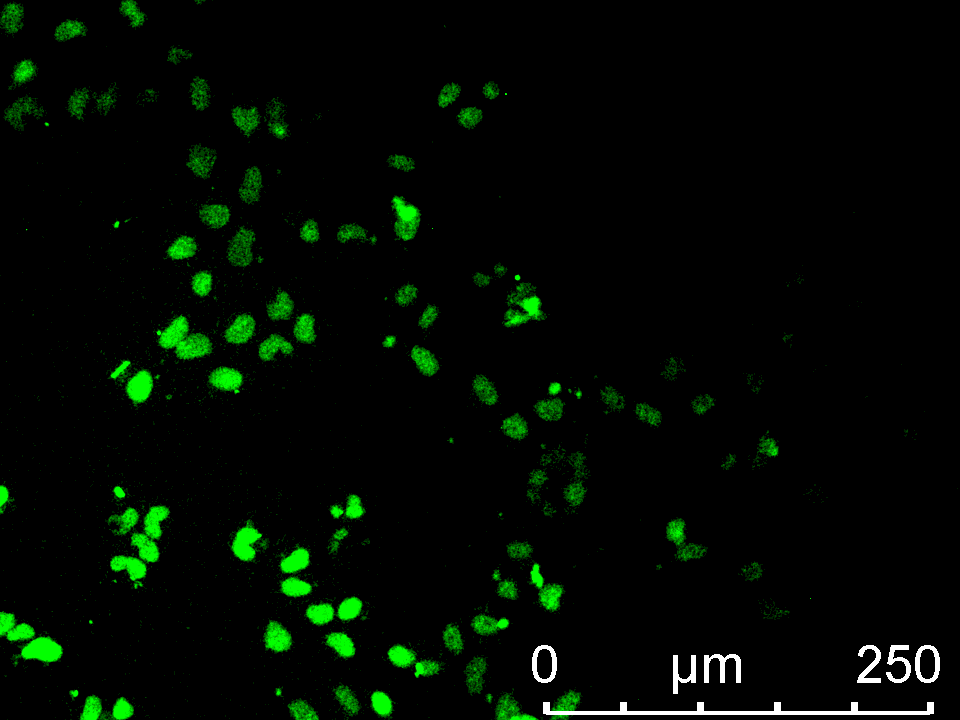

Supplement: Supplementary file 5 [file DataSheet10.zip › Figure 8/FIgure 8J/H+P+K_H+P+K-ROS-200-6-2.tif]

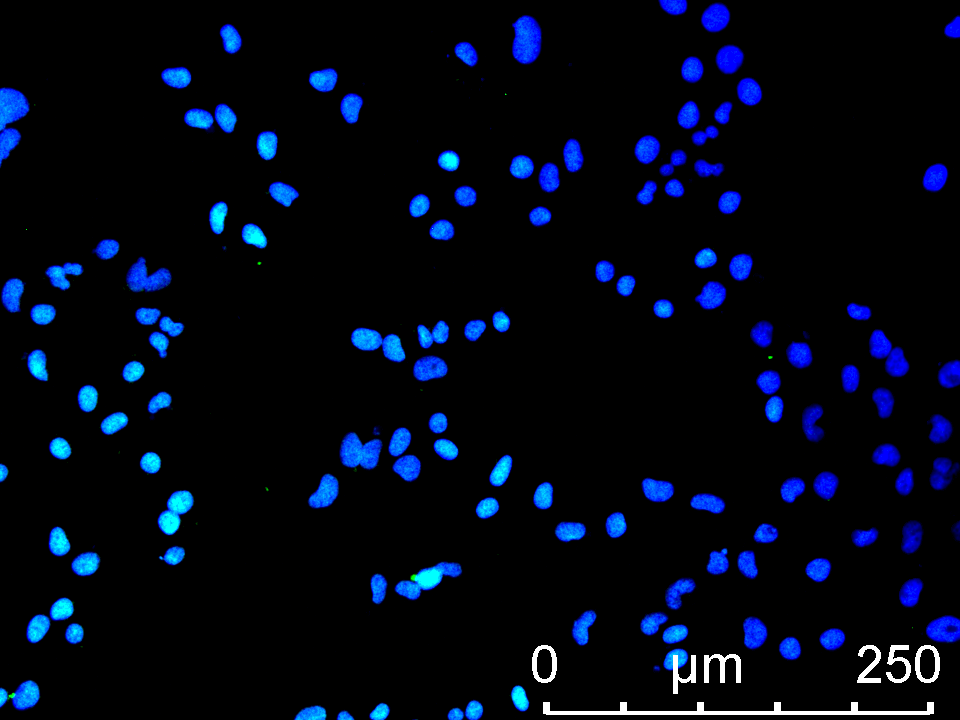

Supplement: Supplementary file 5 [file DataSheet10.zip › Figure 8/FIgure 8J/H2O2_H2O2-ROS-200-9.tif]

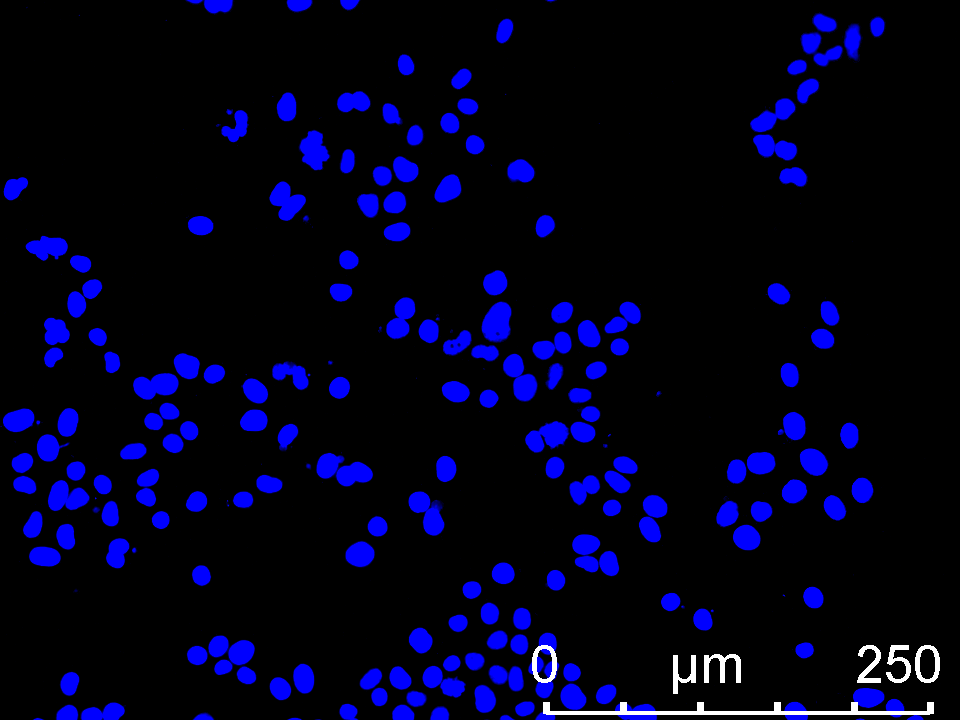

Supplement: Supplementary file 5 [file DataSheet10.zip › Figure 8/FIgure 8J/CTL_CTL-ROS-200-9-1.tif]

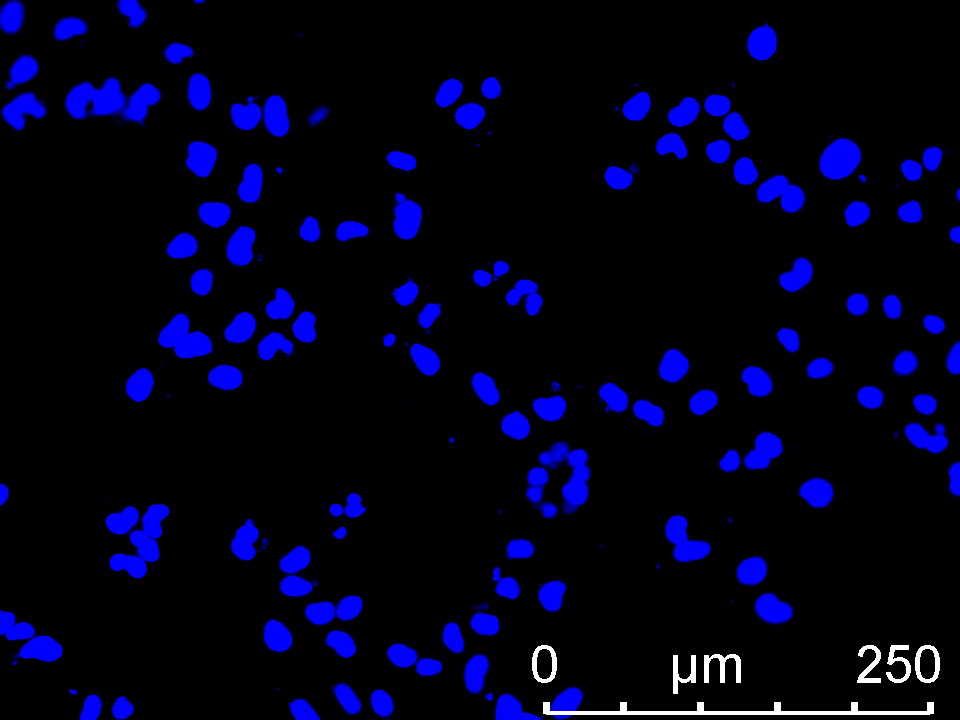

Supplement: Supplementary file 5 [file DataSheet10.zip › Figure 8/FIgure 8J/H+P+K_H+P+K-ROS-200-6-1.tif]

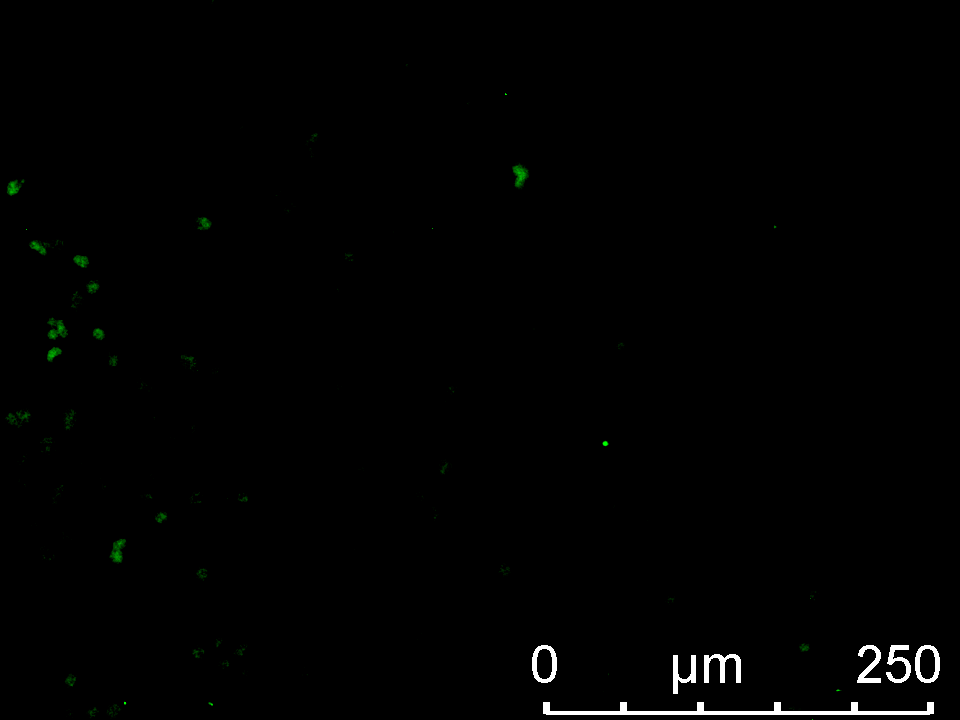

Supplement: Supplementary file 5 [file DataSheet10.zip › Figure 8/FIgure 8J/CTL_CTL-ROS-200-9-2.tif]

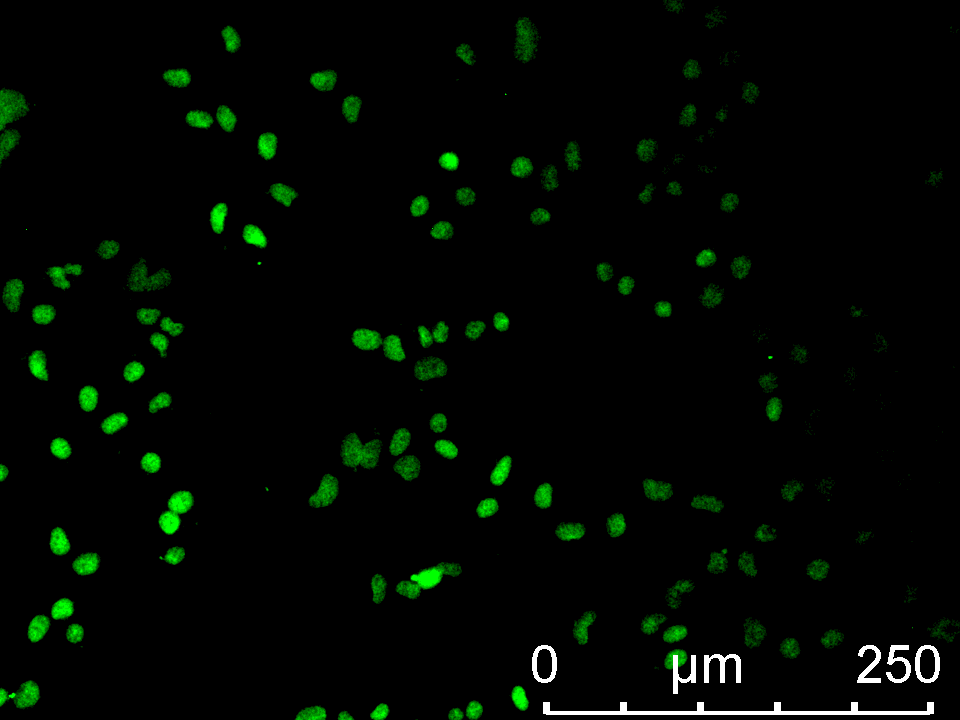

Supplement: Supplementary file 5 [file DataSheet10.zip › Figure 8/FIgure 8J/H2O2_H2O2-ROS-200-9-2.tif]

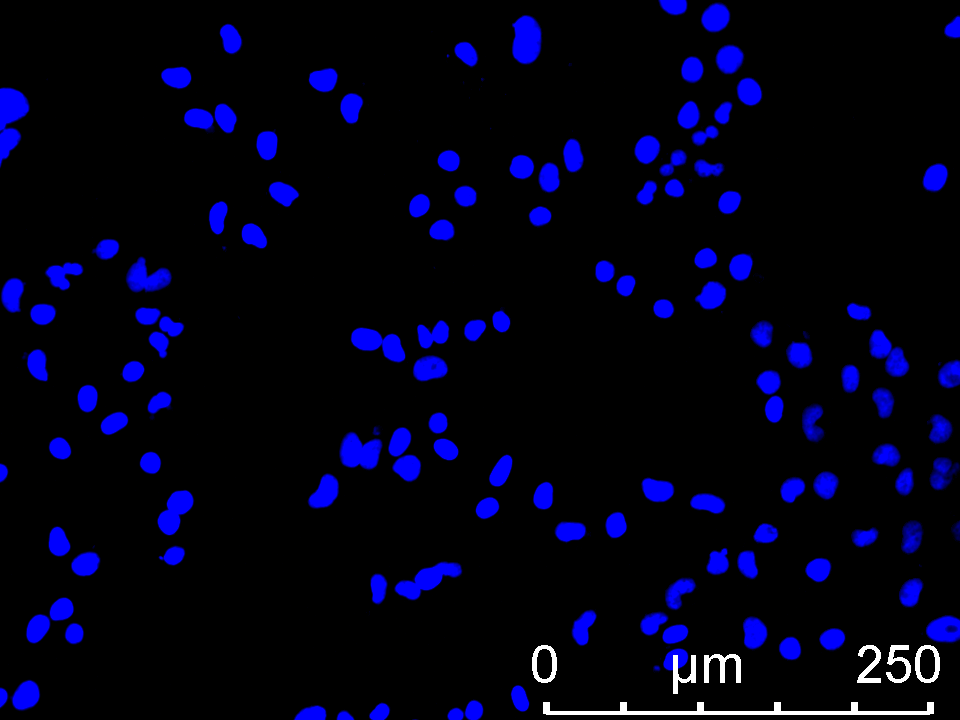

Supplement: Supplementary file 5 [file DataSheet10.zip › Figure 8/FIgure 8J/H2O2_H2O2-ROS-200-9-1.tif]

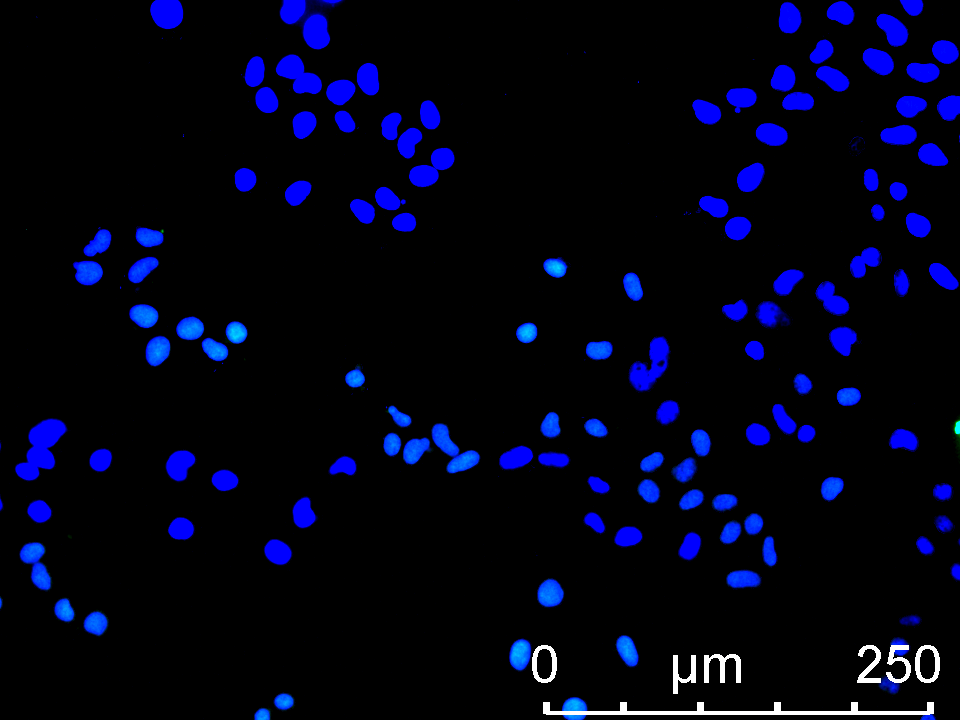

Supplement: Supplementary file 5 [file DataSheet10.zip › Figure 8/FIgure 8J/H+P_H+P-ROS-200-11.tif]

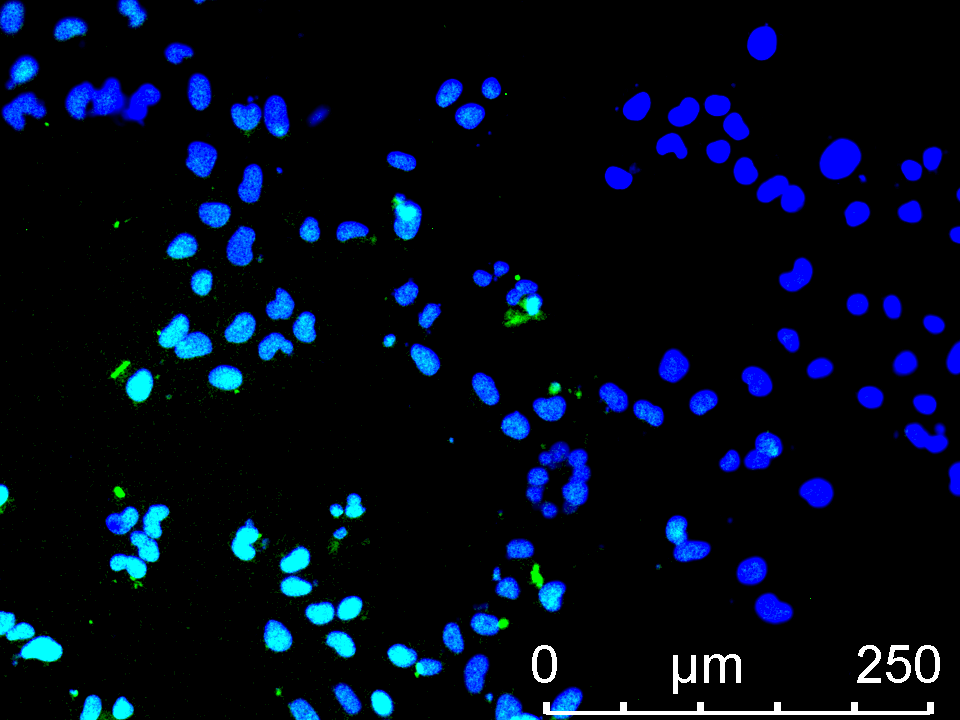

Supplement: Supplementary file 5 [file DataSheet10.zip › Figure 8/FIgure 8J/H+P+K_H+P+K-ROS-200-6.tif]

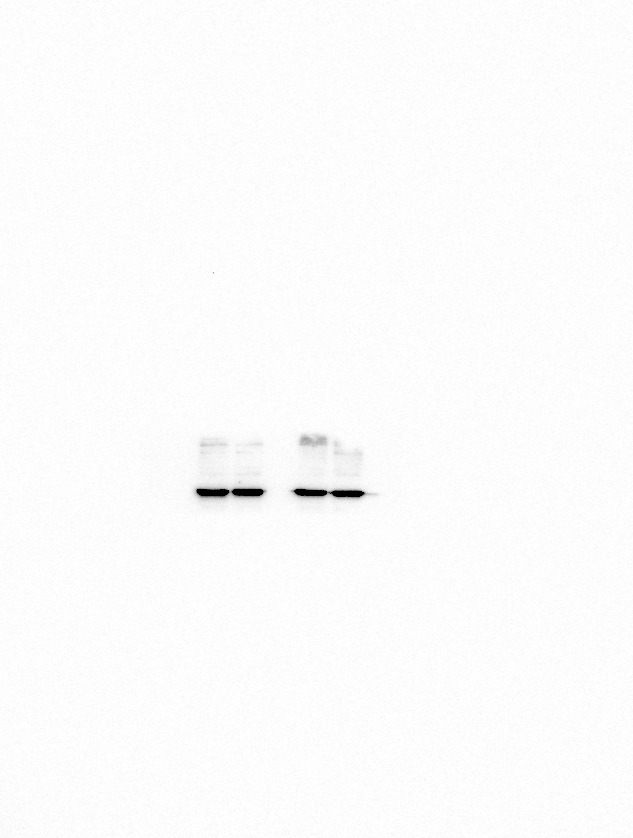

Supplement: Supplementary file 5 [file DataSheet10.zip › Figure 8/Figure 8D/GAPDH-1-CTL.KLF4μèæσê╢σëé-180.130.95.72.55.40.33.25kd.tif]

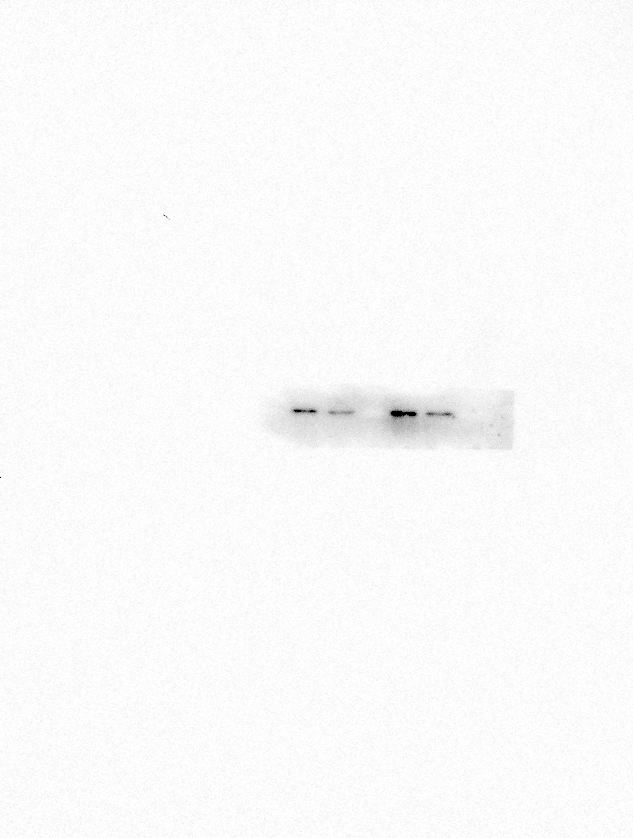

Supplement: Supplementary file 5 [file DataSheet10.zip › Figure 8/Figure 8D/MZB1-1-CTL.KLF4μèæσê╢σëé-25.15.10kd.tiff]

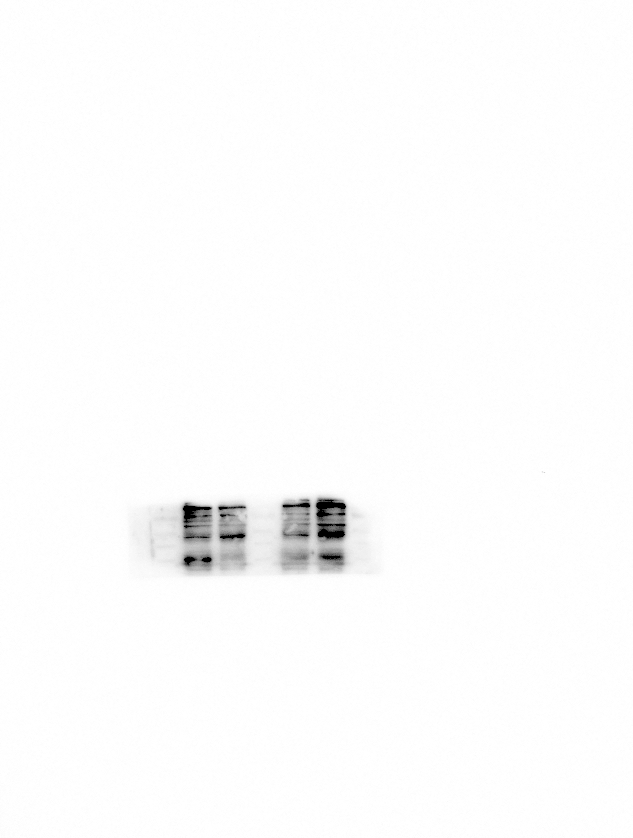

Supplement: Supplementary file 5 [file DataSheet10.zip › Figure 8/Figure 8D/KLF4-1-CTL.KLF4μèæσê╢σëé-180.130.95.72.55.40.33.25kd.tif]

Figure 8C

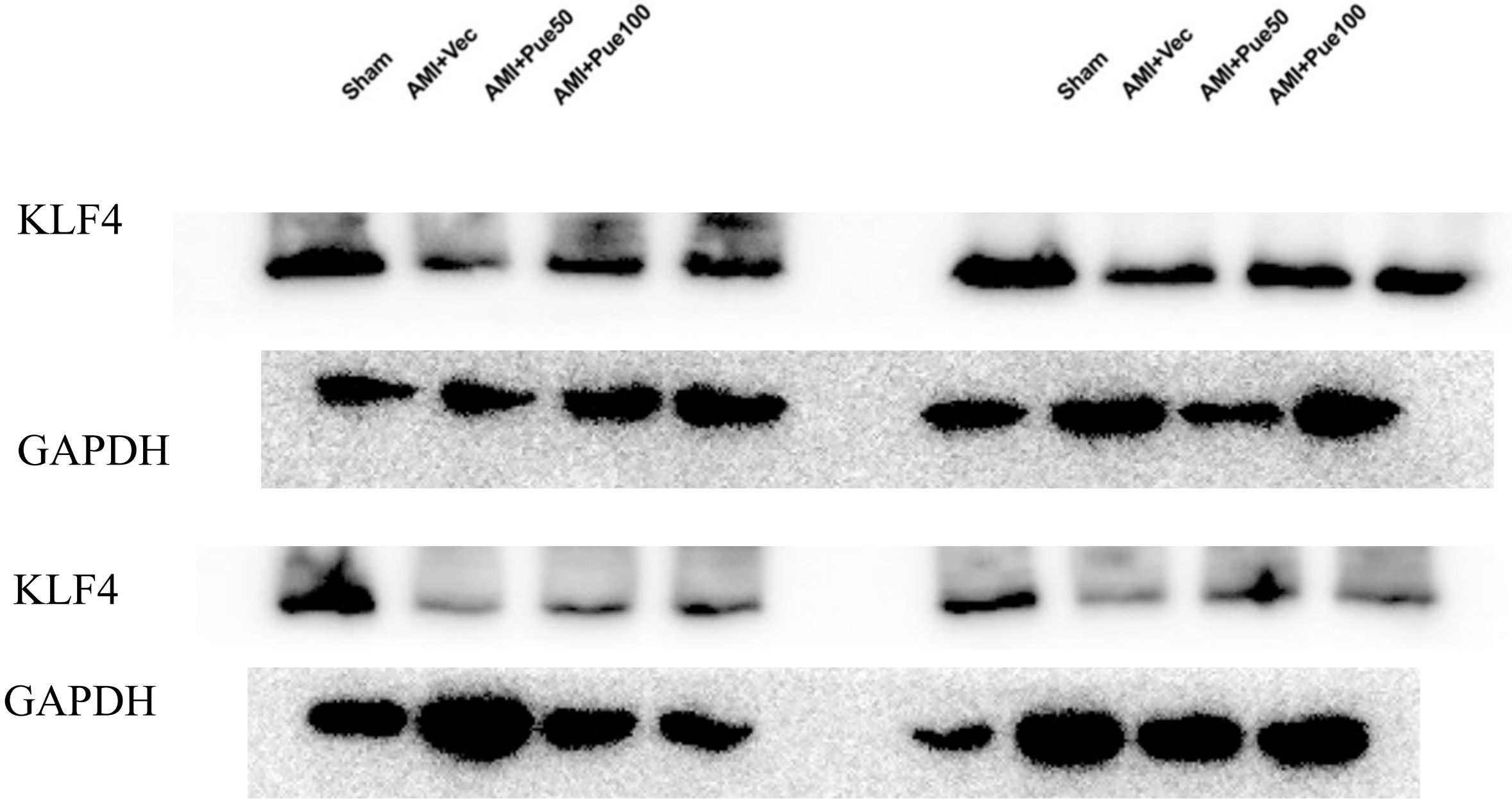

| KLF4 | Sham | AMI+<br>Vec | AMI+<br>Pue50 | AMI+<br>Pue100 |
|------|------|-------------|---------------|----------------|
|      | 1    | 0.31703678  | 0.61461504    | 0.75870283     |
|      | 1    | 0.17127085  | 0.41709496    | 0.56252141     |
|      | 1    | 0.1774114   | 0.39525077    | 0.43286154     |
|      | 1    | 0.35331798  | 0.60196979    | 0.62772969     |

Supplement: Supplementary file 5 [file DataSheet10.zip › Figure 8/Figure 8C/8C.pdf]

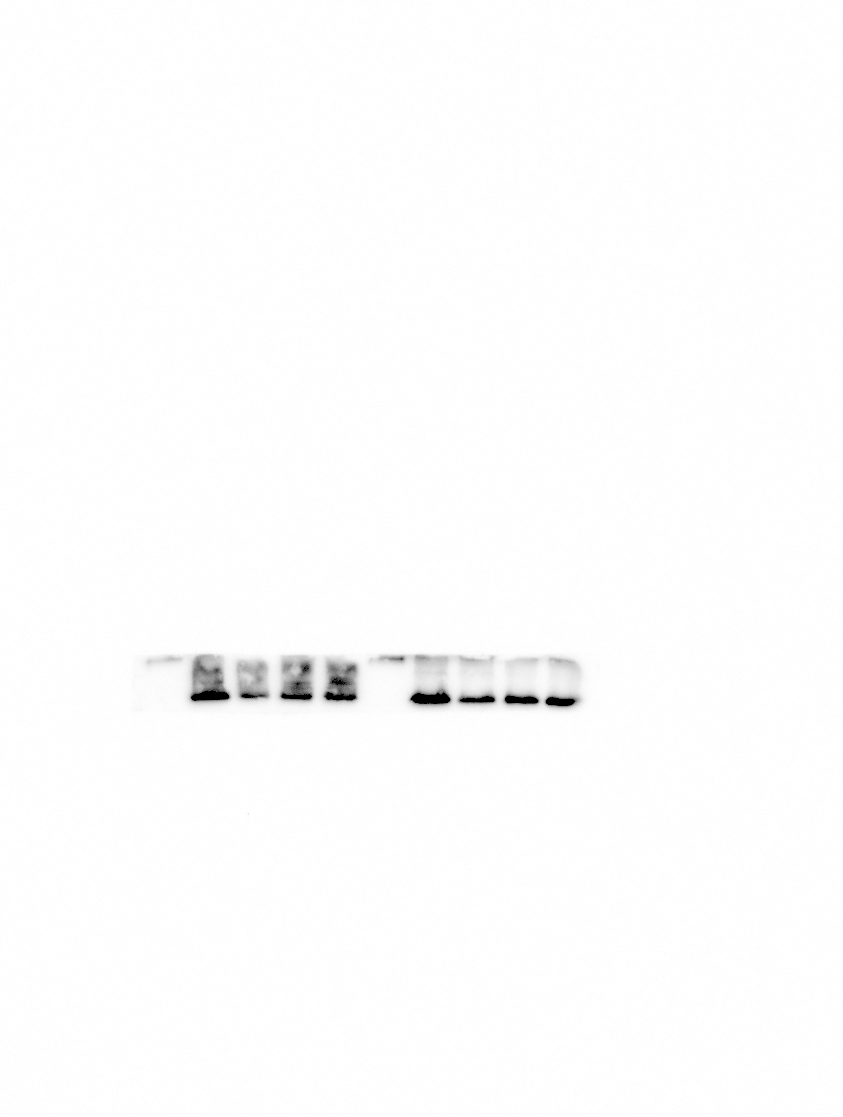

Supplement: Supplementary file 5 [file DataSheet10.zip › Figure 8/Figure 8B/KLF4-1-WT.AMI.P50.P100-180.130.95.72.55kd.tif]

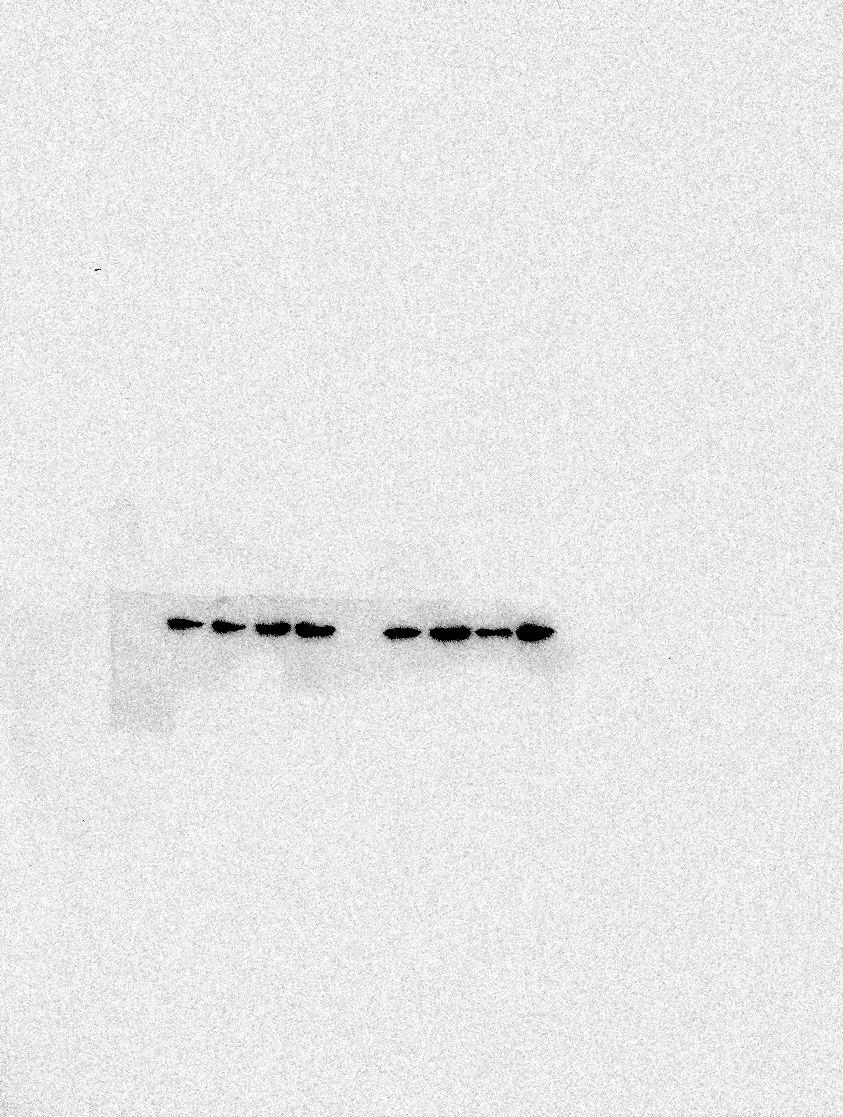

Supplement: Supplementary file 5 [file DataSheet10.zip › Figure 8/Figure 8B/GAPDH-1-WT.AMI.P50.P100-55.40.33.25.15.10kd.tif]

Figure 8E

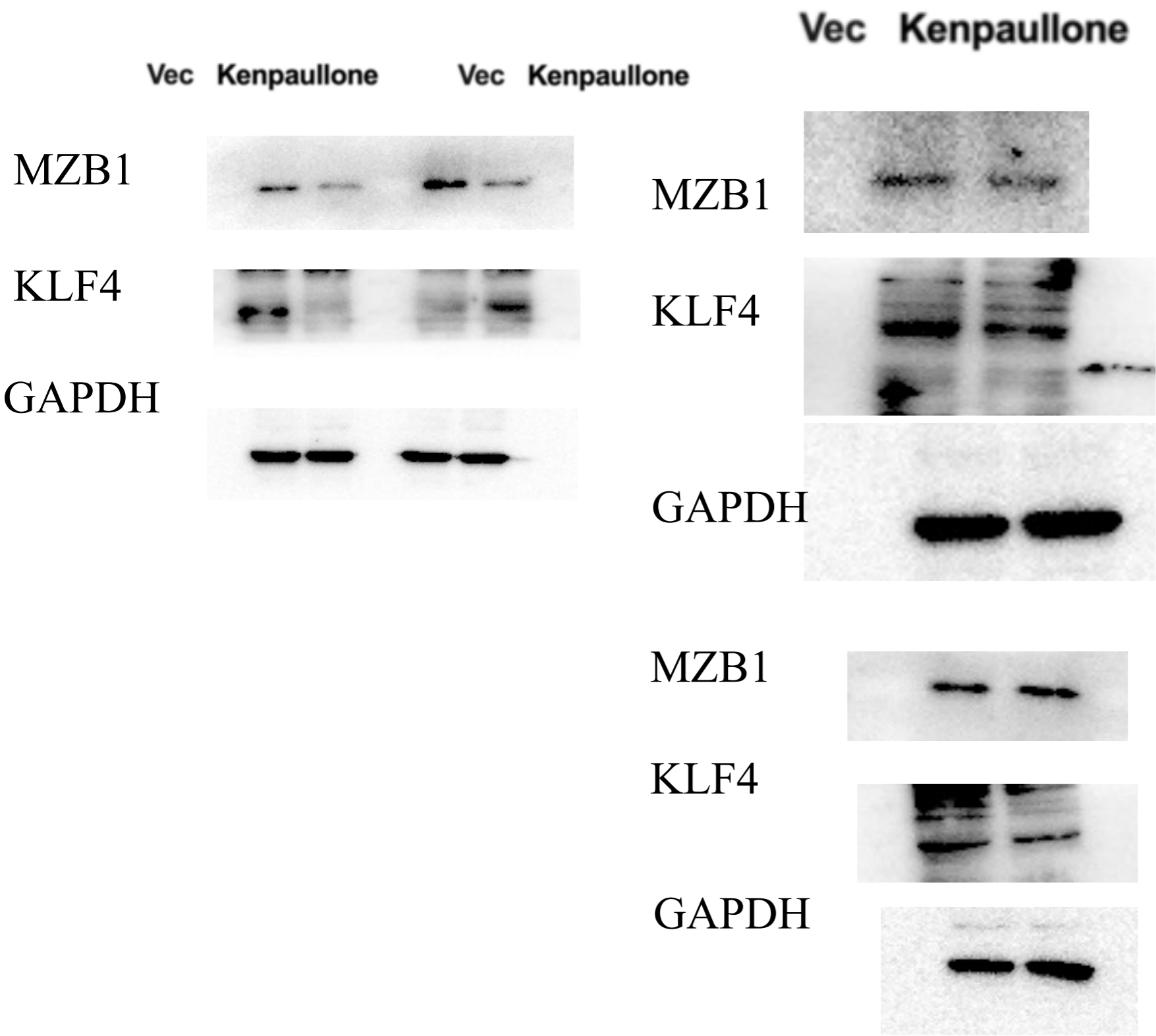

|      | Vec |   |   |  | Kenpaullone |       |       |  |
|------|-----|---|---|--|-------------|-------|-------|--|
| KLF4 | 1   | 1 | 1 |  | 0.297       | 0.482 | 0.561 |  |
| Mzb1 | 1   | 1 | 1 |  | 0.525       | 0.361 | 0.568 |  |

Supplement: Supplementary file 5 [file DataSheet10.zip › Figure 8/Figure 8E/8E.pdf]

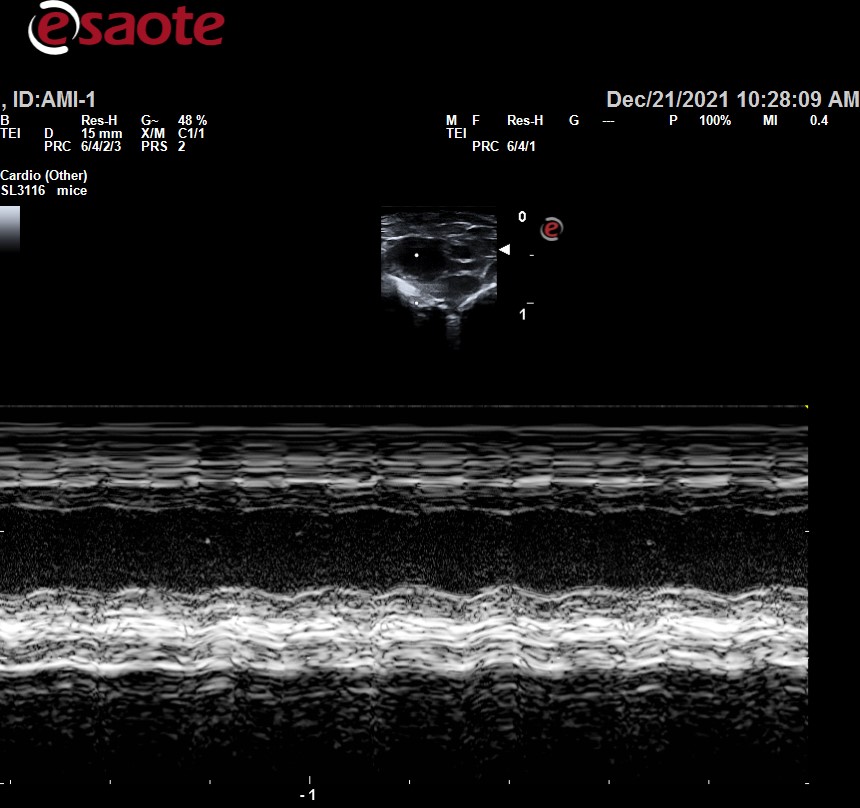

Supplement: Supplementary file 7 [file DataSheet2.zip › Figure 1A/AMI-1-37.2.jpeg]

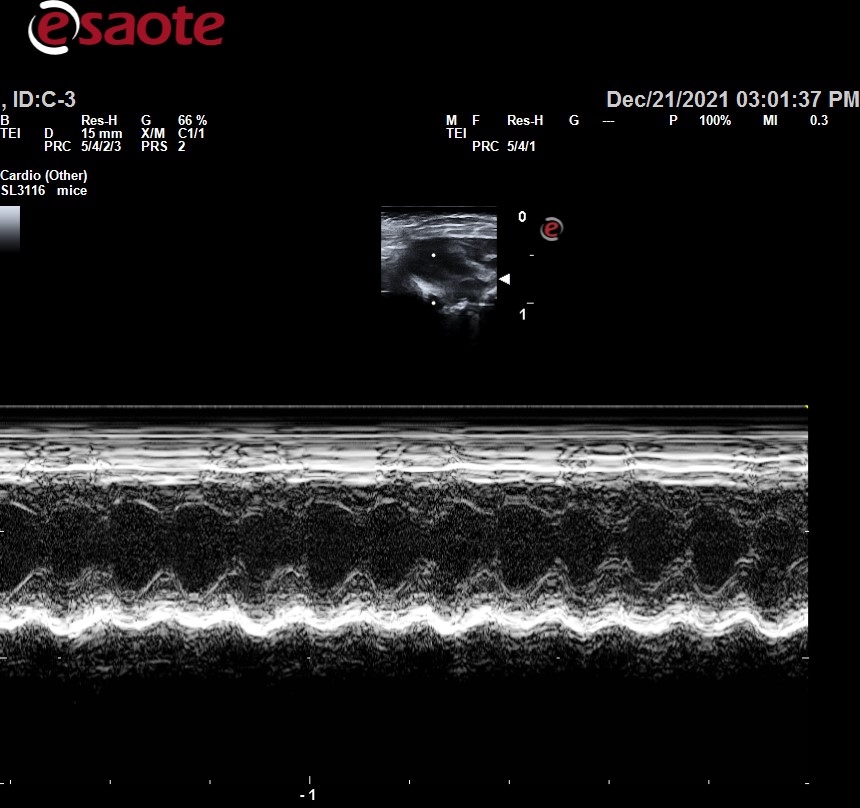

Supplement: Supplementary file 7 [file DataSheet2.zip › Figure 1A/Sham-3-37.3.jpeg]

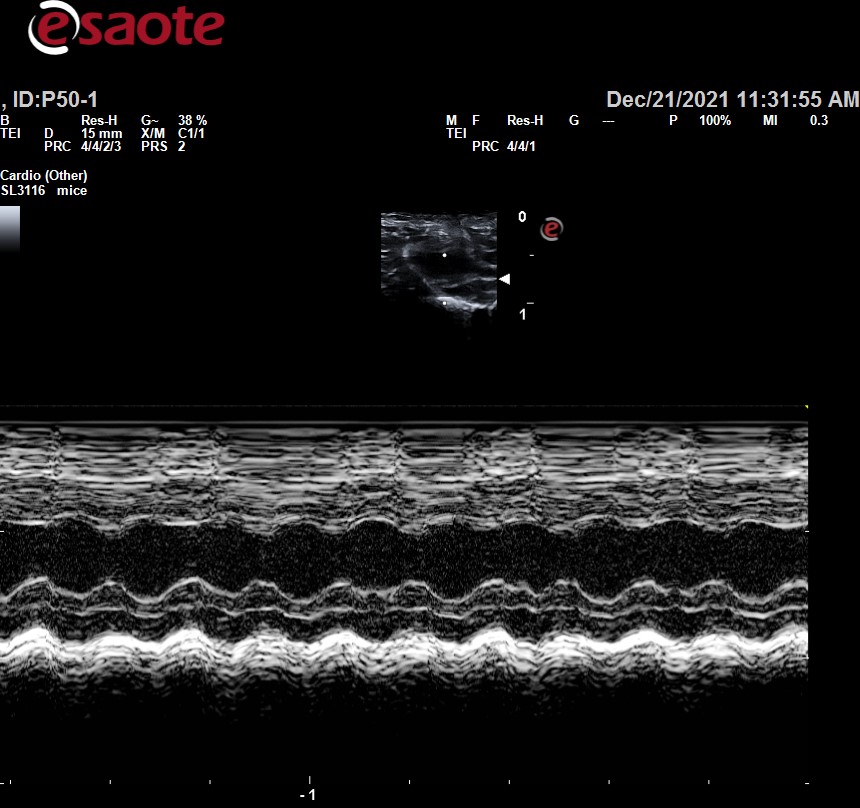

Supplement: Supplementary file 7 [file DataSheet2.zip › Figure 1A/AMI+P50-1-37.5.jpeg]

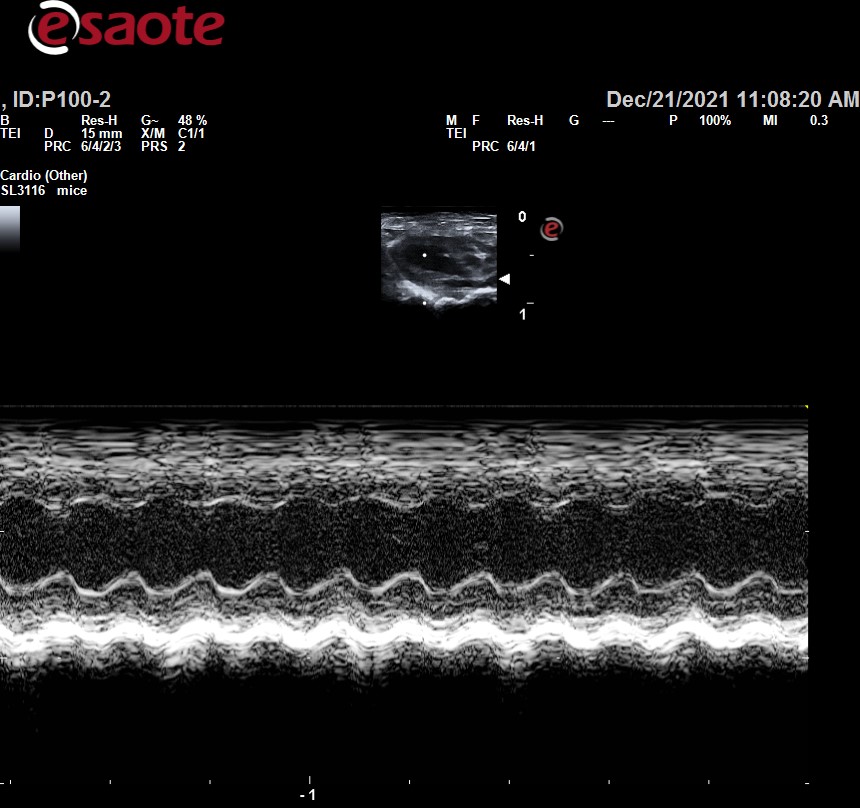

Supplement: Supplementary file 7 [file DataSheet2.zip › Figure 1A/AMI+P100-2-37.4.jpeg]

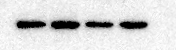

Supplement: Supplementary file 8 [file DataSheet5.zip › Figure 3/Figure 3C-D/gapdh-2-straight.tif]

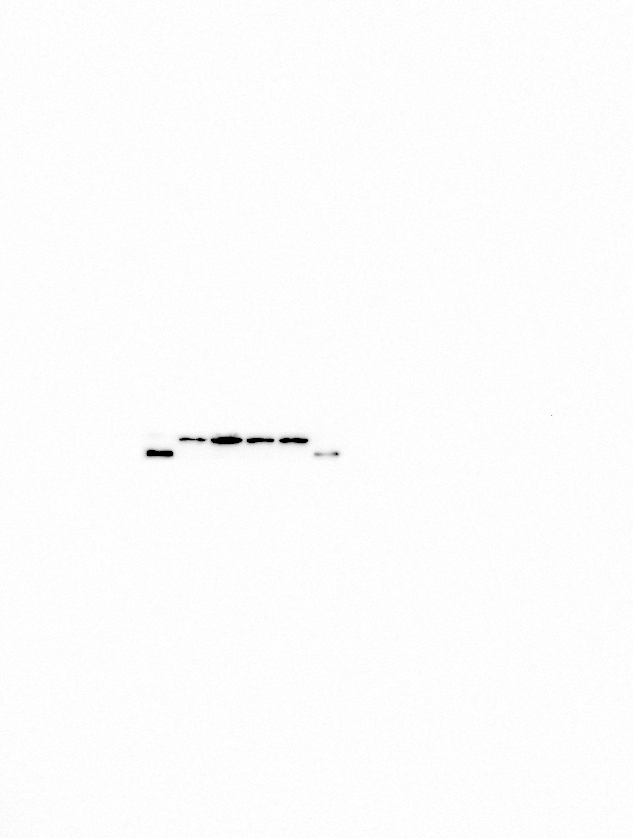

Supplement: Supplementary file 8 [file DataSheet5.zip › Figure 3/Figure 3C-D/cleaved-cas3-2.tif]

Figure 3C-D

Sham    AMI+    AMI+    AMI+  
          Vec    Pue50 Pue100

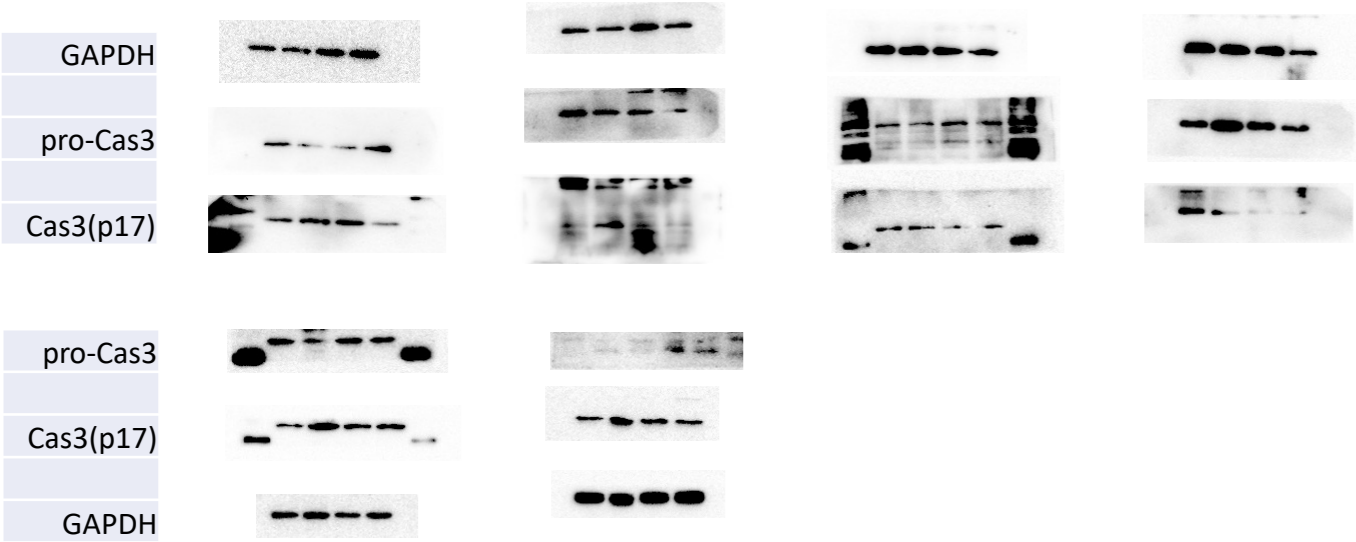

| Cleaved-Caspase3/PR O-cas | Sham | AMI+ Vec    | AMI+ Pue50  | AMI+ Pue100 |
|---------------------------|------|-------------|-------------|-------------|
|                           | 1    | 3.875438444 | 3.076838968 | 0.413647076 |
|                           | 1    | 2.713051184 | 1.789158518 | 1.465754475 |
|                           | 1    | 1.881811974 | 0.299850371 | 0.357137301 |
|                           | 1    | 1.659026372 | 0.268381632 | 0.20844608  |
|                           | 1    | 1.637035608 | 0.251626675 | 0.592204456 |
|                           | 1    | 1.679376486 | 0.640309482 | 0.454196355 |

Supplement: Supplementary file 8 [file DataSheet5.zip › Figure 3/Figure 3C-D/3C-D.pdf]

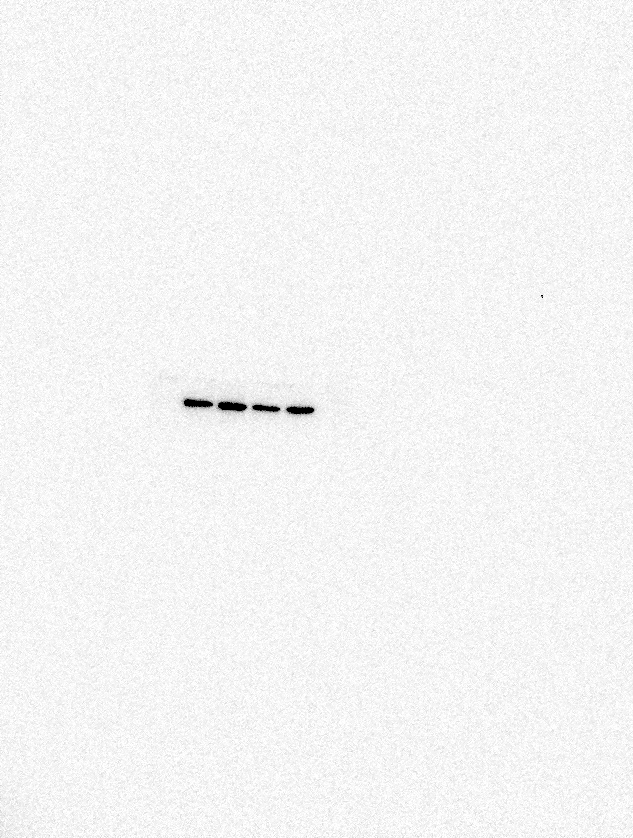

Supplement: Supplementary file 8 [file DataSheet5.zip › Figure 3/Figure 3C-D/gapdh-2.tif]

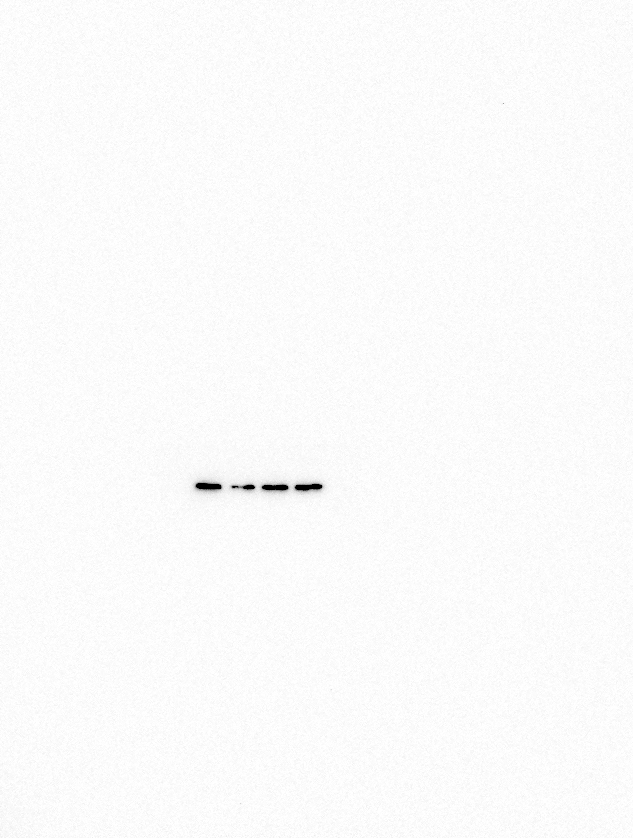

Supplement: Supplementary file 8 [file DataSheet5.zip › Figure 3/Figure 3E-F/mzb1-4.tif]

Figure 3E-F

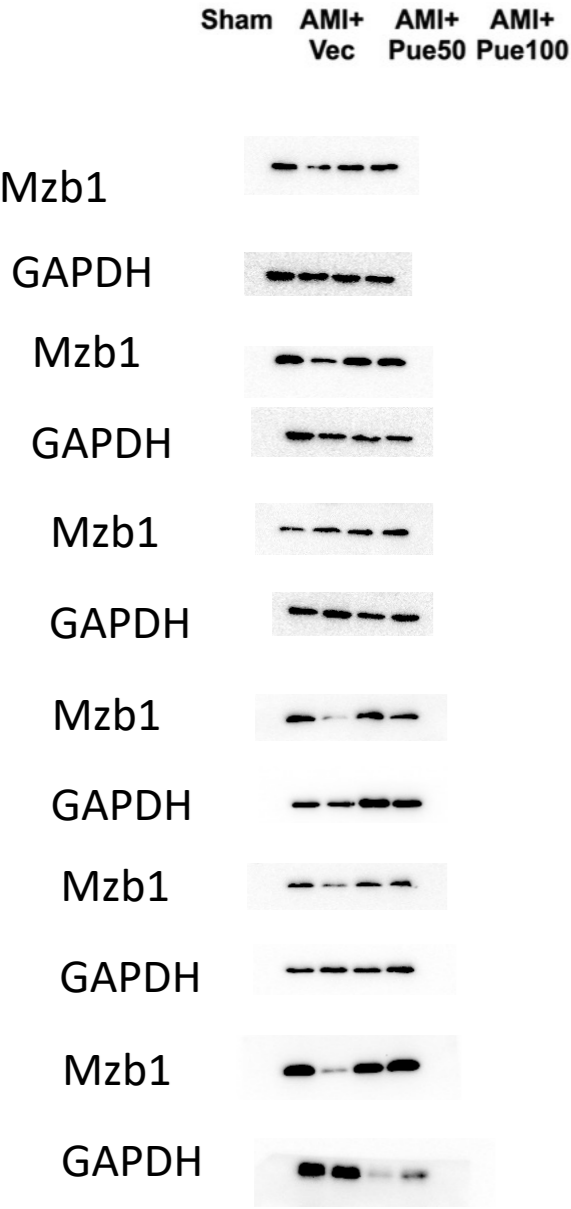

|      |      | AMI+<br>Vec | AMI+<br>Pue50 | AMI+<br>Pue100 |
|------|------|-------------|---------------|----------------|
| Mzb1 | Sham |             |               |                |
|      | 1    | 0.6166592   | 1.0637564     | 1.20744111     |
|      | 1    | 0.69080745  | 1.26824267    | 1.3687133      |
|      | 1    | 0.24796304  | 0.55859312    | 0.69016776     |
|      | 1    | 0.18220591  | 1.2577186     | 1.34979709     |
|      | 1    | 0.4449165   | 0.59855075    | 0.69507905     |
|      | 1    | 0.35825965  | 0.57394496    | 1.00078427     |

Supplement: Supplementary file 8 [file DataSheet5.zip › Figure 3/Figure 3E-F/3E-F.pdf]

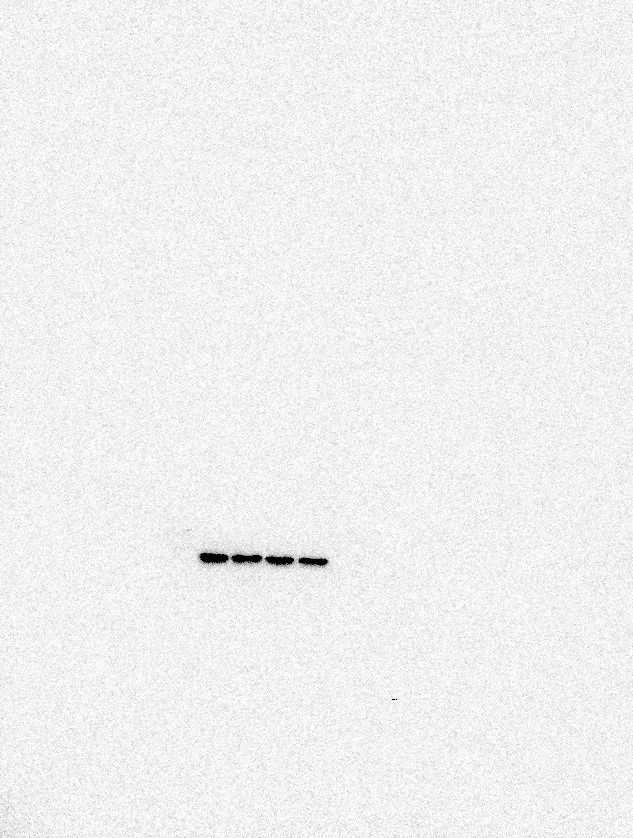

Supplement: Supplementary file 8 [file DataSheet5.zip › Figure 3/Figure 3E-F/gapdh-4.tif]

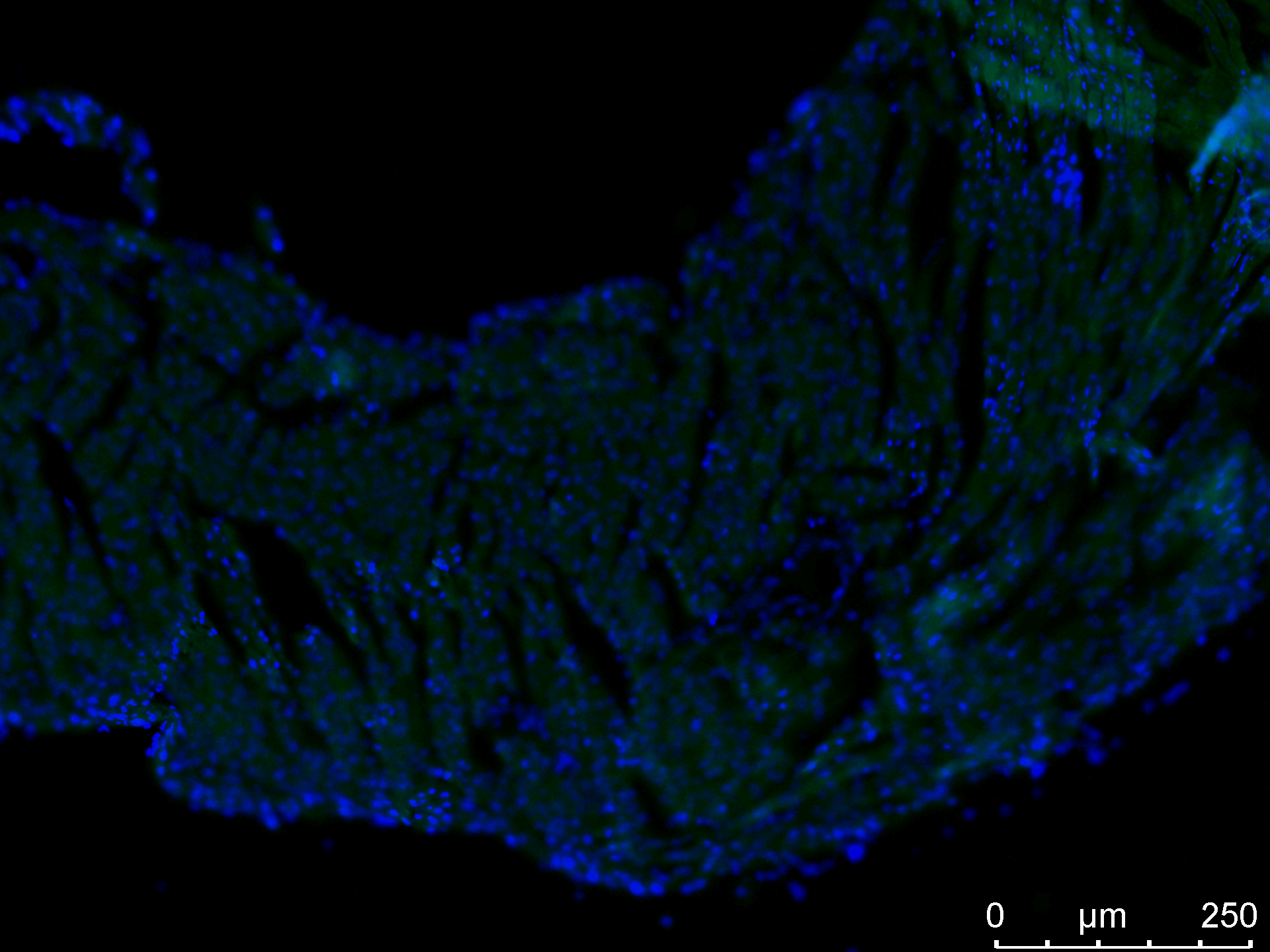

Supplement: Supplementary file 8 [file DataSheet5.zip › Figure 3/Figure 3A/original image/Sham.tif]

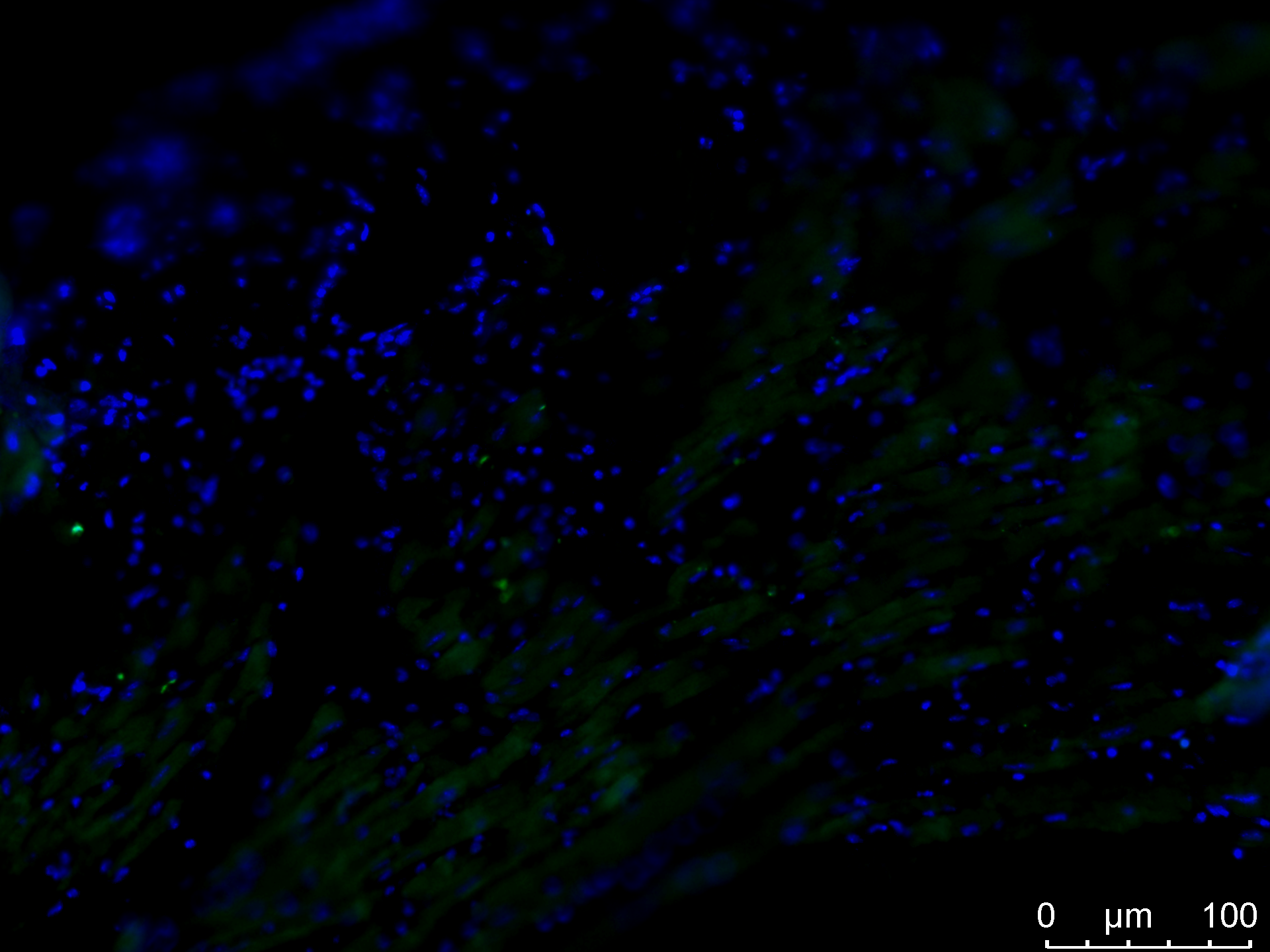

Supplement: Supplementary file 8 [file DataSheet5.zip › Figure 3/Figure 3A/original image/P100.tif]

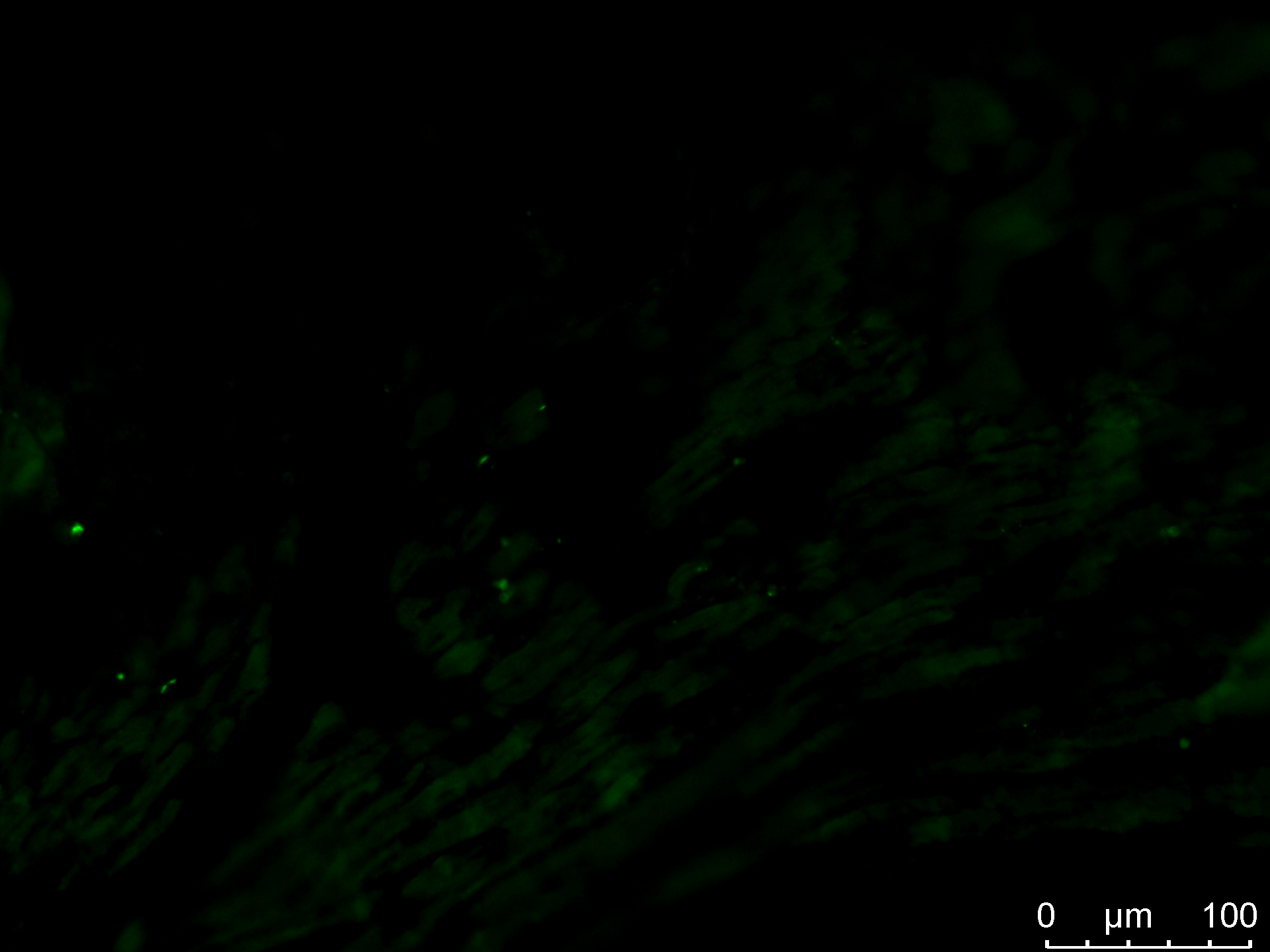

Supplement: Supplementary file 8 [file DataSheet5.zip › Figure 3/Figure 3A/original image/P100-G11.tif]

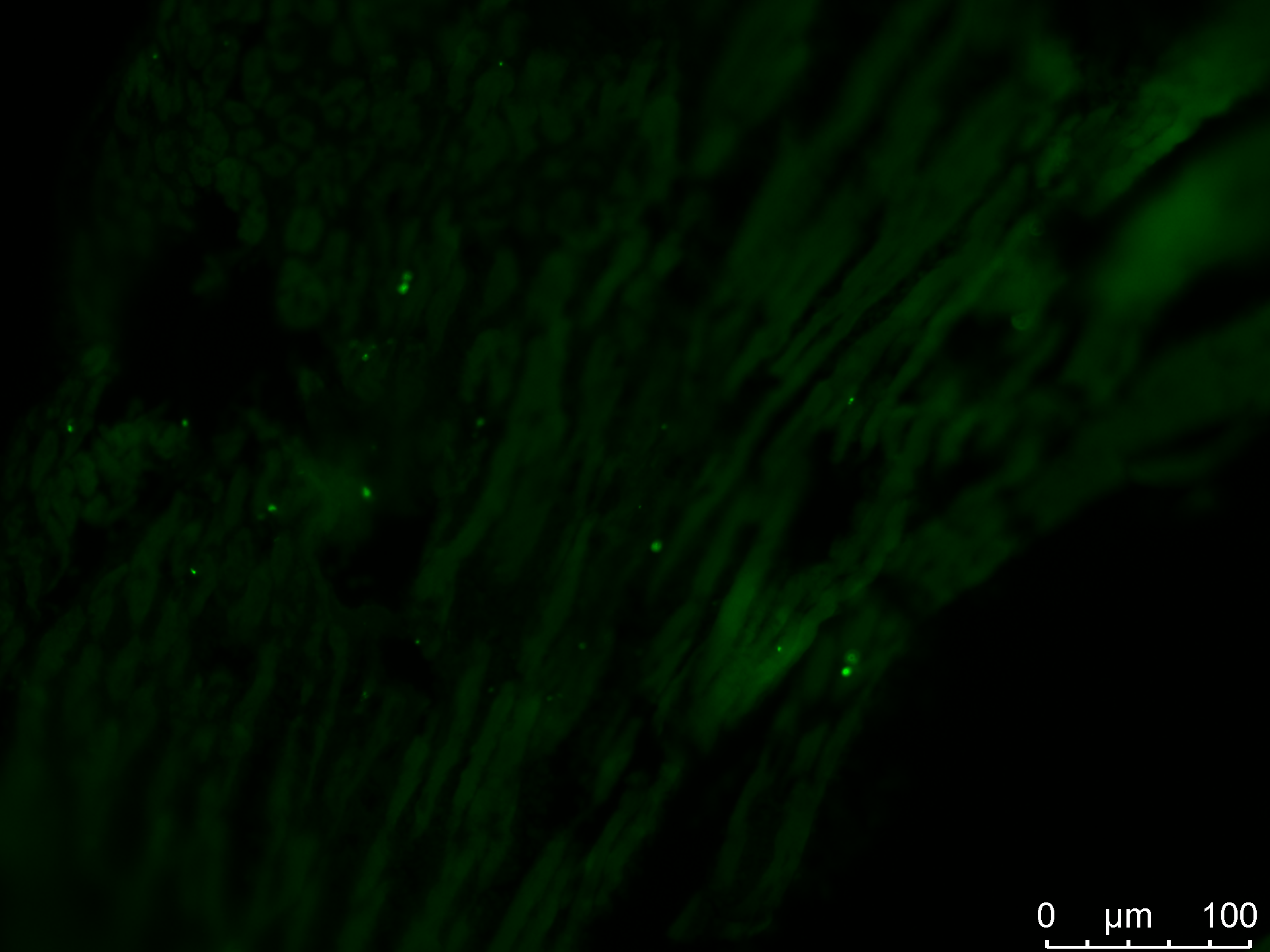

Supplement: Supplementary file 8 [file DataSheet5.zip › Figure 3/Figure 3A/original image/p50-G12.tif]

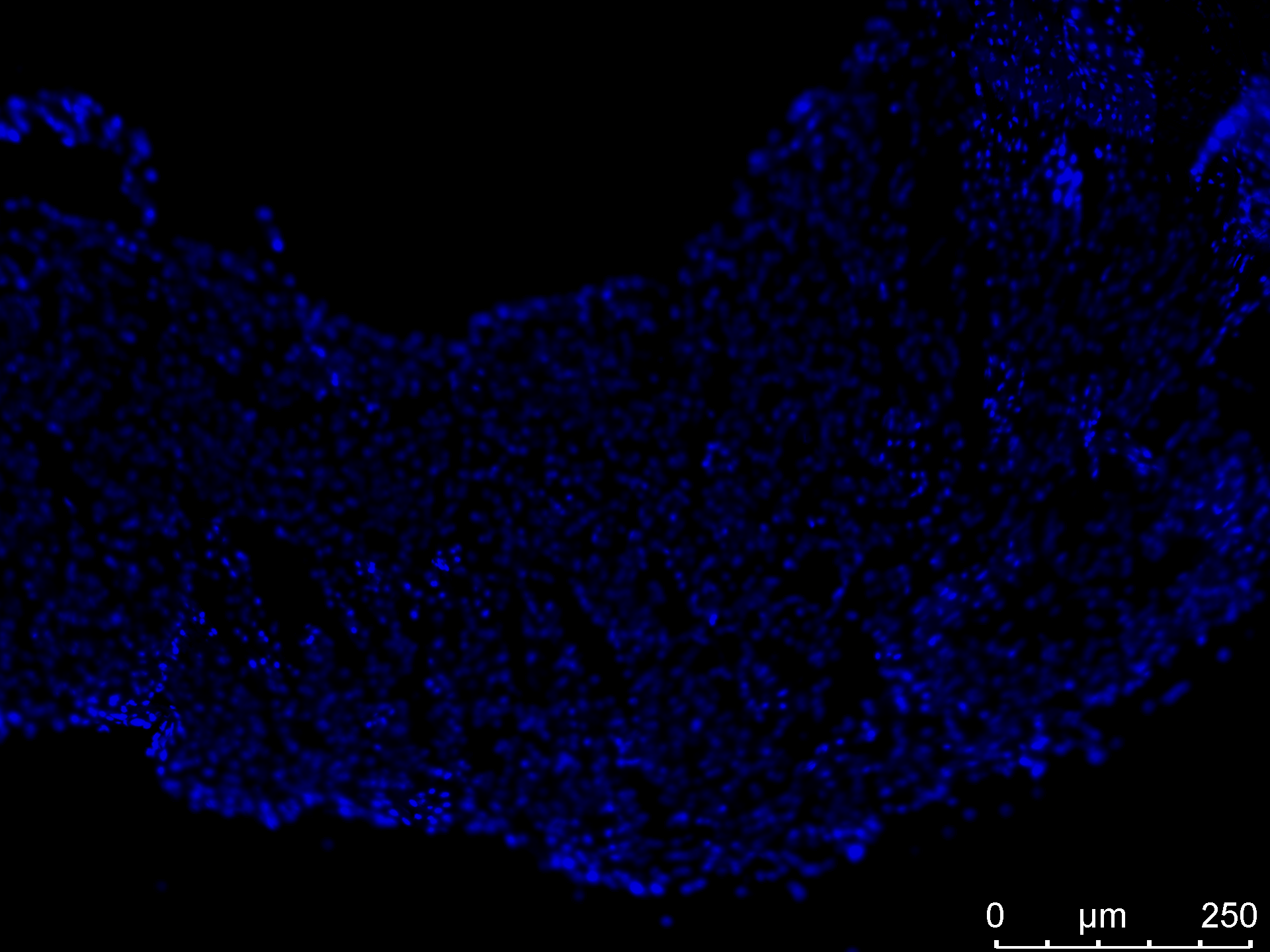

Supplement: Supplementary file 8 [file DataSheet5.zip › Figure 3/Figure 3A/original image/Sham-D1.tif]

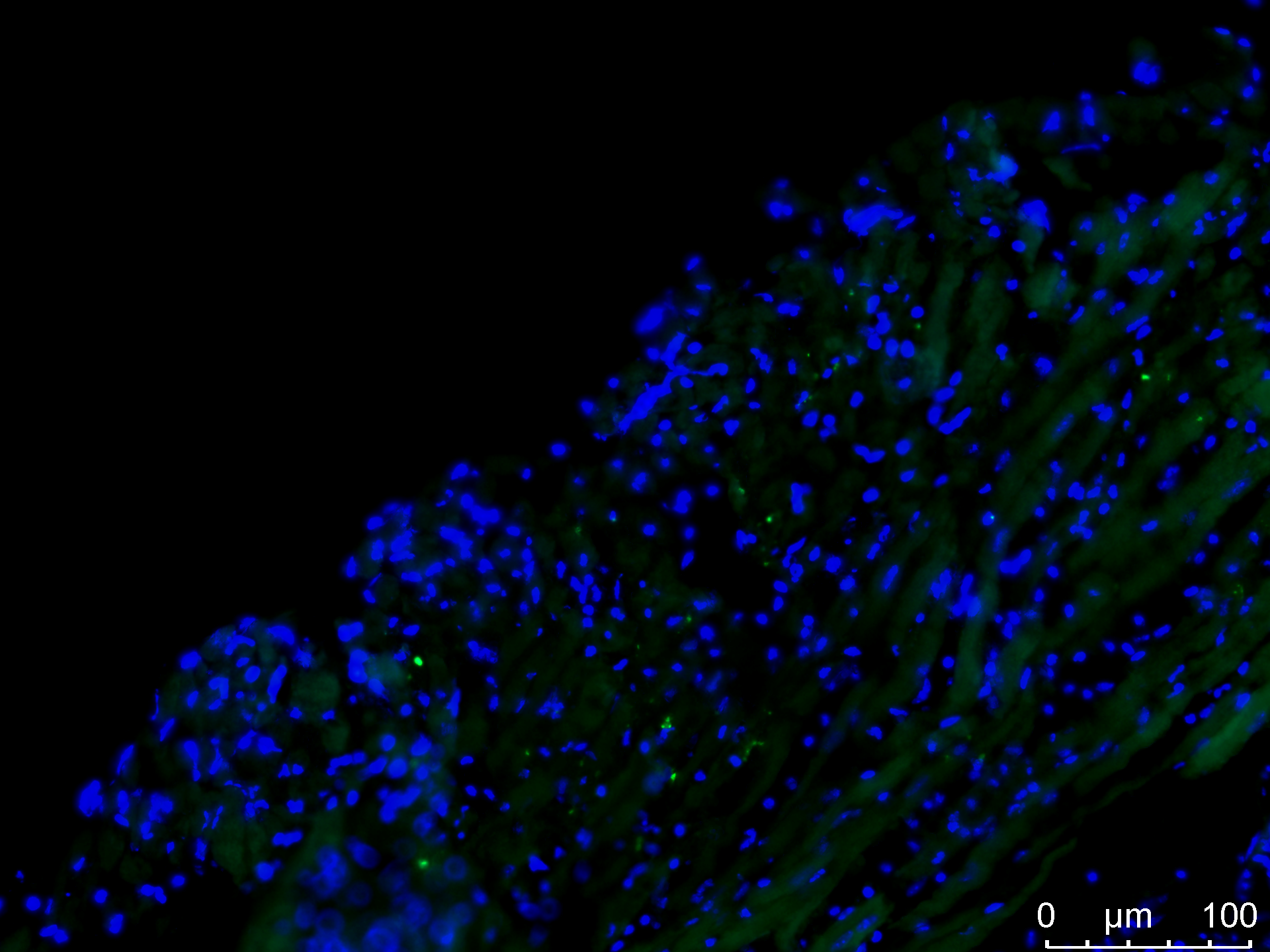

Supplement: Supplementary file 8 [file DataSheet5.zip › Figure 3/Figure 3A/original image/AMI.tif]

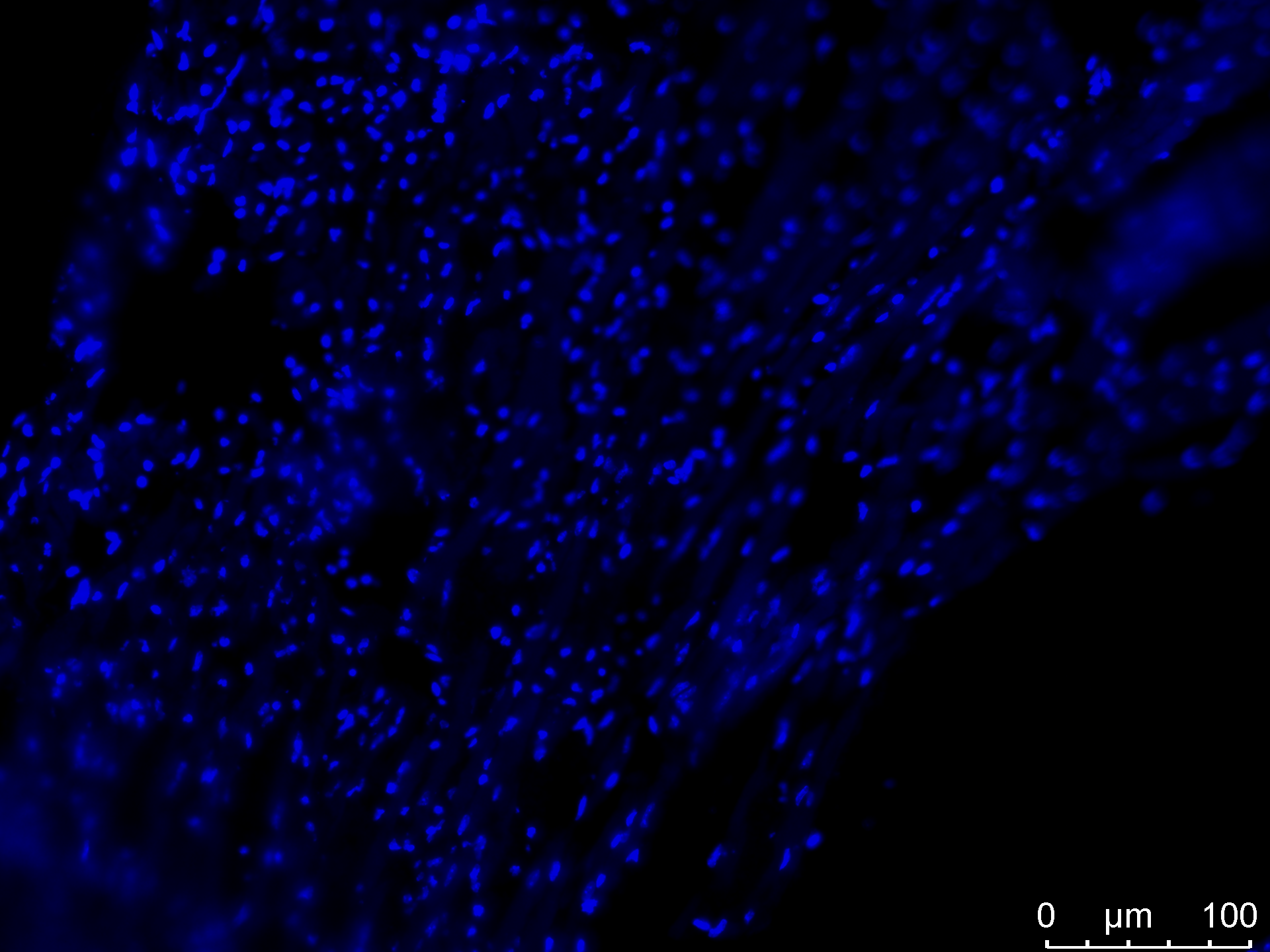

Supplement: Supplementary file 8 [file DataSheet5.zip › Figure 3/Figure 3A/original image/p50-D12.tif]

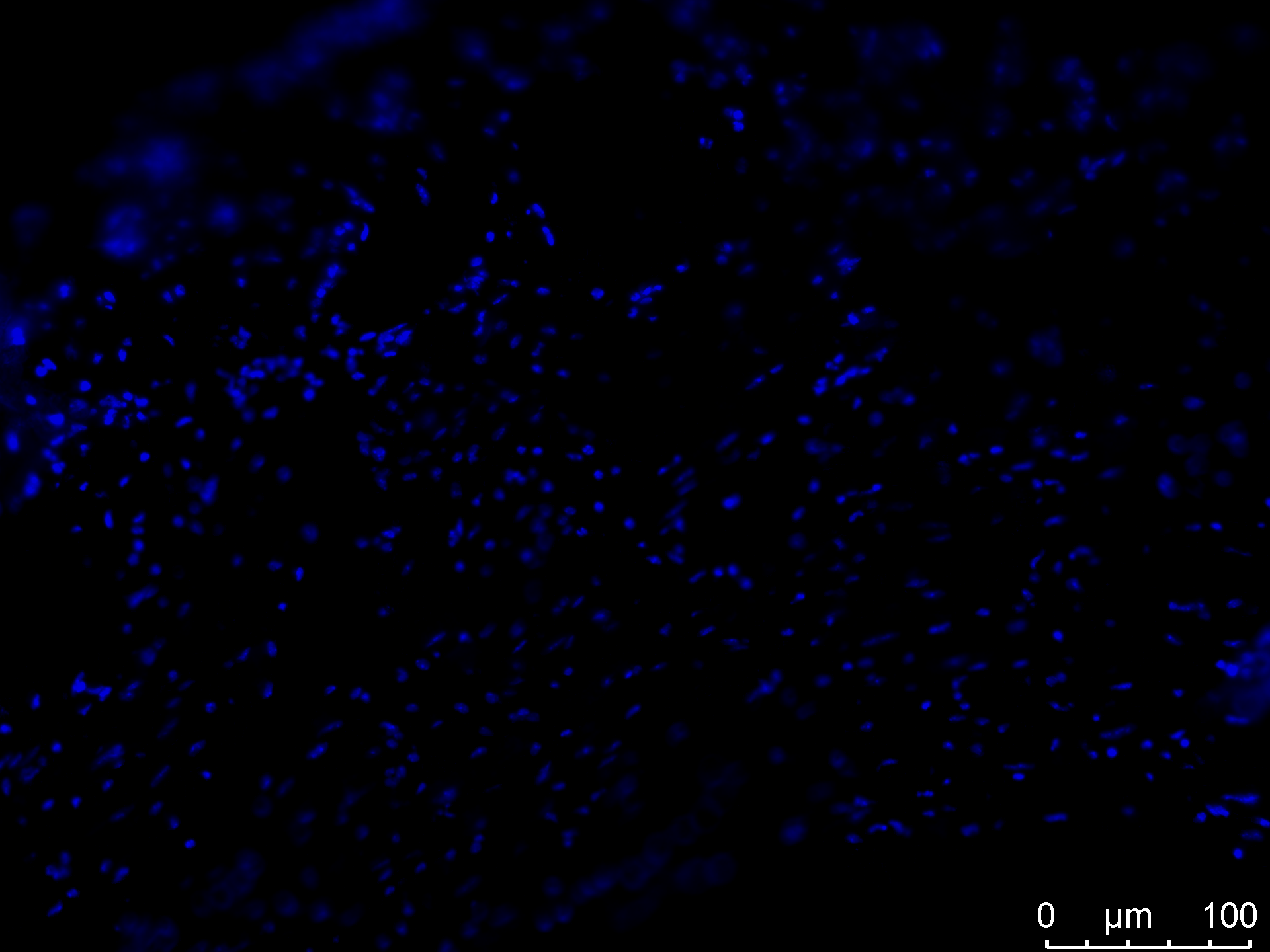

Supplement: Supplementary file 8 [file DataSheet5.zip › Figure 3/Figure 3A/original image/P100-D11.tif]

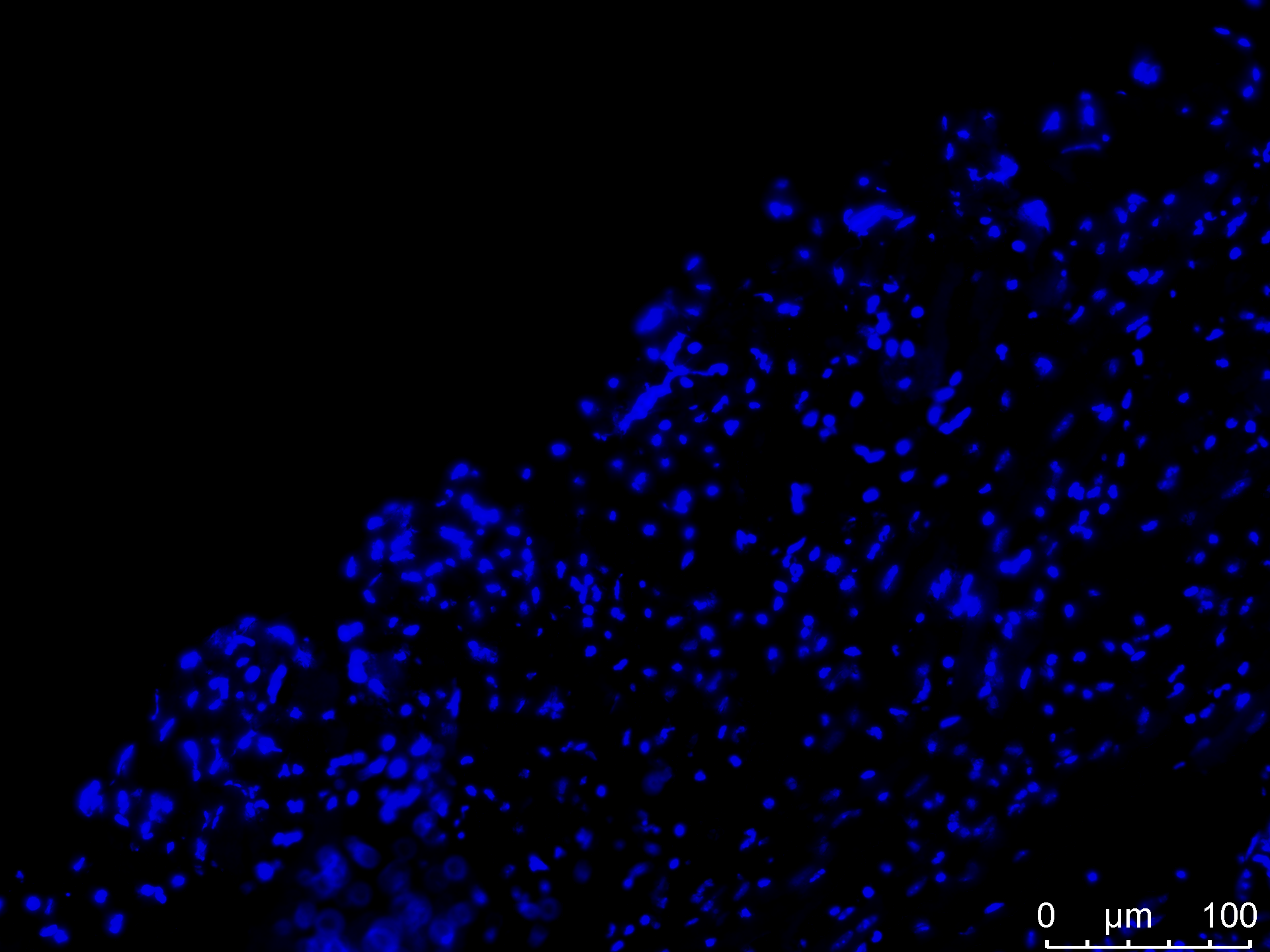

Supplement: Supplementary file 8 [file DataSheet5.zip › Figure 3/Figure 3A/original image/AMI-D10.tif]

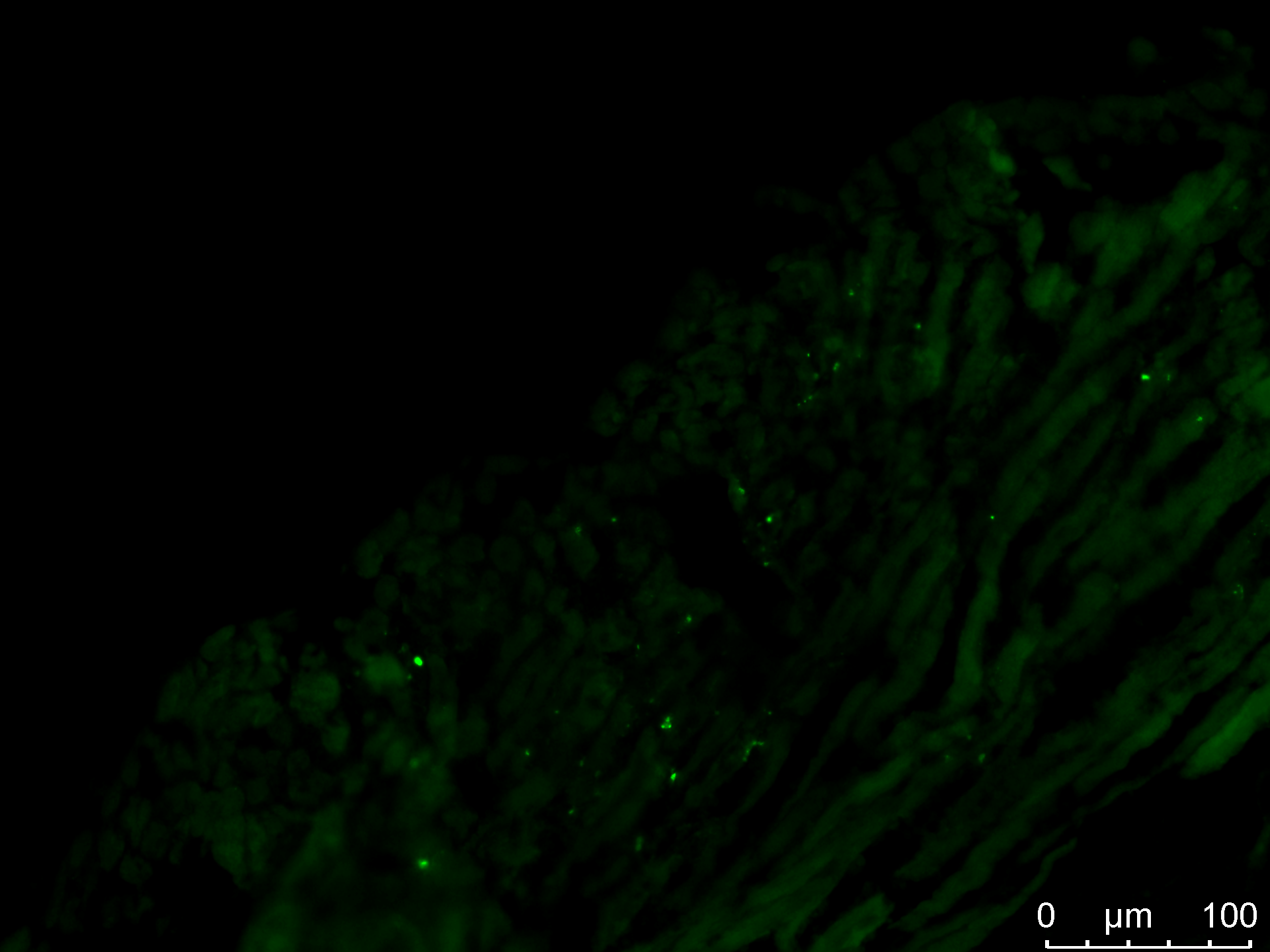

Supplement: Supplementary file 8 [file DataSheet5.zip › Figure 3/Figure 3A/original image/AMI-G10.tif]

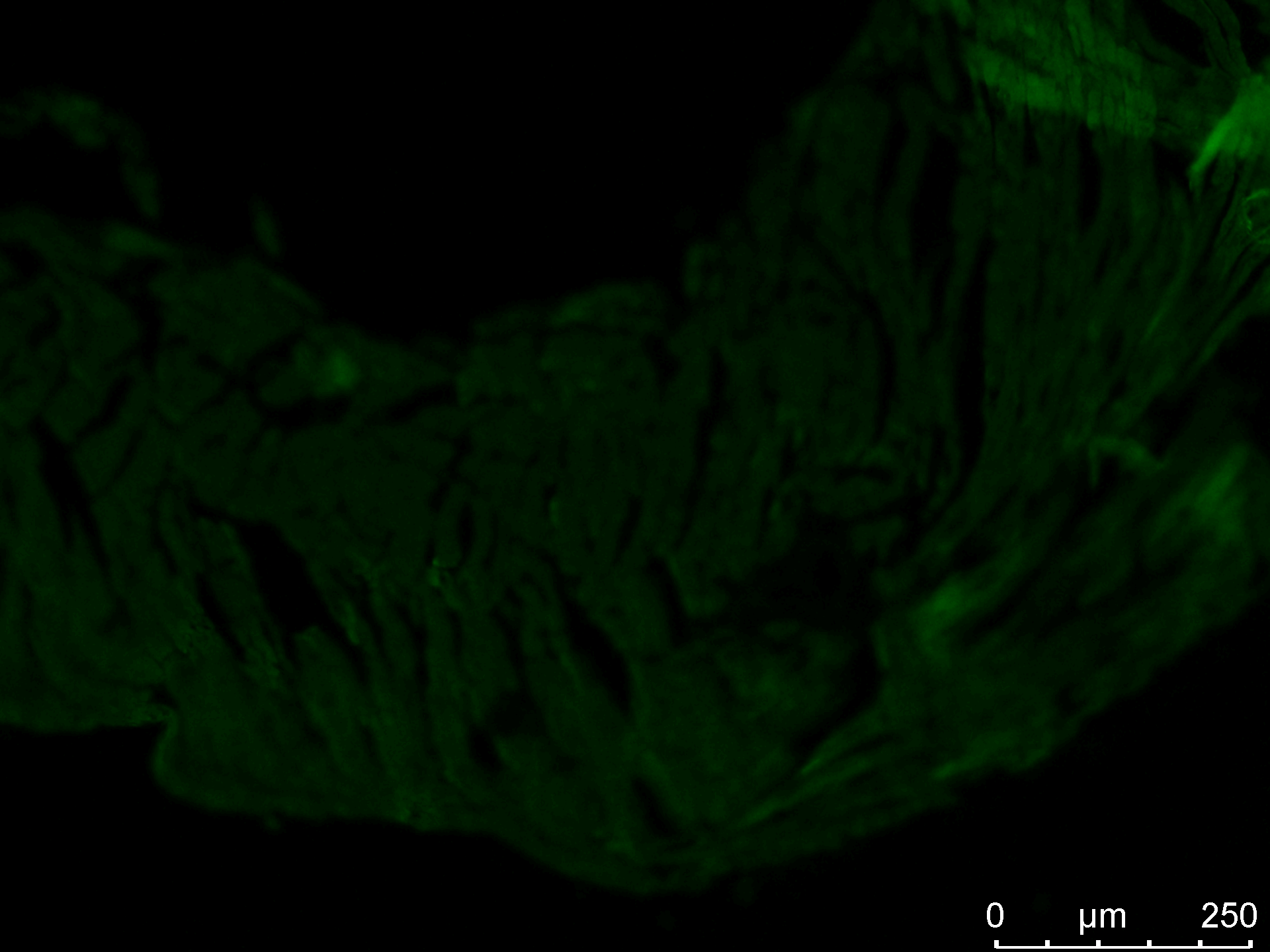

Supplement: Supplementary file 8 [file DataSheet5.zip › Figure 3/Figure 3A/original image/Sham-G1.tif]

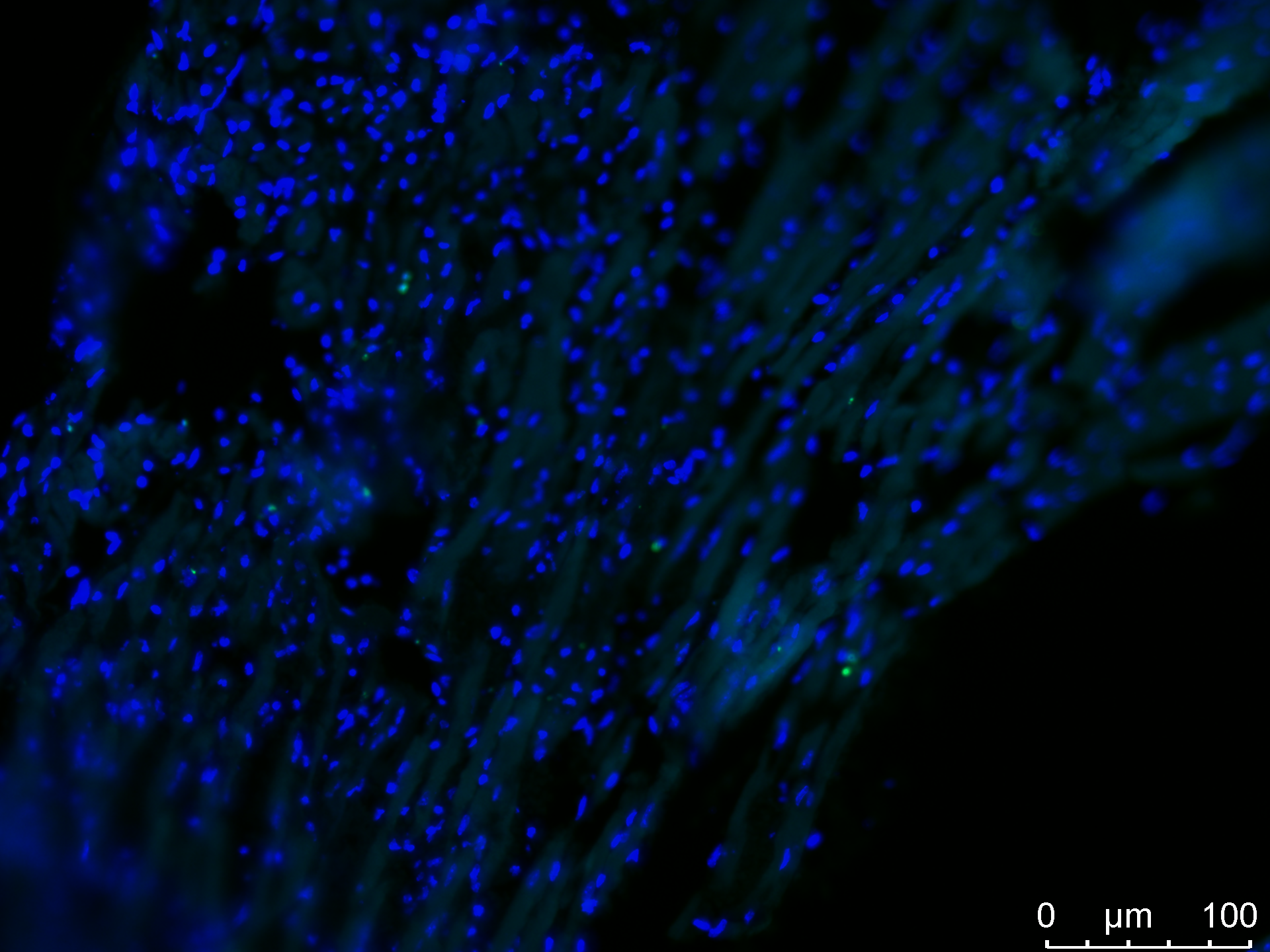

Supplement: Supplementary file 8 [file DataSheet5.zip › Figure 3/Figure 3A/original image/p50.tif]

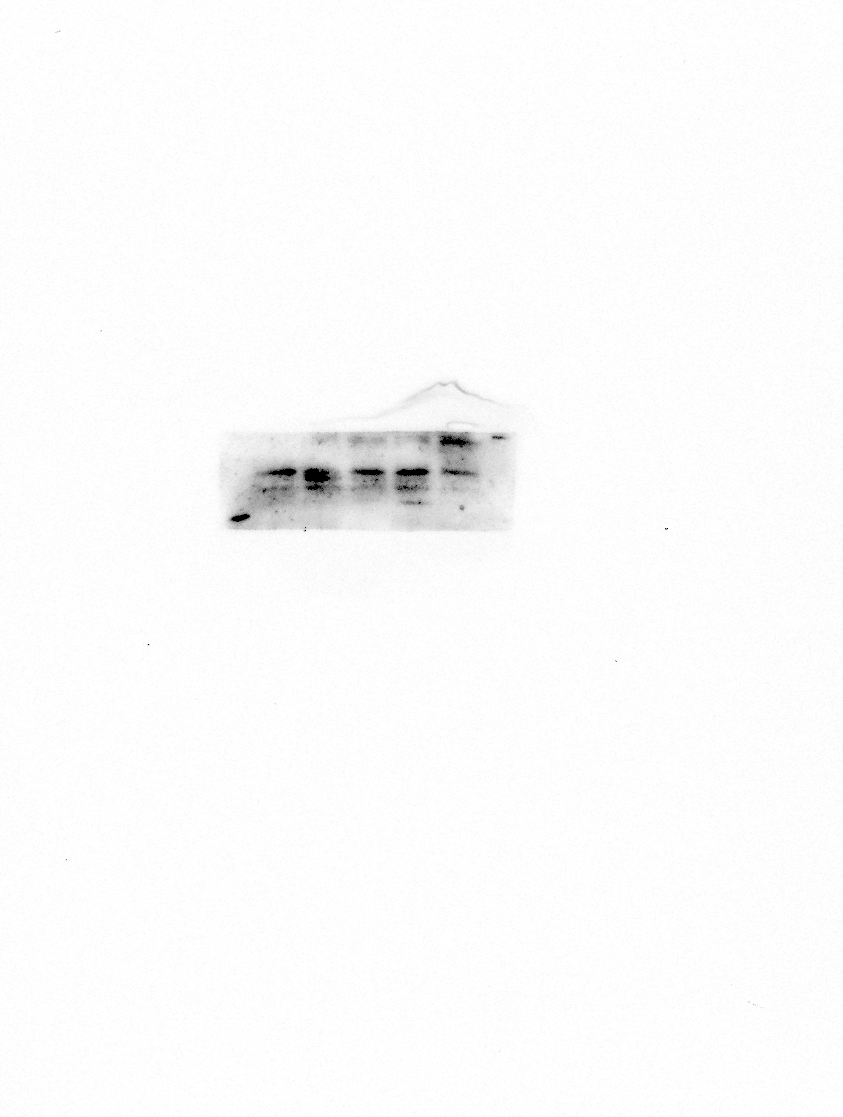

Supplement: Supplementary file 9 [file DataSheet7.zip › Figure 5/Figure 5I/caspase3-1.tif]

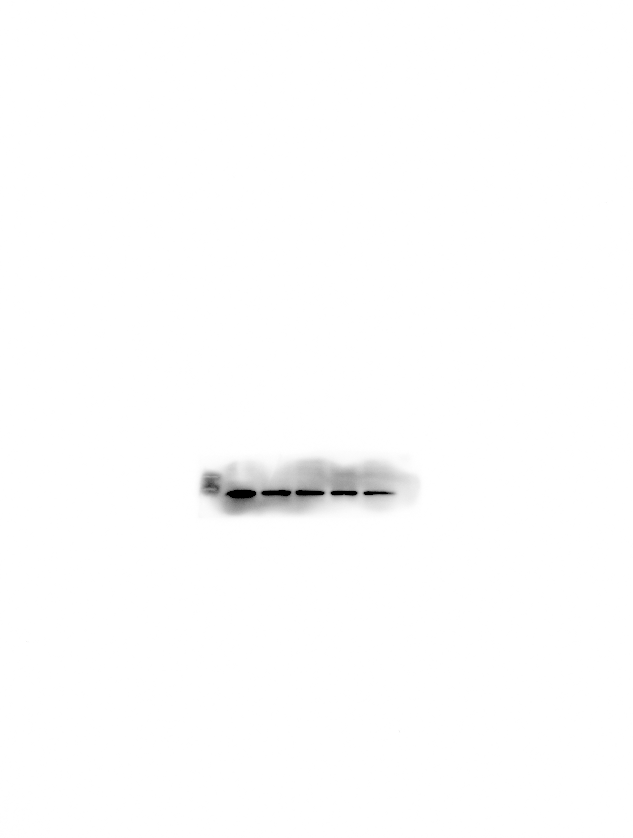

Supplement: Supplementary file 9 [file DataSheet7.zip › Figure 5/Figure 5I/pro-caspase3-1.tif]

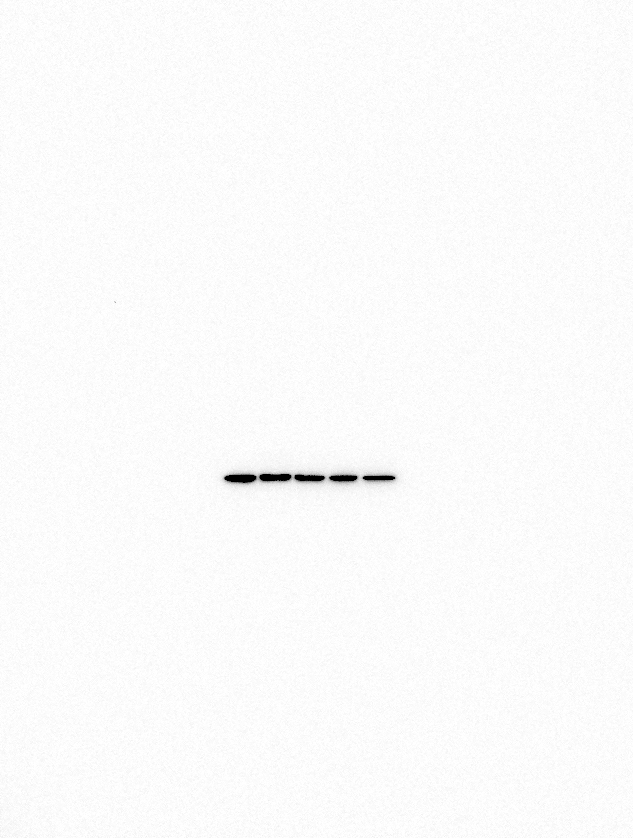

Supplement: Supplementary file 9 [file DataSheet7.zip › Figure 5/Figure 5I/GAPDH-1.tif]

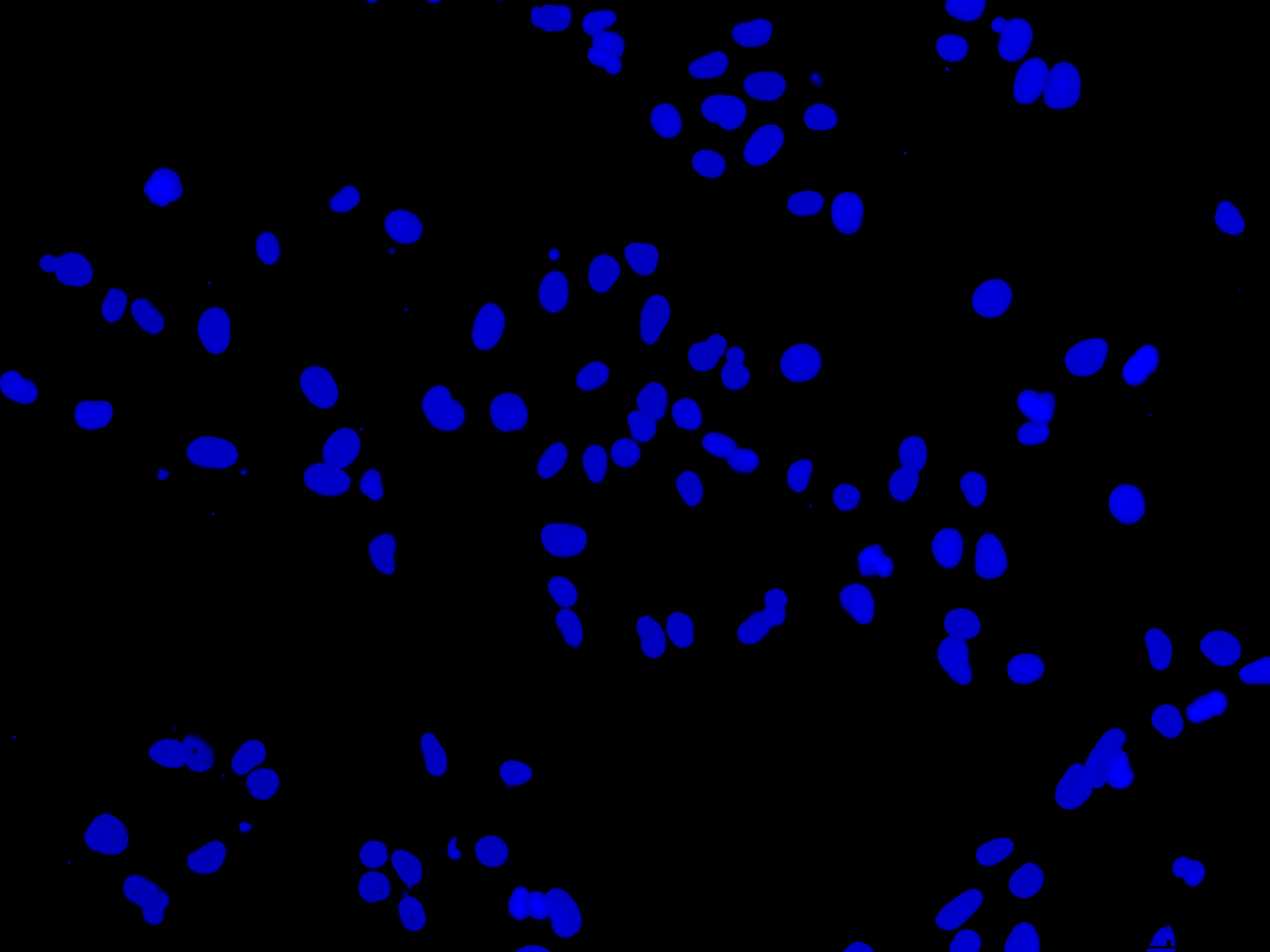

Supplement: Supplementary file 9 [file DataSheet7.zip › Figure 5/Figure 5G/ctl_33-1.tif]

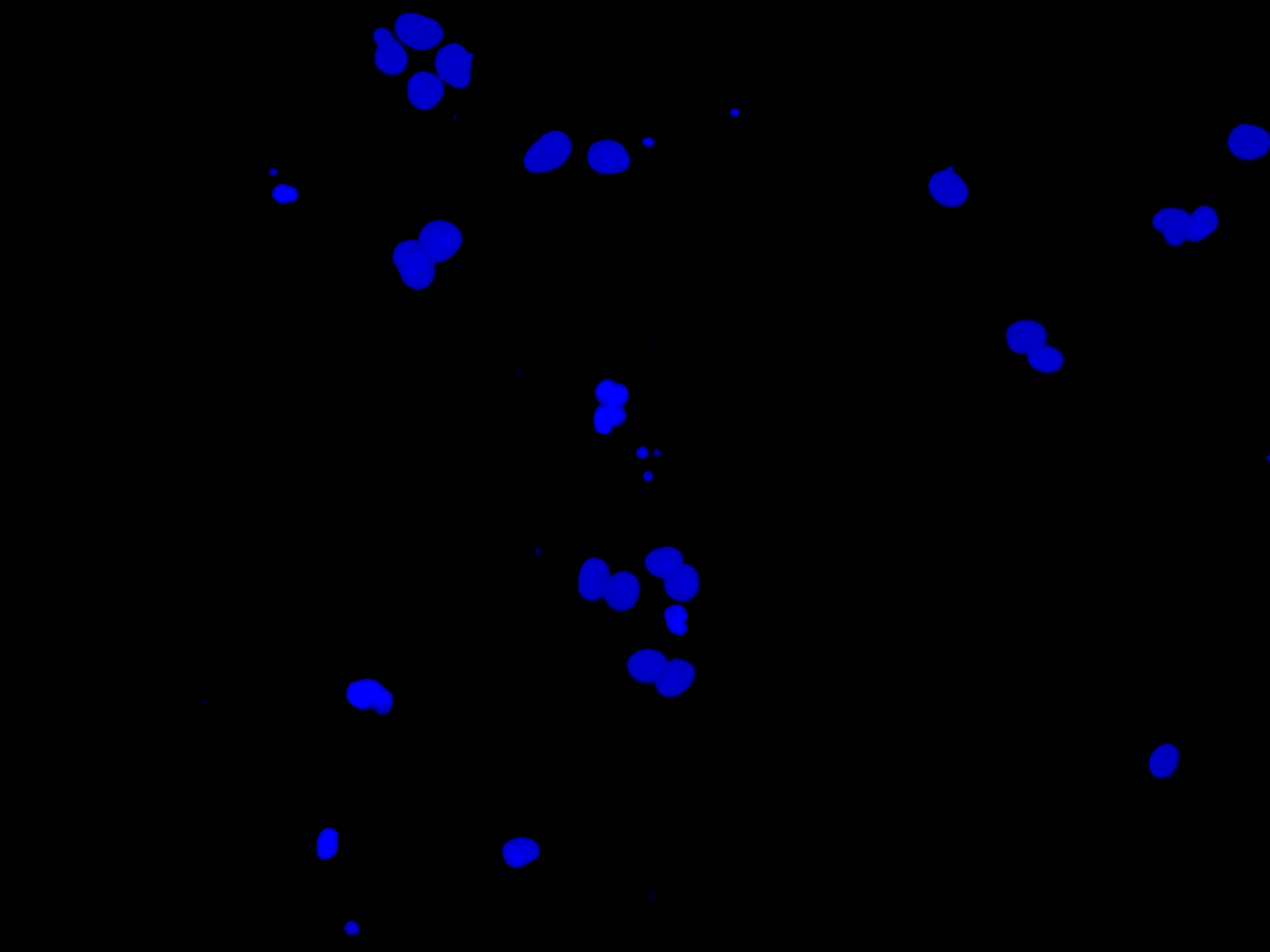

Supplement: Supplementary file 9 [file DataSheet7.zip › Figure 5/Figure 5G/h+p200_15-1.tif]

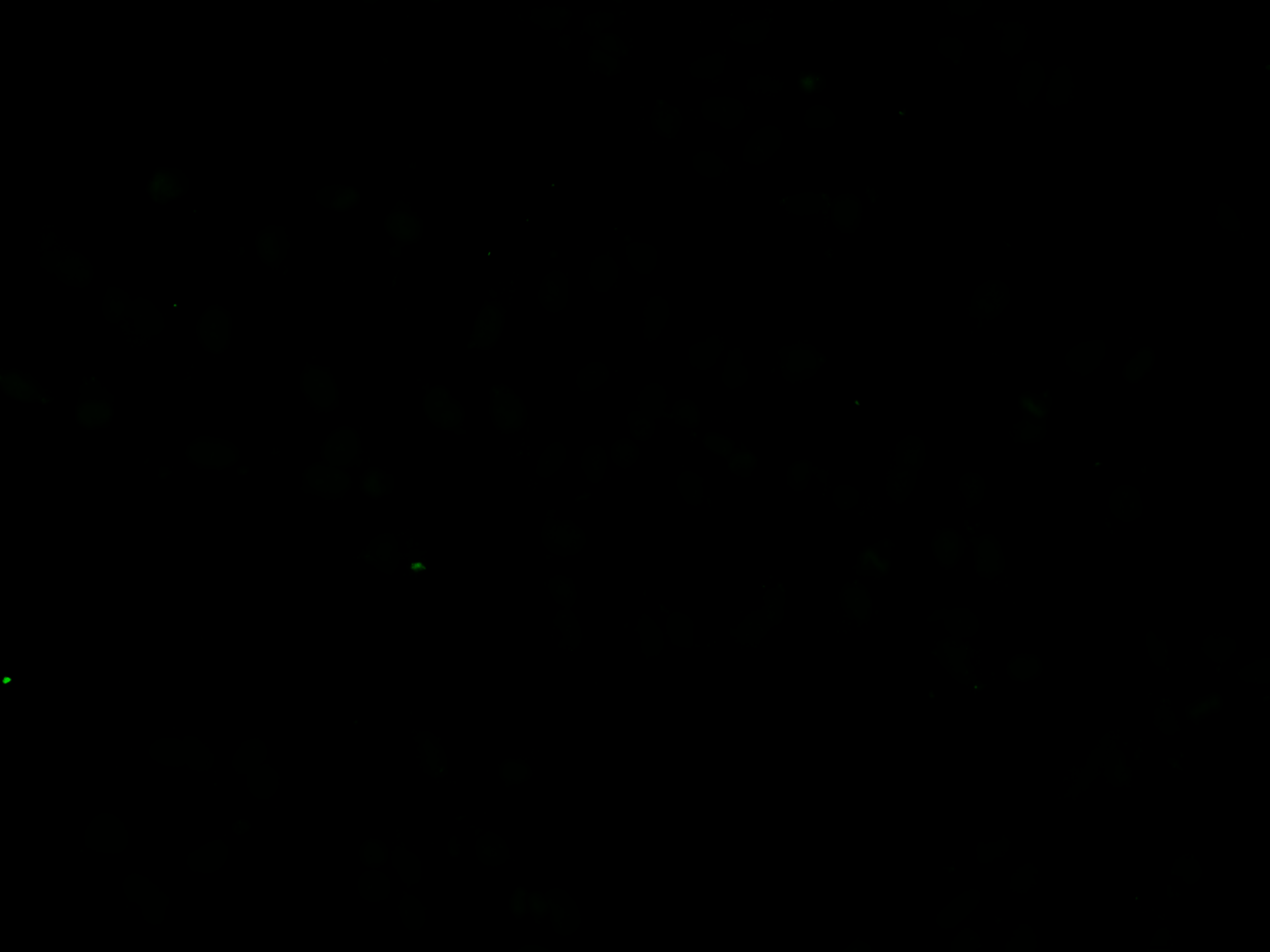

Supplement: Supplementary file 9 [file DataSheet7.zip › Figure 5/Figure 5G/ctl_33-2.tif]

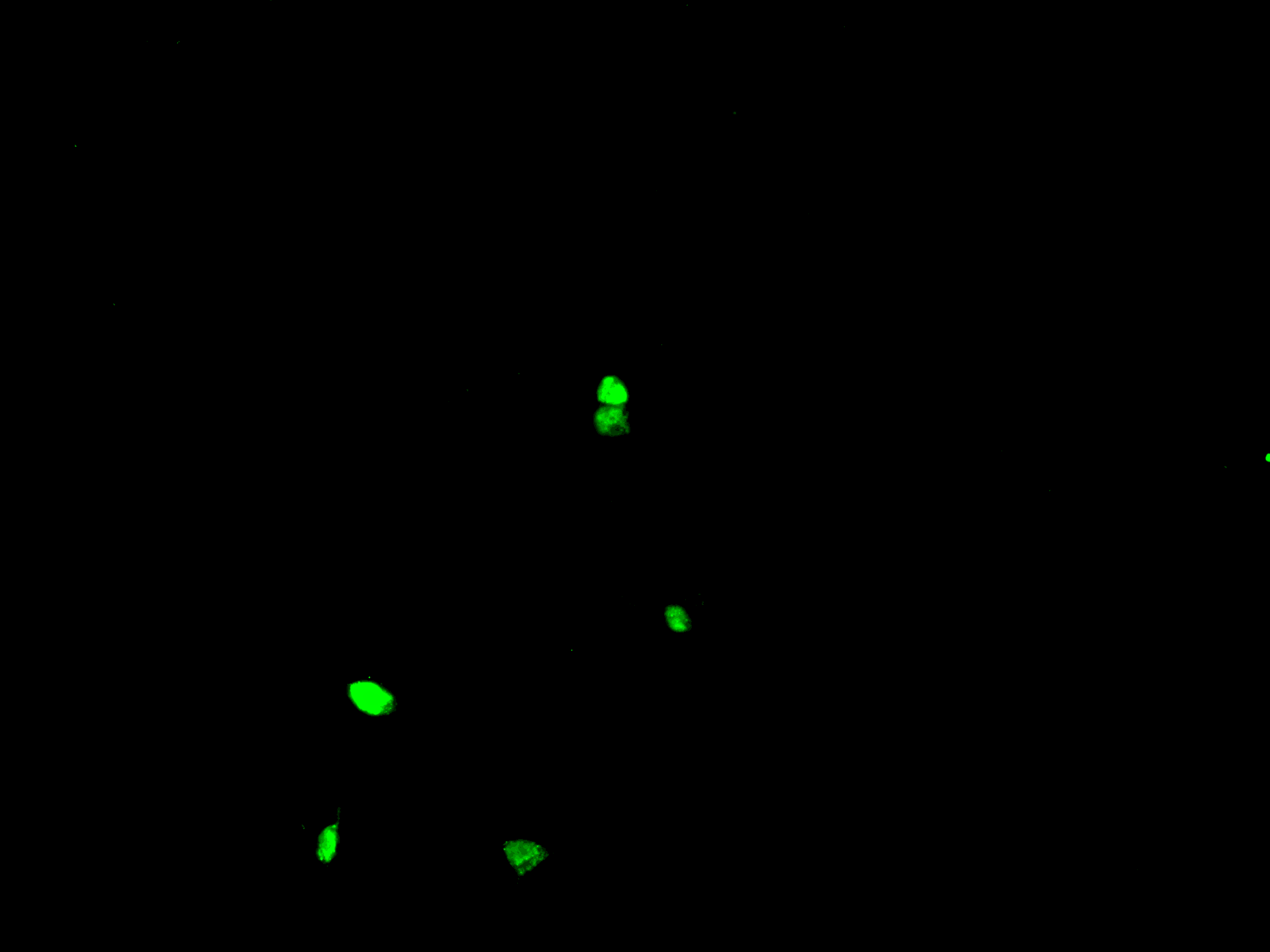

Supplement: Supplementary file 9 [file DataSheet7.zip › Figure 5/Figure 5G/h+p200_15-2.tif]

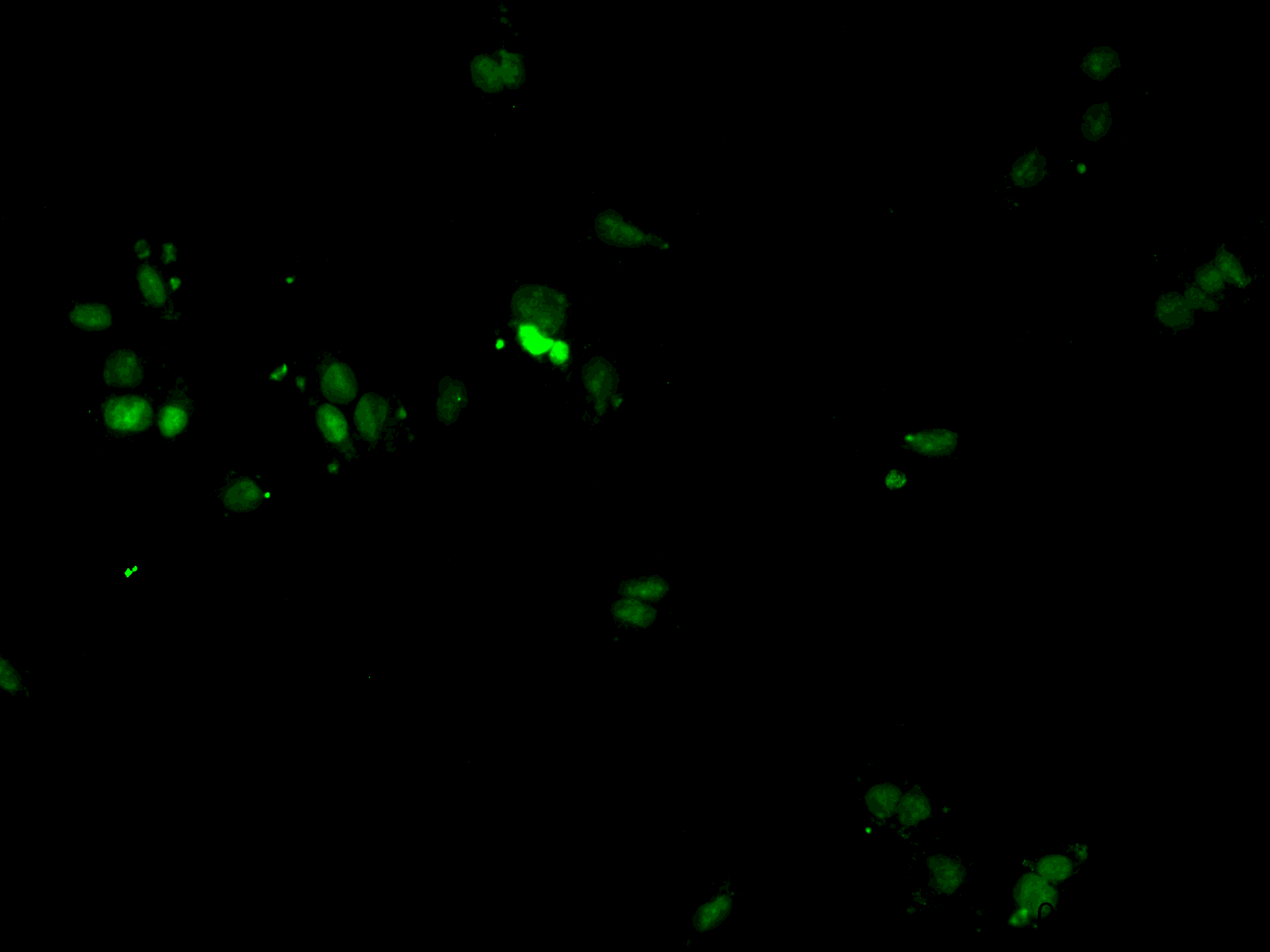

Supplement: Supplementary file 9 [file DataSheet7.zip › Figure 5/Figure 5G/h+p200+si-mzb1_8-2.tif]

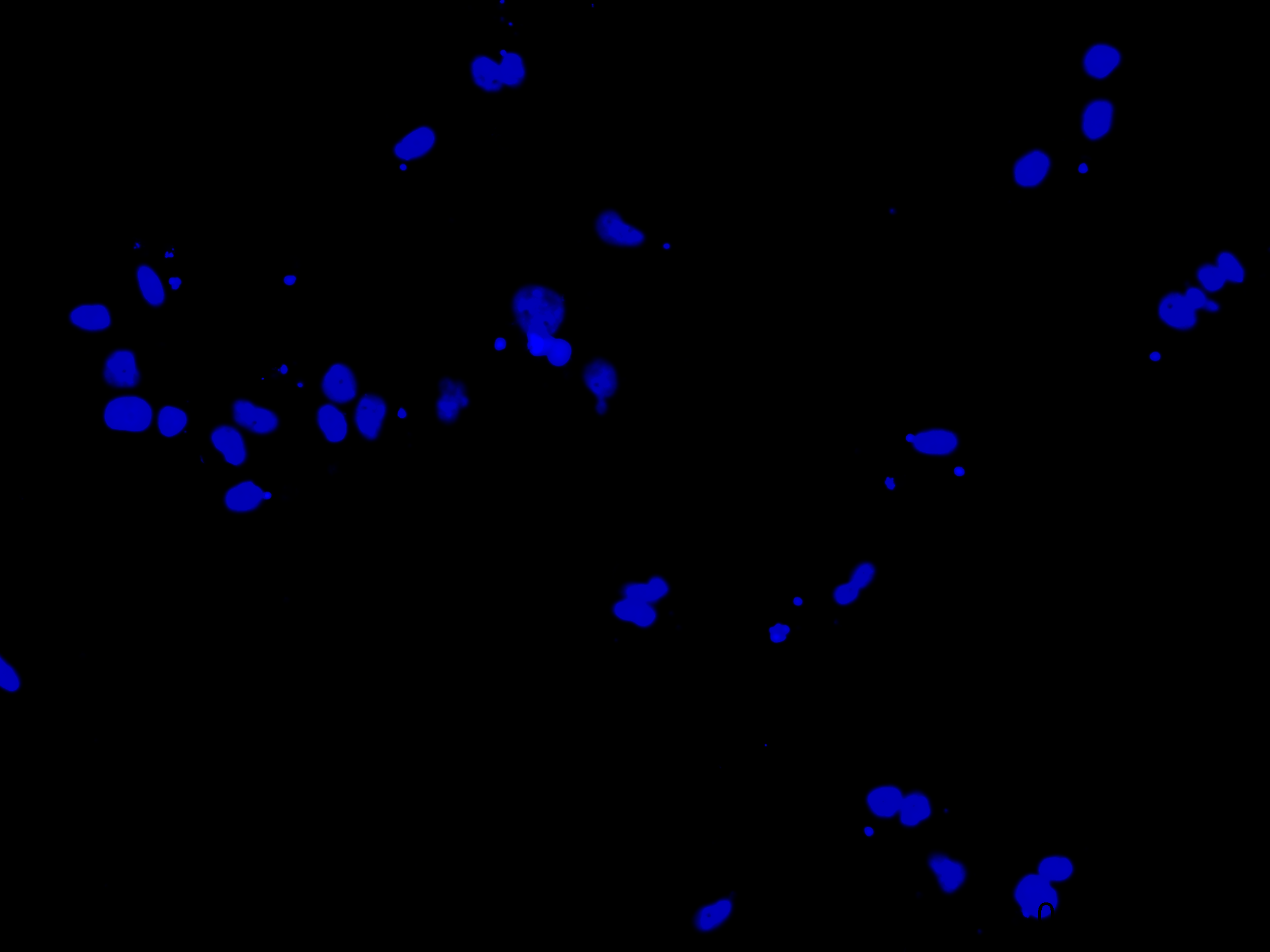

Supplement: Supplementary file 9 [file DataSheet7.zip › Figure 5/Figure 5G/h+p200+si-mzb1_8-1.tif]

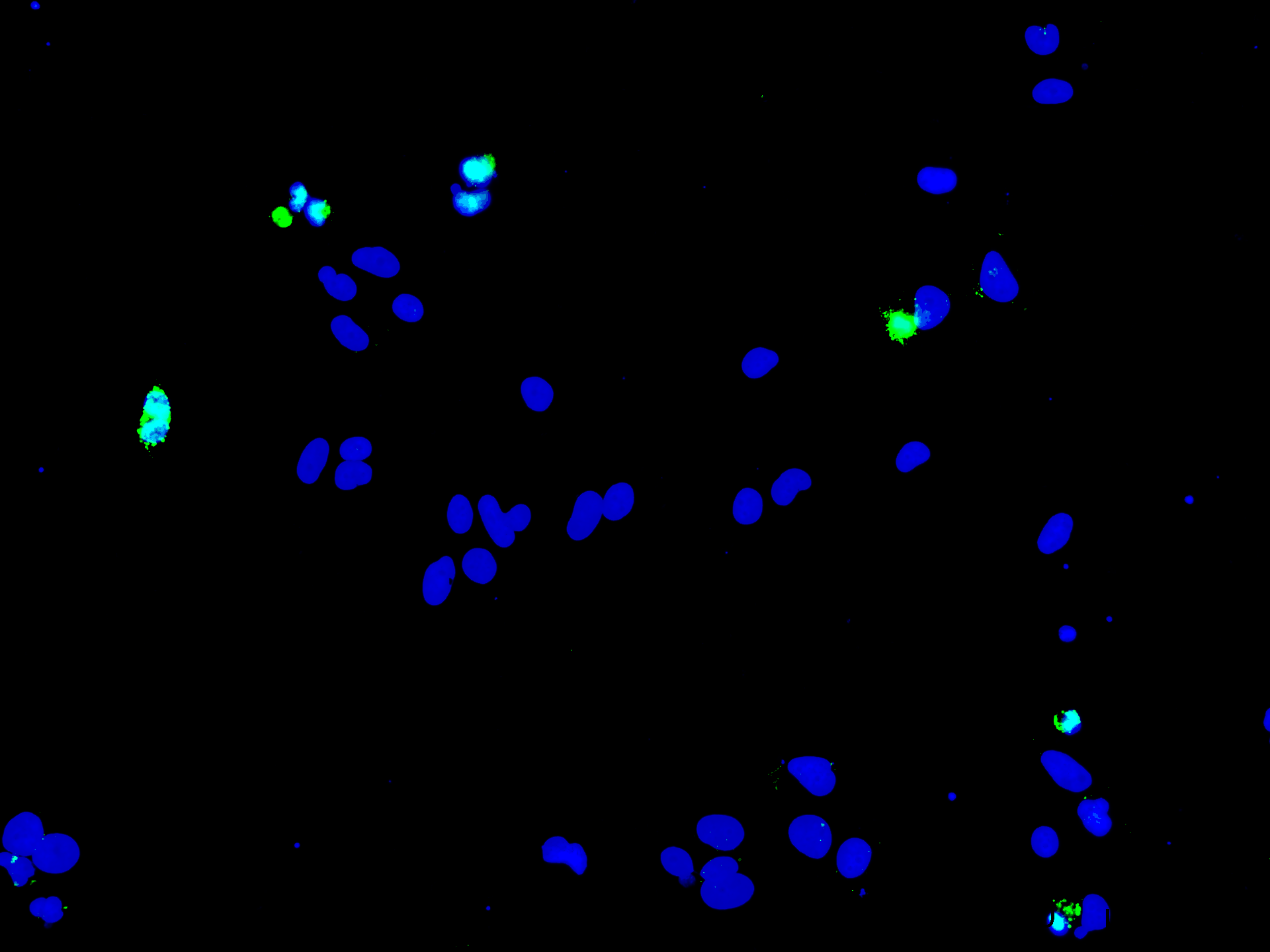

Supplement: Supplementary file 9 [file DataSheet7.zip › Figure 5/Figure 5G/h+p200+si-nc_3.tif]

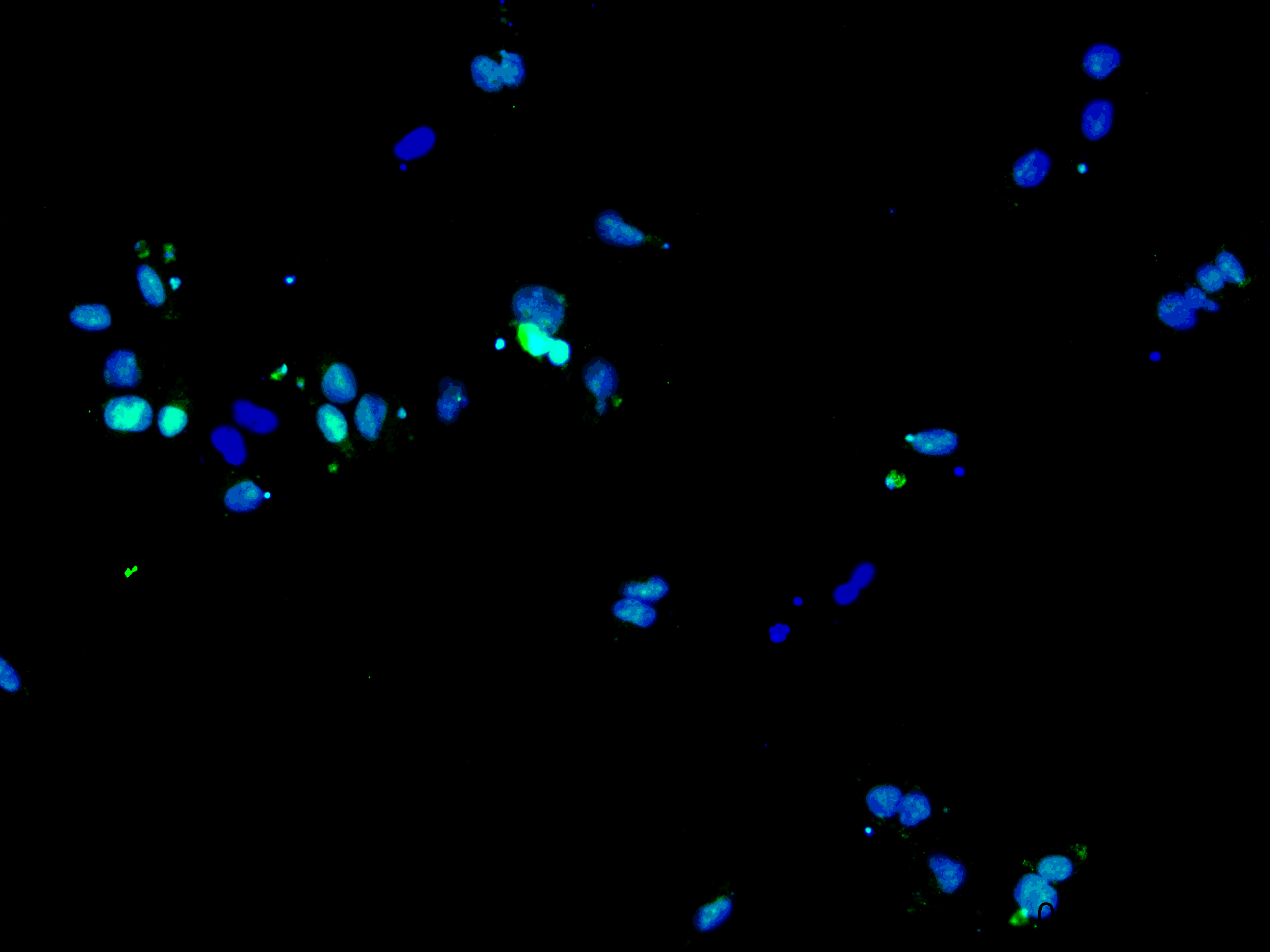

Supplement: Supplementary file 9 [file DataSheet7.zip › Figure 5/Figure 5G/h+p200+si-mzb1_8.tif]

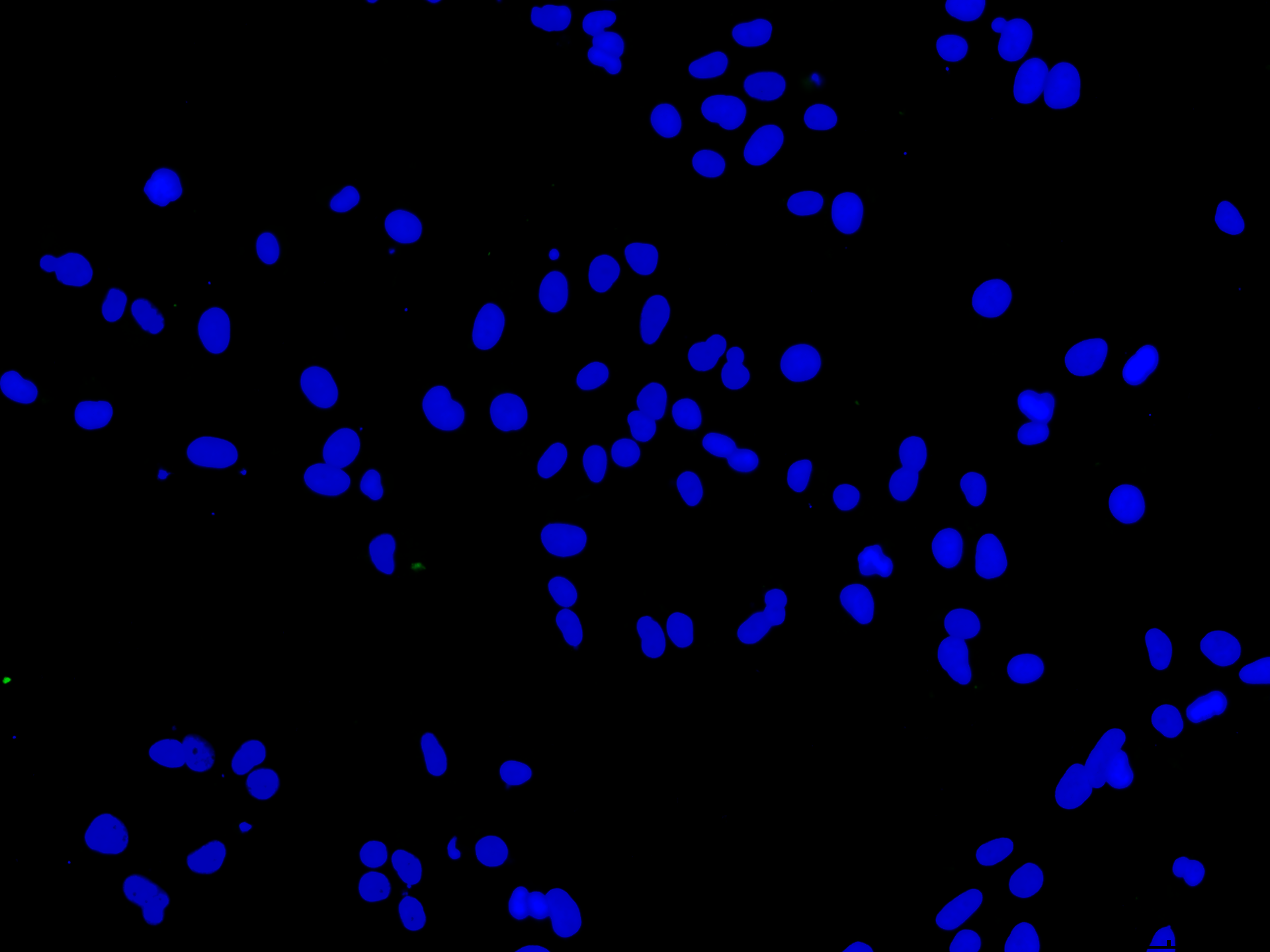

Supplement: Supplementary file 9 [file DataSheet7.zip › Figure 5/Figure 5G/ctl_33.tif]

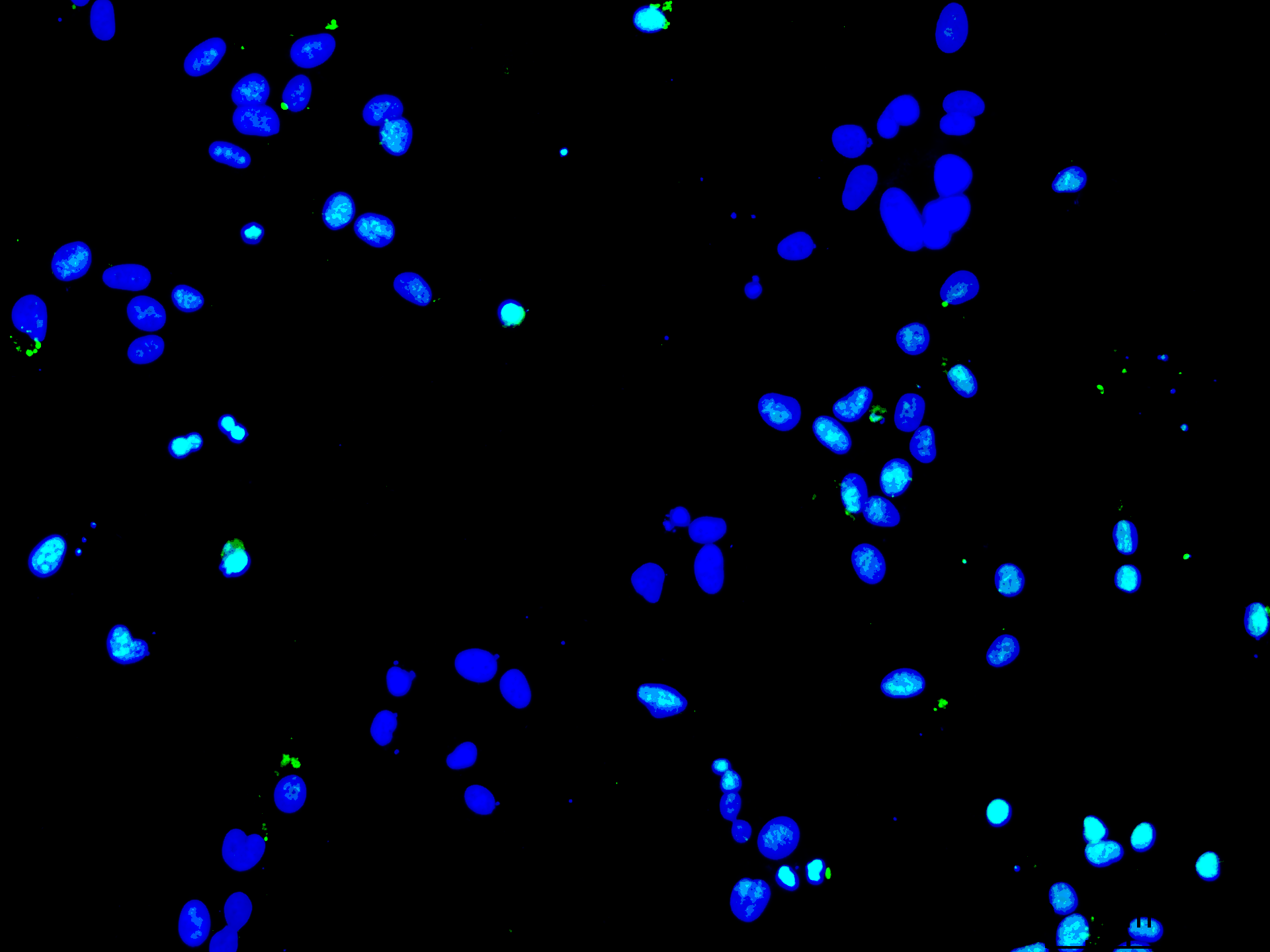

Supplement: Supplementary file 9 [file DataSheet7.zip › Figure 5/Figure 5G/h2o2_31.tif]
