# Supplementary material for: Puerarin attenuates myocardial ischemic injury and endoplasmic reticulum stress by upregulating the Mzb1 signal pathway
Source: Front Pharmacol. 2024 Aug 13;15:1442831. doi: 10.3389/fphar.2024.1442831 (PMC11350615; doi:10.3389/fphar.2024.1442831)
Supplement: Supplementary file 2 [file DataSheet8.zip › Figure 6/Figure 6G/6G.pdf]

Figure 6G

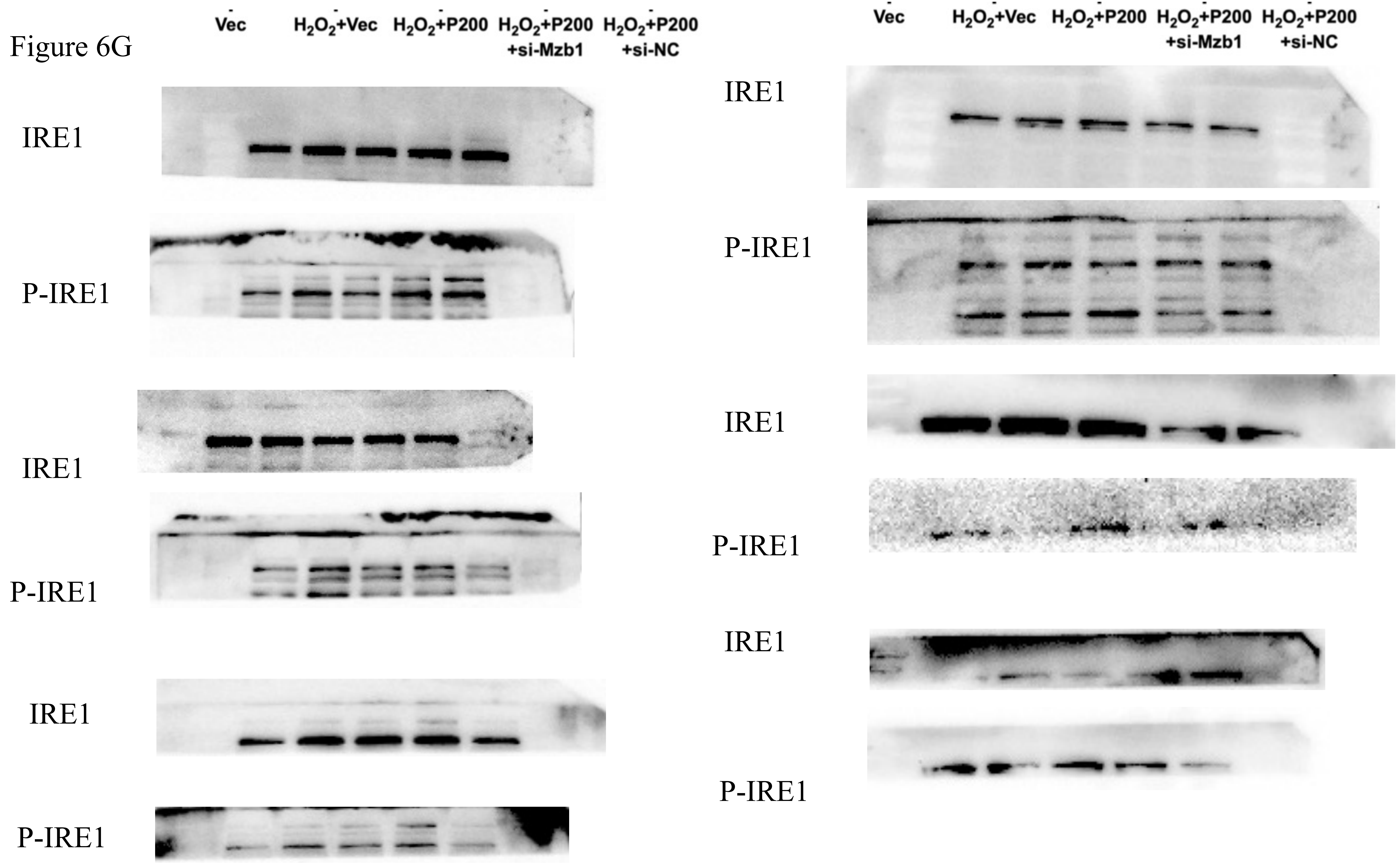
Vec
$H_2O_2$+Vec
$H_2O_2$+P200
$H_2O_2$+P200 +si-Mzb1
$H_2O_2$+P200 +si-NC
IRE1
P-IRE1
IRE1
P-IRE1
IRE1
P-IRE1
Vec
$H_2O_2$+Vec
$H_2O_2$+P200
$H_2O_2$+P200 +si-Mzb1
$H_2O_2$+P200 +si-NC
IRE1
P-IRE1
IRE1
P-IRE1
IRE1
P-IRE1

# Figure 6G

| P-IRE/IRE1 | Vec | $H_2O_2$+Vec | $H_2O_2$+P200 | $H_2O_2$+P200 +si-Mzb1 | $H_2O_2$+P200 +si-NC |
|---|---|---|---|---|---|
| | 1 | 1.756 | 0.9877 | 1.6916 | 1.313 |
| | 1 | 1.789 | 1.1612 | 1.7652 | 1.107 |
| | 1 | 2.604 | 1.1602 | 2.6775 | 1.255 |
| | 1 | 1.597 | 0.6195 | 1.157 | 0.405 |
| | 1 | 1.579 | 0.7532 | 1.375 | 1.087 |
| | 1 | 1.633888288 | 0.811337948 | 1.281426293 | 0.878824615 |
