# Supplementary material for: Puerarin attenuates myocardial ischemic injury and endoplasmic reticulum stress by upregulating the Mzb1 signal pathway
Source: Front Pharmacol. 2024 Aug 13;15:1442831. doi: 10.3389/fphar.2024.1442831 (PMC11350615; doi:10.3389/fphar.2024.1442831)
Supplement: Supplementary file 2 [file DataSheet8.zip › Figure 6/Figure 6D-E/6D-E.pdf]

Figure 6D-E

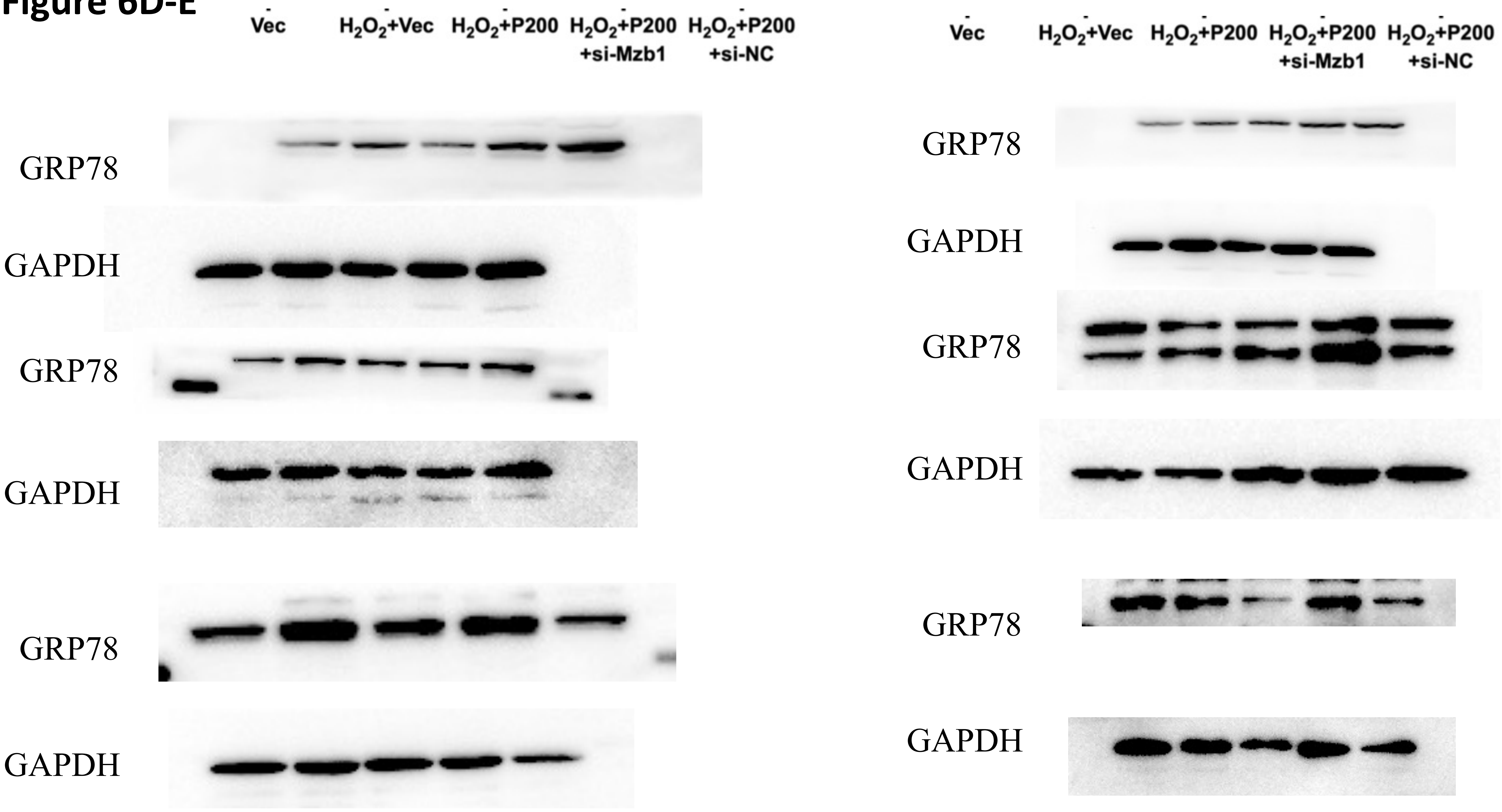

-
Vec
-
H2O2+Vec
-
H2O2+P200
-
H2O2+P200
+si-Mzb1
-
H2O2+P200
+si-NC
GRP78
GAPDH
GRP78
GAPDH
GRP78
GAPDH
-
Vec
-
H2O2+Vec
-
H2O2+P200
-
H2O2+P200
+si-Mzb1
-
H2O2+P200
+si-NC
GRP78
GAPDH
GRP78
GAPDH
GRP78
GAPDH

Figure 6D-E

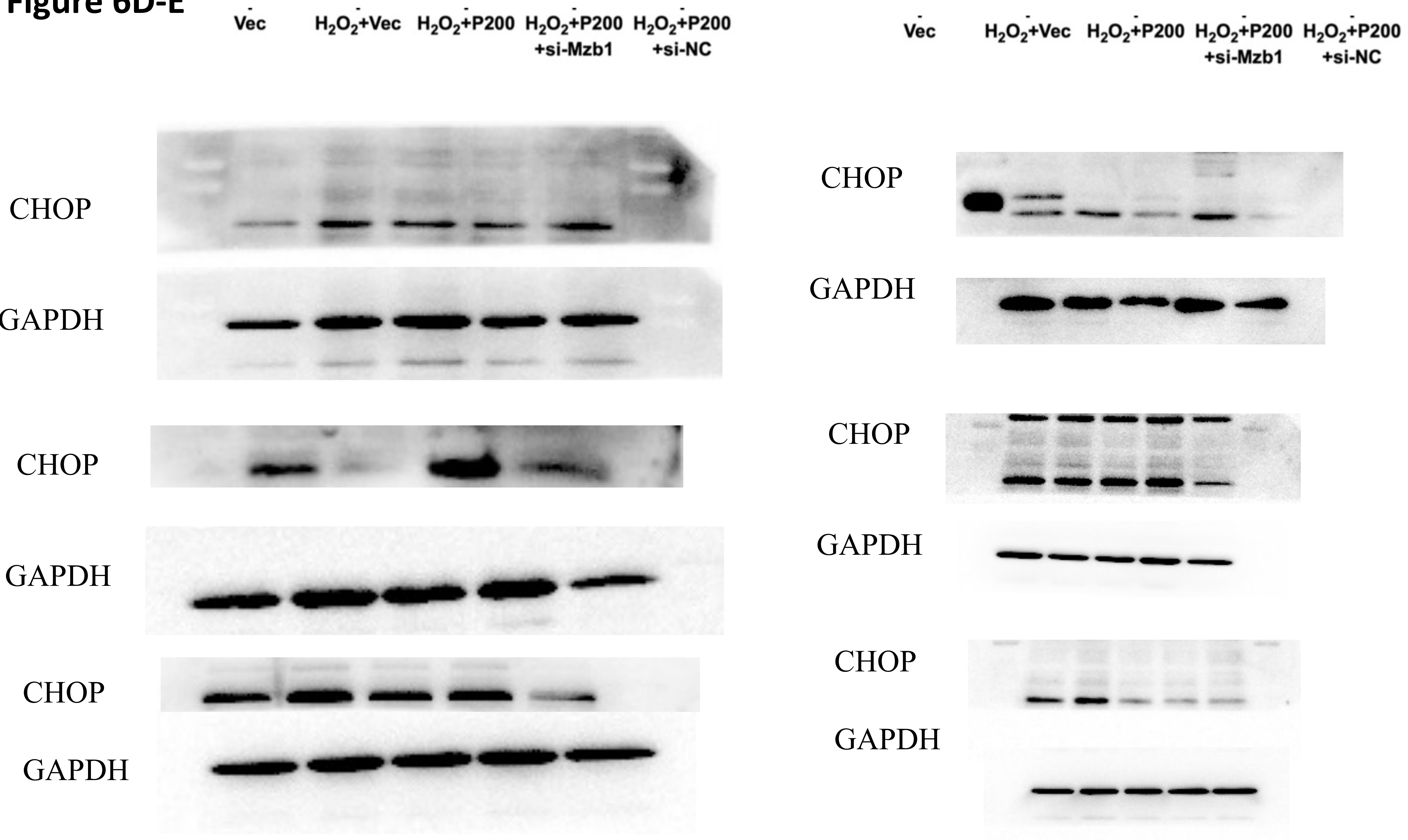
Vec
H_2O_2+Vec
H_2O_2+P200
H_2O_2+P200 +si-Mzb1
H_2O_2+P200 +si-NC
CHOP
GAPDH
CHOP
GAPDH
CHOP
GAPDH
Vec
H_2O_2+Vec
H_2O_2+P200
H_2O_2+P200 +si-Mzb1
H_2O_2+P200 +si-NC
CHOP
GAPDH
CHOP
GAPDH
CHOP
GAPDH

Figure 6D-E

GRP78	Vec	H_2O_2+Vec	H_2O_2+P200	H_2O_2+P200 +si-Mzb1	H_2O_2+P200 +si-NC
	1	1.915	1.0796	1.7484	1.29
	1	1.604	1.1659	1.58	1.14
	1	1.716	1.1908	1.8521	1.381
	1	1.714343546	1.109368675	1.810330371	1.290883944
	1	2.1733877	1.400616472	1.880482051	1.629215311
	1	1.628437274	1.048415851	1.921600318	1.046299019

CHOP	Vec	H_2O_2+Vec	H_2O_2+P200	H_2O_2+P200 +si-Mzb1	H_2O_2+P200 +si-NC
	1	1.421	0.9903	1.2747	0.709
	1	2.105	1.3013	2.0944	1.521
	1	1.732	0.7181	1.3143	0.644
	1	1.517	1.1667	1.4831	0.996
	1	2.643794048	1.520645907	3.321188288	2.132375339
	1	1.507695715	1.017702356	1.426919448	0.914371823
