# Supplementary material for: Puerarin attenuates myocardial ischemic injury and endoplasmic reticulum stress by upregulating the Mzb1 signal pathway
Source: Front Pharmacol. 2024 Aug 13;15:1442831. doi: 10.3389/fphar.2024.1442831 (PMC11350615; doi:10.3389/fphar.2024.1442831)
Supplement: Supplementary file 2 [file DataSheet8.zip › Figure 6/Figure 6B/6B.pdf]

Figure 6B

	Vec	H_2O_2+Vec	H_2O_2+P200	H_2O_2+P200 +si-Mzb1	H_2O_2+P200 +si-NC
ROS	18	55	20	69	19
	20	80	17	57	22
	18	56	23	68	19
	13	71	27	67	27
	14	74	23	65	27
	9	85	14	70	20
