# Supplementary material for: Puerarin attenuates myocardial ischemic injury and endoplasmic reticulum stress by upregulating the Mzb1 signal pathway
Source: Front Pharmacol. 2024 Aug 13;15:1442831. doi: 10.3389/fphar.2024.1442831 (PMC11350615; doi:10.3389/fphar.2024.1442831)
Supplement: Supplementary file 3 [file DataSheet9.zip › Figure 7/Figure 7D/7D.pdf]

Figure 7D

Vec | H_2O_2+Vec | H_2O_2+P200 | H_2O_2+P200 +si-Mzb1 | H_2O_2+P200 +si-NC

DRP1

P-DRP1

DRP1

P-DRP1

DRP1

P-DRP1

DRP1

P-DRP1

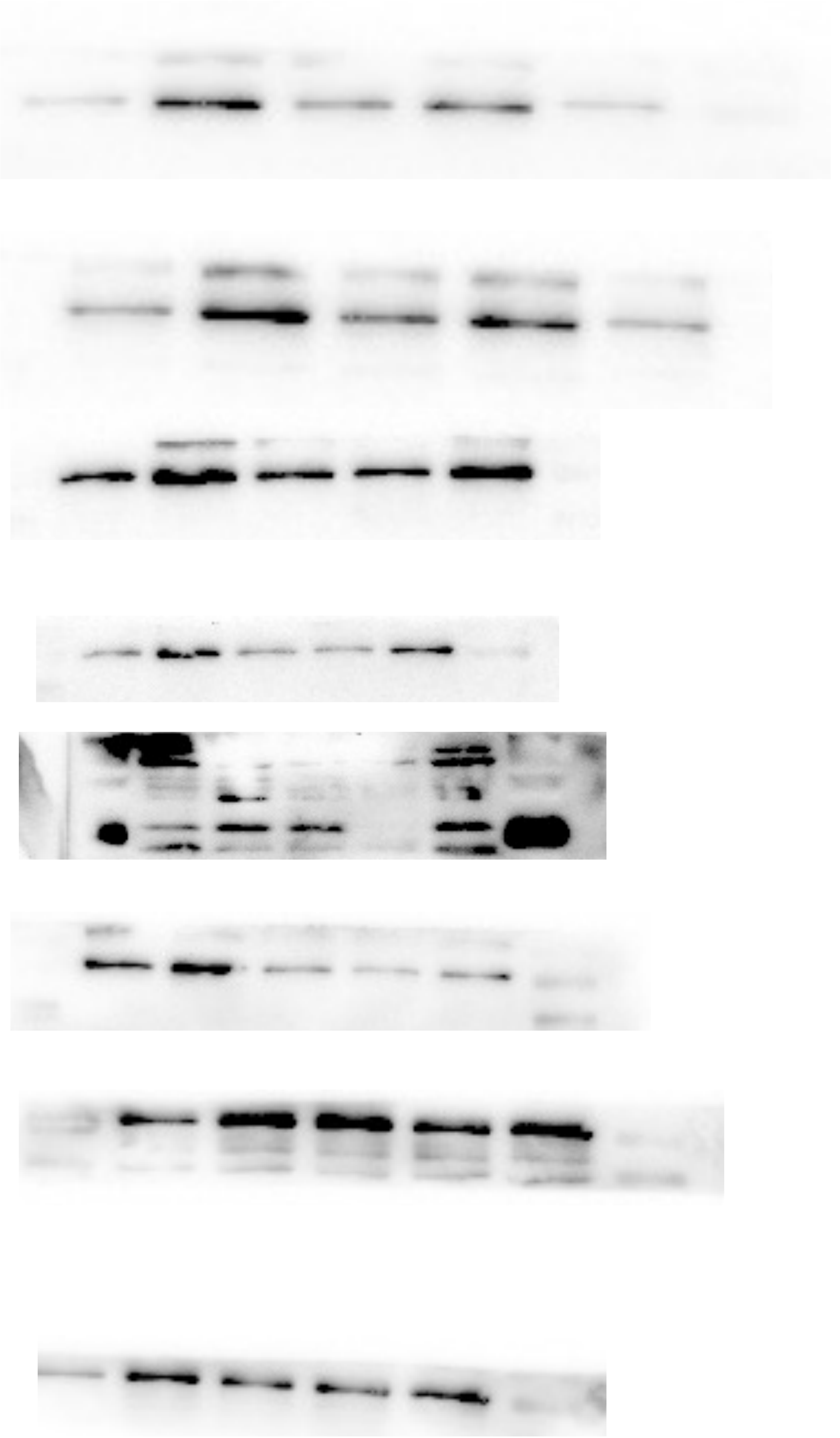

P-DRP1/DRP1	Vec	H_2O_2+Vec	H_2O_2+P200	H_2O_2+P200 +si-Mzb1	H_2O_2+P200 +si-NC
	1	1.717	1.2605	1.6303	1.318
	1	1.767249775	0.663309004	1.011383223	0.58581209
	1	1.5604805	0.955451788	1.522216985	0.977538102
	1	1.757699969	0.923964927	1.602483552	1.130764171
