# Supplementary material for: Puerarin attenuates myocardial ischemic injury and endoplasmic reticulum stress by upregulating the Mzb1 signal pathway
Source: Front Pharmacol. 2024 Aug 13;15:1442831. doi: 10.3389/fphar.2024.1442831 (PMC11350615; doi:10.3389/fphar.2024.1442831)
Supplement: Supplementary file 3 [file DataSheet9.zip › Figure 7/Figure 7C/7C.pdf]

Figure 7C

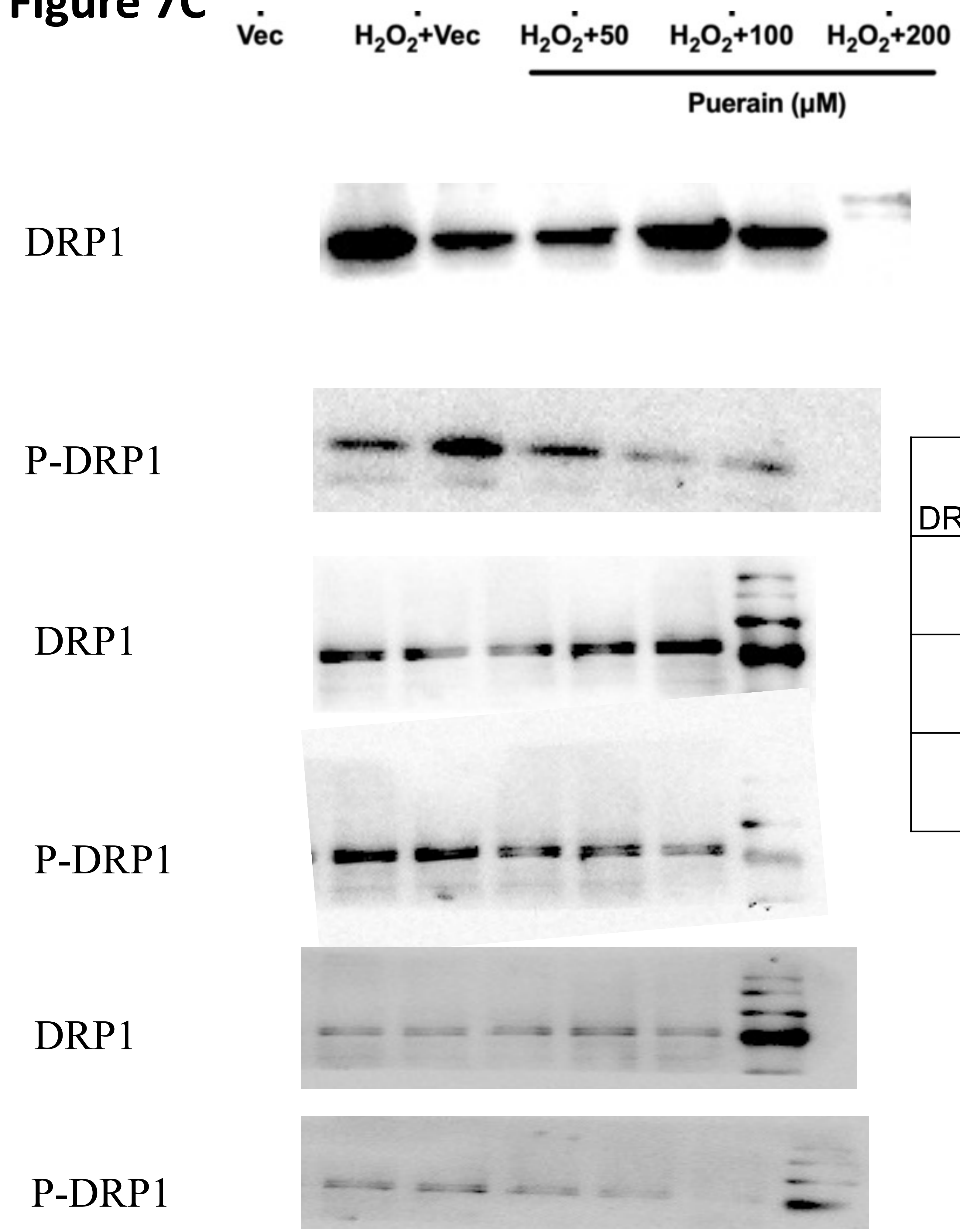

P-DRP1/DRP1	Vec	H_2O_2+Vec	H_2O_2+50	H_2O_2+100	H_2O_2+200
	1	1.55	1.08	0.7	0.38
	1	1.69	1.01	0.51	0.46
	1	2.87	1.93	0.71	0.46
