# Supplementary material for: Puerarin attenuates myocardial ischemic injury and endoplasmic reticulum stress by upregulating the Mzb1 signal pathway
Source: Front Pharmacol. 2024 Aug 13;15:1442831. doi: 10.3389/fphar.2024.1442831 (PMC11350615; doi:10.3389/fphar.2024.1442831)
Supplement: Supplementary file 3 [file DataSheet9.zip › Figure 7/Figure 7B/7B.pdf]

Figure 7B

ATP	Vec	H_2O_2+Vec	H_2O_2+P200	H_2O_2+P200 +si-Mzb1	H_2O_2+P200 +si-NC
	29.71787738	10.02887729	24.72312916	10.44907564	22.31164824
	29.77324753	7.968521928	24.17087418	4.821727462	21.51203471
	22.29242745	8.388930715	20.64543179	10.9934741	10.9934741
