# Supplementary material for: Puerarin attenuates myocardial ischemic injury and endoplasmic reticulum stress by upregulating the Mzb1 signal pathway
Source: Front Pharmacol. 2024 Aug 13;15:1442831. doi: 10.3389/fphar.2024.1442831 (PMC11350615; doi:10.3389/fphar.2024.1442831)
Supplement: Supplementary file 3 [file DataSheet9.zip › Figure 7/Figure 7A/7A.pdf]

Figure 7A

ATP	Vec	H_2O_2+Vec	H_2O_2+50	H_2O_2+100	H_2O_2+200
	22.21635729	7.728296708	11.93970229	18.95240463	19.31367709
	21.58300613	8.584989795	13.52675404	18.10297129	26.14820827
	15.62626309	9.649372852	11.66194227	15.30278109	20.46299087
	22.29242745	8.388930715	14.82216138	19.26460074	25.95534569
	35.89941555	9.193087861	14.13520518	17.94927507	20.64543179
	7.381864457	6.838459137	12.30653872	13.47682862	11.66346378
