# Supplementary material for: Puerarin attenuates myocardial ischemic injury and endoplasmic reticulum stress by upregulating the Mzb1 signal pathway
Source: Front Pharmacol. 2024 Aug 13;15:1442831. doi: 10.3389/fphar.2024.1442831 (PMC11350615; doi:10.3389/fphar.2024.1442831)
Supplement: Supplementary file 4 [file DataSheet4.zip › Figure 2/Figure 2C/2C data.pdf]

Figure 2C

	Sham	AMI+ Vec	AMI+ Pue50	AMI+ Pue100
NADPH	528.428094	1990.52133	959.677419	784.037559
	629.343629	1777.35849	674.129353	747.368421
	508.62069	1547.26368	1025.73529	483.636364
	431.623932	1707.62712	1165.41353	844.919786
	532.608696	2676.47059	1108.10811	809.917355
	555.147059	2481.48148	1064.17112	859.848485
	594.285714	1641.50943	1100.41841	444.444444
	692.307692	1748.3871	948.051948	723.021583
