# Supplementary material for: Puerarin attenuates myocardial ischemic injury and endoplasmic reticulum stress by upregulating the Mzb1 signal pathway
Source: Front Pharmacol. 2024 Aug 13;15:1442831. doi: 10.3389/fphar.2024.1442831 (PMC11350615; doi:10.3389/fphar.2024.1442831)
Supplement: Supplementary file 4 [file DataSheet4.zip › Figure 2/Figure 2B/2B data.pdf]

Figure 2B

	Sham	AMI+ Vec	AMI+ Pue50	AMI+ Pue100
DHE	0.65398592	3.91765626	2.56618273	0.73932018
	1.20156481	3.64277145	2.06815613	1.15503782
	1.17475441	2.72422846	1.79932192	1.25727202
	1.01399635	2.81290098	2.14253673	1.5132748
	1.04278884	3.35776754	1.33978962	1.45193428
	0.91290968	4.38563853	1.61352691	1.40811962
