# Supplementary material for: Puerarin attenuates myocardial ischemic injury and endoplasmic reticulum stress by upregulating the Mzb1 signal pathway
Source: Front Pharmacol. 2024 Aug 13;15:1442831. doi: 10.3389/fphar.2024.1442831 (PMC11350615; doi:10.3389/fphar.2024.1442831)
Supplement: Supplementary file 5 [file DataSheet10.zip › Figure 8/Figure 8I/8I.pdf]

Figure 8I

MTT	Vec	H_2O_2+Vec	H_2O_2+P200	H_2O_2+P200 +Kenpaullone
	101	29	72	30
	80	29	81	49
	100	56	76	48
	105	34	86	26
	86	45	85	31
	117	21	88	51
	103	34	96	29
	109	46	85	50
	98	30	60	28
	100	52	71	49
