# Supplementary material for: Puerarin attenuates myocardial ischemic injury and endoplasmic reticulum stress by upregulating the Mzb1 signal pathway
Source: Front Pharmacol. 2024 Aug 13;15:1442831. doi: 10.3389/fphar.2024.1442831 (PMC11350615; doi:10.3389/fphar.2024.1442831)
Supplement: Supplementary file 5 [file DataSheet10.zip › Figure 8/Figure 8A/8A.pdf]

Figure 8A

MZB1 marginal zone B and B1 cell specific protein [*Homo sapiens* (human)]

Genomic context

Annotation release	Status	Assembly	Chr	Location
RS_2023_10	current	GRCh38.p14 (GCF_000001405.40)	5	NC_000005.10 (139387467..139389913, complement)
RS_2023_10	current	T2T-CHM13v2.0 (GCF_009914755.1)	5	NC_060929.1 (139913564..139916009, complement)
105.20220307	previous assembly	GRCh37.p13 (GCF_000001405.25)	5	NC_000005.9 (138723156..138725602, complement)

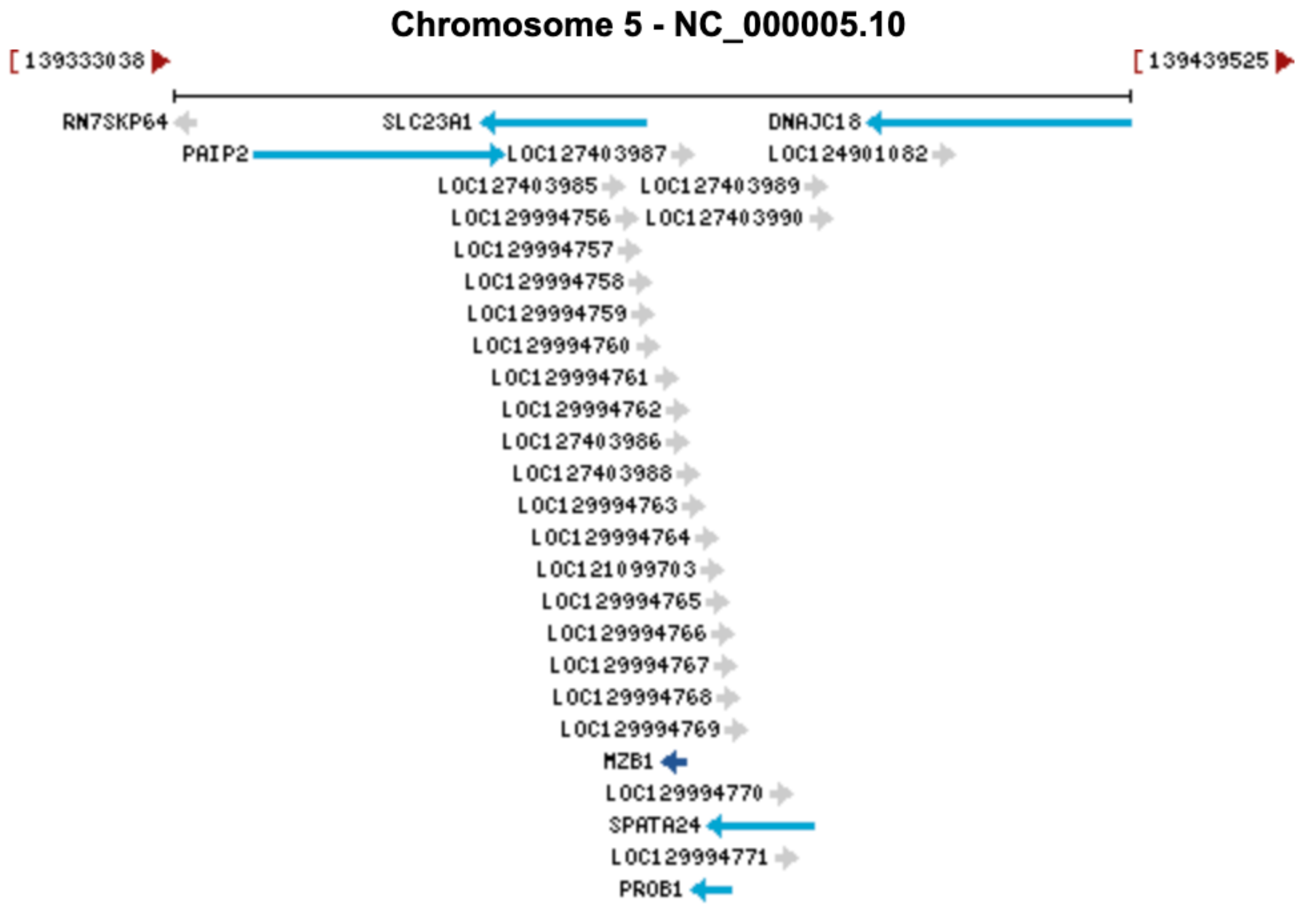

1，查找人源Mzb1基因组信息，获取基因所在的染色体及Location，mzb1在基因组中的位置为：Chr5:139387467–139389913，且在互补链（也叫反义链）上，即转录方向为反向（从下方图谱中的箭头也可看出来，转录方向是从右往左）

Figure 8A

Homo sapiens chromosome 5, GRCh38.p14 Primary Assembly
NCBI Reference Sequence: NC_000005.10GenBank Graphics

```
>NC_000005.10:c139391913-139389814 Homo sapiens chromosome 5, GRCh38.p14 Primary
AssemblyGGACAGGAAGTGATCTTCAGAGAATGAATCTGAGCCCCGAGGCTGGTGGAGCTGGAGTCACCCAGGAAGTGGCTGCCTACTTCCTCCCCCAACAAAATTATG
TATCCCTTTCCCAGGCATTCCCAGGGCGTGGTGTGGTGTGGTGTGTGGGTGTGTGTATATGTGTATATGCGGGCAAGCAAATAGGGAAAGTGAGGCATCCCTGGGCTTGT
CCCCAGGAGCCGATCAGAGGAGGTGCTGTGGCTGCCGTGGGGCAGCAGGAACGGGAGCAGCAGGAGACAGGGCGGGGAACTGTCTGCCCTCCCTGATGGTGGGCCCAGCT
GAGGTGAGGCTGGCCAGTGCCCCTGGTGAGGTGGCAGGAAGGACTCTGGCTGTCGAGTCTGCTCTTAGGAAGAGGAGCTGAGCTTTGGCCCTGGAGGTTACTGGGGGGAA
AGTGGCCAGTACAGCCCAGGCCAGGCTGGCTCCGAGAAAGAGCTAAGGCGGGGGACAGCAGGTGGAGGCAAAGCAAGGAATACAGGGAGGGCCTGGGTTGGGGGTTAGCC
TGGAACTTGGGGTGGGAAGATGGGGAGCCTGGCCCTCAGAGCTGAGCTGACTCATTTTTGGGCCAGGGGTGGGGCTTCAGTCCCCCACCCCCTACATCGGGAGTAGGTGA
AATGGTCATCAGGAAGCTCTGAGAAGAGGGAGGGTTGGGCTCTTGTCCCACAGGAGCCTCTTGACCCCTCTGTGTGGCCAGAAGGTGGGTTAGGGCCAGCAGTAGAAGCG
ATACCCCTGGTGGAGTATCCAGAGGGATCTGAAGCTCTTGGCAGGTTCCACTCCCCAGTAACAGATGTGGCAACAATGGCCAGAGAGGGGCTGGCCCTACAGACCAGTCA
CAGCAAGGGACAGGGCCCAGAGCCTGCACTTCAGTTGCCCTGAGATTCCAGGAGCCTCTGAGGGAGATGAGGGGGGTCTGCATGAGGTGGGAGGTGGTGTGCGTGGGGGT
CTGTATATAGTTCTGAACCGGAAATGGCTGGCCCTTGCCAGCCTGGTGTGTGATGTGATGAGTTTGGGAAGGACAGTTTCCATGAGAAGCAGTGGCAGGGATGGGGATGG
ATGCCTTCGTTGTTCCAGGCCTGTCAGGGGGCCCAGGTCCCTAGCAGTCCCTGCCCTCTGCCCTGGACTGGAAGCTGACTAGGAGAGCTACGAATCAGAACAACTGGCCA
TTTTCTAGCTGGAGATGCCAGCTCCGATGGCATGGAGGGCCCTGGGGCCACCCCAGGTCATCTTCGGGGCCCTGGAGGAGGGAGTGGGAGGAGGATGGGAGGGGTGAGGT
GTCAGTTCCCCATTGGTCTAGGCCAGGTGTTCTGTGGCGGTCCCCACACCACACAAGCACACACACATCTGCACCTCAACCACAGACTACACTTGCTGAACTGGCTCCTG
GGGCCATGAGGCTGTCACTGCCACTGCTGCTGCTGCTGCTGGGAGCCTAGGTGCCTGATTTCCTGCCACAGCCTCTCAACACCCTCCACAGTGCCCTTCCACGCCTGGGT
ATGGCCTGGGTGGAGGGGACCTCAGGGCTAGGGAAGGAGACCATGTGTGTCTTGGAAACACTGTAGCATCCCTGTCTCCCTGAACTTGCCTCCCAGGAGGCTGGCAGCCA
CACCTCATTCCTTCTGGTGGTTGAGTGGCCAGGGAGCCTTGGGGTCAATCAGGCTTGGGTTTCTCAGCATGGCCCCTCTAGCTATGTGGCCTTGGATTTGTCACCTCTCT
CTGAGCCTTTGTGTCGGAAGAGGCAGAATAAACATGACAGTCTCATTTCTCAGAAAGGTCAATGAAACAAAAAAAGATGACGGCAGCCAACCAACTCTTCCCACAAGGCA
CTCAGCACAGTGCCTGCCACTTGGCCAGATCACAGGATGTGGGCAGTATCGGCCCCTATGTGTTCCTGCCTAGGCCCAGCCCCTCTCCTTGCCTCAGTGCTCCCTATCTG
TGAAATGGAGCTGAGATGGCTGGAGCCAGGGTCCAGCTAGAGGACAATGCCCACGTGG
```

确定启动子区域获得序列：

Genome Browser 里找到并点击Track Hubs， JASPAR组件添加到UCSC基因浏览器里；在搜索框里输入基因的启动子区位置，获得预测结果，查阅文献筛选，初步确定klf4

Figure 8A

Matrix ID	Name	Score	Relative score	Sequence ID	Start	End	Strand	Predicted sequence
MA0039.5	MA0039.5.KLF4	14.5459	0.999999998	NC_000005.10:c139391913-139389814	625	632	+	CCCCACCC
MA0039.4	MA0039.4.KLF4	15.1204	0.97966713	NC_000005.10:c139391913-139389814	623	634	+	TCCCCCACCCCC
MA0039.3	MA0039.3.KLF4	12.9629	0.946162637	NC_000005.10:c139391913-139389814	1359	1369	+	CAACACCCTCC

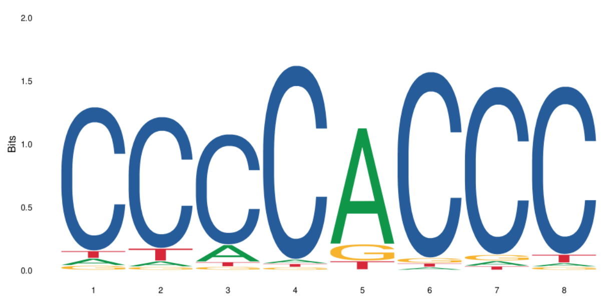

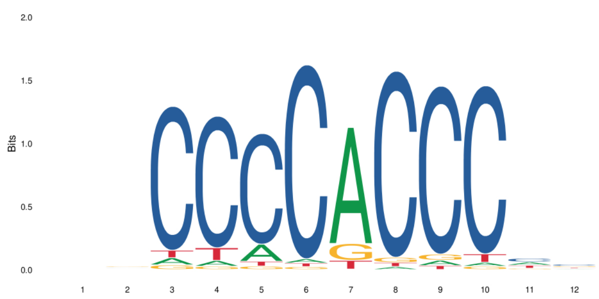

MA0039.4

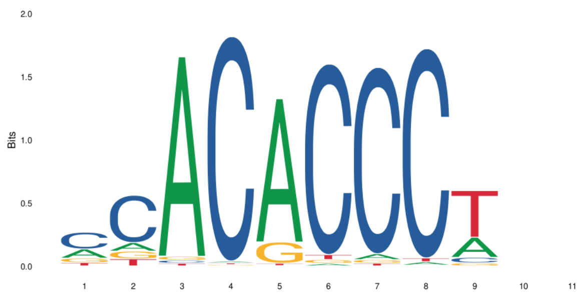

MA0039.3

MA0039.5

JASPAR主页http://jaspar.genereg.net/ ， 检索需要分析的转录因子，把前面从NCBI获得的基因启动子序列复制到“Scan”下方的文本框中，阈值设置为90%,点击“Scan”，显示预测的结合位点Analysis results
