# Supplementary material for: Puerarin attenuates myocardial ischemic injury and endoplasmic reticulum stress by upregulating the Mzb1 signal pathway
Source: Front Pharmacol. 2024 Aug 13;15:1442831. doi: 10.3389/fphar.2024.1442831 (PMC11350615; doi:10.3389/fphar.2024.1442831)
Supplement: Supplementary file 5 [file DataSheet10.zip › Figure 8/Figure 8G-H/8G-H.pdf]

Figure 8G-H

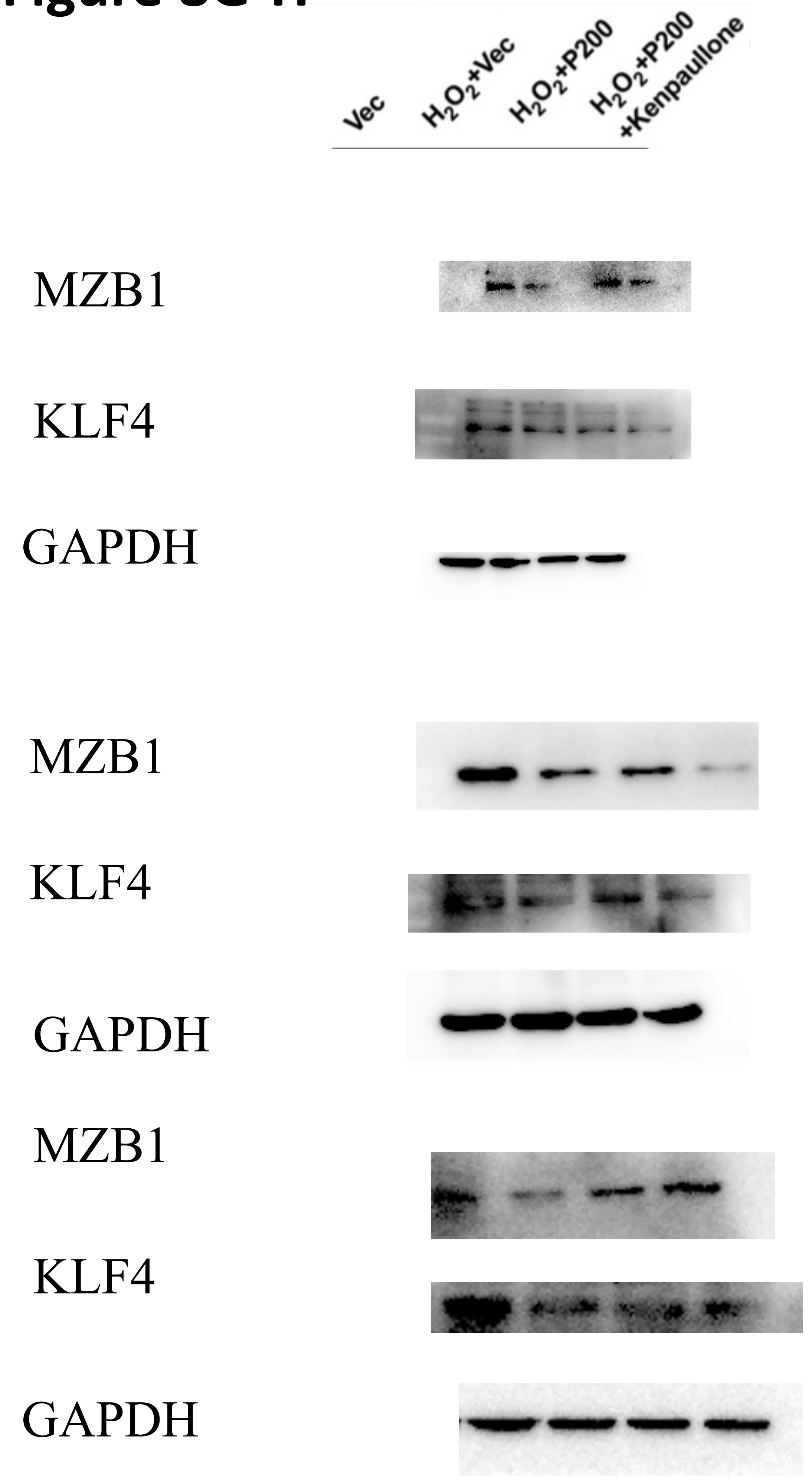

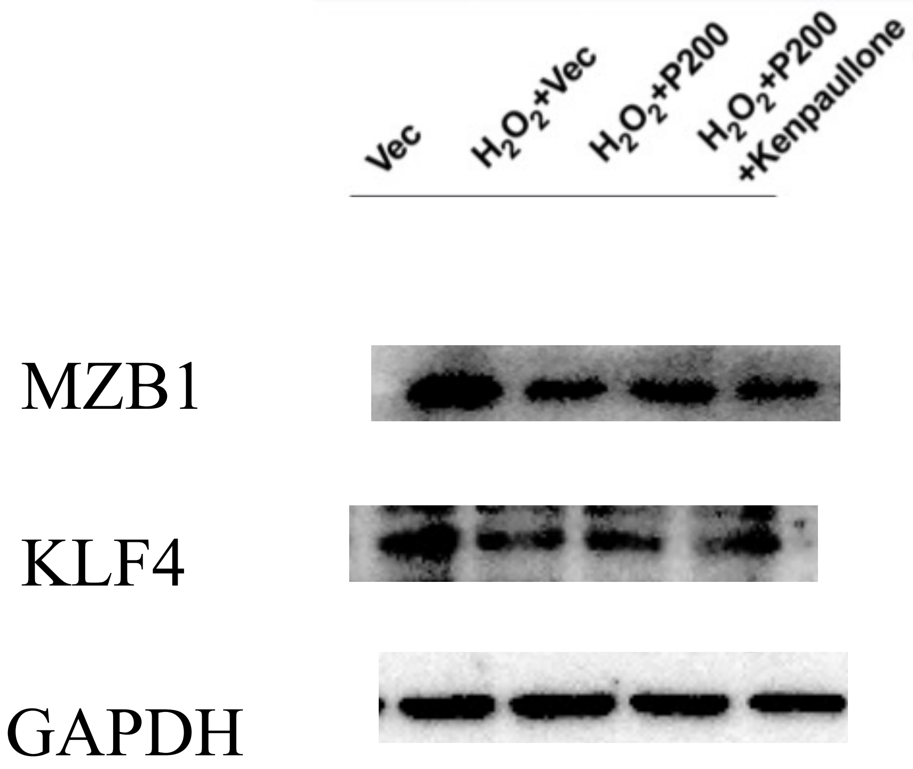

KLF4	Vec	H_2O_2+Vec	H_2O_2+P200	H_2O_2+P200 +Kenpaullone
	1	0.485	0.909	0.507
	1	0.441	0.607	0.409
	1	0.579	1.266	0.652
	1	0.52	1.021	0.694

MZB1	Vec	H_2O_2+Vec	H_2O_2+P200	H_2O_2+P200 +Kenpaullone
	1	0.456	0.674	0.373
	1	0.547	1.031	0.72
	1	0.595	0.938	0.551
	1	0.342	0.778	0.82
