# Supplementary material for: Puerarin attenuates myocardial ischemic injury and endoplasmic reticulum stress by upregulating the Mzb1 signal pathway
Source: Front Pharmacol. 2024 Aug 13;15:1442831. doi: 10.3389/fphar.2024.1442831 (PMC11350615; doi:10.3389/fphar.2024.1442831)
Supplement: Supplementary file 5 [file DataSheet10.zip › Figure 8/Figure 8K/8K.pdf]

Figure 8K

ROS	Vec	H_2O_2+Vec	H_2O_2+P200	H_2O_2+P200 +Kenpaullone
	6.8	46.6	20	37.97
	7.55	43.06	16.22	48.48
	7.45	64.41	30.3	31.25
	21.98	43.53	18.75	44.16
	5.98	60.61	28.17	35.45
	4.96	59.5	36.76	57.3
