# Supplementary material for: Puerarin attenuates myocardial ischemic injury and endoplasmic reticulum stress by upregulating the Mzb1 signal pathway
Source: Front Pharmacol. 2024 Aug 13;15:1442831. doi: 10.3389/fphar.2024.1442831 (PMC11350615; doi:10.3389/fphar.2024.1442831)
Supplement: Supplementary file 7 [file DataSheet2.zip › Figure 1E/1E data.pdf]

Figure 1E

	Sham	AMI+ Vec	AMI+ Pue50	AMI+ Pue100
TTC	0	52	32	16
	0	42	28	18
	0	46	24	13
