# Supplementary material for: Puerarin attenuates myocardial ischemic injury and endoplasmic reticulum stress by upregulating the Mzb1 signal pathway
Source: Front Pharmacol. 2024 Aug 13;15:1442831. doi: 10.3389/fphar.2024.1442831 (PMC11350615; doi:10.3389/fphar.2024.1442831)
Supplement: Supplementary file 7 [file DataSheet2.zip › Figure 1B-C/1B-C data.pdf]

Figure 1B

	Sham	AMI+ Vec	AMI+ Pue50	AMI+ Pue100
EF	78	37	40	65
	77	38	54	57
	83	38	49	61
	61	21	59	42
		39	57	83
			68	

Figure 1C

	Sham	AMI+ Vec	AMI+ Pue50	AMI+ Pue100
FS	40	21	24	31
	40	23	34	35
	46	22	37	48
	48	24	27	27
		19	35	45
			33	
