# Supplementary material for: Puerarin attenuates myocardial ischemic injury and endoplasmic reticulum stress by upregulating the Mzb1 signal pathway
Source: Front Pharmacol. 2024 Aug 13;15:1442831. doi: 10.3389/fphar.2024.1442831 (PMC11350615; doi:10.3389/fphar.2024.1442831)
Supplement: Supplementary file 7 [file DataSheet2.zip › Figure 1B-C/report/__ID_C-2__2021-12-21_09_07_04.pdf]

Patient Data

**Owner name**		**Animal name**	
**Breed**		**Neutered**	---
**Identification**	C-2	**Exam Date**	Dec/21/2021
**Report Date**	Dec/21/2021		

Cardio (Other)

Cust M-Mode

LV

LVIDd	3.6	mm	LVIDs	2.1	mm
[3.4, 3.5, 3.4, 3.7, 3.7]			[1.9, 2.3, 2.2, 2.3, 1.9]		
EF	78	%	%LV FS	40	%
SV	0.088	ml			

M-Mode

Left Ventricle

IVSd	0.61	mm	LVIDd	3.6	mm
[0.63, 0.67, 0.63, 0.47, 0.63]			[3.4, 3.5, 3.4, 3.7, 3.7]		
LVPWd	0.70	mm	IVSs	1.1	mm
[0.71, 0.75, 0.75, 0.59, 0.67]			[1.3, 1.1, 1.0, 1.2, 1.0]		
LVIDs	2.1	mm	LVPWs	1.1	mm
[1.9, 2.3, 2.2, 2.3, 1.9]			[1.1, 0.9, 1.1, 0.9, 1.1]		
EF	78	%	%LV FS	40	%
% IVS	84	%	%PW	51	%
LV Mass	-14	g			
