# Supplementary material for: Puerarin attenuates myocardial ischemic injury and endoplasmic reticulum stress by upregulating the Mzb1 signal pathway
Source: Front Pharmacol. 2024 Aug 13;15:1442831. doi: 10.3389/fphar.2024.1442831 (PMC11350615; doi:10.3389/fphar.2024.1442831)
Supplement: Supplementary file 7 [file DataSheet2.zip › Figure 1B-C/report/__ID_P100-4__2021-12-21_12_50_10.pdf]

Patient Data

**Owner name**		**Animal name**	
**Breed**		**Neutered**	---
**Identification**	P100-4	**Exam Date**	Dec/21/2021
**Report Date**	Dec/21/2021		

Cardio (Other)

Cust M-Mode

LV

LVIDd	3.6	mm	LVIDs	2.6	mm
[3.6, 3.6, 3.6]			[2.6, 2.6, 2.6]		
EF	61	%	%LV FS	48	%
SV	0.073	ml			

M-Mode

Left Ventricle

IVSd	0.61	mm	LVIDd	3.6	mm
[0.55, 0.55, 0.71]			[3.6, 3.6, 3.6]		
LVPWd	0.73	mm	IVSs	0.95	mm
[0.63, 0.79, 0.75]			[0.99, 0.83, 1.03]		
LVIDs	2.6	mm	LVPWs	1.0	mm
[2.6, 2.6, 2.6]			[0.9, 1.0, 1.2]		
EF	61	%	%LV FS	48	%
% IVS	57	%	%PW	44	%
LV Mass	-14	g			
