# Supplementary material for: Puerarin attenuates myocardial ischemic injury and endoplasmic reticulum stress by upregulating the Mzb1 signal pathway
Source: Front Pharmacol. 2024 Aug 13;15:1442831. doi: 10.3389/fphar.2024.1442831 (PMC11350615; doi:10.3389/fphar.2024.1442831)
Supplement: Supplementary file 7 [file DataSheet2.zip › Figure 1B-C/report/__ID_C-3__2021-12-21_14_55_05.pdf]

Patient Data

**Owner name**		**Animal name**	
**Breed**		**Neutered**	---
**Identification**	C-3	**Exam Date**	Dec/21/2021
**Report Date**	Dec/21/2021		

Cardio (Other)

Cust M-Mode

LV

LVIDd	3.7	mm	LVIDs	2.2	mm
[3.8, 3.8, 3.6, 3.7]			[2.3, 2.2, 2.4, 2.1]		
EF	77	%	%LV FS	40	%
SV	0.100	ml			

M-Mode

Left Ventricle

IVSd	0.61	mm	LVIDd	3.7	mm
[0.55, 0.67, 0.63, 0.59]			[3.8, 3.8, 3.6, 3.7]		
LVPWd	0.71	mm	IVSs	1.2	mm
[0.75, 0.79, 0.63, 0.67]			[1.4, 1.2, 1.1, 1.1]		
LVIDs	2.2	mm	LVPWs	1.2	mm
[2.3, 2.2, 2.4, 2.1]			[1.1, 1.4, 1.1, 1.3]		
EF	77	%	%LV FS	40	%
% IVS	97	%	%PW	72	%
LV Mass	-14	g			
