# Supplementary material for: Puerarin attenuates myocardial ischemic injury and endoplasmic reticulum stress by upregulating the Mzb1 signal pathway
Source: Front Pharmacol. 2024 Aug 13;15:1442831. doi: 10.3389/fphar.2024.1442831 (PMC11350615; doi:10.3389/fphar.2024.1442831)
Supplement: Supplementary file 7 [file DataSheet2.zip › Figure 1B-C/report/__ID_AMI-6__2021-12-24_10_09_50.pdf]

Patient Data

**Owner name**		**Animal name**	
**Breed**		**Neutered**	---
**Identification**	AMI-6	**Exam Date**	Dec/24/2021
**Report Date**	Dec/24/2021		

Cardio (Other)

Cust M-Mode

LV

LVIDd<br>[3.6, 4.0, 3.8]	3.8	mm	LVIDs<br>[2.0, 1.6, 1.9]	1.8	mm
EF	38	%	%LV FS	22	%
SV	0.120	ml			

M-Mode

Left Ventricle

IVSd<br>[0.87, 0.51, 0.79]	0.73	mm	LVIDd<br>[3.6, 4.0, 3.8]	3.8	mm
LVPWd<br>[0.83, 0.83, 1.11]	0.92	mm	IVSs<br>[1.2, 1.3, 1.5]	1.3	mm
LVIDs<br>[2.0, 1.6, 1.9]	1.8	mm	LVPWs<br>[1.5, 1.7, 1.5]	1.6	mm
EF	38	%	%LV FS	22	%
% IVS	85	%	%PW	69	%
LV Mass	-13	g			
