# Supplementary material for: Puerarin attenuates myocardial ischemic injury and endoplasmic reticulum stress by upregulating the Mzb1 signal pathway
Source: Front Pharmacol. 2024 Aug 13;15:1442831. doi: 10.3389/fphar.2024.1442831 (PMC11350615; doi:10.3389/fphar.2024.1442831)
Supplement: Supplementary file 7 [file DataSheet2.zip › Figure 1B-C/report/__ID_CTL-5__2021-12-24_11_43_40.pdf]

Patient Data

**Owner name**		**Animal name**	
**Breed**		**Neutered**	---
**Identification**	CTL-5	**Exam Date**	Dec/24/2021
**Report Date**	Dec/24/2021		

Cardio (Other)

Cust M-Mode

LV

LVIDd	2.5	mm	LVIDs	2.5	mm
[3.6, 3.5, 3.7, 1.5, 0.0]			[2.2, 2.5, 3.1, 2.4]		
EF	61	%	%LV FS	48	%
SV	0.0	ml			

M-Mode

Left Ventricle

IVSd	1.8	mm	LVIDd	2.5	mm
[1.0, 0.8, 0.9, 2.8, 3.4]			[3.6, 3.5, 3.7, 1.5, 0.0]		
LVPWd	0.64	mm	IVSs	1.1	mm
[0.51, 0.67, 0.91, 0.47]			[1.3, 1.2, 0.9]		
LVIDs	2.5	mm	LVPWs	1.1	mm
[2.2, 2.5, 3.1, 2.4]			[1.1, 1.1, 1.1]		
EF	61	%	%LV FS	48	%
% IVS	36	%	%PW	72	%
LV Mass	13	g			
