# Supplementary material for: Puerarin attenuates myocardial ischemic injury and endoplasmic reticulum stress by upregulating the Mzb1 signal pathway
Source: Front Pharmacol. 2024 Aug 13;15:1442831. doi: 10.3389/fphar.2024.1442831 (PMC11350615; doi:10.3389/fphar.2024.1442831)
Supplement: Supplementary file 7 [file DataSheet2.zip › Figure 1B-C/report/__ID_AMI-3__2021-12-21_09_31_01.pdf]

Patient Data

**Owner name**		**Animal name**	
**Breed**		**Neutered**	---
**Identification**	AMI-3	**Exam Date**	Dec/21/2021
**Report Date**	Dec/21/2021		

Cardio (Other)

Cust M-Mode

LV

LVIDd	3.9	mm	LVIDs	2.6	mm
[4.2, 3.8, 3.9, 3.6, 3.8]			[3.3, 2.7, 2.5, 2.4, 2.2]		
EF	38	%	%LV FS	23	%
SV	0.099	ml			

M-Mode

Left Ventricle

IVSd	0.63	mm	LVIDd	3.9	mm
[0.71, 0.47, 0.63, 0.67, 0.67]			[4.2, 3.8, 3.9, 3.6, 3.8]		
LVPWd	0.61	mm	IVSs	1.1	mm
[0.67, 0.44, 0.79, 0.63, 0.51]			[1.1, 1.1, 1.0, 1.0, 1.3]		
LVIDs	2.6	mm	LVPWs	0.95	mm
[3.3, 2.7, 2.5, 2.4, 2.2]			[0.99, 0.63, 1.03, 1.03, 1.07]		
EF	38	%	%LV FS	23	%
% IVS	75	%	%PW	56	%
LV Mass	-14	g			
