# Supplementary material for: Puerarin attenuates myocardial ischemic injury and endoplasmic reticulum stress by upregulating the Mzb1 signal pathway
Source: Front Pharmacol. 2024 Aug 13;15:1442831. doi: 10.3389/fphar.2024.1442831 (PMC11350615; doi:10.3389/fphar.2024.1442831)
Supplement: Supplementary file 7 [file DataSheet2.zip › Figure 1B-C/report/__ID_AMI-8__2021-12-24_11_05_39.pdf]

Patient Data

**Owner name**		**Animal name**	
**Breed**		**Neutered**	---
**Identification**	AMI-8	**Exam Date**	Dec/24/2021
**Report Date**	Dec/24/2021		

Cardio (Other)

Cust M-Mode

LV

LVIDd	3.8	mm	LVIDs	2.8	mm
[3.8, 3.7, 3.7, 4.0, 3.8]			[2.8, 2.7, 2.5, 3.1, 2.8]		
EF	39	%	%LV FS	19	%
SV	0.081	ml			

M-Mode

Left Ventricle

IVSd	0.72	mm	LVIDd	3.8	mm
[0.75, 0.63, 0.71, 0.83, 0.67]			[3.8, 3.7, 3.7, 4.0, 3.8]		
LVPWd	0.72	mm	IVSs	1.0	mm
[0.71, 0.79, 0.59, 0.79, 0.71]			[0.9, 1.0, 1.1, 1.0, 1.1]		
LVIDs	2.8	mm	LVPWs	1.0	mm
[2.8, 2.7, 2.5, 3.1, 2.8]			[1.1, 0.9, 1.0, 1.0, 1.1]		
EF	39	%	%LV FS	19	%
% IVS	42	%	%PW	40	%
LV Mass	-14	g			
