# Supplementary material for: Puerarin attenuates myocardial ischemic injury and endoplasmic reticulum stress by upregulating the Mzb1 signal pathway
Source: Front Pharmacol. 2024 Aug 13;15:1442831. doi: 10.3389/fphar.2024.1442831 (PMC11350615; doi:10.3389/fphar.2024.1442831)
Supplement: Supplementary file 7 [file DataSheet2.zip › Figure 1B-C/report/__ID_P50-4__2021-12-21_14_32_19.pdf]

Patient Data

**Owner name**		**Animal name**	
**Breed**		**Neutered**	---
**Identification**	P50-4	**Exam Date**	Dec/21/2021
**Report Date**	Dec/21/2021		

Cardio (Other)

Cust M-Mode

LV

LVIDd	3.8	mm	LVIDs	2.9	mm
[3.3, 3.8, 4.2, 4.0]			[2.7, 2.9, 3.1]		
EF	54	%	%LV FS	34	%
SV	0.077	ml			

M-Mode

Left Ventricle

IVSd	0.74	mm	LVIDd	3.8	mm
[0.75, 0.67, 0.79, 0.75]			[3.3, 3.8, 4.2, 4.0]		
LVPWd	0.75	mm	IVSs	0.99	mm
[0.75, 0.59, 0.71, 0.95]			[0.95, 0.99, 0.95, 1.07]		
LVIDs	2.9	mm	LVPWs	0.96	mm
[2.7, 2.9, 3.1]			[0.87, 0.83, 1.19]		
EF	54	%	%LV FS	34	%
% IVS	33	%	%PW	28	%
LV Mass	-14	g			
