# Supplementary material for: Puerarin attenuates myocardial ischemic injury and endoplasmic reticulum stress by upregulating the Mzb1 signal pathway
Source: Front Pharmacol. 2024 Aug 13;15:1442831. doi: 10.3389/fphar.2024.1442831 (PMC11350615; doi:10.3389/fphar.2024.1442831)
Supplement: Supplementary file 7 [file DataSheet2.zip › Figure 1B-C/report/__ID_CTL-4__2021-12-24_11_15_12.pdf]

Patient Data

**Owner name**		**Animal name**	
**Breed**		**Neutered**	---
**Identification**	CTL-4	**Exam Date**	Dec/24/2021
**Report Date**	Dec/24/2021		

Cardio (Other)

Cust M-Mode

LV

LVIDd	3.0	mm	LVIDs	1.6	mm
[3.1, 3.2, 2.9, 2.8]			[1.8, 1.6, 1.7, 1.4]		
EF	83	%	%LV FS	46	%
SV	0.059	ml			

M-Mode

Left Ventricle

IVSd	0.74	mm	LVIDd	3.0	mm
[0.59, 0.63, 0.91, 0.83]			[3.1, 3.2, 2.9, 2.8]		
LVPWd	0.91	mm	IVSs	1.3	mm
[1.07, 0.75, 0.67, 1.15]			[1.4, 1.3, 1.3, 1.0]		
LVIDs	1.6	mm	LVPWs	1.4	mm
[1.8, 1.6, 1.7, 1.4]			[1.2, 1.4, 1.5, 1.7]		
EF	83	%	%LV FS	46	%
% IVS	69	%	%PW	59	%
LV Mass	-14	g			
