# Supplementary material for: Puerarin attenuates myocardial ischemic injury and endoplasmic reticulum stress by upregulating the Mzb1 signal pathway
Source: Front Pharmacol. 2024 Aug 13;15:1442831. doi: 10.3389/fphar.2024.1442831 (PMC11350615; doi:10.3389/fphar.2024.1442831)
Supplement: Supplementary file 7 [file DataSheet2.zip › Figure 1B-C/report/__ID_P50-6__2022-01-09_08_29_51.pdf]

Patient Data

**Owner name**		**Animal name**	P50-6
**Breed**		**Neutered**	---

**Exam Date**	09/01/2022
**Report Date**	09/01/2022

Cardio (Other)

M-Mode

Left Ventricle

IVSd	0.75	mm	LVIDd	3.6	mm
LVPWd	0.53	mm	IVSs	1.2	mm
LVIDs	2.9	mm	LVPWs	0.96	mm
EF	49	%	%LV FS	37	%
HR	286	bpm	HR (ECG)	286	bpm
% IVS	57	%	%PW	80	%
