# Supplementary material for: Puerarin attenuates myocardial ischemic injury and endoplasmic reticulum stress by upregulating the Mzb1 signal pathway
Source: Front Pharmacol. 2024 Aug 13;15:1442831. doi: 10.3389/fphar.2024.1442831 (PMC11350615; doi:10.3389/fphar.2024.1442831)
Supplement: Supplementary file 7 [file DataSheet2.zip › Figure 1B-C/report/__ID_P50-1__2021-12-21_11_28_05.pdf]

Patient Data

**Owner name**		**Animal name**	
**Breed**		**Neutered**	---
**Identification**	P50-1	**Exam Date**	Dec/21/2021
**Report Date**	Dec/21/2021		

Cardio (Other)

Cust M-Mode

LV

LVIDd<br>[3.3, 3.1, 3.2]	3.2	mm	LVIDs<br>[2.5, 1.9, 2.0]	2.1	mm
EF	40	%	%LV FS	24	%
SV	0.061	ml			

M-Mode

Left Ventricle

IVSd<br>[0.59, 0.67, 0.67]	0.65	mm	LVIDd<br>[3.3, 3.1, 3.2]	3.2	mm
LVPWd<br>[0.79, 0.75, 0.71]	0.75	mm	IVSs<br>[0.9, 1.1, 1.2]	1.1	mm
LVIDs<br>[2.5, 1.9, 2.0]	2.1	mm	LVPWs<br>[1.1, 1.1, 1.1]	1.1	mm
EF	40	%	%LV FS	24	%
% IVS	65	%	%PW	47	%
LV Mass	-14	g			
