# Supplementary material for: Puerarin attenuates myocardial ischemic injury and endoplasmic reticulum stress by upregulating the Mzb1 signal pathway
Source: Front Pharmacol. 2024 Aug 13;15:1442831. doi: 10.3389/fphar.2024.1442831 (PMC11350615; doi:10.3389/fphar.2024.1442831)
Supplement: Supplementary file 7 [file DataSheet2.zip › Figure 1B-C/report/__ID_P50-8__2022-01-09_07_08_20.pdf]

Patient Data

**Owner name**		**Animal name**	P50-8
**Breed**		**Neutered**	---

**Exam Date**	09/01/2022
**Report Date**	09/01/2022

Cardio (Other)

M-Mode

Left Ventricle

IVSd	0.96	mm	LVIDd	3.4	mm
LVPWd	0.64	mm	IVSs	1.1	mm
LVIDs	2.5	mm	LVPWs	1.2	mm
EF	57	%	%LV FS	35	%
% IVS	11	%	%PW	83	%
LV Mass	-14	g			
