# Supplementary material for: Puerarin attenuates myocardial ischemic injury and endoplasmic reticulum stress by upregulating the Mzb1 signal pathway
Source: Front Pharmacol. 2024 Aug 13;15:1442831. doi: 10.3389/fphar.2024.1442831 (PMC11350615; doi:10.3389/fphar.2024.1442831)
Supplement: Supplementary file 7 [file DataSheet2.zip › Figure 1B-C/report/__ID_P100-8__2022-01-09_07_27_17.pdf]

Patient Data

**Owner name**		**Animal name**	P100-8
**Breed**		**Neutered**	---

**Exam Date**	09/01/2022
**Report Date**	09/01/2022

Cardio (Other)

M-Mode

Left Ventricle

IVSd	1.9	mm	LVIDd	2.3	mm
LVPWd	0.85	mm	IVSs	2.3	mm
LVIDs	1.0	mm	LVPWs	1.5	mm
EF	42	%	%LV FS	27	%
HR	566	bpm	HR (ECG)	566	bpm
% IVS	23	%	%PW	81	%
