# Supplementary material for: Puerarin attenuates myocardial ischemic injury and endoplasmic reticulum stress by upregulating the Mzb1 signal pathway
Source: Front Pharmacol. 2024 Aug 13;15:1442831. doi: 10.3389/fphar.2024.1442831 (PMC11350615; doi:10.3389/fphar.2024.1442831)
Supplement: Supplementary file 7 [file DataSheet2.zip › Figure 1B-C/report/__ID_P100-3__2021-12-21_13_31_12.pdf]

# Patient Data

| | | | |
|---|---|---|---|
| **Owner name** | | **Animal name** | |
| **Breed** | | **Neutered** | --- |
| **Identification** | P100-3 | **Exam Date** | Dec/21/2021 |
| **Report Date** | Dec/21/2021 | | |

# Cardio (Other)

## Cust M-Mode

***LV***

| | | | | | |
|---|---|---|---|---|---|
| LVIDd | 3.9 | mm | LVIDs | 2.9 | mm |
| [3.9, 3.8, 3.9, 4.0, 4.0] | | | [2.9, 2.8, 3.0, 3.1, 2.7] | | |
| EF | 57 | % | %LV FS | 35 | % |
| SV | 0.086 | ml | | | |

## M-Mode

**Left Ventricle**

| | | | | | |
|---|---|---|---|---|---|
| IVSd | 0.74 | mm | LVIDd | 3.9 | mm |
| [0.71, 0.87, 0.83, 0.67, 0.63] | | | [3.9, 3.8, 3.9, 4.0, 4.0] | | |
| LVPWd | 0.75 | mm | IVSs | 1.1 | mm |
| [0.79, 0.83, 0.59, 0.75, 0.79] | | | [1.1, 1.1, 1.0, 1.0, 1.2] | | |
| LVIDs | 2.9 | mm | LVPWs | 1.1 | mm |
| [2.9, 2.8, 3.0, 3.1, 2.7] | | | [1.1, 1.1, 0.9, 0.9, 1.2] | | |
| EF | 57 | % | %LV FS | 35 | % |
| % IVS | 46 | % | %PW | 40 | % |
| LV Mass | -13 | g | | | |
