# Supplementary material for: Puerarin attenuates myocardial ischemic injury and endoplasmic reticulum stress by upregulating the Mzb1 signal pathway
Source: Front Pharmacol. 2024 Aug 13;15:1442831. doi: 10.3389/fphar.2024.1442831 (PMC11350615; doi:10.3389/fphar.2024.1442831)
Supplement: Supplementary file 7 [file DataSheet2.zip › Figure 1B-C/report/__ID_AMI-1__2021-12-21_10_16_54.pdf]

Patient Data

**Owner name**		**Animal name**	
**Breed**		**Neutered**	---
**Identification**	AMI-1	**Exam Date**	Dec/21/2021
**Report Date**	Dec/21/2021		

Cardio (Other)

Cust M-Mode

LV

LVIDd	3.4	mm	LVIDs	1.7	mm
[3.8, 3.3, 3.0, 3.2, 3.8]			[1.6, 1.5, 1.3, 1.3, 2.8]		
EF	37	%	%LV FS	21	%
SV	0.091	ml			

M-Mode

Left Ventricle

IVSd	0.79	mm	LVIDd	3.4	mm
[0.63, 0.91, 0.75, 0.99, 0.67]			[3.8, 3.3, 3.0, 3.2, 3.8]		
LVPWd	0.74	mm	IVSs	1.3	mm
[0.67, 0.83, 0.75, 0.79, 0.63]			[1.3, 1.4, 1.4, 1.5, 1.1]		
LVIDs	1.7	mm	LVPWs	1.4	mm
[1.6, 1.5, 1.3, 1.3, 2.8]			[1.5, 1.6, 1.6, 1.4, 0.9]		
EF	37	%	%LV FS	21	%
% IVS	70	%	%PW	90	%
LV Mass	-14	g			
