# Supplementary material for: Puerarin attenuates myocardial ischemic injury and endoplasmic reticulum stress by upregulating the Mzb1 signal pathway
Source: Front Pharmacol. 2024 Aug 13;15:1442831. doi: 10.3389/fphar.2024.1442831 (PMC11350615; doi:10.3389/fphar.2024.1442831)
Supplement: Supplementary file 7 [file DataSheet2.zip › Figure 1B-C/report/__ID_AMI-7__2021-12-24_10_41_44.pdf]

Patient Data

**Owner name**		**Animal name**	
**Breed**		**Neutered**	---
**Identification**	AMI-7	**Exam Date**	Dec/24/2021
**Report Date**	Dec/24/2021		

Cardio (Other)

Cust M-Mode

LV

LVIDd	3.6	mm	LVIDs	2.0	mm
[3.6, 3.7, 3.6, 3.4]			[1.9, 2.0, 2.0, 2.2]		
EF	21	%	%LV FS	24	%
SV	0.095	ml			

M-Mode

Left Ventricle

IVSd	0.82	mm	LVIDd	3.6	mm
[0.87, 0.79, 0.71, 0.91]			[3.6, 3.7, 3.6, 3.4]		
LVPWd	0.92	mm	IVSs	1.5	mm
[0.99, 0.79, 1.03, 0.87]			[1.5, 1.5, 1.4, 1.3]		
LVIDs	2.0	mm	LVPWs	1.2	mm
[1.9, 2.0, 2.0, 2.2]			[1.3, 1.3, 1.3, 1.0]		
EF	21	%	%LV FS	24	%
% IVS	78	%	%PW	33	%
LV Mass	-13	g			
