# Supplementary material for: Puerarin attenuates myocardial ischemic injury and endoplasmic reticulum stress by upregulating the Mzb1 signal pathway
Source: Front Pharmacol. 2024 Aug 13;15:1442831. doi: 10.3389/fphar.2024.1442831 (PMC11350615; doi:10.3389/fphar.2024.1442831)
Supplement: Supplementary file 7 [file DataSheet2.zip › Figure 1B-C/report/__ID_P50-7__2021-12-24_10_24_09.pdf]

Patient Data

**Owner name**		**Animal name**	
**Breed**		**Neutered**	---
**Identification**	P50-7	**Exam Date**	Dec/24/2021
**Report Date**	Dec/24/2021		

Cardio (Other)

Cust M-Mode

LV

LVIDd	3.0	mm	LVIDs	2.2	mm
[3.1, 3.0, 2.9]			[1.8, 2.5, 2.3]		
EF	59	%	%LV FS	27	%
SV	0.042	ml			

M-Mode

Left Ventricle

IVSd	1.0	mm	LVIDd	3.0	mm
[0.9, 0.9, 1.1]			[3.1, 3.0, 2.9]		
LVPWd	0.71	mm	IVSs	1.3	mm
[0.63, 0.71, 0.79]			[1.5, 1.1, 1.2]		
LVIDs	2.2	mm	LVPWs	0.95	mm
[1.8, 2.5, 2.3]			[0.95, 0.95, 0.95]		
EF	59	%	%LV FS	27	%
% IVS	28	%	%PW	33	%
LV Mass	-14	g			
