# Supplementary material for: Puerarin attenuates myocardial ischemic injury and endoplasmic reticulum stress by upregulating the Mzb1 signal pathway
Source: Front Pharmacol. 2024 Aug 13;15:1442831. doi: 10.3389/fphar.2024.1442831 (PMC11350615; doi:10.3389/fphar.2024.1442831)
Supplement: Supplementary file 7 [file DataSheet2.zip › Figure 1B-C/report/__ID_P100-2__2021-12-21_11_05_35.pdf]

Patient Data

**Owner name**		**Animal name**	
**Breed**		**Neutered**	---
**Identification**	P100-2	**Exam Date**	Dec/21/2021
**Report Date**	Dec/21/2021		

Cardio (Other)

Cust M-Mode

LV

LVIDd	3.9	mm	LVIDs	2.7	mm
[3.8, 4.1, 3.8]			[2.7, 3.0, 2.4]		
EF	65	%	%LV FS	31	%
SV	0.097	ml			

M-Mode

Left Ventricle

IVSd	0.69	mm	LVIDd	3.9	mm
[0.83, 0.63, 0.59]			[3.8, 4.1, 3.8]		
LVPWd	0.78	mm	IVSs	1.1	mm
[0.75, 0.83, 0.75]			[1.2, 1.1, 1.1]		
LVIDs	2.7	mm	LVPWs	1.1	mm
[2.7, 3.0, 2.4]			[1.0, 1.2, 1.1]		
EF	65	%	%LV FS	31	%
% IVS	67	%	%PW	41	%
LV Mass	-14	g			
