# Supplementary material for: Puerarin attenuates myocardial ischemic injury and endoplasmic reticulum stress by upregulating the Mzb1 signal pathway
Source: Front Pharmacol. 2024 Aug 13;15:1442831. doi: 10.3389/fphar.2024.1442831 (PMC11350615; doi:10.3389/fphar.2024.1442831)
Supplement: Supplementary file 8 [file DataSheet5.zip › Figure 3/Figure 3B/3B data.pdf]

Figure 3B

	Sham	AMI+ Vec	AMI+ Pue50	AMI+ Pue100
TUNEL	6.923	9.576	8.439	9.933
	6.094	24.014	11.475	15.751
	8.988	48.751	12.129	11.531
	9.908	31.502	12.383	9.145
	10.501	10.917	13.659	5.052
	9.07	23.501	9.493	9.82
