# Supplementary material for: Puerarin attenuates myocardial ischemic injury and endoplasmic reticulum stress by upregulating the Mzb1 signal pathway
Source: Front Pharmacol. 2024 Aug 13;15:1442831. doi: 10.3389/fphar.2024.1442831 (PMC11350615; doi:10.3389/fphar.2024.1442831)
Supplement: Supplementary file 9 [file DataSheet7.zip › Figure 5/Figure 5I/5I.pdf]

Figure 5I

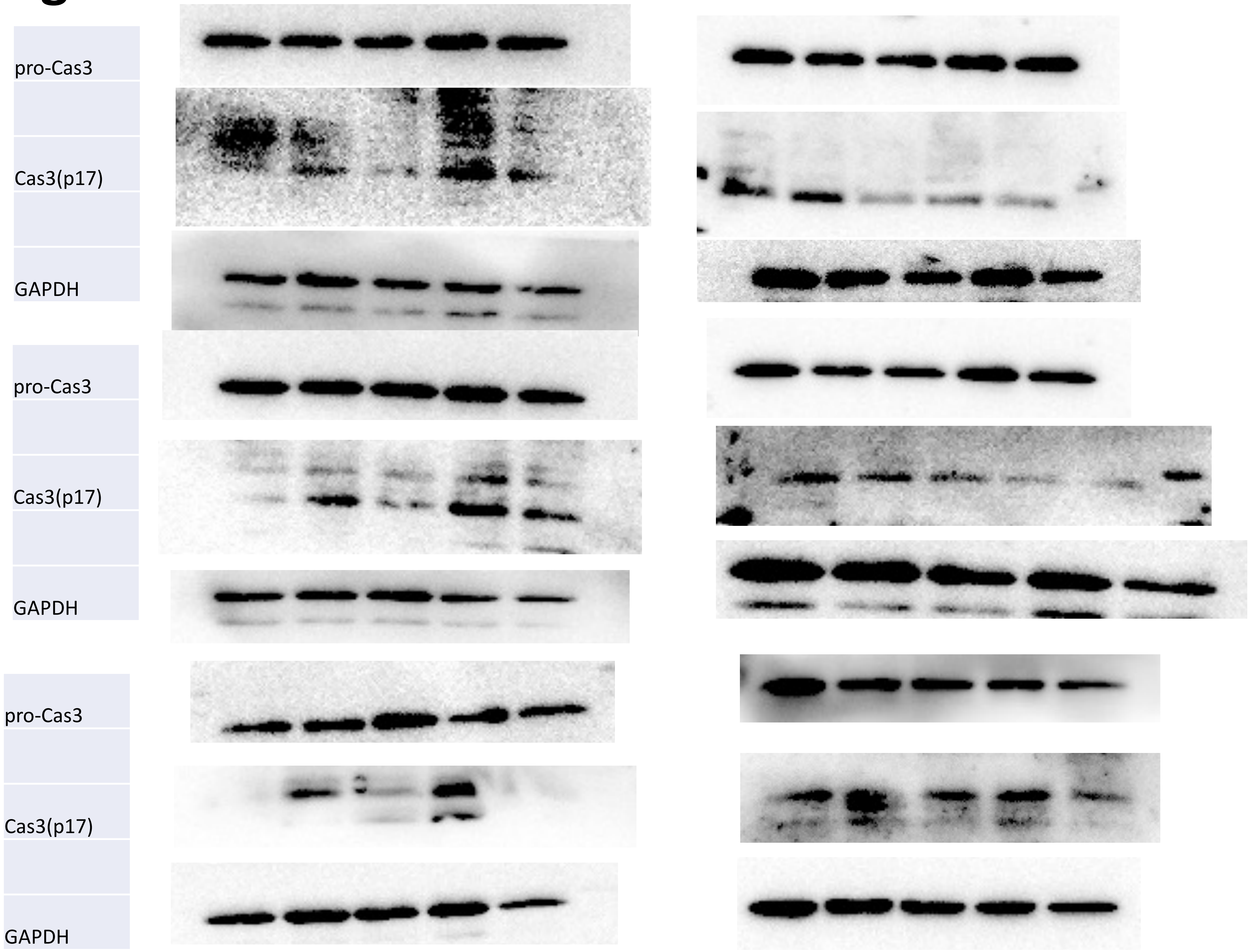

	Vec	H_2O_2+Vec	H_2O_2+P200	H_2O_2+P200 +si-Mzb1	H_2O_2+P200 +si-NC
Cleave/Pro cas3	1	2.201404456	1.05776314	1.974783734	1.267920455
	1	4.326059513	2.484058695	4.043867724	3.073054376
	1	5.063903628	2.000457791	5.321569021	1.592326413
	1	2.036702065	1.774826537	2.260766708	0.829818649
	1	2.730919862	1.223895842	2.502008475	1.23615893
	1	4.51900002	3.080540508	2.998966939	2.676997125
