# Supplementary material for: Puerarin attenuates myocardial ischemic injury and endoplasmic reticulum stress by upregulating the Mzb1 signal pathway
Source: Front Pharmacol. 2024 Aug 13;15:1442831. doi: 10.3389/fphar.2024.1442831 (PMC11350615; doi:10.3389/fphar.2024.1442831)
Supplement: Supplementary file 9 [file DataSheet7.zip › Figure 5/Figure 5F/5F.pdf]

Figure 5F

	Vec	H_2O_2+Vec	H_2O_2+P200	H_2O_2+P200 +si-Mzb1	H_2O_2+P200 +si-NC
	109.36	51.46	46.2	46.2	47.37
MTT	113.45	50.29	49.12	74.27	56.73
	87.13	64.33	214.62	62.57	184.21
	53.8	54.39	156.73	65.5	56.14
	62.57	53.8	147.95	56.73	45.61
	106.43	48.54	53.8	70.18	60.82
	76.02	39.18	64.91	97.66	62.57
	109.36	64.91	229.24	77.19	309.36
	218.13	57.89	342.11	57.89	238.01
	63.74	57.89	60.23	61.99	256.73
