# Supplementary material for: Puerarin attenuates myocardial ischemic injury and endoplasmic reticulum stress by upregulating the Mzb1 signal pathway
Source: Front Pharmacol. 2024 Aug 13;15:1442831. doi: 10.3389/fphar.2024.1442831 (PMC11350615; doi:10.3389/fphar.2024.1442831)
Supplement: Supplementary file 9 [file DataSheet7.zip › Figure 5/Figure 5H/5H.pdf]

Figure 5H

	Vec	H_2O_2+Vec	H_2O_2+P200	H_2O_2+P200 +si-Mzb1	H_2O_2+P200 +si-NC
TUNEL	2	79	18	86	22
	1	86	30	74	22
	6	85	12	68	18
	1	83	23	60	14
	3	61	2	71	11
	4	39	6	82	9
