# Supplementary material for: Puerarin attenuates myocardial ischemic injury and endoplasmic reticulum stress by upregulating the Mzb1 signal pathway
Source: Front Pharmacol. 2024 Aug 13;15:1442831. doi: 10.3389/fphar.2024.1442831 (PMC11350615; doi:10.3389/fphar.2024.1442831)
Supplement: Supplementary file 9 [file DataSheet7.zip › Figure 5/Figure 5C/5C.pdf]

Figure 5C

PCR	Control	si-NC	si-Mzb1
	0.0896615	1.70011521	0.04255972
	1.41483372	0.84711663	0.0866073
	1.49550477	0.90164568	0.07182513
	1.44677967	0.99505297	0.05193479
	0.95121623	0.71096243	0.11327059
	0.60200411	0.50974415	0.01460742
