# Supplementary material for: Puerarin attenuates myocardial ischemic injury and endoplasmic reticulum stress by upregulating the Mzb1 signal pathway
Source: Front Pharmacol. 2024 Aug 13;15:1442831. doi: 10.3389/fphar.2024.1442831 (PMC11350615; doi:10.3389/fphar.2024.1442831)
Supplement: Supplementary file 9 [file DataSheet7.zip › Figure 5/Figure 5D/5D.pdf]

Figure 5D

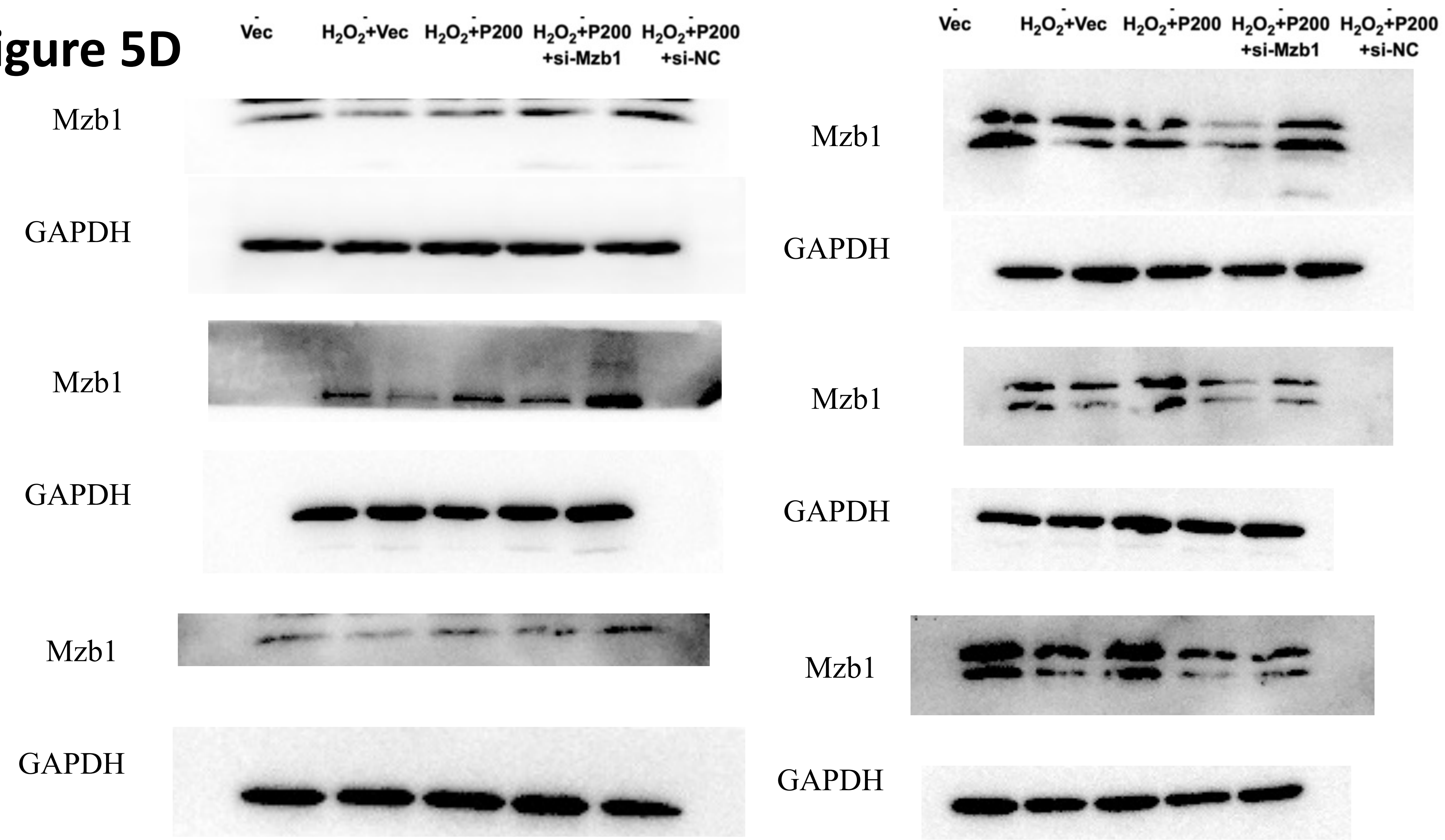

Mzb1	Vec	H_2O_2+Vec	H_2O_2+P200	H_2O_2+P200 +si-Mzb1	H_2O_2+P200 +si-NC
	1	0.411743492	0.952602735	0.463487003	1.187703683
	1	0.397430432	1.395844423	0.345985645	1.088385609
	1	0.482441291	1.236594047	0.506126156	1.485860383
	1	0.336083229	0.762435039	0.338928723	1.136072008
	1	0.473213945	1.144030115	0.23312194	0.925713834
	1	0.327770963	1.03693309	0.290892851	0.982259223
