# Supplementary material for: Puerarin attenuates myocardial ischemic injury and endoplasmic reticulum stress by upregulating the Mzb1 signal pathway
Source: Front Pharmacol. 2024 Aug 13;15:1442831. doi: 10.3389/fphar.2024.1442831 (PMC11350615; doi:10.3389/fphar.2024.1442831)
Supplement: Supplementary file 9 [file DataSheet7.zip › Figure 5/Figure 5E/5E.pdf]

Figure 5E

	NC	si-Mzb1	NC	si-Mzb1
mtt	77		14.07	7.3
	112.31	76.22	30.1	8.21
	106.06	68.53	34.66	8.6
	108.93	88.34	26.71	8.21
	105.02	82.35	25.93	8.6
	90.68	97.2	32.57	7.82
