# Supplementary figures and images for: Puerarin attenuates myocardial ischemic injury and endoplasmic reticulum stress by upregulating the Mzb1 signal pathway (part 2 of 2)
Source: Front Pharmacol. 2024 Aug 13;15:1442831. doi: 10.3389/fphar.2024.1442831 (PMC11350615; doi:10.3389/fphar.2024.1442831)

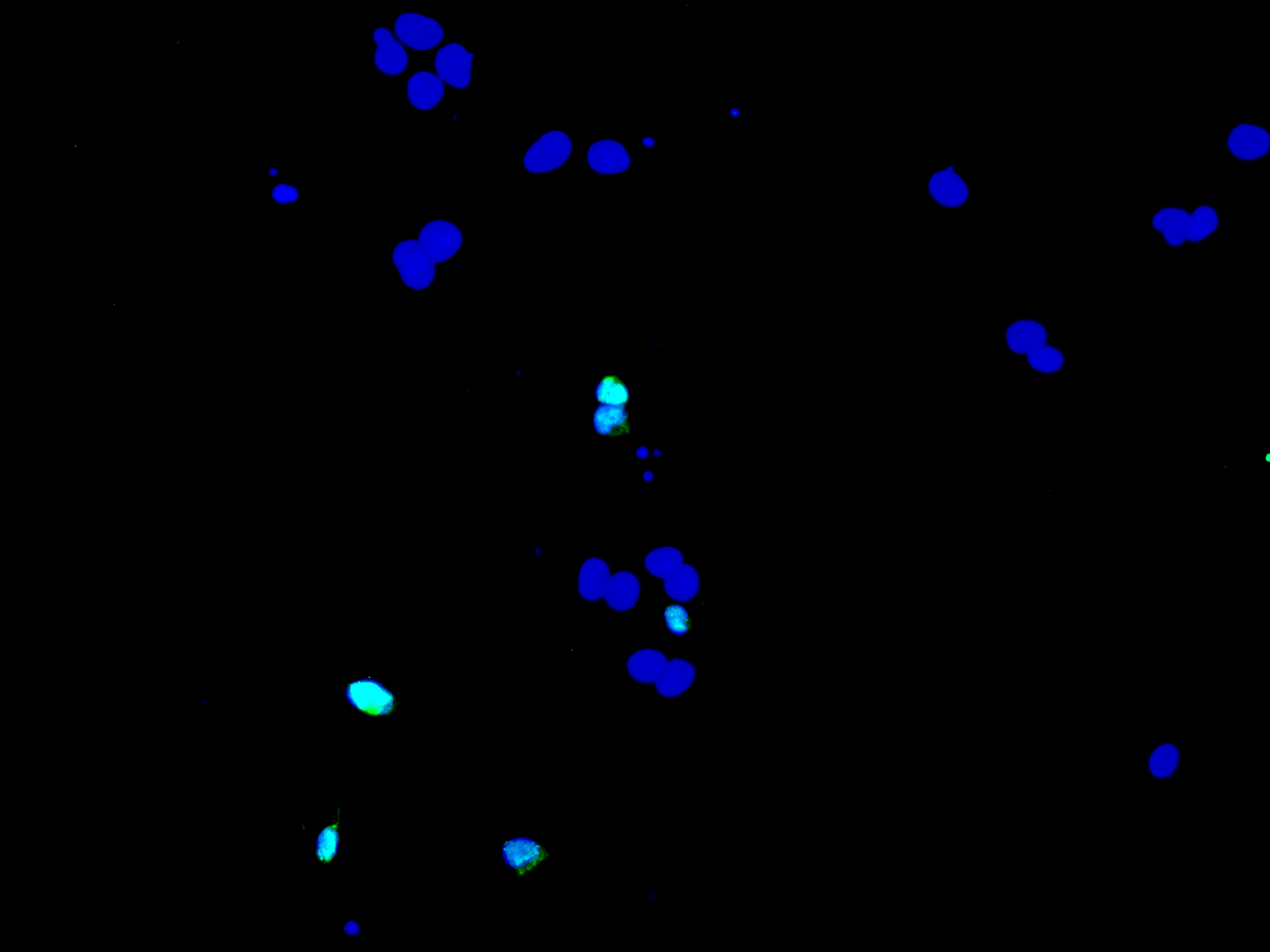

Supplement: Supplementary file 9 [file DataSheet7.zip › Figure 5/Figure 5G/h+p200_15.tif]

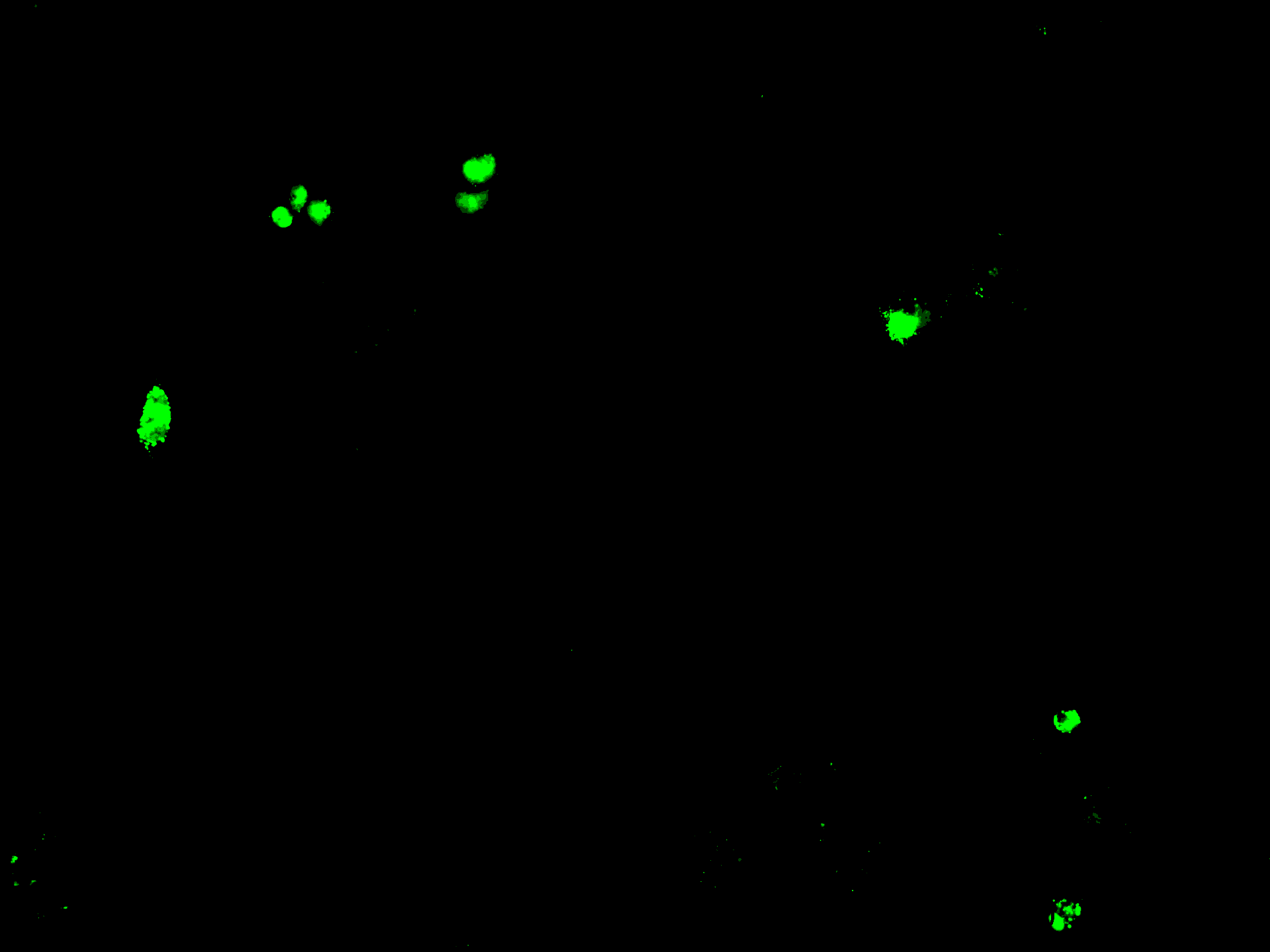

Supplement: Supplementary file 9 [file DataSheet7.zip › Figure 5/Figure 5G/h+p200+si-nc_3-2.tif]

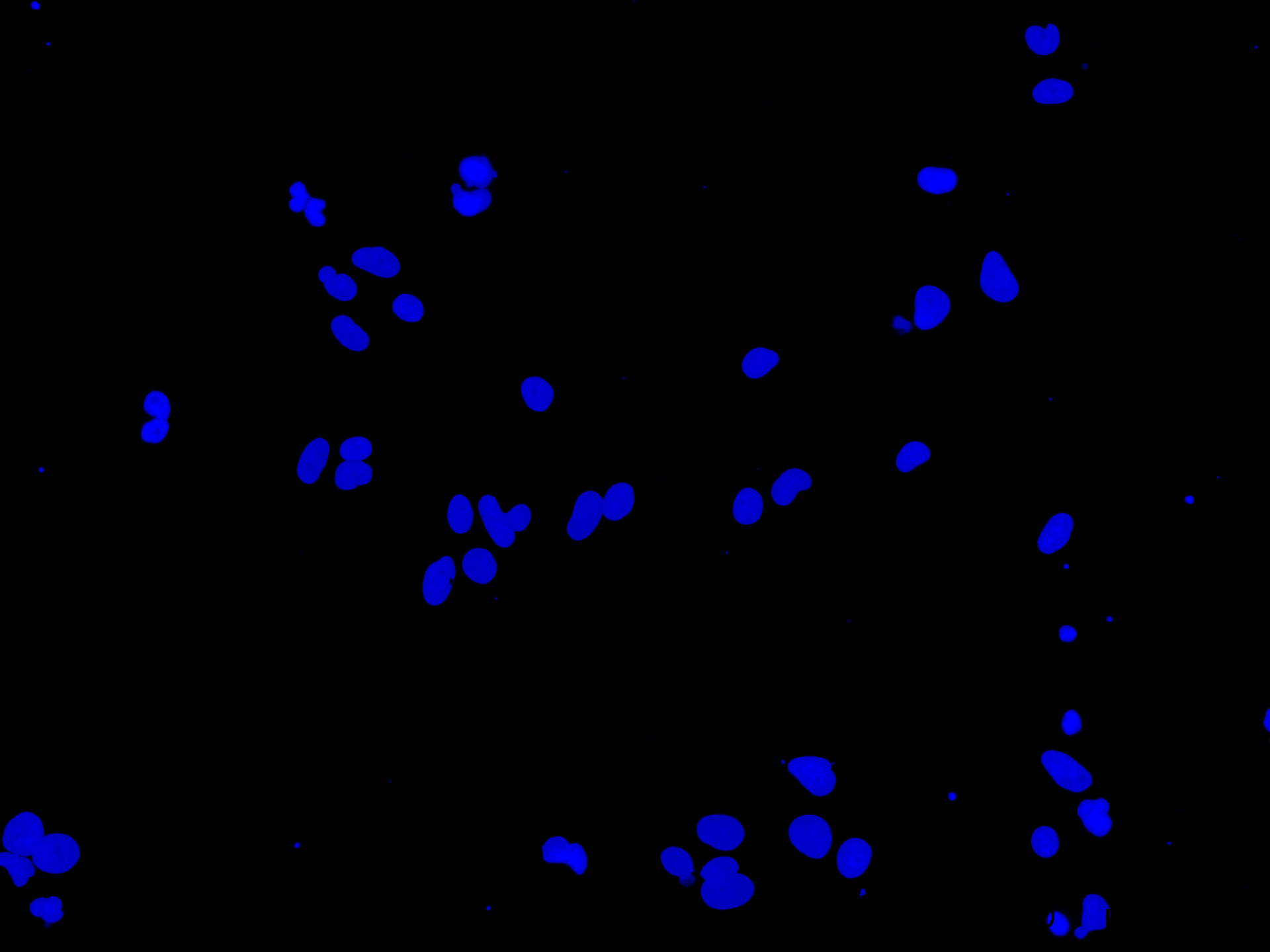

Supplement: Supplementary file 9 [file DataSheet7.zip › Figure 5/Figure 5G/h+p200+si-nc_3-1.tif]

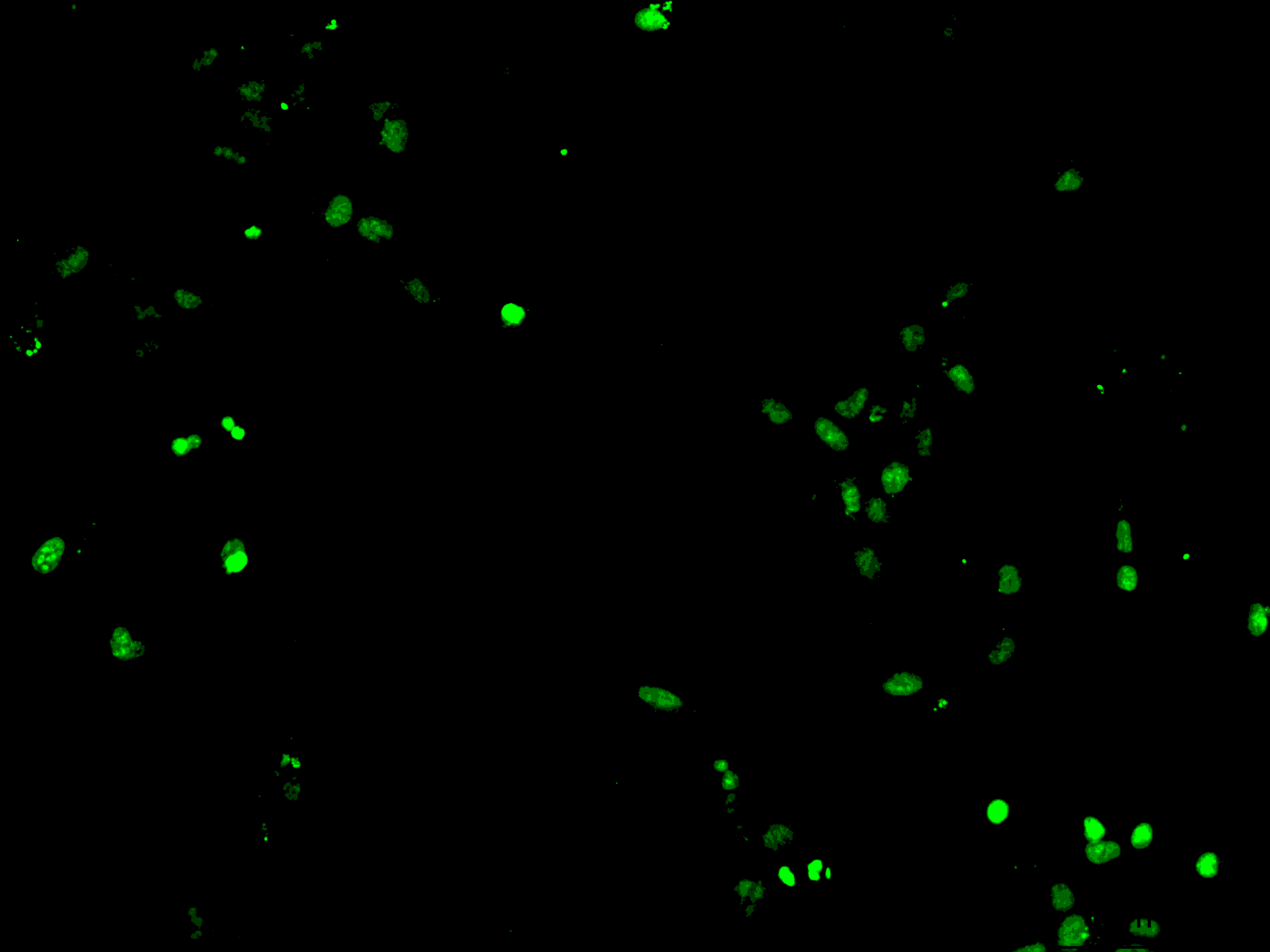

Supplement: Supplementary file 9 [file DataSheet7.zip › Figure 5/Figure 5G/h2o2_31-2.tif]

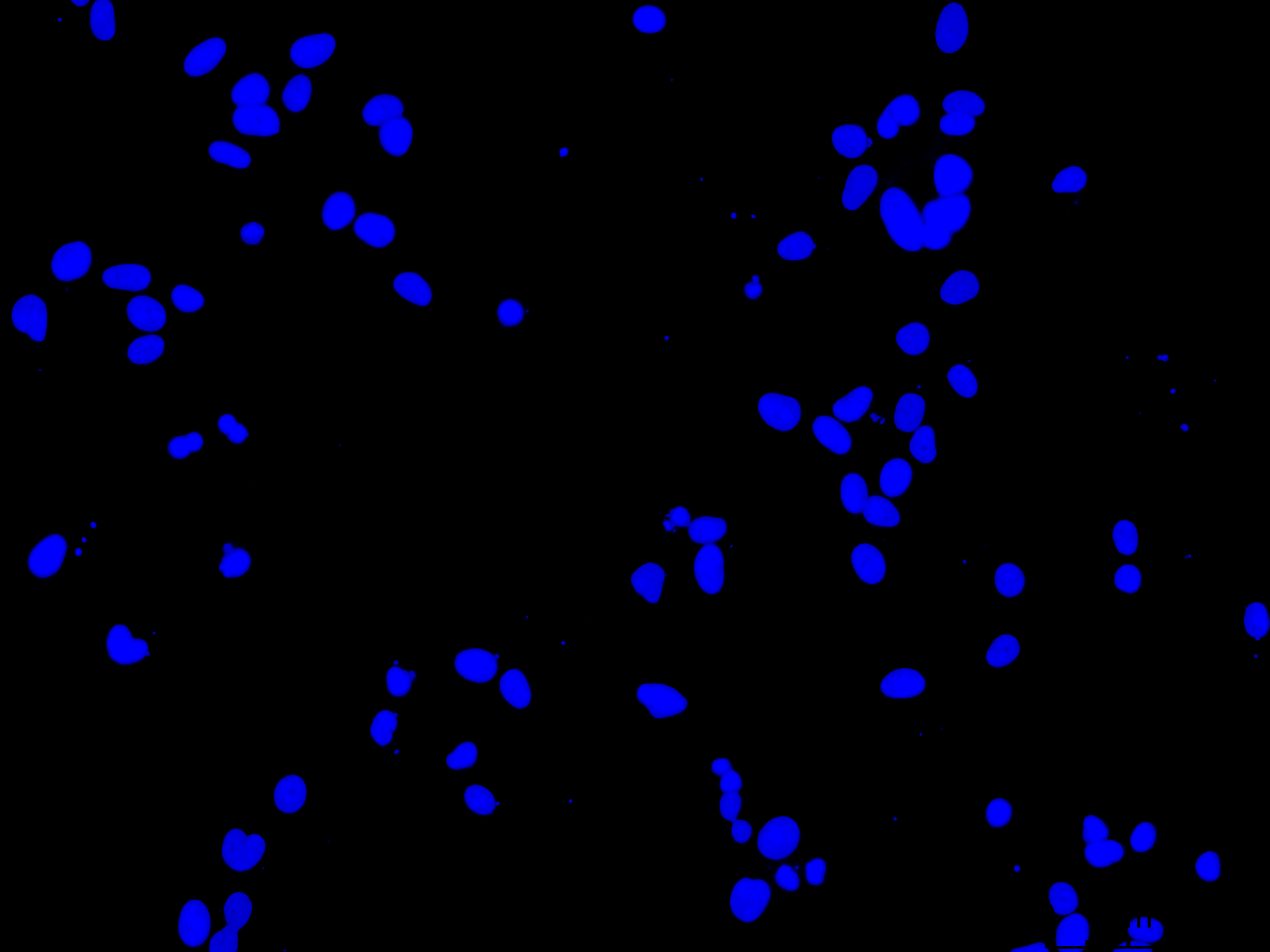

Supplement: Supplementary file 9 [file DataSheet7.zip › Figure 5/Figure 5G/h2o2_31-1.tif]

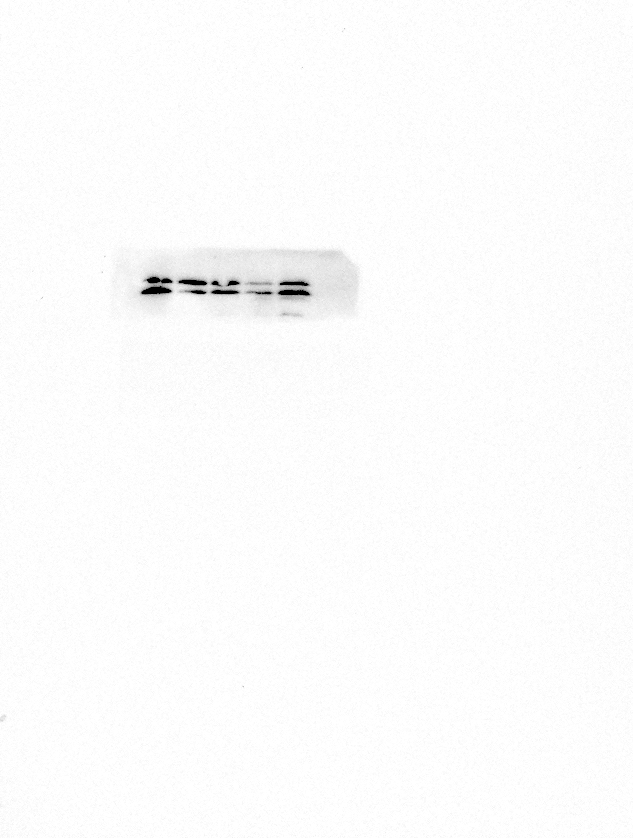

Supplement: Supplementary file 9 [file DataSheet7.zip › Figure 5/Figure 5D/mzb1-1.tif]

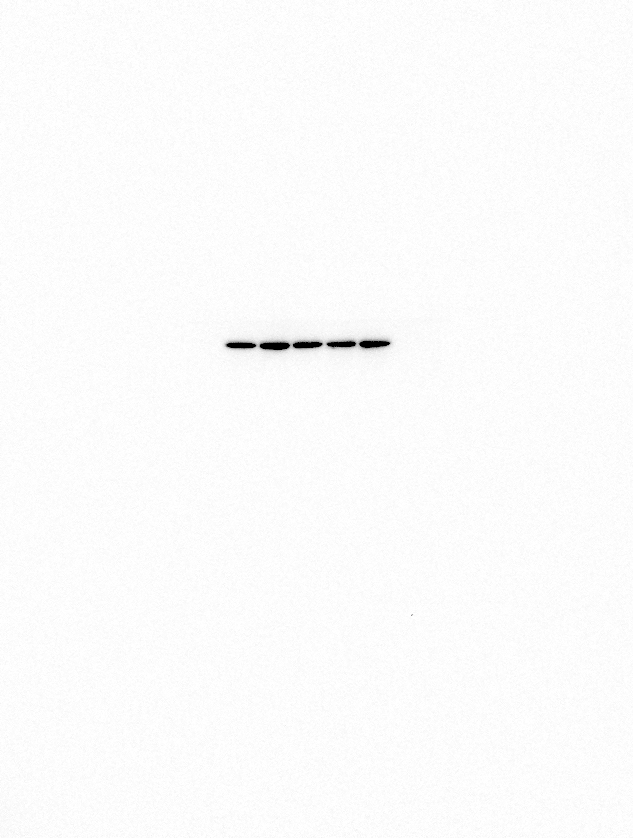

Supplement: Supplementary file 9 [file DataSheet7.zip › Figure 5/Figure 5D/gapdh-1.tif]

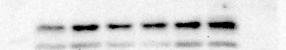

Supplement: Supplementary file 9 [file DataSheet7.zip › Figure 5/Figure 5B/Mzb1-2-Straighten.tif]

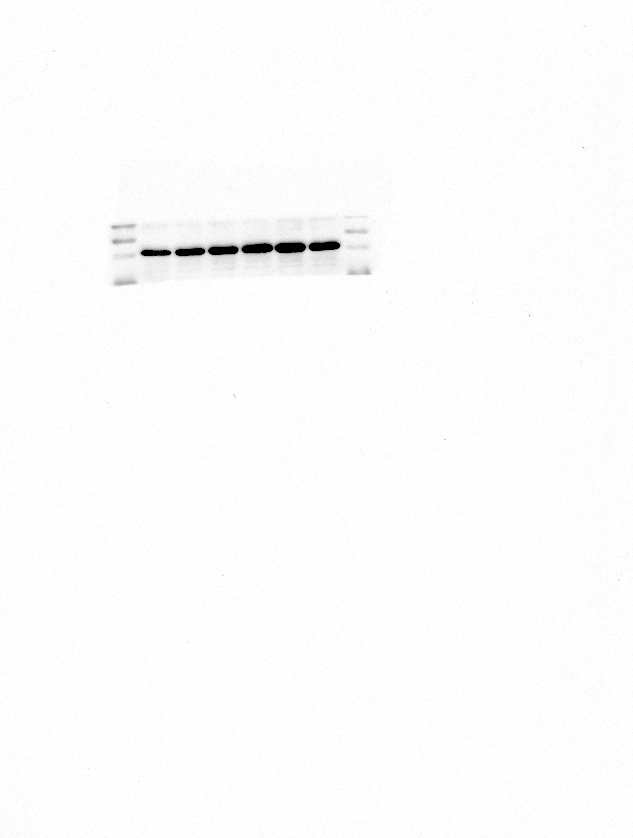

Supplement: Supplementary file 9 [file DataSheet7.zip › Figure 5/Figure 5B/gapdh-2-ctl.vec.h.50.100.200.tif]

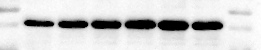

Supplement: Supplementary file 9 [file DataSheet7.zip › Figure 5/Figure 5B/GAPDH-2-Straighten.tif]

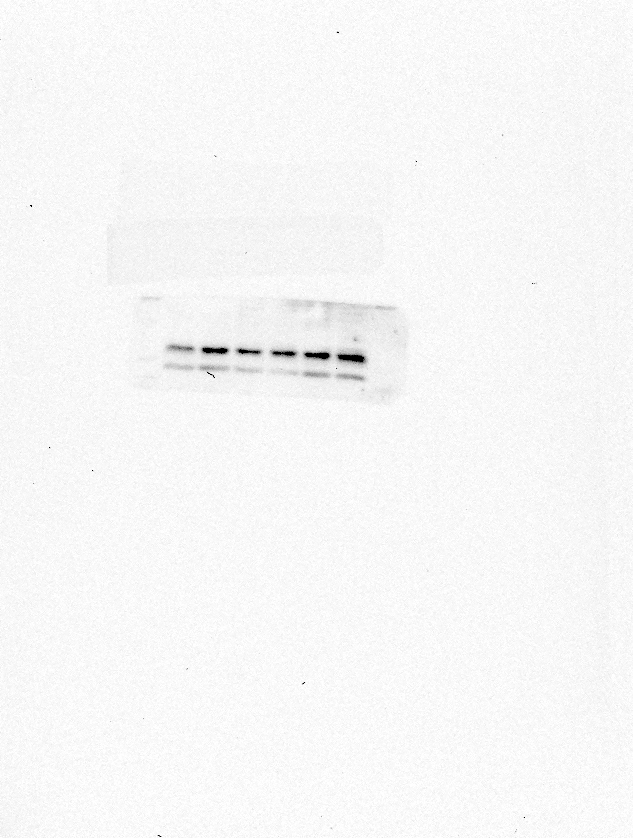

Supplement: Supplementary file 9 [file DataSheet7.zip › Figure 5/Figure 5B/mzb1-2-ctl.vec.h.50.100.200.tif]

Figure 5B

Figure 5B

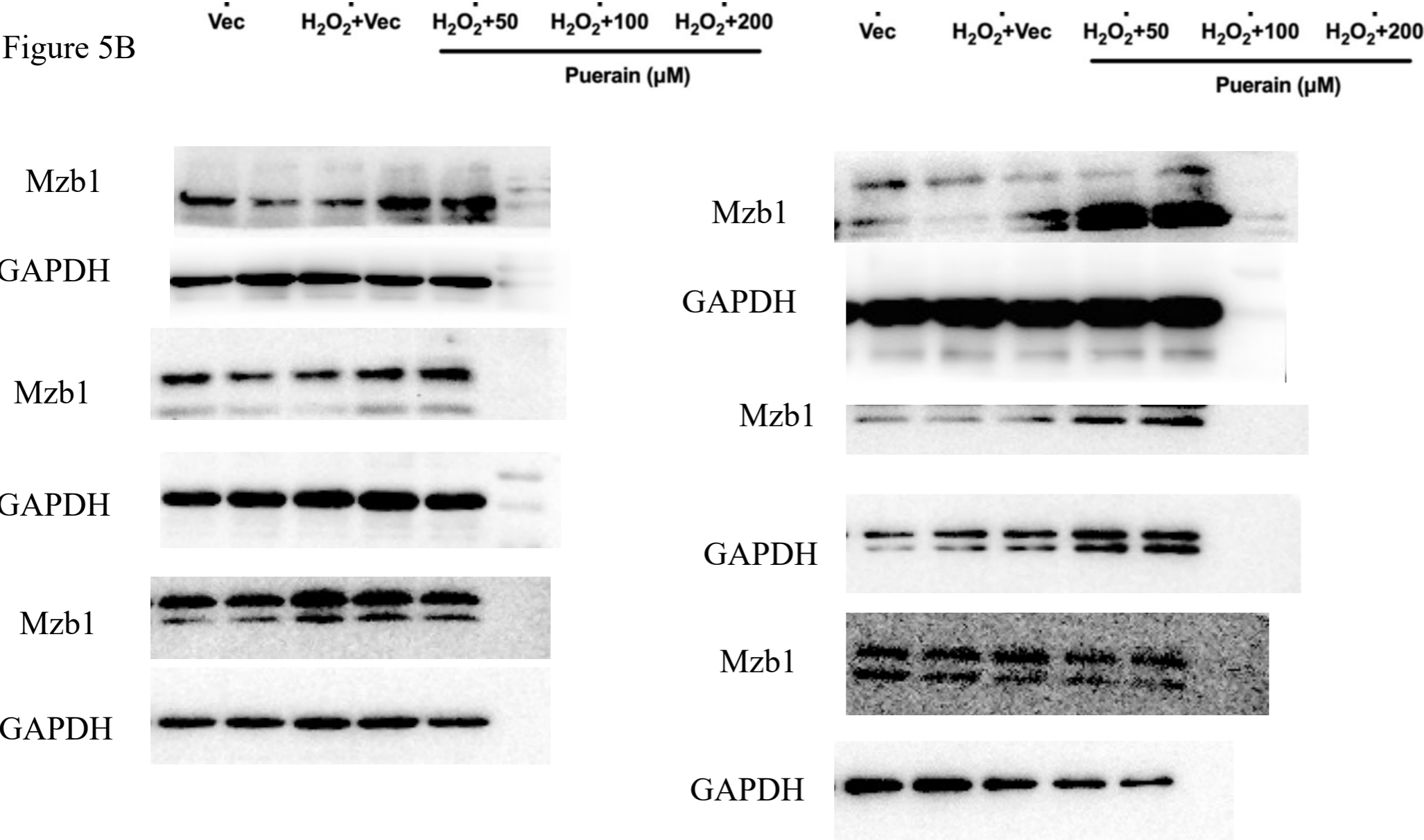

|  | Vec | H <sub>2</sub> O <sub>2</sub> +Vec | H <sub>2</sub> O <sub>2</sub> +50 | H <sub>2</sub> O <sub>2</sub> +100 | H <sub>2</sub> O <sub>2</sub> +200 |
|--|-----|------------------------------------|-----------------------------------|------------------------------------|------------------------------------|
|  | 1   | 0.39                               | 1.4                               | 1.78                               | 2.25                               |
|  | 1   | 0.66                               | 0.87                              | 1.09                               | 1.39                               |
|  | 1   | 0.53                               | 1.18                              | 1.38                               | 1.62                               |
|  | 1   | 0.49                               | 1.09                              | 1.05                               | 1.06                               |
|  | 1   | 0.41                               | 0.65                              | 1.13                               | 1.24                               |
|  | 1   | 0.59                               | 0.68                              | 0.81                               | 1.09                               |

Supplement: Supplementary file 9 [file DataSheet7.zip › Figure 5/Figure 5B/5B.pdf]
