# Supplementary material for: Puerarin attenuates myocardial ischemic injury and endoplasmic reticulum stress by upregulating the Mzb1 signal pathway
Source: Front Pharmacol. 2024 Aug 13;15:1442831. doi: 10.3389/fphar.2024.1442831 (PMC11350615; doi:10.3389/fphar.2024.1442831)
Supplement: Supplementary file 10 [file DataSheet1.docx]

Supplementary Material

# Supplementary Figures


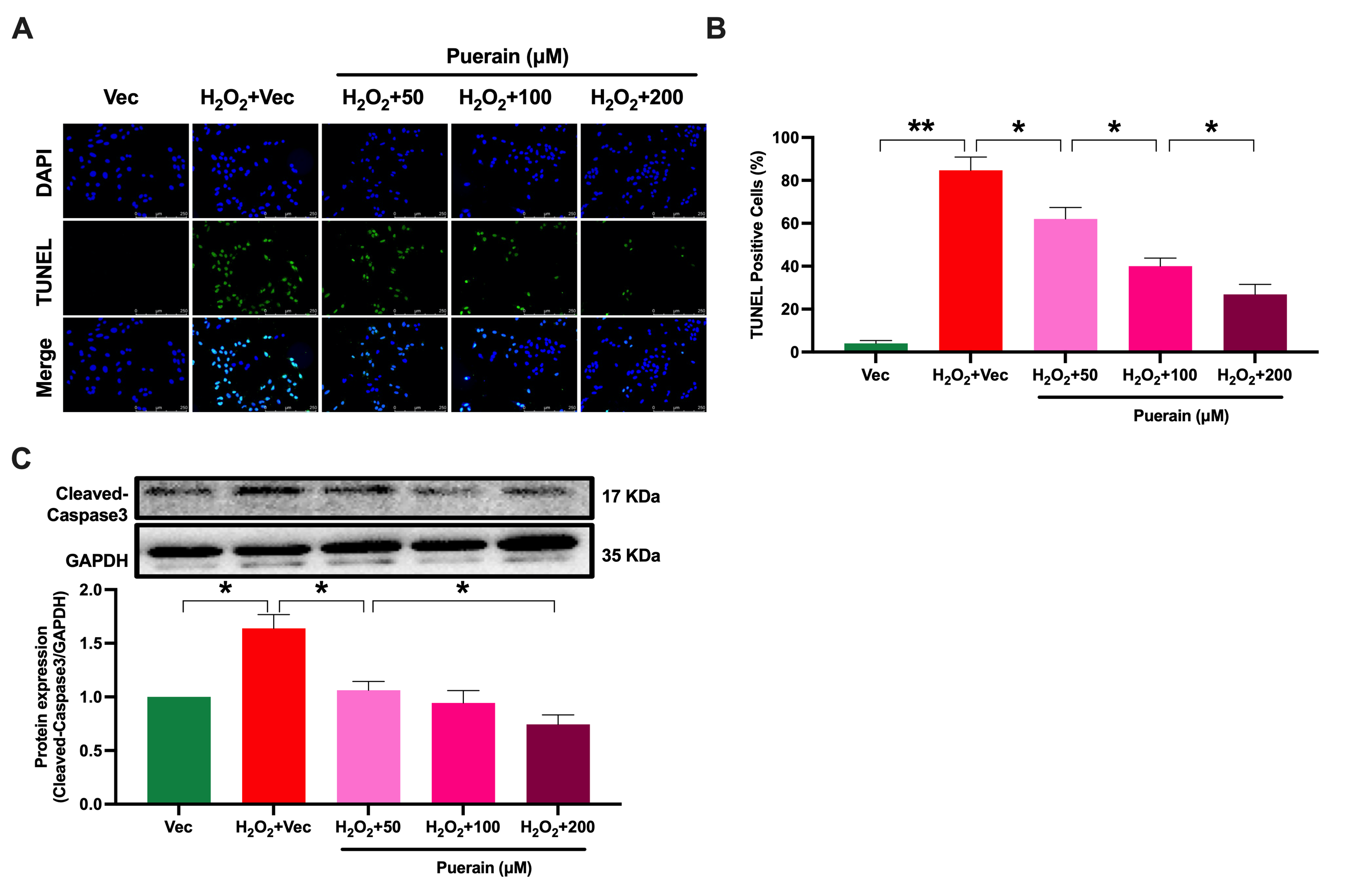


**Supplementary Figure 1. Puerarin dose-dependently decreases apoptosis level in H_2_O_2_-treated cells.** (A) The representative image of TUNEL staining positive cells (magnification 200×, scale bars = 250 μm); (B) The quantitation of TUNEL staining positive cells (n=6); (C) The blot and statistical graph of Cleaved-Caspase 3 *in vitro* (n=5). *p < 0.05; **p < 0.01.

**
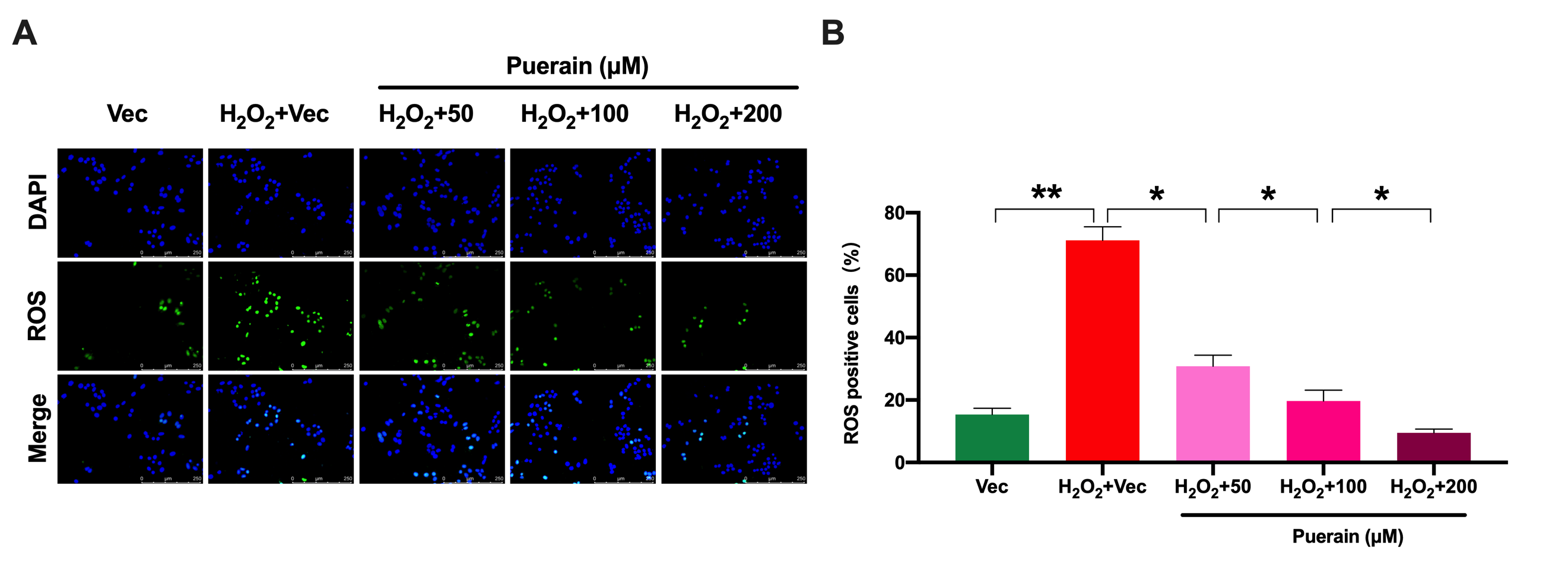
Supplementary Figure 2. Puerarin dose-dependently inhibits ROS level in H_2_O_2_-treated cells.** (A&B) The representative image and statistical graph of ROS production (magnification 200×, scale bars = 250 μm, n=6). *p < 0.05; **p < 0.01.

**
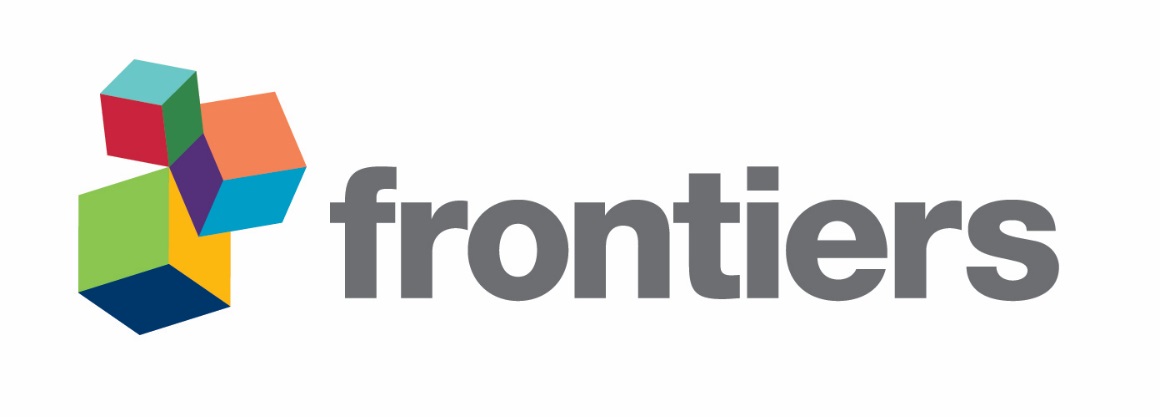
**
